# Mechanistic investigation into the C(sp^3^)–H acetoxylation of morpholinones†
†Electronic supplementary information (ESI) available: Experimental procedures, characterization data and kinetic details. See DOI: 10.1039/c8sc03434f


**DOI:** 10.1039/c8sc03434f

**Published:** 2018-10-01

**Authors:** Cornelia S. Buettner, Darren Willcox, Ben. G. N. Chappell, Matthew J. Gaunt

**Affiliations:** a Department of Chemistry , University of Cambridge , Lensfield Road , Cambridge , UK . Email: mjg32@cam.ac.uk

## Abstract

The study of a selective palladium(ii)-catalyzed C(sp^3^)–H acetoxylation reaction on a class of cyclic alkyl amines is reported. Computational modelling and kinetic studies were used to provide support for a mechanism involving selective C–O bond formation from a γ-aminoalkyl-Pd(iv) intermediate. The C–O bond forming step was computed to occur by a dissociative ionization mechanism followed by an S_N_2 process involving external acetate attack at the C–Pd(iv) bond. This pathway was computed to be of lowest energy with no competing C–N products observed. Additionally, with a few modifications to reaction conditions, preliminary studies showed that this process could be rendered enantioselective in the presence of a non-racemic BINOL-phosphoric acid.

## Abstract 2

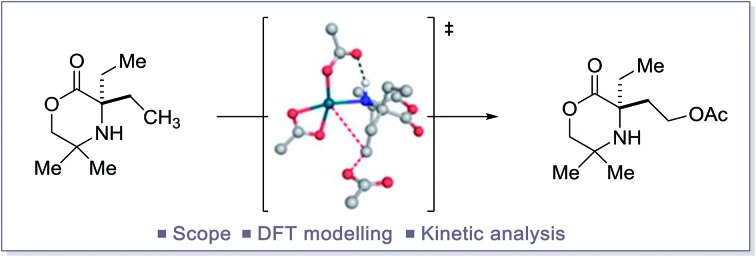
The study of a selective palladium(ii)-catalyzed C(sp^3^)–H acetoxylation reaction on a class of cyclic alkyl amines is reported.

## 

The reductive elimination from transient palladium(iv) species has enabled a range of carbon–heteroatom bond forming processes,^1^ particularly in the area of C(sp^3^)–H bond functionalization. Of these transformations, palladium catalyzed C(sp^3^)–H acetoxylation has been the focus of significant study.^2^ However, despite the report of an increasing number of catalytic processes, mechanistic understanding of the reductive elimination from the transient alkyl-palladium(iv) species has remained obscure. Important stoichiometric studies, by Sanford and co-workers, on C–H acetoxylation has led to three distinct mechanistic rationales being proposed for the reductive elimination pathway: (1) direct reductive elimination from palladium(iv) without loss of a ligand; (2) dissociative neutral (D_N_) where a L-type ligand dissociates to form a neutral five-coordinate palladium(iv) intermediate followed by reductive elimination and (3) dissociative ionization (D_I_), where a X-type ligand dissociates forming a five-coordinate cationic palladium(iv) species prior to reductive elimination (Scheme 1a).^3^ Further investigations and DFT modelling studies into C(sp^3^)–H acetoxylation reactions, using model palladium(iv) intermediates, indicated that a dissociative ionization mechanism is the major pathway for carbon–oxygen reductive elimination (Scheme 1b).^4^

**Scheme 1:**
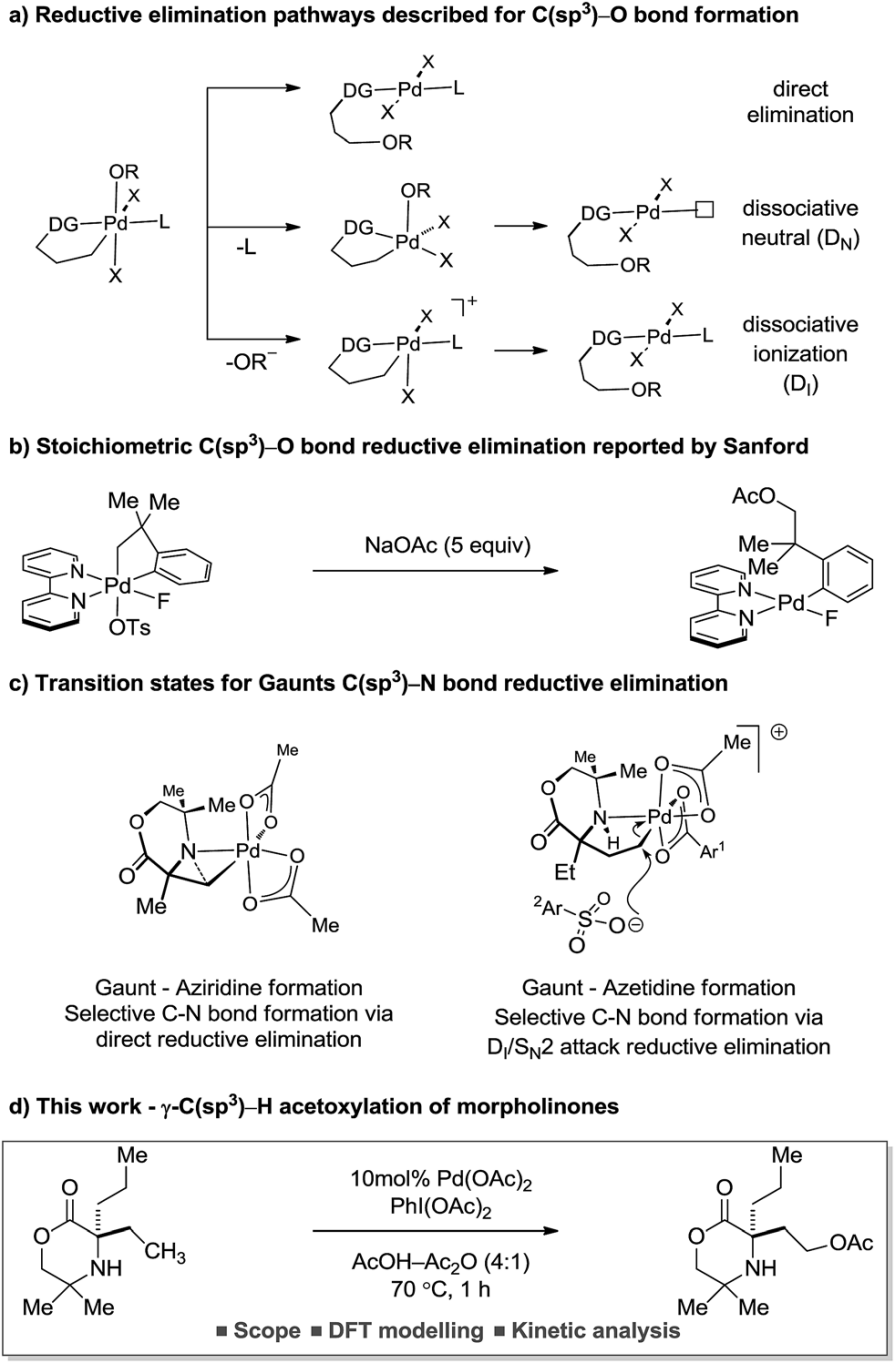
Mechanistic studies conducted for carbon–heteroatom bond formation.

Our group has a long standing interest in the development of processes founded on palladium(ii)-catalyzed free(NH) alkylamine-directed C(sp^3^)–H activation. One aspect of this work has involved the deployment of oxidants to access aminoalkyl-Pd(iv) intermediates, from which reductive elimination can take place to form carbon–heteroatom bonds. While we have reported a number of selective carbon–nitrogen bond formation reactions for the synthesis of both aziridines and azetidines,^5^ the development of carbon–oxygen bond forming processes has been hindered by poor selectivity in the (product forming) reductive elimination step. Mechanistic studies into the β-C(sp^3^)–H amination of alkylamines to form aziridines, facilitated by iodosobenzene diacetate as oxidant, identified that the process proceeded *via* a direct C–N bond forming reductive elimination from the aminoalkyl-palladium(iv) intermediate that was triggered by deprotonation of the amine by an internal acetate.^6^ In contrast, we found that the corresponding γ-C(sp^3^)–H amination to generate the azetidine product required the use of a benziodoxole tosylate oxidant. Interestingly, DFT studies identified that carbon–nitrogen bond formation occurred *via* a two-step dissociative ionization pathway involving loss of a sulfonate group from a palladium(iv) species followed by its S_N_2 attack at the carbon–palladium(iv) bond to form a γ-C–OTs bond and finally internal displacement of the tosylate to form the 4-membered ring amine (Scheme 1c). Here, we report that controlling the mechanism of the reductive elimination process has enabled the development of a palladium(ii)-catalyzed γ-C(sp^3^)–H acetoxylation process on a class of cyclic amines (that we call morpholinones). We detail our preliminary mechanistic and computational studies that support the putative two-step dissociative pathway for C–O bond formation, optimize and explore the scope of the reaction, and, finally, identify that the γ-C(sp^3^)–H acetoxylation reaction can be rendered enantioselective using chiral anionic ligands.

## Results and discussion

**Scheme 2:**
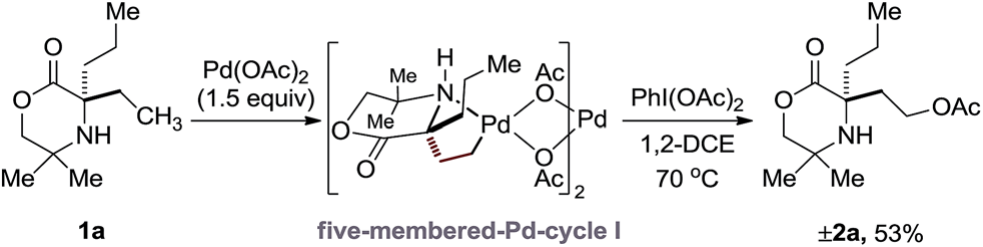
Stoichiometric acetoxylation of morpholinone **1a**.

At the outset of our studies, we had observed that the treatment of amine **1a** with Pd(OAc)_2_ and PhI(OAc)_2_ produced no trace of azetidine product, instead undergoing exclusive γ-C(sp^3^)–H acetoxylation to form **2b** in 48%.5*a* Based on this initial result, we confirmed the fundamental features of the mechanism by conducting a stoichiometric reaction with morpholinone **1a** and Pd(OAc)_2_; stirring amine **1a** in the presence of 1.5 equivalents of PdI(OAc)_2_ at 60 °C in a chloroform solution gave the anticipated γ-aminoalkyl-palladacycle and treatment with PhI(OAc)_2_ in 1,2-DCE afforded the expected C–H acetoxylation product in 53% yield (Scheme 2).

Next, we assessed the reaction conditions required for a catalytic process. First, a simple survey of reaction parameters (Table 1) revealed that acetic acid in the presence of Ac_2_O was the optimal media for the process, producing **2a** in 68% yield (entries 1–3). The Ac_2_O sequestered any water in the reaction mixture. Varying the temperature afforded no significant change in yield (entry 4). However, decreasing the reaction time from 5 h to 3 h allowed formation of **2a** in 72% yield (entry 5), suggesting that the acetoxylated amine **2a** was not indefinitely stable under the reaction conditions. Increasing the catalyst loading from 5 mol% to 10 mol% led to a further increase in yield to 82% (entry 6). Time course experiments indicated that the reaction was completed within 1 h with 10 mol% catalyst (entry 7). Adjusting the equivalents of PhI(OAc)_2_ had no effect on the outcome of the reaction (entries 8 and 9). In conclusion, optimal conditions for the γ-C(sp^3^)–H acetoxylation were found to involve treatment of **1a** with 10 mol% Pd(OAc)_2_ as catalyst and 1.5 equivalents of PhI(OAc)_2_ in a 0.1 M solution of AcOH/Ac_2_O (4 : 1) at 70 °C for one hour, which afforded a 75% yield of isolated amine product after chromatography.

**Table 1:** Standard reaction conditions: 5 mol% Pd(OAc)_2_, 1.5 equiv. PhI(OAc)_2_, solvent/Ac_2_O (4 : 1, 0.1 M)

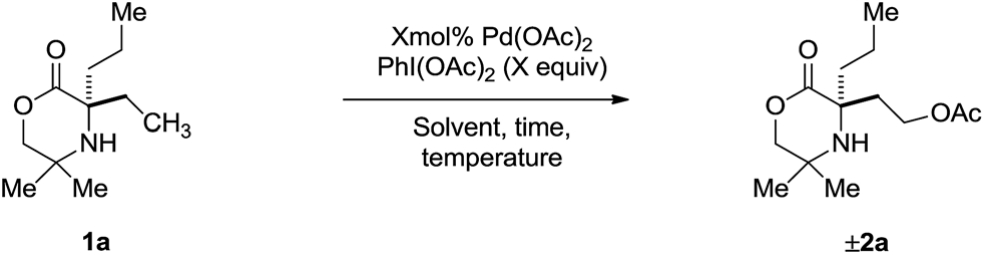				
Entry	Solvent	Reaction time	Temperature	Yield^*a*^
1	PhMe	5 h	60 °C	52%
2	DCE	5 h	60 °C	41%
3	AcOH	5 h	60 °C	68%
4	AcOH	5 h	70 °C	66%
5	AcOH	3 h	70 °C	72%^*b*^
6	AcOH^*c*^	3 h	70 °C	82%
7	AcOH^*c*^	1 h	70 °C	75%^*b*^
8	AcOH^*c*^ ^,^^*d*^	1 h	70 °C	69%
9	AcOH^*c*^ ^,^^*e*^	1 h	70 °C	72%

^*a*^Yield determined by ^1^H-NMR spectroscopy using 1,1,2,2-tetrachloroethane as an internal standard.

^*b*^Yield of isolated product.

^*c*^10 mol% Pd(OAc)_2_ loading.

^*d*^1.0 equiv. PhI(OAc)_2_.

^*e*^2.0 equiv. PhI(OAc)_2_.

With optimized conditions in hand, we turned our attention to investigating the mechanism of this (Csp^3^)–H acetoxylation process. Based on previous investigations of C–H activation reactions on morpholinone scaffolds, we envisaged a similar pathway for the C–H activation step *via* a mono-amino palladium(ii) complex that proceeds through a CMD-type mechanism. Oxidation of the resulting γ-aminoalkyl-palladacycle, with PhI(OAc)_2_, to generate the corresponding palladium(iv) intermediate primes the complex for the C–O bond forming step to furnish the desired acetoxylated amine product. The nature of the reductive elimination step could be in line with that observed in the C–H amination to aziridines (direct C–O bond reductive elimination) or *via* the two-step dissociative ionization/S_N_2 pathway indentified in our azetidine forming reaction. To this end, both *in vitro* and *in silico* mechanistic studies were investigated to help to enlighten the mechanism of this process.

**Fig. 1:**
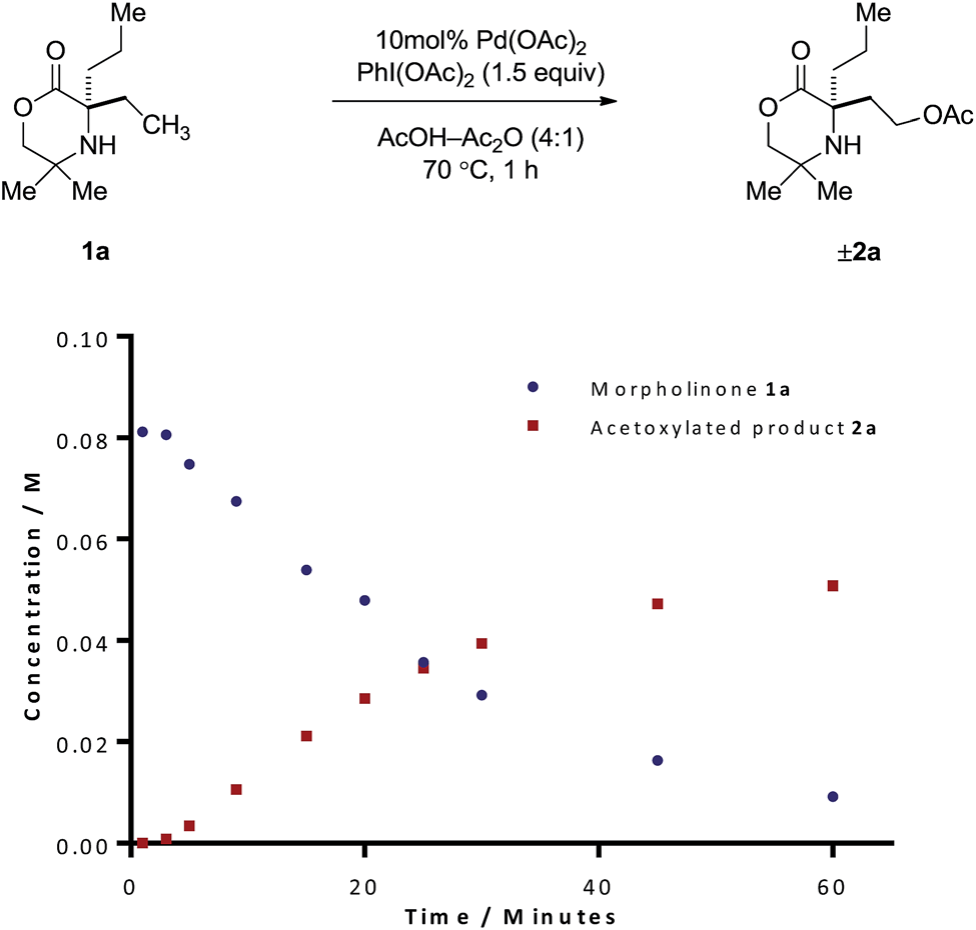
Reaction profile for the acetoxylation of morpholinone **1a**.

Our procedure to explore the kinetics of this reaction featured experiments to probe the reagent concentration dependencies and isotopic labelling studies to interrogate the mechanism of the stoichiometric reactions in Scheme 2. The process was monitored by taking aliquots from the reaction mixture at specific time intervals and measuring the concentration with 1,1,2,2-tetrachloroethane as internal standard by Flame ionization detector-gas chromatography (FID-GC). The reaction conditions involved the treatment of morpholinone **1a** with 10 mol% Pd(OAc)_2_ formula, 1.5 equivalents of PhI(OAc)_2_ in AcOH/Ac_2_O (4 : 1) solvent mixture at 70 °C. These conditions led to a rate profile which enabled us to follow the whole reaction over a 1 hour time scale (Fig. 1).

Due to the linear nature of the kinetic profile at the beginning of the reaction, the initial rates may be determined from the gradient of the concentration profile during this period and used to obtain the order in reagents. We first began by determining the order with respect to PhI(OAc)_2_. Based on the rates obtained from reactions containing between one and three equivalents of PhI(OAc)_2_ (Table S1, ESI†), the reaction exhibited zero-order kinetics with respect to the oxidant, indicating that the oxidation of the palladacycle occurs after the turnover limiting step (TOLS). By comparing the initial rates, we were also able to determine, from a concentration *vs.* 1/[**1a**] plot, a reaction order of –1 for the amine component (Table S3, ESI†). We propose that negative order in amine arises from the formation of an off-cycle bisamine complex at higher amine concentration. At lower amine concentrations, the mono/bis-amine equilibrium lies towards the mono coordinated amine complex, thus enabling C–H activation to proceed, analogous to that observed in our previous work.^6^ Determination of the order with respect to Pd(OAc)_2_ under first-order conditions showed saturation type kinetics, however a plot of ln[Pd(OAc)_2_] *vs.* ln(rate) reveals an order in Pd(OAc)_2_ of 0.31 (Table S2, ESI†). This can be explained due to (1) Pd(OAc)_2_ existing in a trimeric form in AcOH, (2) the slow dissociation of this trimer into the reactive monomer, even at elevated temperatures and (3) there being insufficient free amine to efficiently break down this trimer due to the amine being fully protonated under the reaction conditions. The combination of these factors should lead to the observed order of 0.33.^7^ The dissociation of this palladium acetate-trimer to the monomeric species could also account for the induction period for starting material consumption observed in Fig. 1.

**Scheme 3:**
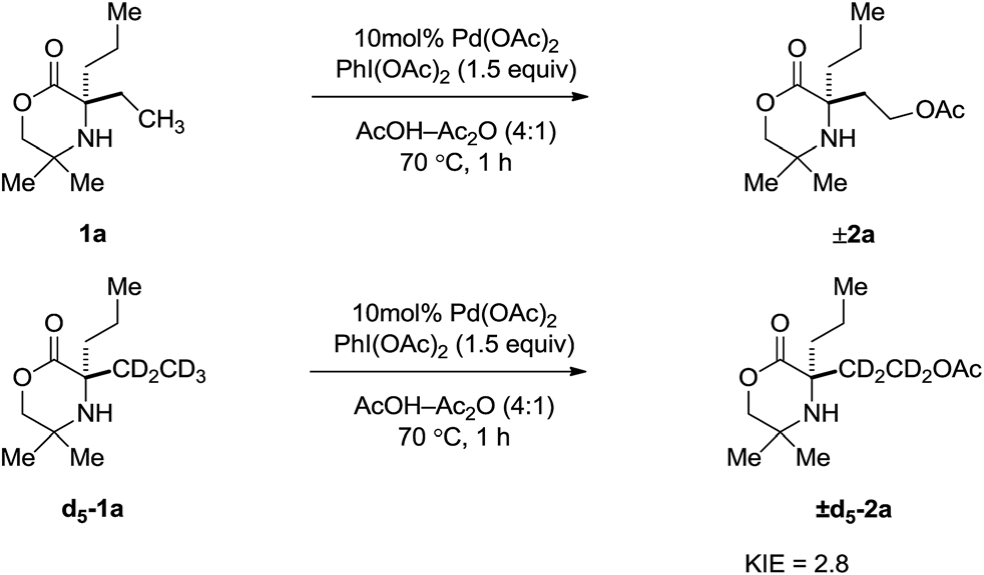
KIE measured from initial rate comparisons of **1a** and d_5_-**1a**.

Further kinetic information was obtained by measurement of the kinetic isotope effect (KIE). A KIE was determined from initial rate measurements of substrate **1a** and d_5_-**1a** (Scheme 3). A primary kinetic isotope effect of 2.8 was obtained, suggesting the C–H bond cleavage occurs as part of the TOLS.^8^

To test whether the acetoxylated product **2a** was inhibiting the reaction or leading to catalyst degradation, same “excess” experiments were performed.^9^,^10^ Starting at 20% completion, time adjusting these results and overlaying onto the 0% completion plot (equivalent initial amine concentrations), indicated no product inhibition or catalyst deactivation (Table S4, ESI†). The kinetic data obtained in this study agrees with our previously reported mechanistic work on C–H activation of morpholinones^6^ and so we envisage that the exclusive formation for the acetoxylated product must result from a difference in reductive elimination mechanism being in operation. As the reductive elimination step appears to take place after the rate-limiting C–H bond activation and the high reactivity of the γ-aminoalkyl-palladium(iv) intermediate, elucidation of the mechanism in this part of the catalytic cycle is challenging through experimental means.

Accordingly, DFT studies were conducted to interrogate the energetically favoured pathway for the γ-C(sp^3^)–H acetoxylation reaction using **1b** as a model substrate. The calculations were performed on Amsterdam Density Functional (ADF) software, using ZORA-BLYP-D3 which has been used previously for palladium catalysed reactions and more specifically for the palladium catalysed C–H activation of amines within our group.5d,^6^,^11^,^12^ The solvent effects were considered using an implicit conductor like solvation model (COSMO) in dichloroethane.

**Fig. 2:**
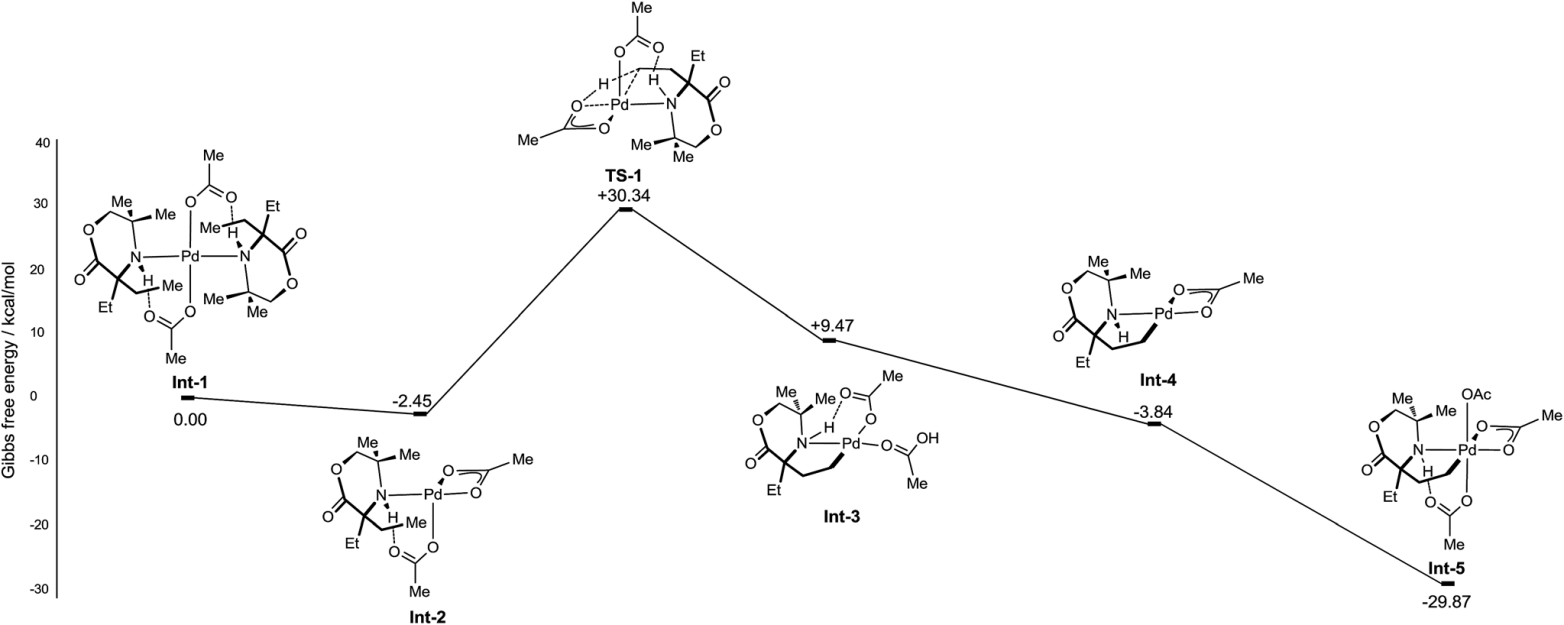
Computed CMD C–H activation mechanism of morpholinones.

Initially, the C–H activation/oxidation sequence of to generate **Int**-**5** from the mono-coordinate amino-complex was explored to enable a comparison with the kinetics results presented previously (Fig. 2). Initial dissociation of a single molecule of **1b** from **Int-1** led to the formation energetically favourable mono-amine complex **Int-2** (–2.45 kcal mol^–1^ lower than **Int-1**). From mono-amine complex **Int-2**, C–H activation proceeds through the expected six membered CMD transition state **TS1**, which was found to be +27.89 kcal mol^–1^ above **Int-2**. The palladium(ii) complex **Int-3** then underwent dissociation of an acetate ligand to form the γ-aminoalkyl-palladacycle with a κ^2^-bound acetate group (–13.31 kcal mol^–1^, **Int-4**). Oxidation of **Int-4** with PhI(OAc)_2_ yields the key γ-aminoalkyl-palladium(iv) complex **Int-5**.

**Fig. 3:**
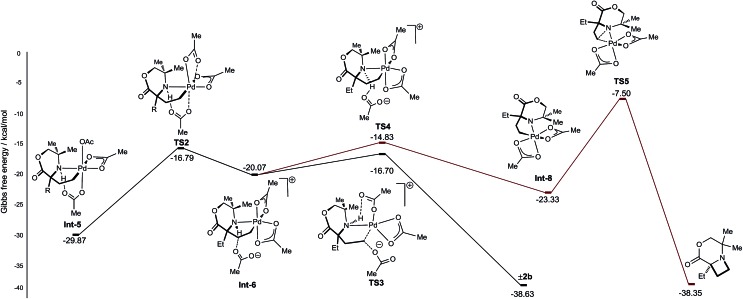
Calculated energy barriers of C–N and C–O reductive elimination.

From γ-aminoalkyl-palladium(iv) complex **Int-5**, the chemoselectivity of the reductive elimination process towards the formation of the C–O (acetoxylation) or C–N (azetidine) products was explored (Fig. 3). For the C–O bond formation product **2b**, the lowest energy pathway involves the full dissociation of the hydrogen bonded acetate leading to **Int-6**, *via* transition state **TS2**, which was found to be +13.08 kcal mol^–1^ above **Int-5**. Intermediate **Int-6** then undergoes attack by an external acetate at the electrophilic C–Pd(iv) bond to form the key C–O bond (+3.37 kcal mol^–1^ above **Int-6**). After the S_N_2-type process, the amine remains bound to the reduced palladium(ii) complex and upon de-ligation yields the acetoxylated product **2b**.

For azetidine formation to occur, **Int**-**6**, containing two κ^2^-bound acetate groups, would be required to undergo deprotonation by an external acetate (**TS4**) resulting in the amido-Pd(iv) complex **Int-8**. From this complex, C–N bond forming reductive elimination can occur *via***TS5** to give the azetidine product. We computed **TS5** to be +13.83 kcal mol^–1^ above **Int-8**. Therefore, we rationalise the exclusive C–O bond formation due to the significant energy barrier of C–N reductive elimination from complex **Int-8**.

**Fig. 4:**
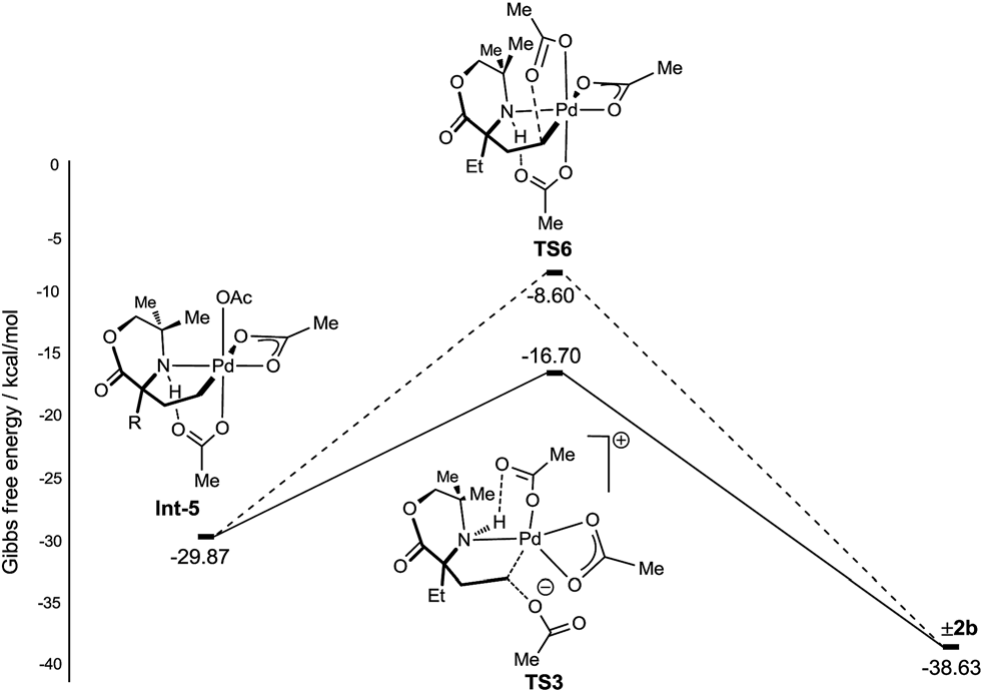
Reductive elimination sequences for the C–O bond formation from **Int-5**.

With a rationale in hand for the chemoselectivity of C–O bond formation, we explored other potential pathways of classical reductive elimination from the γ-aminoalkyl-palladium(iv) intermediate **Int-5**. Aside from external attack at the C–Pd(iv) bond, direct reductive elimination (transition state **TS6**) from the γ-aminoalkyl-palladium(iv) complex was computed to have a significantly greater energy barrier of +21.47 kcal mol^–1^. The C–O reductive elimination processes from **Int-5** involving both the κ^2^-bound, as well as the hydrogen bonded, acetate ligand was examined. However, these proved to be even higher in energy (see ESI† for details) (Fig. 4).

**Scheme 4:**
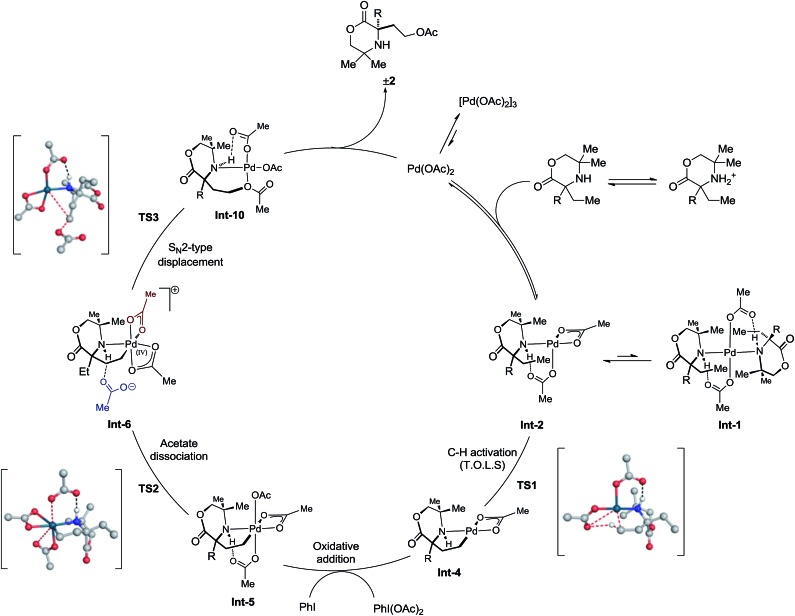
Final elucidated catalytic cycle.

Consolidation of the kinetic data with the DFT modeling allows a more complete mechanism to be proposed for the C(sp^3^)–H acetoxylation (Scheme 4). The amine first coordinates to Pd(OAc)_2_ formula to afford the mono-amine complex **Int-2**. This species is then capable of coordinating a further amine, to form the off-cycle bis-amine complex **Int-1**, or can undergo intramolecular γ-C–H activation to form the 5-membered cyclopalladated species **Int-4**, *via***TS1**. This intermediate then undergoes oxidation by PhI(OAc)_2_ to form γ-aminoalkyl-palladium(iv) species **Int-5**. Dissociation of an acetate ligand (**TS2**) precedes an S_N_2-type displacement (**TS3**) from palladium(iv) species **Int-6** by the hydrogen-bonded acetate anion to generate the product ligated to Pd(OAc)_2_, which upon decomplexation delivers the desired product **2** and regenerates Pd(OAc)_2_ to renter the catalytic cycle.

**Scheme 5:**
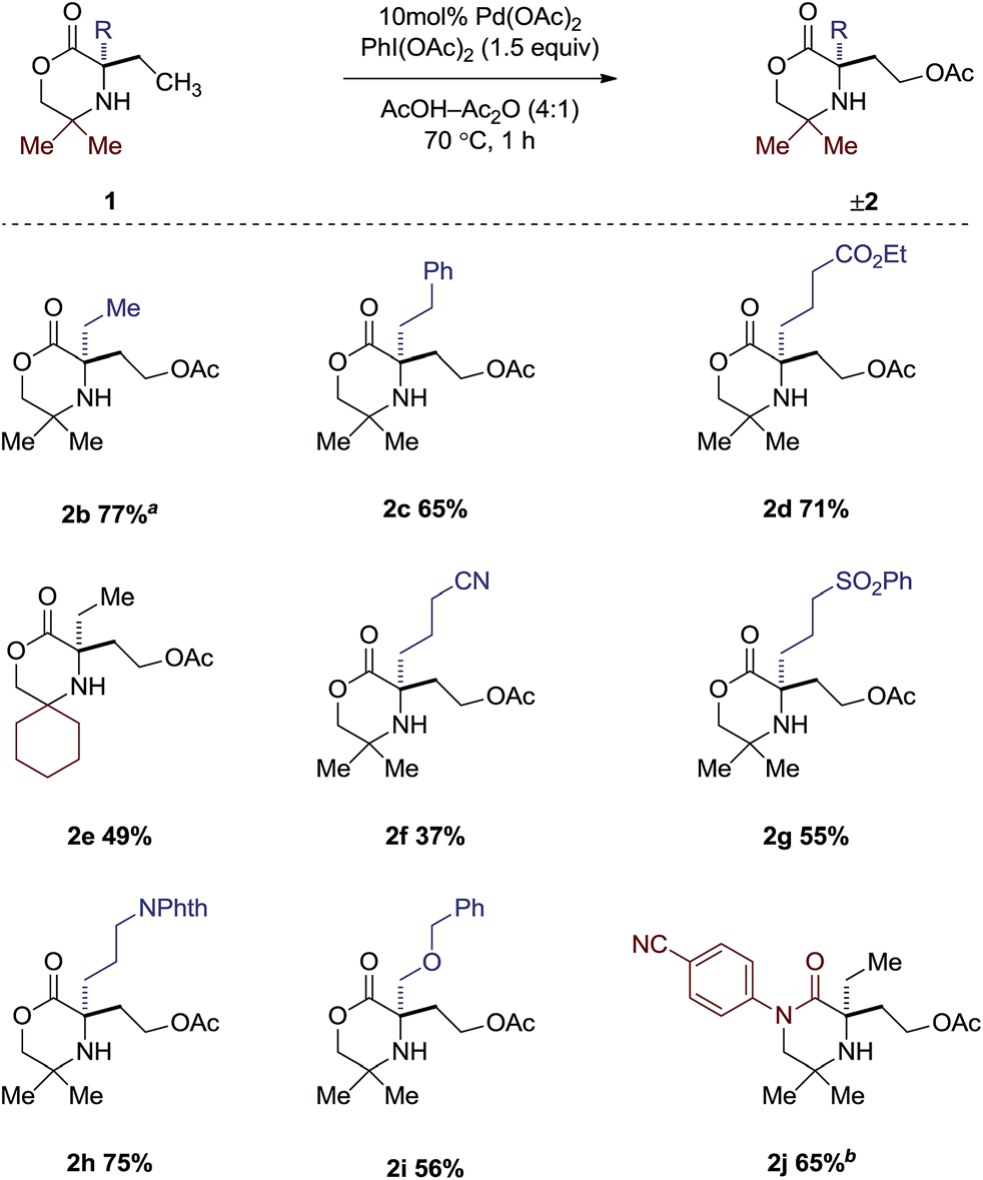
Scope of acetoxylated morpholinones. ^*a*^ Isolated as a mixture of mono- and diacetoxylated products (1.6 : 1). ^*b*^ 15 mol% Pd(OAc)_2_ in CH_2_Cl_2_–Ac_2_O (4 : 1).

Having gained a clearer understanding of the mechanism of the γ-C–H acetoxylation process, we briefly explored the scope of the new reaction. We found that simple alkyl substituents on the reacting side of the morpholinone scaffold were tolerated affording the corresponding acetoxylated products **2b–j** in good yield (Scheme 5). When di-ethylated compound **1b** was used, 77% product as a 1.6 : 1 ratio of mono- to di-acetoxylated was observed. A range of functional groups were also tolerated in moderate to good yields such as esters (**2d**), sulfones (**2g**) and nitriles (**2f**), as well as protected alcohols (**2h**) and amines (**2i**). It is interesting to note that changing the *gem*-dimethyl groups on the non-reacting side for the spirocyclic cyclohexyl group affords only the mono-acetoxylated product (**2e**), albeit in 49% yield. Switching from the morpholinone scaffold to the piperazidinone scaffold (**1j**) required a slight modification of reaction conditions. It was found that when AcOH was used, a 1 : 1 mixture of mono- and di-acetoxylation was observed in 43% yield. However, a slight increase in catalyst loading, coupled with using dichloromethane as solvent, afforded the desired mono-acetoxylated product in 65% yield.

**Scheme 6:**
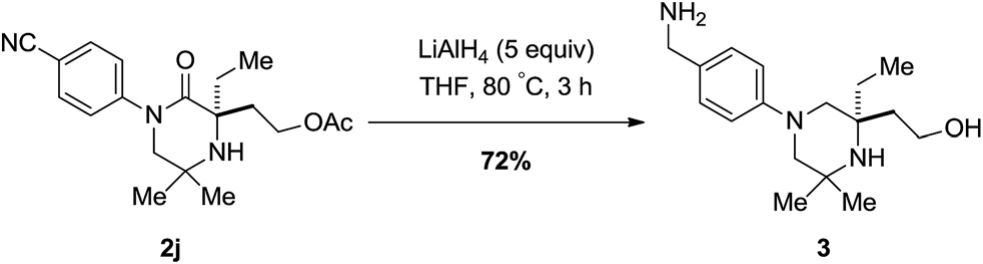
Reduction of piperazidinone ±**2j**.

To highlight a simple application, piperazidinone **2j** could be reduced with lithium aluminium hydride afforded the corresponding piperazine bearing both a primary amine moiety and alcohol in 72% yield; heavily substituted piperazine scaffolds are difficult to form by other means (Scheme 6).^13^

Finally, in light of the mechanistic studies conducted above, we investigated the potential for an asymmetric C–H acetoxylation process. We reasoned that with the C–H activation step being a part of the TOLS, that this should also be enantio-determining. From Int-2 in the catalytic cycle, we envisage that a chiral hydrogen bond acceptor ligand could induce asymmetry in the C–H activation step. Based on our work on asymmetric C–H amination to aziridines, we assessed a selection of chiral phosphoric acid ligands^14^ under various reaction conditions (Table 2). We found that using the optimized AcOH/Ac_2_O solvent mixture lead to high yields, but racemic, product formation (entry 1). A solvent screen (entries 2–5) indicated that dichloromethane could be a suitable solvent for an enantioselective acetoxylation returning the product in 56% with 53 : 47 enantiomeric ratio (e.r.). Encouraged by this initial finding, we switched oxidant system to the I_2_/AgOAc oxidant system^13^ and found the desired product was obtained in 39% yield with 85 : 15 e.r. (entry 6). Using (*R*)-H_8_-TRIP **2b** could be obtained in 33% yield, but with a decreased e.r. of 75 : 25. The results presented herein represent a rare example of catalytic enantioselective C(sp^3^)–H acetoxylation and provide an exciting starting point for further development.

**Table 2:** Initial results towards an enantioselective C–H acetoxylation of morpholinones

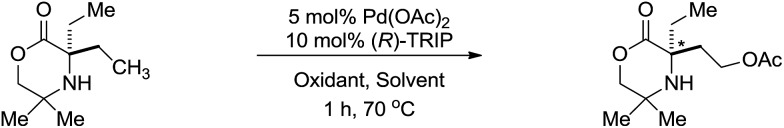				
Entry	Oxidant	Solvent	Yield (%)	e.r.
1	PhI(OAc)_2_	AcOH/Ac_2_O	70	50 : 50
2	PhI(OAc)_2_	MeNO_2_	42	50 : 50
3	PhI(OAc)_2_	EtOAc	—	—
4	PhI(OAc)_2_	1,2-DCE	—	—
5	PhI(OAc)_2_	CH_2_Cl_2_	56	53 : 47
6	I_2_/AgOAc	CH_2_Cl_2_	39	85 : 15
7	I_2_/AgOAc	CH_2_Cl_2_	33	75 : 25

In summary, we have developed a palladium-catalyzed C–H acetoxylation of aliphatic amines using PhI(OAc)_2_ as oxidant in AcOH/Ac_2_O solvent system. This process transforms readily available amine motifs into highly functionalized amino-alcohol derivatives. The mechanism of this C(sp^3^)–H acetoxylation has been elucidated by detailed DFT and kinetic studies. These studies reveal the reaction proceeds *via* rate limiting C–H activation from the mono-amine complex. After oxidation of the 5-membered ring cyclopalladation complex, a dissociative ionization/S_N_2-type reductive elimination sequence is responsible for the exclusive C(sp^3^)–O bond formation product. Finally, nonracemic binol-phosphoric acid ligands were assessed for the induction of enantioselectivity in this transformation and an 85 : 15 e.r. was observed using (*R*)-TRIP and a modified oxidant system. We envisage this as a viable starting point for further development.

## Conflicts of interest

There are no conflicts to declare.

## Supplementary Material

Supplementary information
