# Supplementary material for: Mechanistic investigation into the C(sp3)–H acetoxylation of morpholinones
Source: Chem Sci. 2018 Oct 1;10(1):83–9. doi: 10.1039/c8sc03434f (PMC6331033; doi:10.1039/c8sc03434f)
Supplement: Supplementary file 1 [file SC-010-C8SC03434F-s001.pdf]

Electronic Supplementary Material (ESI) for Chemical Science.
This journal is © The Royal Society of Chemistry 2018

General Experimental

Solvents were distilled using standard procedures. Petroleum ether is 40-60 b.p. unless stated otherwise. Reagents were used as supplied using standard procedures. Palladium(II) acetate was purchased from Alfa Aesar. All flash chromatography was carried out using Merck 9385 Kieselgel 60 silica gel under a positive pressure of nitrogen. Thin layer chromatography (TLC) was carried out on Merck Kieselgel 60 PF254 0.2 mm plates Ultra-violet light (254 nm) and acidic potassium permanganate ($KMnO_4$) solution was used for visualization. All reactions were carried out using oven dried glassware under a nitrogen atmosphere unless stated otherwise. ^1H NMR yields were determined with 1,1,2,2-tetrachloroethane. ^1H NMR spectra were recorded on Bruker DPX 400 MHz or 500 MHz spectrometers. ^{13}C NMR spectra were recorded at 101 MHz and 125 MHz on the same instruments. Spectra were recorded at 298 K. 2D experiments (COSY, HSQC, HMBC) were used to assign spectra but are not included. Chemical shifts (δ) are quoted in parts per million (ppm) relative to residual solvent ($CDCl_3$: δ = 7.26 ppm for ^1H NMR spectra and δ = 77.16 ppm for ^{13}C NMR spectra). Coupling constants are quoted to the nearest 0.1 Hz. Signal multiplicity is described using the following abbreviations: s = singlet, d = doublet, t = triplet, q = quartet, m = multiplet, br = broad, app. = apparent. High resolution mass spectra (HRMS) was measured at the EPSRC Mass Spectrometry Service at the University of Swansea. Infrared (IR) spectra were recorded on the Perkin Elmer FT-IR spectrometer. Melting points (m.p.) were recorded using a Gallenkamp melting point apparatus and are uncorrected. 2,2-Diethyl-4-oxa-1-azaspiro[5.5]undecan-3-one was prepared by Chuan He.[1]

General Procedure 1: Bargellini Reactioon

Amino alcohol (1 equiv), ketone (10 equiv), $CHCl_3$ (1.5 equiv) were added to a flask, cooled to 0 °C and powdered NaOH (5.2 equiv) was added slowly. The ice bath was removed after 1 h and the reaction mixture stirred at rt for 16 h. The reaction was filtered and the white gum washed generously with MeOH. The filtrate was concentrated *in vacuo*, conc. HCl was added until acidic and then refluxed at 130 °C for 2 days. The reaction was cooled and neutralized by slow addition of $NaHCO_3$. The neutralised phase was extracted with CH_2Cl_2 (3x), dried over $MgSO_4$, filtered and concentrated *in vacuo*. The crude material was then purified as specified.

General Procedure 2: Alkylation of Morpholinones

Morpholinone (1 equiv) was dissolved in THF/DME (1:1, 2 mL/mmol) and cooled to -78 °C. NaHMDS (1 equiv) was added dropwise and the mixture stirred for 10 minutes. Alkyl iodide (2–3 equiv) was added to the reaction mixture which was stirred o/n, while warming to rt. The reaction was quenched by the addition of sat. aq. NH_4Cl and extracted with diethyl ether (3x), dried over $MgSO_4$ and concentrated *in vacuo*. The crude material was purified by flash chromatography as specified.

General Procedure 3: Hydrogenation of Morpholinones

The benzyl-protected morpholinone (1 equiv) was dissolved in MeOH (0.1 mL/mg) and Pd/C (10% Pd, 20 mmol) added. The reaction mixture was evacuated and backfilled with N_2 (3x), before being backfilled with H_2. The reaction mixture was stirred under a H_2 atmosphere o/n. Once completed the reaction was filtered through celite and concentrated *in vacuo*.

General Procedure 4: Ozonolysis and Olefination of Allyl Morpholinones

tert-Butyl 3-allyl-3-ethyl-5,5-dimethyl-2-oxomorpholine-4-carboxylate (1 equiv) was dissolved in CH_2Cl_2 (10 mL/mmol), cooled to -78 °C and ozone bubbled through the reaction mixture for 0.5 h. Triphenylphosphine/dimethylsulfide was added to the reaction mixture which was allowed to warm to rt, stirring for 1h and then concentrated *in vacuo*. In a separate oven-dried flask, the Horner-Wadworth-Emmons reagent (3-5 equiv) was dissolved in THF (10 mL/mmol), cooled to 0 °C, NaH (3-5 equiv) added and stirred for 0.5 h. The crude ozonolysis product was added to the ylide in THF and stirred

at rt o/n. Water (50 mL) was added to the reaction, extracted with EtOAc (3x 50 mL), dried over $MgSO_4$ and concentrated *in vacuo*.

General Procedure 5: Boc deprotection of Morpholinones

Morpholinone (1 equiv) was dissolved in CH_2Cl_2 (10 mL/mmol), trifluoroacetic acid (2 mL/mmol) added and stirred at rt o/n. The reaction mixture was quenched by the addition of sat. aq. $NaHCO_3$, extracted with CH_2Cl_2 (3x), dried over $MgSO_4$ and concentrated *in vacuo*.

3-Ethyl-5,5-dimethyl-3-propylmorpholin-2-one,[12] 1a

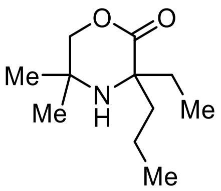

Prepared according to General Procedure **1**, using 2-amino-2-methyl-1-propanol (5.0 mL, 52.4 mmol). The crude material was purified by flash chromatography (silica gel, 13 cm, 5 cm Ø) eluting with 0-20% EtOAc/petroleum ether. This material was re-purified by kugelrohr distillation (160 °C, 20 mBar) to give the title compound as a light yellow oil (6.76 g, 33.9 mmol, 32%). **TLC** R_f = 0.26 (20% EtOAc/petroleum ether). **IR** V_{max}/cm^{-1} ($CHCl_3$) 3676, 3347, 2965, 2875, 1728, 1463, 1379, 1284, 1130, 1055. **^{1}H NMR** (400 MHz, Chloroform-*d*) δ 4.10 (s, 2H), 1.82 – 1.50 (m, 4H), 1.44 – 1.30 (m, 2H), 1.18 (s, 6H), 0.92 (app. td, *J* = 7.3, 3.0 Hz, 6H). **^{13}C NMR** (101 MHz, Chloroform-*d*) δ 174.3, 77.5, 61.2, 48.6, 42.7, 33.5, 26.9, 26.8, 17.2, 14.5, 8.3. **HRMS**-NSI: $[C_{11}H_{21}NO_2+H]^+$ requires: 200.1645, found: 200.1642.

3,3-Diethyl-5,5-dimethylmorpholin-2-one,[12] 1b

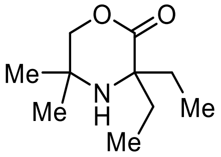

Prepared according to General Procedure **1**, using 2-amino-2-methyl-1-propanol (1.1 mL, 11.2 mmol). The crude material was purified by flash chromatography (silica gel, 15 cm, 3 cm Ø) eluting with 0-30% EtOAc/petroleum ether to give the title compound as a light yellow oil (1.29 g, 6.95 mmol, 62%). **TLC** R_f = 0.20 (20% EtOAc/petroleum ether). **IR** V_{max}/cm^{-1} ($CHCl_3$) 2970, 2950, 2880, 1709, 1459, 1379, 1285, 1222, 1183, 1131, 1104, 1050. **^{1}H NMR** (400 MHz, Chloroform-*d*) δ 4.08 (s, 2H), 1.72 (dq, *J* = 14.0, 7.4 Hz, 2H), 1.61 (dq, *J* = 14.0, 7.4 Hz, 2H), 1.16 (s, 6H), 0.90 (t, *J* = 7.4 Hz, 6H). **^{13}C NMR** (101 MHz, Chloroform-*d*) δ 174.2, 77.5, 61.4, 48.6, 32.9, 26.8, 8.3. **HRMS**-NSI: $[C_{10}H_{19}NO_2+H]^+$ requires: 186.1489, found: 186.1485.

3-Ethyl-5,5-dimethyl-3-phenethylmorpholin-2-one 1c:

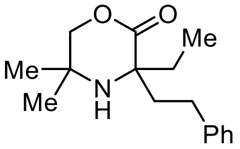

A round bottom flask was charged with 2-amino-2-methyl-1-propanol (99 µL, 1.6 mmol), $CHCl_3$ (200 uL, 2.49 mmol), 1-phenylpentan-3-one (1.6 g, 10 mmol), The flask was equipped with a stir bar, and cooled to 0 ºC using an ice bath. Powdered NaOH (328 mg, 8.2 mmol) was then added portion-wise at a rate that maintained the internal temperature below 5 ºC. After the addition was complete the reaction mixture was allowed to gradually warm to room temperature overnight. The resulting slurry was filtered on a frit, the white solid was washed with CH_2Cl_2 (30 mL) followed by methanol (100 mL) and the combined filtrates were concentrated *in vacuo* to provide the crude carboxylate. The amorphous solid was treated with a concentrated solution of

hydrochloric acid (2 mL) and heated to reflux for 6 hours. The reaction mixture was cooled to room temperature and concentrated *in vacuo*. The residue was cooled to 0 ºC and basified by the addition of a saturated aqueous solution of $NaHCO_3$. The aqueous solution was extracted with ethyl acetate (3 x 30 mL) and the combined organic extracts were dried ($MgSO_4$), filtered and concentrated *in vacuo*. The crude material was purified by flash chromatography (20% EtOAc in 40-60 °C Petroleum ether) to provide the title compound as a colourless oil (147 mg, 35% yield) **IR** V_{max}/cm^{-1} (neat film) 2957, 2882, 1732, 1448, 1372, 1103; **^{1}H NMR** (400 MHz, Chloroform-*d*) δ: 7.34–7.15 (5H, m), 4.31–4.00 (2H, m), 2.75–2.63 (2H, m), 2.06–1.95 (1H, m), 1.95–1.78 (2H, m), 1.74–1.69 (1H, m), 1.21 (6H, s), 0.97 (3H, t, *J* = 7.4 Hz); **^{13}C NMR** (101 MHz, Chloroform-*d*) δ: 173.9, 142.0, 128.6, 128.6, 126.1, 77.7, 61.2, 48.7, 42.4, 33.4, 30.4, 26.9, 26.9, 8.4.; **HRMS**-NSI: $[C_{16}H_{23}O_2N+H]^+$ requires: 262.1802, found: 262.1804.

4-Benzyl-5,5-dimethylmorpholin-2-one

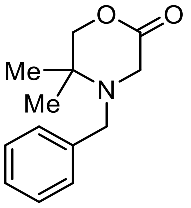

A reaction mixture of 2-amino-2-methylpropan-1-ol (5.0 mL, 50 mmol) and benzaldehyde (6.9 mL, 68 mmol) in toluene (100 mL) was refluxed under Dean-Stark conditions for 1.5 h. The reaction mixture was then concentrated in vacuo, dissolved in EtOH (150 mL) and sodium borohydride (6.6 g, 175 mmol) added portion-wise at 0 °C and left to stir at rt o/n. The reaction was quenched with 3 M HCl, concentrated *in vacuo* and extracted with CH_2Cl_2 (2x 100 mL). The aqueous layer was then basified with solid NaOH, extracted with CH_2Cl_2 (2x 100 mL), dried over $MgSO_4$, filtered and concentrated *in vacuo*. The crude amine was dissolved in toluene (100 mL), *N,N*-diisopropylethylamine (12 mL, 70 mmol) and 2-bromomethyl acetate (5.2 mL, 55 mmol) added to the reaction mixture which was stirred at 50 °C o/n. The reaction mixture was diluted with water (100 mL), extracted with CH_2Cl_2 (3x, 100 mL), dried over $MgSO_4$, and concentrated *in vacuo*. The crude material was purified by flash chromatography (silica gel, 14 cm, 5.5 cm Ø) eluting with 0-20% EtOAc/petroleum ether to yield the title compound as a white crystalline solid (5.8 g, 27 mmol, 53%). **TLC** R_f = 0.27 (20% EtOAc/petroleum ether). **IR** V_{max}/cm^{-1} ($CHCl_3$) 2980, 2954, 2933, 1744, 1382, 1292, 1231, 1054, 863. **^{1}H NMR** (500 MHz, Chloroform-*d*) δ 7.35 – 7.27 (m, 5H), 4.11 (s, 2H), 3.57 (s, 2H), 3.26 (s, 2H), 1.22 (s, 6H). **^{13}C NMR** (126 MHz, Chloroform-*d*) δ 168.6, 137.8, 128.7, 127.6, 78.7, 53.7, 51.5, 50.5, 19.2. **IR** V_{max}/cm^{-1} ($CHCl_3$) 2980, 2954, 2933, 1744, 1382, 1292, 1231, 1054, 863. **HRMS**-NSI: $[C_{13}H_{17}NO_2+H]^+$ requires: 220.1332, found: 220.1332. **Mp** = 69-72 °C.

4-Benzyl-3-ethyl-5,5-dimethylmorpholin-2-one

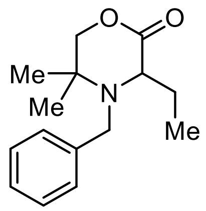

Prepared according to General Procedure **2** using 4-benzyl-5,5-dimethylmorpholin-2-one (3.85 g, 17.6 mmol). The crude material was purified by flash chromatography (silica gel, 18 cm, 3 cm Ø) eluting with 0-10% EtOAc/petroleum ether to yield the title compound as a white solid (3.5 g, 14 mmol, 81%). **TLC** R_f = 0.43 (20% EtOAc/petroleum ether). **IR** V_{max}/cm^{-1} ($CHCl_3$) 2966, 2935, 2901, 1726, 1452, 1387, 1222, 1063, 874, 718, 696. **^{1}H NMR** (500 MHz, Chloroform-*d*) δ 7.37 – 7.27 (m, 4H), 7.25 – 7.21 (m, 1H), 4.25 (d, *J* = 10.8 Hz, 1H), 4.10 (d, *J* = 15.4 Hz, 1H), 3.93 (d, *J* = 10.8 Hz, 1H), 3.45 (dd, *J* = 6.0, 3.6 Hz, 1H), 3.29 (d, *J* = 15.4 Hz, 1H), 1.69 (dqd, *J* = 14.5, 7.3, 3.6 Hz, 1H), 1.38 (dqd, *J* = 14.5, 7.3, 6.0 Hz, 1H), 1.20 (s, 3H), 1.11 (s, 3H), 0.83 (t, *J* = 7.4 Hz, 3H). **^{13}C NMR** (126 MHz, Chloroform-*d*) δ 172.2, 141.3, 128.4, 127.7, 127.1, 77.3, 65.2, 55.1, 53.0, 27.2, 25.0, 16.6, 9.2. **HRMS**-NSI: $[C_{15}H_{21}NO_2+H]^+$ requires: 248.1645, found: 248.1646. **Mp =** 78-80 °C.

3-Allyl-4-benzyl-3-ethyl-5,5-dimethylmorpholin-2-one

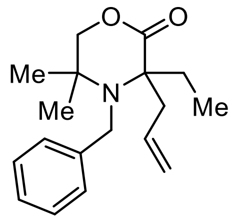

Prepared according to General Procedure **2** using 4-benzyl-3-ethyl-5,5-dimethylmorpholin-2-one (3.52 g, 14.2 mmol). The crude material was purified by flash chromatography (silica gel, 20 cm, 5 cm Ø) eluting with 0-10% EtOAc/petroleum ether to yield the title compound as a light yellow oil (1.2 g, 4.3 mmol, 30%). **TLC** R_f = 0.46 (20% EtOAc/petroleum ether). **IR** V_{max}/cm^{-1} ($CHCl_3$) 2975, 1731, 145, 1382, 1285, 1176, 1137, 1066. **^{1}H NMR** (400 MHz, Chloroform-*d*) δ 7.41 – 7.34 (m, 2H), 7.35 – 7.26 (m, 2H), 7.27 – 7.18 (m, 1H), 5.90 (ddt, *J* = 16.6, 10.6, 7.3 Hz, 1H), 5.16 – 5.05 (m, 2H), 4.10 (s, 2H), 4.06 (d, *J* = 16.6 Hz, 1H), 3.92 (d, *J* = 16.6 Hz, 1H), 2.53 (ddt, *J* = 14.3, 7.3, 1.3 Hz, 1H), 2.42 (ddt, *J* = 14.3, 7.3, 1.2 Hz, 1H), 1.85 (dq, *J* = 14.6, 7.4 Hz, 1H), 1.60 (dq, *J* = 16.4, 7.4 Hz, 1H), 1.20 (s, 3H), 1.13 (s, 3H), 1.00 (t, *J* = 7.4 Hz, 3H). **^{13}C NMR** (101 MHz, Chloroform-*d*) δ 173.2, 143.1, 135.0, 128.4, 127.5, 126.9, 118.5, 76.6, 69.5, 52.3, 48.0, 44.7, 32.8, 24.7, 23.7, 10.4. **HRMS**-NSI: $[C_{18}H_{25}NO_2+H]^+$ requires: 288.1958, found: 288.1961.

(*E*)-Ethyl 4-(4-benzyl-3-ethyl-5,5-dimethyl-2-oxomorpholin-3-yl)but-2-enoate

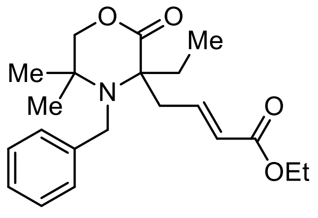

A 50 mL round bottom flask equipped with stir bar and condenser was charged with Grubbs' second generation catalyst (13.3 mg, 0.016 mmol) and 3-allyl-4-benzyl-3-ethyl-5,5-dimethylmorpholin-2-one (287 mg, 1.0 mmol). To this was added dichloromethane (10 mL) and ethyl acrylate (0.917 mL, 10.1 mmol). The mixture was quickly subjected to three cycles of vacuum / nitrogen backfill and then and heated at 60 ºC under a nitrogen atmosphere for 24 hours. The reaction mixture was cooled to room temperature, filtered through celite, eluting with ethyl acetate, and concentrated *in vacuo*. The crude material was purified by flash column chromatography (20% ethyl acetate in petroleum ether 40-60 ºC) to provide (*E*)-ethyl 4-(4-benzyl-3-ethyl-5,5-dimethyl-2-oxomorpholin-3-yl)but-2-enoate as a colourless oil (79 mg, 74% yield). **TLC** R*f* = 0.29 (20% ethyl acetate in petroleum ether 40-60 ºC); **IR** V_{max}/cm^{-1} (neat film) 2977, 2255, 1714, 1651, 1451, 1383, 1368, 1270, 1173, 1136; **^{1}H NMR** (400 MHz, Chloroform-*d*) δ: 7.37–7.22 (5H, m), 7.02 (1H, pent, *J* = 7.8 Hz), 5.84 (1H, d, *J* = 15.6 Hz), 4.19 (2H, q, *J* = 7.1 Hz), 4.15–4.09 (1H, m), 4.05–3.91 (3H, m), 2.68 (1H, ddd, *J* = 14.4, 8.2, 1.1 Hz), 2.52 (1H, ddd, *J* = 14.4, 7.3, 1.1 Hz), 1.83 (1H, dq, *J* = 14.5, 7.3 Hz), 1.64 (1H, dq, *J* = 14.5, 7.3 Hz), 1.29 (3H, t, *J* = 7.1 Hz), 1.21 (3H, s), 1.13 (3H, s), 1.00 (3H, t, *J* = 7.3 Hz); **^{13}C NMR** (101 MHz, Chloroform-*d*) δ: 172.7, 166.3, 145.0, 142.5, 128.6, 127.5, 127.1, 124.4, 76.6, 69.5, 60.5, 52.5, 48.1, 42.5, 33.3, 24.9, 23.3, 14.4, 10.3; **HRMS**-NSI: $[C_{21}H_{29}NO_4+H]^+$ requires: 360.2169, found: 360.2172.

Ethyl 4-(3-ethyl-5,5-dimethyl-2-oxomorpholin-3-yl)butanoate 1d:

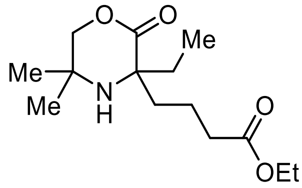

A solution of (*E*)-ethyl 4-(4-benzyl-3-ethyl-5,5-dimethyl-2-oxomorpholin-3-yl)but-2-enoate (296 mg, 0.84 mmol) in methyl acetate (11.5 mL) was subjected to three cycles of vacuum / nitrogen backfill. Palladium on activated carbon (Pd/C, 10 wt. %, 162 mg) was added in one portion, the atmosphere was exchanged for hydrogen and the reaction stirred at room temperature for 16 hours. The reaction was filtered through celite and concentrated *in vacuo* to provide the title compound as a colourless oil (230 mg, 99% yield). **IR** V_{max}/cm^{-1} (neat film) 2968, 1725, 1460, 1377, 1284, 1177, 1114, 1053; **^{1}H NMR** (400 MHz, Chloroform-*d*) δ: 4.25–4.05 (4H, m), 2.38–2.23 (2H, m), 1.83–1.56 (6H, m), 1.25 (3H, t, *J* = 7.1 Hz), 1.19 (3H, s), 1.18 (3H, s), 0.93 (3H, t, *J* = 7.4 Hz); **^{13}C NMR** (101 MHz, Chloroform-*d*) δ: 174.0, 173.5, 77.7, 61.0, 60.5, 48.7, 39.4, 34.3, 33.3, 26.9, 26.7, 19.3, 14.4, 8.4; **HRMS**-NSI: $[C_{14}H_{25}NO_4+H]^+$ requires: 272.1856, found: 272.1856.

***tert*-Butyl 5,5-dimethyl-2-oxomorpholine-4-carboxylate**

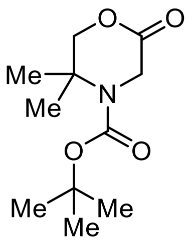

2-Amino-2-methylpropan-1-ol (4.8 mL, 50 mmol) was dissolved in THF (100 mL), triethylamine (12 mL, 85 mmol) and ethylbromo acetate (7.1 mL, 75 mmol) were added to the reaction and stirred at 50 °C for 4 h. The reaction mixture was cooled to 0 °C, filtered and washed with THF (100 mL). The filtrate was concentrated to approx. 100 mL, di-*tert*-butyl decarbonate (15 g, 70 mmol) was added to the reaction and stirred at rt o/n. The reaction was concentrated *in vacuo*, re-dissolved in toluene (150 mL) and washed with sat. aq. $NaHCO_3$ (100 mL), brine (100 mL), dried over $MgSO_4$ and concentrated to approx. 100 mL. *p*-Toluenesulfonic acid (0.95 g, 5.0 mmol) was added and the reaction mixture refluxed under Dean-Stark conditions for 4 h. The reaction was cooled to rt, washed with water (100 mL), dried over $MgSO_4$ and concentrated *in vacuo*. The crude material was purified by flash chromatography (silica gel, 10 cm, 7 cm Ø) eluting with 0-10% EtOAc/petroleum ether to yield the title compound as a white solid (3.7 g, 16 mmol, 33%). **TLC** R_f = 0.22 (20% EtOAc/petroleum ether). **IR** V_{max}/cm^{-1} ($CHCl_3$) 2973, 1760, 1683, 1362, 1302, 1255, 1148, 100, 1051. **^{1}H NMR** (400 MHz, Chloroform-*d*) δ 4.20 (s, 2H), 4.06 (s, 2H), 1.48 (s, 9H), 1.46 (s, 6H). **^{13}C NMR** (126 MHz, Chloroform-*d*) δ 169.2, 153.6, 81.3, 75.5, 53.7, 44.6, 28.6, 23.0. **HRMS**-NSI: $[C_{11}H_{19}NO_4-{}^tButyl+H]^+$ requires: 173.0688, found: 173.0683. **Mp** = 98-100 °C.

***tert*-Butyl 3-ethyl-5,5-dimethyl-2-oxomorpholine-4-carboxylate**

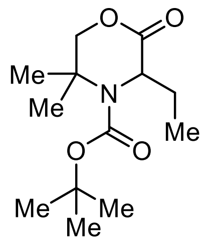

Title compound isolated as residual starting material (from the synthesis of *tert*-butyl 3-allyl-3-ethyl-5,5-dimethyl-2-oxomorpholine-4-carboxylate) by flash chromatography (silica gel, 13 cm, 7 cm Ø) eluting with 5% EtOAc/petroleum ether to yield the title compound as a light yellow solid (2.1 g, 8.2 mmol, 50%). **TLC** R_f = 0.16 (10% EtOAc/petroleum ether). **IR** V_{max}/cm^{-1} ($CHCl_3$) 2973, 2934, 1753, 1688, 1367, 1291, 1170, 1062, 731. **^{1}H NMR** (400 MHz, Chloroform-*d*) δ 4.50 (dd, *J* = 9.3, 6.6 Hz, 1H), 4.31 (d, *J* = 12.1 Hz, 1H), 3.90 (d, *J* = 12.1 Hz, 1H), 1.80 (m, 2H), 1.48 (s, 9H), 1.47 (s, 3H), 1.45 (s, 3H), 1.04 (t, *J* = 7.5 Hz, 3H). **^{13}C NMR** (101 MHz, Chloroform-*d*) δ 170.0, 81.1, 74.5, 57.9, 53.3, 28.6, 28.2, 24.6, 22.9, 10.8. **Mp** = 66-68 °C.

tert-Butyl 3-allyl-3-ethyl-5,5-dimethyl-2-oxomorpholine-4-carboxylate

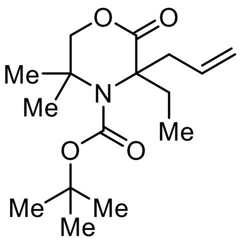

Prepared by sequential alkylation according to General Procedure **2** from _tert_-butyl 5,5-dimethyl-2-oxomorpholine-4-carboxylate (3.7 g, 16.3 mmol) using ethyl iodide (2 equiv) to give crude _tert_-butyl 3-ethyl-5,5-dimethyl-2-oxomorpholine-4-carboxylate which was alkylated with allyl iodide (3 equiv) using general procedure **2**. The crude material was purified by flash chromatography (silica gel, 13 cm, 7 cm Ø) eluting with 5% EtOAc/petroleum ether to yield the title compound as a light yellow oil (1.0 g, 3.4 mmol, 21%). **TLC** R_f = 0.21 (10% EtOAc/petroleum ether). **IR** V_{max}/cm^{-1} ($CHCl_3$) 2973, 2934, 1744, 1693, 1367, 1344, 1298, 1157, 1074. **^{1}H NMR** (500 MHz, Chloroform-_d_) δ 5.70 (ddt, _J_ = 17.6, 10.1, 7.6 Hz, 1H), 5.19 – 5.08 (m, 2H), 4.12 (d, _J_ = 11.7 Hz, 1H), 3.95 (d, _J_ = 11.7 Hz, 1H), 3.10 (dd, _J_ = 13.9, 7.8 Hz, 1H), 2.83 (dd, _J_ = 13.9, 7.3 Hz, 1H), 2.34 (dq, _J_ = 14.8, 7.5 Hz, 1H), 2.27 (dq, _J_ = 14.8, 7.5 Hz, 1H), 1.50 (s, 9H), 1.41 (s, 3H), 1.39 (s, 3H), 0.84 (t, _J_ = 7.5 Hz, 3H). **^{13}C NMR** (126 MHz, Chloroform-_d_) δ 171.2, 154.0, 133.6, 119.5, 80.9, 74.3, 68.8, 52.7, 42.5, 30.9, 28.6, 24.7, 9.9. **HRMS**-NSI: $[C_{16}H_{27}NO_4+H]^+$ requires: 298.2013, found: 298.2009.

tert-Butyl 3-(3-cyanoallyl)-3-ethyl-5,5-dimethyl-2-oxomorpholine-4-carboxylate

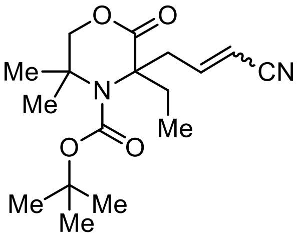

Prepared according to General Procedure **4**. The ozonolysis of _tert_-butyl 3-allyl-3-ethyl-5,5-dimethyl-2-oxomorpholine-4-carboxylate (0.45 g, 1.5 mmol) was quenched by the addition of dimethylsulfide (2 equiv). The olefination step used diethyl (cyanomethyl)phosphonate (3 equiv) and NaH (3 equiv). The crude material was purified by flash chromatography (silica gel, 16 cm, 3 cm Ø) eluting with 10-30% EtOAc/petroleum ether to yield the title compound as an inseparable, isomeric mixture of E/Z-isomers (1:1.5), as a colourless oil (0.36 g, 1.1 mmol, 74%). **TLC** R_f = 0.19 (20% EtOAc/petroleum ether). **IR** V_{max}/cm^{-1} ($CHCl_3$) 2972, 1743, 1690, 1337, 1295, 1246, 1163, 1137, 1071. **^{1}H NMR** (500 MHz, Chloroform-_d_) δ 6.50 (ddd, J = 15.8, 8.6, 6.9 Hz, 0.4H, H-9_E), 6.37 (ddd, J = 10.9, 8.6, 6.5 Hz, 0.6H, H-9_Z), 5.47 – 5.37 (m, 1H, H-10), 4.19 (d, J = 11.9 Hz, 0.4H, H-3_E), 4.17 (d, J = 11.9 Hz, 0.6H, H-3_Z), 4.07 (d, J = 11.9 Hz, 0.6H H-3_Z), 3.93 (d, J = 11.9 Hz, 0.4H, H-3_E), 3.42 (ddd, J = 15.1, 6.5, 1.7 Hz, 0.6H, H-8_Z), 3.38 – 3.27 (m, 1H, H-8), 3.13 (ddd, J = 14.0, 7.0, 1.6 Hz, 0.4H, H-8_E), 2.42 – 2.31 (m, 1.6H, H-6), 2.20 (dq, J = 14.8, 7.5 Hz, 0.4H, H-6_E), 1.51 (app. d, _J_ = 4.1 Hz, 9H, H-14), 1.43 (app. d, _J_ = 2.8 Hz, 4.8H, H-1), 1.37 (s, 1.2H, H-1), 0.89 (t, J = 7.5 Hz, 1.2H, H-7_E), 0.85 (t, J = 7.4 Hz, 1.8H, H-7_Z). **^{13}C NMR** (126 MHz, Chloroform-_d_) δ 169.8 (C-4_E), 169.6 (C-4_Z), 153.9 (C-12_E), 153.8 (C-12_Z), 150.7 (C-9_E), 149.3 (C-9_Z), 117.1 (C-11_E), 115.7 (C-11_Z), 103.8 (C-

10$_E$), 102.5 (C-10$_Z$), 81.7 (C-13), 81.6 (C-13), 74.4 (C-3$_E$), 74.3 (C-3$_Z$), 67.7 (C-5$_E$), 67.3 (C-5$_Z$), 53.5 (C-2$_Z$), 53.3 (C-2$_E$), 40.0 (C-8$_Z$), 39.6 (C-8$_E$), 31.4 (C-6$_E$), 29.8 (C-6$_Z$), 28.5 (C-14), 25.5 (C-1), 25.0 (C-1), 24.1 (C-1), 9.7 (C-7$_E$), 9.6 (C-7$_Z$). **HRMS**-NSI: $[C_{17}H_{26}N_2O_4+H]^+$ requires: 323.1965, found: 323.1966.

4-(3-Ethyl-5,5-dimethyl-2-oxomorpholin-3-yl)butanenitrile 1f

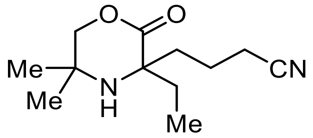

Prepared according to General Procedure **3** from *tert*-butyl 3-(3-cyanoallyl)-3-ethyl-5,5-dimethyl-2-oxomorpholine-4-carboxylate (0.35 g, 1.1 mmol) and subsequent deprotection as described by General Procedure **5**, stirring for 3 d. The crude material was purified by flash chromatography (silica gel, 12 cm, 2 cm Ø) eluting with 0-20% EtOAc/petroleum ether to yield the title compound as a colourless oil (64 mg, 0.29 mmol, 26%). **TLC** R_f = 0.40 (20% EtOAc/CH_2Cl_2). **IR** V_{max}/cm^{-1} ($CHCl_3$) 2968, 1726, 1458, 1379, 1284, 1219, 1155, 1117. **^{1}H NMR** (400 MHz, Chloroform-*d*) δ 4.12 (s, 2H), 2.37 (td, *J* = 6.8, 2.1 Hz, 2H), 1.88 – 1.61 (m, 6H), 1.20 (s, 3H), 1.19 (s, 3H), 0.95 (t, *J* = 7.4 Hz, 3H). **^{13}C NMR** (101 MHz, Chloroform-*d*) δ 173.4, 119.6, 77.9, 60.8, 48.7, 39.2, 33.5, 26.9, 26.6, 20.3, 17.6, 8.4. **HRMS**-NSI: $[C_{12}H_{20}N_2O_2+H]^+$ requires: 225.1598, found: 225.1600.

***tert*-Butyl 3-ethyl-5,5-dimethyl-2-oxo-3-(3-(phenylsulfonyl)propyl)morpholine-4-carboxylate**

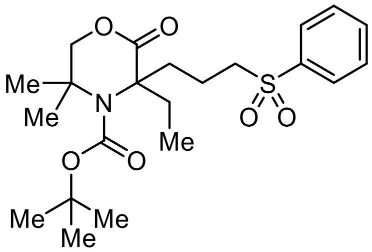

Prepared according to General Procedure **4**. The ozonolysis of *tert*-butyl 3-allyl-3-ethyl-5,5-dimethyl-2-oxomorpholine-4-carboxylate (0.30 g, 1.0 mmol) was quenched by the addition of triphenylphosphine (1.05 equiv). The olefination step used diethyl ((phenylsulfonyl)methyl)phosphonate (4.8 equiv) and NaH (4.8 equiv). The crude material was subjected to hydrogenation as described in General Procedure **3**. The crude material was purified by flash chromatography (silica gel, 14 cm, 2 cm Ø) eluting with 20-50% EtOAc/petroleum ether to yield the title compound as a colourless oil (0.15 g, 0.34 mmol, 34%). **TLC** R_f = 0.38 (50% EtOAc/petroleum ether). **IR** V_{max}/cm^{-1} ($CHCl_3$) 2972, 1740, 1689, 1447, 1367, 1323, 1294, 1146, 1070. **^{1}H NMR** (500 MHz, Chloroform-*d*) δ 7.90 – 7.85 (m, 2H), 7.68 – 7.62 (m, 1H), 7.59 – 7.52 (m, 2H), 4.11 (d, *J* = 11.8 Hz, 1H), 3.96 (d, *J* = 11.8 Hz, 1H), 3.11 (ddd, *J* = 14.0, 9.9, 6.3 Hz, 1H), 2.98 (ddd, *J* = 14.0, 9.8, 6.0 Hz, 1H), 2.40 – 2.12 (m, 4H), 1.64 – 1.50 (m, 2H), 1.45 (s, 9H), 1.41 (s, 3H), 1.36 (s, 3H), 0.82 (t, *J* = 7.5 Hz, 3H). **^{13}C NMR** (126 MHz, Chloroform-*d*) δ 170.3, 153.9, 139.1, 133.9, 129.4, 128.3, 81.3, 74.4, 67.9, 56.3, 53.0, 34.9, 30.9, 28.5, 25.0, 24.2, 19.2, 9.7. **HRMS**-NSI: $[C_{22}H_{33}NO_6S+NH_4]^+$ requires: 457.2367, found: 457.2365.

3-Ethyl-5,5-dimethyl-3-(3-(phenylsulfonyl)propyl)morpholin-2-one 1g

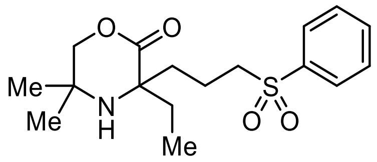

Prepared according to General Procedure **5** from tert-butyl 3-ethyl-5,5-dimethyl-2-oxo-3-(3-(phenylsulfonyl)propyl)morpholine-4-carboxylate (0.15 g, 0.34 mmol) to give the title compound as a colourless oil (114 mg, 0.34 mmol, quant.). **IR** V_{max}/cm^{-1} ($CHCl_3$) 2968, 1726, 1446, 1303, 1286, 1146, 1086. **^{1}H NMR** (400 MHz, Chloroform-*d*) δ 7.94 – 7.88 (m, 2H), 7.70 – 7.63 (m, 1H), 7.61 – 7.53 (m, 2H), 4.08 (s, 2H), 3.20 – 3.01 (m, 2H), 1.90 – 1.57 (m, 6H), 1.16 (s, 3H), 1.14 (s, 3H), 0.91 (t, *J* = 7.4 Hz, 3H). **^{13}C NMR** (126 MHz, Chloroform-*d*) δ 173.4, 139.3, 133.9, 129.5, 128.2, 77.9, 60.9, 56.5, 48.7, 38.5, 33.5, 26.8, 26.5, 17.6, 8.4. **HRMS**-NSI: $[C_{17}H_{25}NO_4S+H]^+$ requires: 340.1577, found: 340.1575.

2-(3-(4-Benzyl-5,5-dimethyl-2-oxomorpholin-3-yl)propyl)isoindoline-1,3-dione

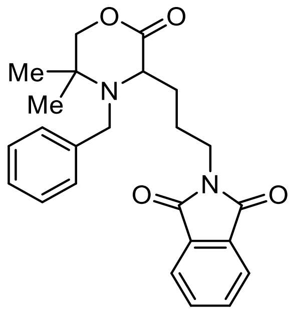

Prepared according to General Procedure **2** using 4-benzyl-5,5-dimethylmorpholin-2-one (0.33 g, 1.5 mmol) and 2-(3-iodopropyl)isoindoline-1,3-dione (2 equiv) prepared by a literature procedure.[1] The crude material was purified by flash chromatography (silica gel, 12 cm, 2 cm Ø) eluting with 0-30% EtOAc/petroleum ether to give the title compound as a yellow oil (0.43 g, 1.0 mmol, 70%). **TLC** R_f = 0.10 (20% EtOAc/petroleum ether). **IR** V_{max}/cm^{-1} ($CHCl_3$) 1771, 1735, 1708, 1467, 1396, 1066, 1037, 719. **^{1}H NMR** (500 MHz, Chloroform-*d*) δ 7.80 (dd, *J* = 5.4, 3.0 Hz, 2H), 7.71 (dd, *J* = 5.4, 3.0 Hz, 2H), 7.26 – 7.23 (m, 2H), 7.15 – 7.09 (m, 2H), 6.91 – 6.84 (m, 1H), 4.23 (d, *J* = 10.8 Hz, 1H), 4.10 (d, *J* = 15.2 Hz, 1H), 3.93 (d, *J* = 10.8 Hz, 1H), 3.47 (dd, *J* = 6.1, 3.5 Hz, 1H), 3.44 (dt, *J* = 13.6, 6.8 Hz, 1H), 3.38 (dt, *J* = 13.6, 6.8 Hz, 1H), 3.19 (d, *J* = 15.2 Hz, 1H), 1.86 (ttd, *J* = 12.7, 6.7, 4.4 Hz, 1H), 1.65 (dtd, *J* = 12.1, 4.4, 3.5 Hz, 1H), 1.44 (ttd, *J* = 12.7, 6.7, 4.4 Hz, 1H), 1.31 (ddt, *J* = 12.1, 6.1, 4.4 Hz, 1H), 1.17 (s, 3H), 1.15 (s, 3H). **^{13}C NMR** (126 MHz, Chloroform-*d*) δ 171.7, 168.3, 140.8, 133.9, 132.3, 128.4, 127.6, 127.0, 123.3, 77.2, 64.1, 55.4, 53.0, 37.7, 31.7, 24.9, 24.2, 16.5. **HRMS**-NSI: $[C_{24}H_{26}N_2O_4+H]^+$ requires: 407.1965, found: 407.1964.

2-(3-(4-Benzyl-3-ethyl-5,5-dimethyl-2-oxomorpholin-3-yl)propyl)isoindoline-1,3-dione

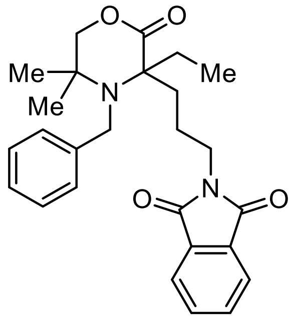

Prepared according to General Procedure **2** from 2-(3-(4-benzyl-5,5-dimethyl-2-oxomorpholin-3-yl)propyl)isoindoline-1,3-dione (0.50 g, 2.0 mmol) and ethyl iodide (2.5 equiv). The crude material was purified by flash chromatography (silica gel, 13 cm, 3 cm Ø) eluting with 0-20% EtOAc/petroleum ether to give the title compound as a yellow oil (0.17 g, 0.38 mmol, 19%). **TLC** R_f = 0.11 (20% EtOAc/petroleum ether). **IR** V_{max}/cm^{-1} ($CHCl_3$) 2971, 2901, 1707, 1730, 1396, 1065, 908, 714. **^{1}H NMR** (400 MHz, Chloroform-*d*) δ 7.84 (dd, *J* = 5.4, 3.0 Hz, 2H), 7.72 (dd, *J* = 5.4, 3.0 Hz, 2H), 7.33 – 7.25 (m, 2H), 7.26 – 7.17 (m, 2H), 7.10 – 7.01 (m, 1H), 4.11 (d, *J* = 10.9 Hz, 1H), 4.05 (d, *J* = 10.9 Hz, 1H), 3.92 (d, *J* = 16.5 Hz, 1H), 3.86 (d, *J* = 16.5 Hz, 1H), 3.60 (ddd, *J* = 13.6, 7.7, 6.0 Hz, 1H), 3.50 (dt, *J* = 13.7, 7.0 Hz, 1H), 2.06 – 1.90 (m, 1H), 1.86 (dq, *J* = 14.8, 7.4 Hz, 1H), 1.75 (td, *J* = 13.3, 3.9 Hz, 1H), 1.71 – 1.59 (m, 2H), 1.40 (td, *J* = 13.3, 3.9 Hz, 1H), 1.19 (s, 3H), 1.17 (s, 3H), 0.96 (t, *J* = 7.4 Hz, 3H). **^{13}C NMR** (126 MHz, Chloroform-*d*) δ 173.1, 168.3, 142.6, 133.9, 132.2, 128.3, 127.2, 126.6, 123.2, 76.3, 68.3, 52.1, 47.6, 37.9, 37.8, 32.4, 24.9, 23.6, 10.7. **HRMS**-NSI: $[C_{26}H_{30}N_2O_4+H]^+$ requires: 435.2278, found: 435.2276.

2-(3-(3-Ethyl-5,5-dimethyl-2-oxomorpholin-3-yl)propyl)isoindoline-1,3-dione 1h

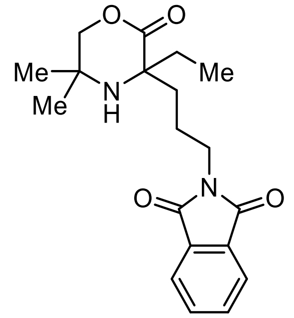

Prepared according to General Procedure **3** from 2-(3-(4-benzyl-3-ethyl-5,5-dimethyl-2-oxomorpholin-3-yl)propyl)isoindoline-1,3-dione (0.38 g, 0.87 mmol). The crude material was purified by flash chromatography (silica gel, 9 cm, 3 cm Ø) eluting with 50% EtOAc/petroleum ether to yield the title compound as a colourless oil (60 mg, 0.17 mmol, 20%). **TLC** R_f = 0.30 (50% EtOAc/petroleum ether). **IR** V_{max}/cm^{-1} ($CHCl_3$) 2972, 1709, 1396, 1286, 1047, 719. **^{1}H NMR** (500 MHz, Chloroform-*d*) δ 7.84 (dd, *J* = 5.5, 3.0 Hz, 2H), 7.71 (dd, *J* = 5.5, 3.0 Hz, 2H), 4.12 (d, *J* = 10.8 Hz, 1H), 4.07 (d, *J* = 10.8 Hz, 1H), 3.70 (td, *J* = 7.0, 1.9 Hz, 2H), 1.83 – 1.59 (m, 6H), 1.17 (s, 3H), 1.16 (s, 3H), 0.91 (t, *J* = 7.4 Hz, 3H). **^{13}C NMR** (126 MHz, Chloroform-*d*) δ 173.8, 168.5, 134.1, 132.3, 123.4, 77.7, 60.9, 48.7, 38.3, 37.4, 33.2, 26.9, 26.7, 23.2, 8.4. **HRMS**-NSI: $[C_{19}H_{24}N_2O_4+H]^+$ requires: 345.1809, found: 345.1813.

3-Ethyl-3,5,5-trimethylmorpholin-2-one

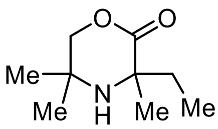

Prepared according to General Procedure **1**, using 2-amino-2-methyl-1-propanol (2.50 mL, 26.2 mmol), 2-butanone (23.0 mL, 262 mmol), chloroform (3.20 mL, 39.3 mmol) and sodium hydroxide (3.40 g, 136 mmol). The crude material was purified by flash chromatography (silica gel, 9 cm, 5.5 cm Ø) eluting with 0-50% EtOAc/petroleum ether and then purified by kugelrohr distillation (155 °C, 25 mBar) giving the title compound as a colourless oil (1.88 g, 10.98 mmol, 42%). **TLC** R_f = 0.12 (20% EtOAc/petroleum ether). **IR** V_{max}/cm^{-1} ($CHCl_3$) 2970, 1727, 1378, 1284, 1223, 1118, 1054, 915, 732. **^{1}H NMR** (400 MHz, Chloroform-*d*) δ 4.13 (d, *J* = 16.4 Hz, 1H), 4.10 (d, *J* = 16.4 Hz, 1H), 1.80 (dq, *J* = 14.7, 7.4 Hz, 1H), 1.57 (dq, *J* = 14.7, 7.4 Hz, 1H), 1.39 (s, 3H), 1.24 (s, 3H), 1.14 (s, 3H), 0.95 (t, *J* = 7.4 Hz, 3H). **^{13}C NMR** ^{13}C NMR (101 MHz, Chloroform-*d*) δ 174.7, 78.0, 58.3, 48.9, 35.8, 29.3, 27.0, 26.1, 8.6. **HRMS**-NSI: $[C_9H_{17}NO_2+H]^+$ requires: 172.1332, found: 172.1330.

3-((Benzyloxy)methyl)-3-ethyl-5,5-dimethylmorpholin-2-one 1i

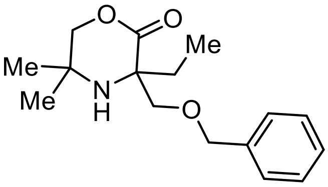

To a microwave vial containing a solution of 6-ethyl-2,2-dimethyl-4-oxa-1-azabicyclo[4.1.0]heptan-5-one (140 mg, 0.83 mmol) in BnOH (3.0 mL) at rt was added p-toluenesulfonic acid (157.0 mg, 0.83 mmol). The vial was sealed and placed in an oil bath pre-heated to 60 °C and was held for 3 h (GCMS analysis showed complete consumption of the starting material). The reaction was cooled to rt, then the solution was diluted with NaOH (6 mL, 10% aqueous). The aqueous phase was extracted with CH_2Cl_2 (3×6 mL), then the combined organic extracts were washed with brine (3 mL), dried ($MgSO_4$), then concentrated *in vacuo* to afford the crude product as a pale yellow oil. Purification by SCX followed by flash column chromatography (silica, 30% EtOAc/Petroleum ether) gave the title compound as a colourless oil (112 mg, 0.403 mmol, 49%); **IR** V_{max}/cm^{-1} (film) 2969, 1732, 1455, 1379, 1287, 1219, 1099, 1056; **^{1}H NMR** (400 MHz, Chloroform-*d*) δ: 7.37–7.20 (5H, m), 4.55 (2H, q, J = 12.5 Hz), 4.11 (2H, s), 3.70 (1H, d, J = 9.0 Hz), 3.44 (1H, d, J = 9.0 Hz), 1.79–1.66 (3H, m), 1.18 (3H, s), 1.15 (3H, s), 0.93 (3H, t, J = 7.5 Hz); **^{13}C NMR** (101 MHz, Chloroform-*d*) δ: 173.1, 137.8, 128.4, 127.8, 127.6, 77.5, 75.7, 73. 4, 61.9, 48.3, 31.9, 26.4, 26.3, 8.3; **HRMS**-NSI: $[C_{16}H_{23}NO_3+H]^+$ requires: 278.1751, found: 278.1750.

4-((2-Amino-2-methylpropyl)amino)benzonitrile

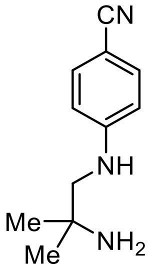

To a solution of 4-fluorocyanobenzene (242 mg, 2.00 mmol) in DMSO (1.0 mL) at rt was added 2-methylpropane-1,2-diamine (415 μL, 4.00 mmol). The reaction was then heated to 120 °C and was held for 24 h, then was cooled to rt and diluted with 10% aq. NaOH (2 mL). The aqueous phase was extracted with Et_2O (3 × 5 mL), then the combined organic extracts were washed with brine (5 mL), dried ($MgSO_4$), then concentrated *in vacuo* to afford the title compound as an off-white crystalline solid (89% 338 mg, 1.79 mmol); **TLC** R_f = 0.31 (CH_2Cl_2/MeOH/aq. NH_3, 90:9:1); **IR** V_{max}/cm^{-1} (film) 2967, 2932, 2226, 1655, 1601,1505, 1461, 1379, 1316, 1270, 1175; **^{1}H NMR** (400 MHz, Chloroform-*d*) δ: 7.36 (2H, d, J = 8.7 Hz), 6.56 (2H, d, J = 8.7 Hz), 4.91 (1H, br. s) 2.97 (2H, d, J = 5.5 Hz), 1.20 (6H, s); **^{13}C NMR** (400 MHz, Chloroform-*d*) δ: 152.1, 133.7, 120.7, 112.3, 98.0, 54.2, 50.0, 29.3; **HRMS**-NSI: $[C_{11}H_{15}N_3+H]^+$ requires: 190.1339, found: 190.1338. **mp.** 93–96 °C;

4-(3,3-diethyl-5,5-dimethyl-2-oxopiperazin-1-yl)benzonitrile 1j

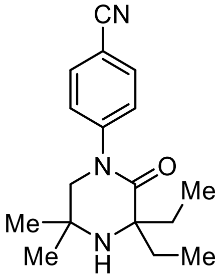

To a stirred solution of the diamine (338 mg, 1.79 mmol), chloroform (215 uL, 2.68 mmol), 3-penttanone (2.3 mL, 21.7 mmol) at 0 °C was added portion wise KOH (502 mg, 8.94 mmol) ensuring the temperature of the reaction remained below 5 °C. After the addition was complete, the reaction was allowed to warm to room temperature and was held for 18 h. The organic phase was then separated and the aqueous phase was extracted with CH_2Cl_2. The combined organic phases were washed with brine, dried ($MgSO_4$), then concentrated in vacuo to afford the crude product which was purified by flash column chromatography (Silica, 33% EtOAc in Petroleum ether 40-60 °C) to yield the title compound as a colourless oil (43%, 219 mg, 0.77 mmol); **TLC** R_f = 0.42 (33% EtOAc in Petroleum ether 40-60 °C); **IR** V_{max}/cm^{-1} (film) 2967, 2932, 2226, 1655, 1601,1505, 1461, 1379, 1316, 1270, 1175; **^{1}H NMR** (400 MHz, Chloroform-*d*) δ: 7.70–7.63 (2H, m), 7.46–7.39 (2H, m), 3.56 (2H, s), 1.79 (2H, dq, *J* = 14.9, 7.5 Hz), 1.68 (2H, dq, *J* = 14.7, 7.4 Hz), 1.28 (6H, s), 0.95 (6H, t, *J* = 7.4 Hz); **^{13}C NMR** (400 MHz, Chloroform-*d*) δ: 173.7, 147.4, 133.0, 126.0, 118.7, 109.6, 62.4, 61.4, 49.2, 33.0, 28.2, 8.4; **HRMS**-NSI: $[C_{17}H_{23}N_3O+H]^+$ requires: 286.1914, found: 286.1911.

4-Benzyl-5,5-dimethyl-3-propylmorpholin-2-one

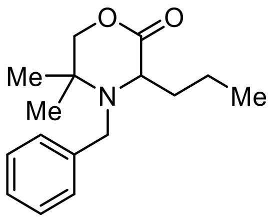

To a solution of 4-benzyl-5,5-dimethylmorpholin-2-one (323 mg, 1.47 mmol) in THF:DME (1:1, 3 mL) at −78 °C was added NaHMDS (2M in THF, 735 µL, 1.47 mmol) dropwise. After stirring at −78 °C for 0.5 h, neat *n*-propyl iodide (216 □L, 2.21 mmol) was added dropwise. The reaction was allowed to warm to r.t over 16 h before being quenched with sat. aq. NH_4Cl (5 mL). The reaction mixture was diluted with ethyl acetate (10 mL) and the organic phase separated. The aqueous phase was re-extracted with ethyl acetate (2 x 5mL) and the combined organics dried over $MgSO_4$, filtered and evaporated. The crude material was purified by silica gel column chromatography (20% EtOAc in petroleum ether) to give the title compound as a colourless oil (84%, 323 mg, 1.23 mmol); **TLC** Rf = 0.35 (20% EtOAc in petroleum ether); **IR** V_{max}/cm^{-1} (film) 2963, 2873, 1738, 1494, 1466, 1384, 1285, 1218, 1179, 1066; **^{1}H NMR** (400 MHz, Chloroform-*d*) δ: 7.48–7.17 (5H, m), 4.24 (1H, d, *J* = 10.7 Hz), 4.10 (1H, d, *J* = 15.3 Hz), 3.93 (1H, d, *J* = 10.8 Hz), 3.45 (1H, dd, *J* = 6.3, 3.4 Hz), 3.28 (1H, d, *J* = 15.4 Hz), 1.66–1.44 (2H, m), 1.36–1.22 (1H, m), 1.19 (3H, s), 1.12 (3H, s), 0.64 (3H, t, *J* = 7.2 Hz); **^{13}C NMR** (400 MHz, Chloroform-*d*) δ: 172.1, 141.1, 128.3, 127.6, 126.9, 77.1, 63.9, 54.9, 52.8, 36.2, 24.8, 17.8, 16.5, 13.6; **HRMS**-NSI: $[C_{16}H_{23}NO_2+H]^+$ requires: 262.1802, found: 262.1805.

4-Benzyl-3-*d*^{5}-ethyl-5,5-dimethyl-3-propylmorpholin-2-one

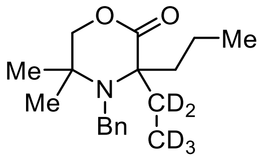

To a solution of 4-benzyl-5,5-dimethyl-3-propylmorpholin-2-one (323 mg, 1.23 mmol) in THF:DME (1:1, 2 mL) at −78 °C was added NaHMDS (2M in THF, 615 µL, 1.23 mmol) dropwise. After stirring at −78 °C for 0.5 h, neat *d5*-ethyl iodide (150 µL, 1.85 mmol) was added dropwise. The reaction was allowed to warm to r.t over 16 h before being quenched with sat. aq. NH_4Cl (5 mL). The reaction mixture was diluted with ethyl acetate (10 mL) and the organic phase separated. The aqueous phase was re-extracted with ethyl acetate (2 x 5mL) and the combined organics dried over $MgSO_4$, filtered and evaporated. The crude material was purified by silica gel column chromatography (20% EtOAc in petroleum ether) to give the title compound as a colourless oil (77%, 277 mg, 0.94 mmol); **TLC** R_f = 0.45 (20% EtOAc in Petroleum ether 40-60 °C); **IR** V_{max}/cm^{-1} (film) 2920, 2876, 1727, 1464, 1452, 1381, 1296, 1144, 1066; **^{1}H NMR** (400 MHz, Chloroform-*d*) δ: 7.35 (2H, d, *J* = 7.4 Hz), 7.30 (2H, t, *J* = 7.5 Hz), 7.22 (1H, t, *J* = 7.1 Hz), 4.09 (2H, q, *J* = 10.8 Hz), 3.93 (2H, s), 1.71 (1H, td, *J* = 12.9, 4.1 Hz), 1.62–1.48 (1H, m), 1.46–1.36 (1H, m), 1.36–1.24 (1H, m), 1.18 (6H, s), 0.82 (3H, t, *J* = 7.1 Hz); **^{13}C NMR** (126 MHz, Chloroform-*d*) δ: 173.9, 143.3, 128.4, 128.4, 127.4, 126.8, 76.5, 68.7, 52.2, 47.9, 42.7, 24.5, 24.13, 19.1, 14.3; **^{2}H NMR** (77 MHz, Chloroform-*d*) δ: 1.80 (1^{2}H, s), 1.57 (1^{2}H, s), 0.93 (3^{2}H, s); **HRMS**-NSI: $[C_{18}H_{22}{}^2H_5NO_2+H]^+$ requires: 295.2428, found: 295.2424.

3-d^5-Ethyl-5,5-dimethyl-3-propylmorpholin-2-one d_5-1a

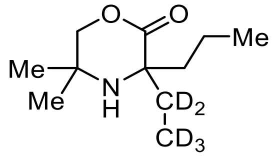

A solution of 4-benzyl-3-d^5-ethyl-5,5-dimethyl-3-propylmorpholin-2-one (277 mg, 0.94 mmol) in methyl acetate (11.5 mL) was subjected to three cycles of vacuum / nitrogen backfill. Palladium on activated carbon (Pd/C, 10 wt. %, 181 mg) was added in one portion, the atmosphere was exchanged for hydrogen and the reaction stirred at room temperature for 16 hours. The reaction was filtered through celite and concentrated *in vacuo* to provide the title compound as a colourless oil (88%, 169 mg, 0.83 mmol). **IR** V_{max}/cm^{-1} (film) 2962, 1727, 1467, 1378, 1285, 1213, 1117, 1058; **^{1}H NMR** (400 MHz, Chloroform-*d*) δ: 4.10 (2H, s), 1.70–1.63 (1H, m), 1.60–1.51 (1H, m), 1.42–1.32 (2H, m), 1.18 (6H, s), 0.92 (3H, t, *J* = 7.3 Hz); **^{2}H NMR** (77 MHz, Chloroform-*d*) δ: 1.73 (1^{2}H, s), 1.63 (1^{2}H, s), 0.89 (3^{2}H, s);**^{13}C NMR** (400 MHz, Chloroform-*d*) δ: 174.3, 77.5, 61.0, 48.7, 43.2, 42.6, 27.0, 26.8, 17.2, 14.5; **HRMS**-NSI: [$C_{11}H_{17}{}^2H_5NO_2$+H]$^+$ requires: 205.1959, found: 205.1956.

General Procedure 6: Acetoxylation of Morpholinones

The morpholinone (1 equiv) was dissolved in acetic acid/acetic anhydride (4:1, 0.1 M), palladium(II) acetate (10 mol%) and PIDA (1.5 equiv) were added to the reaction flask and stirred for a specified time at 70 °C. The reaction was cooled to rt, quenched by the addition of sat. aq. $NaHCO_3$ and extracted with CH_2Cl_2 (3x 20 mL). The organic phases were combined, dried over $MgSO_4$, filtered and concentrated *in vacuo*.

2-(5,5-Dimethyl-2-oxo-3-propylmorpholin-3-yl)ethyl acetate 2a

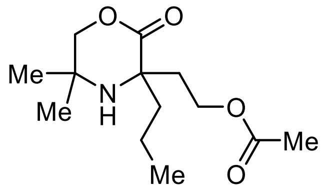

Prepared according to General Procedure **6** using 3-ethyl-5,5-dimethyl-3-propylmorpholin-2-one (60 mg, 0.30 mmol). The reaction was stirred at 70 °C for 1 h. The crude material was purified by flash chromatography (silica gel, 21 cm, 2 cm Ø) eluting with 0-20% EtOAc/CH_2Cl_2 to give the title compound as a yellow oil (58 mg, 0.23 mmol, 75%). **TLC** R_f = 0.56 (20% EtOAc/CH_2Cl_2). **IR** V_{max}/cm^{-1} ($CHCl_3$) 2966, 2876, 17.29, 1465, 1380, 1367, 1284, 1232, 1182, 1126, 1047. **^{1}H NMR** (400 MHz, Chloroform-*d*) δ 4.24 (t, *J* = 6.7 Hz, 2H), 4.10 (s, 2H), 2.10 (dt, *J* = 14.2, 6.7 Hz, 1H), 2.03 (s, 3H), 1.95 (dt, *J* = 14.2, 6.7 Hz, 1H), 1.73 – 1.58 (m, 2H), 1.44 – 1.31 (m, 2H), 1.19 (s, 3H), 1.18 (s, 3H), 0.92 (t, *J* = 7.3 Hz, 3H). **^{13}C NMR** (101 MHz, Chloroform-*d*) δ 173.4, 170.9, 77.6, 60.8, 59.5, 48.4, 43.3, 38.6, 26.7, 26.2, 21.0, 17.2, 14.2. **HRMS**-NSI: [$C_{13}H_{23}NO_4$+H]$^+$ requires: 258.1700, found: 258.1701.

2-(3-Ethyl-5,5-dimethyl-2-oxomorpholin-3-yl)ethyl acetate 2b

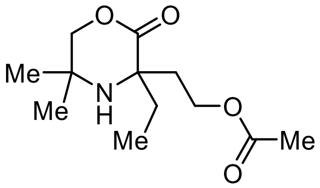

Prepared according to General Procedure **6** using 3,3-diethyl-5,5-dimethylmorpholin-2-one (56 mg, 0.30 mmol). The reaction was stirred at 70 °C for 1 h. The crude material was purified by flash chromatography (silica gel, 11 cm, 2 cm Ø) eluting with 0-20% EtOAc/CH_2Cl_2 to give the title compound as a yellow oil (34 mg, 0.14 mmol, 47%). **TLC** R_f = 0.55 (20% EtOAc/CH_2Cl_2). **IR** V_{max}/cm^{-1} ($CHCl_3$) 2970, 1728, 1460, 1379, 1366, 1232, 1051, 915, 731. **^{1}H NMR** (400 MHz, Chloroform-*d*) δ 4.24 (t, *J* = 6.7 Hz, 2H), 4.11 (s, 2H), 2.10 (dt, *J* = 14.4, 7.2 Hz, 1H), 2.03 (s, 3H), 1.92 (dt, *J* = 14.2, 6.4 Hz, 1H), 1.83 – 1.63 (m, 2H), 1.20 (s, 3H), 1.19 (s, 3H), 0.95 (t, *J* = 7.4 Hz, 3H). **^{13}C NMR** (101 MHz, Chloroform-*d*) δ 173.5, 171.0, 77.7, 60.9, 59.9, 48.6, 38.3, 33.9, 26.9, 26.5, 21.1, 8.4. **HRMS**-NSI: $[C_{12}H_{21}NO_4+H]^+$ requires: 244.1543, found: 244.1545.

(5,5-Dimethyl-2-oxomorpholine-3,3-diyl)bis(ethane-2,1-diyl) diacetate

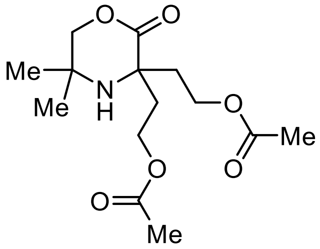

Prepared according to General Procedure **6** using 3,3-diethyl-5,5-dimethylmorpholin-2-one (56 mg, 0.30 mmol). The reaction was stirred at 70 °C for 1 h. The crude material was purified by flash chromatography (silica gel, 11 cm, 2 cm Ø) eluting with 0-20% EtOAc/CH_2Cl_2 to give the title compound as a yellow oil (27 mg, 0.09 mmol, 30%). **TLC** R_f = 0.29 (20% EtOAc/petroleum ether). **IR** V_{max}/cm^{-1} ($CHCl_3$) 2971, 1729, 1366, 1229, 1036, 915, 730. **^{1}H NMR** (400 MHz, Chloroform-*d*) δ 4.25 (t, *J* = 6.5 Hz, 4H), 4.12 (s, 2H), 2.13 (dt, *J* = 14.2, 6.5 Hz, 2H), 2.04 (s, 6H), 1.98 (dt, *J* = 14.2, 6.5 Hz, 2H), 1.20 (s, 6H). **^{13}C NMR** (101 MHz, Chloroform-*d*) δ 172.8, 170.9, 77.7, 60.6, 58.6, 48.6, 39.2, 26.7, 21.1. **HRMS**-NSI: $[C_{14}H_{23}NO_6+H]^+$ requires: 302.1598, found: 302.1600.

2-(5,5-Dimethyl-2-oxo-3-phenethylmorpholin-3-yl)ethyl acetate 2c

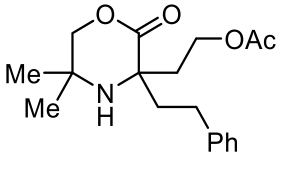

Prepared according to general procedure **6** using 3-ethyl-5,5-dimethyl-3-phenethylmorpholin-2-one (78.0 mg, 0.30 mmol) in acetic acid (2.4 mL). Compound purified by column chromatography (dichloromethane to 20% ethyl acetate in dichloromethane) to yield the title compound as a pale yellow oil (62 mg, 65%). **TLC** R*f* = 0.48 (20% ethyl acetate in dichloromethane); **IR** V_{max}/cm^{-1} (neat film) 2971, 1729, 1603, 1496, 1454, 1366, 1230, 1047; **^{1}H NMR** (400 MHz, Chloroform-*d*) δ: 7.30–7.17

(5H, m), 4.28 (2H, t, *J* = 6.7 Hz), 4.15 (2H, s), 2.75–2.64 (2H, m), 2.20 (1H, dt, *J* = 14.1, 7.0 Hz), 2.08–1.92 (6H, m), 1.23 (3H, s), 1.20 (3H, s); **^{13}C NMR** (101 MHz, Chloroform-*d*) δ: 173.2, 171.0, 141.3, 128.7, 128.5, 126.3, 60.8, 59.7, 48.7, 43.2, 38.6, 30.5, 26.9, 26.6, 21.1; **HRMS**-NSI: $[C_{18}H_{25}NO_4+H]^+$ requires: 320.1856, found: 320.1858.

Ethyl 4-(3-(2-acetoxyethyl)-5,5-dimethyl-2-oxomorpholin-3-yl)butanoate 2d

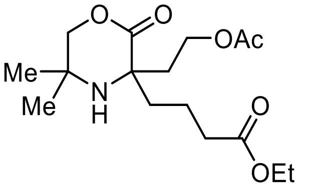

Prepared according to General Procedure **6** using ethyl 4-(3-ethyl-5,5-dimethyl-2-oxomorpholin-3-yl)butanoate (81.0 mg, 0.30 mmol) in acetic acid (2.4 mL). Compound purified by column chromatography (dichloromethane to 20% ethyl acetate in dichloromethane) to yield the title compound as a pale yellow oil (70.3 mg, 71%). **TLC** R*f* = 0.48 (20% ethyl acetate in dichloromethane); **IR** V_{max}/cm^{-1} (neat film) 2970, 1726, 1462, 1367, 1285, 1231, 1178, 1035; **^{1}H NMR** (400 MHz, Chloroform-*d*) δ: 4.24 (2H, t, *J* = 6.7 Hz), 4.16–4.09 (4H, m), 2.35–2.26 (2H, m), 2.16–2.06 (2H, m), 2.03 (3H, s), 2.00–1.90 (1H, m), 1.82–1.64 (4H, m), 1.25 (3H, t, *J* = 7.1 Hz), 1.20 (6H, s); **^{13}C NMR** (101 MHz, Chloroform-*d*) δ: 173.3, 173.3, 171.0, 77.8, 60.8, 60.6, 59. 5, 48.6, 40.3, 38.7, 34.0, 26.7, 26.6, 21.1, 19.3, 14.4; **HRMS**-NSI: $[C_{16}H_{27}NO_6+H]^+$ requires: 330.1911, found: 330.1913.

2-(2-Ethyl-3-oxo-4-oxa-1-azaspiro[5.5]undecan-2-yl)ethyl acetate 2e

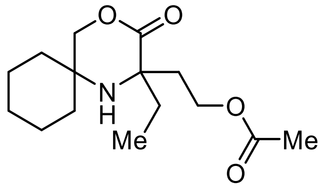

Prepared according to General Procedure **6** using 2,2-diethyl-4-oxa-1-azaspiro[5.5]undecan-3-one (68 mg, 0.30 mmol). The reaction was stirred at 70 °C for 1.5 h. The crude material was purified by flash chromatography (silica gel, 12 cm, 2 cm Ø) eluting with 0-10% EtOAc/CH_2Cl_2 to give the title compound as a yellow oil (42 mg, 0.15 mmol, 49%). **TLC** R_f = 0.71 (20% EtOAc/CH_2Cl_2). **IR** V_{max}/cm^{-1} ($CHCl_3$) 2970, 1729, 1227, 1045, 915, 730. **^{1}H NMR** (400 MHz, Chloroform-*d*) δ 4.26 (t, *J* = 6.8 Hz, 2H), 4.18 (d, *J* = 10.9 Hz, 1H), 4.12 (d, *J* = 10.9 Hz, 1H), 2.10 – 1.94 (m, 5H), 1.81 – 1.33 (m, 12H), 1.24 (br. s, 1H), 0.96 (t, *J* = 7.4 Hz, 3H). **^{13}C NMR** (101 MHz, Chloroform-*d*) δ 173.8, 171.1, 76.4, 61.0, 59.9, 50.0, 38.2, 35.5, 34.8, 33.8, 25.9, 21.8, 21.7, 21.1, 8.4. **HRMS**-NSI: $[C_{15}H_{25}NO_4+H]^+$ requires: 284.1856, found: 284.1858. **Mp** = 62-64 °C.

2-(3-(3-Cyanopropyl)-5,5-dimethyl-2-oxomorpholin-3-yl)ethyl acetate 2f

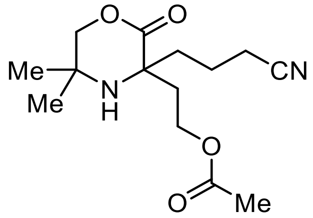

Prepared according to General Procedure **6** using 4-(3-Ethyl-5,5-dimethyl-2-oxomorpholin-3-yl)butanenitrile (60 mg, 0.27 mmol). The reaction was stirred at 70 °C for 2.5 h. The crude material was purified by flash chromatography (silica gel, 14 cm, 2 cm Ø) eluting with 0-20% EtOAc/CH_2Cl_2 to give the title compound as a yellow oil (28 mg, 0.99 mmol, 37%). **TLC** R_f = 0.26 (20% EtOAc/CH_2Cl_2). **IR** V_{max}/cm^{-1} ($CHCl_3$) 2968, 1729, 1460, 1479, 1367, 1285, 1234, 1177, 1048. **^{1}H NMR** (400 MHz, Chloroform-*d*) δ 4.22 (t, J = 6.5 Hz, 2H), 4.12 (s, 2H), 2.41 – 2.33 (m, 2H), 2.10 (dt, J = 14.2, 6.5 Hz, 1H), 2.04 (s, 3H), 1.92 (dt, J = 14.2, 6.5 Hz, 1H), 1.87 – 1.71 (m, 4H), 1.30 (br. s, 1H), 1.19 (s, 6H). **^{13}C NMR** (101 MHz, Chloroform-*d*) δ 172.8, 170.8, 119.3, 77.8, 60.5, 59.2, 48.7, 40.1, 38.5, 26.7, 26.6, 21.0, 20.2, 17.4. **HRMS**-NSI: $[C_{14}H_{22}N_2O_4+H]^+$ requires: 283.1652, found: 283.1655.

2-(5,5-Dimethyl-2-oxo-3-(3-(phenylsulfonyl)propyl)morpholin-3-yl)ethyl acetate 2g

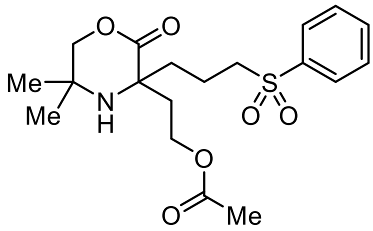

Prepared according to General Procedure **6** using 3-ethyl-5,5-dimethyl-3-(3-(phenylsulfonyl)propyl)morpholin-2-one (97 mg, 0.30 mmol). The reaction was stirred at 70 °C for 2 h. The crude material was purified by flash chromatography (silica gel, 11 cm, 2 cm Ø) eluting with 0-20% EtOAc/CH_2Cl_2 to give the title compound as a light yellow oil (63 mg, 0.16 mmol, 55%).**TLC** R_f = 0.24 (20% EtOAc/CH_2Cl_2). **IR** V_{max}/cm^{-1} ($CHCl_3$) 2972, 1732, 1447, 1367, 1287, 1255, 1147, 1086. **^{1}H NMR** (400 MHz, Chloroform-*d*) δ 7.90 – 7.84 (m, 2H), 7.67 – 7.61 (m, 1H), 7.55 (m, 2H), 4.17 (t, *J* = 6.5 Hz, 2H), 4.06 (s, 2H), 3.11 – 3.03 (m, 2H), 2.10 – 1.97 (m, 4H), 1.91 – 1.69 (m, 5H), 1.15 (s, 3H), 1.12 (s, 3H). **^{13}C NMR** (101 MHz, Chloroform-*d*) δ 172.8, 170.8, 139.1, 133.9, 129.4, 128.0, 77.7, 60.5, 59.3, 56.1, 48.5, 39.4, 38.5, 26.6, 26.5, 21.0, 17.5. **HRMS**-NSI: $[C_{19}H_{27}NO_6S+H]^+$ requires: 398.1632, found: 398.1629.

2-(3-(3-(1,3-dioxoisoindolin-2-yl)propyl)-5,5-dimethyl-2-oxomorpholin-3-yl)ethyl acetate 2h

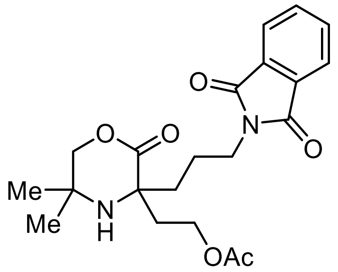

Prepared according to General Procedure **6** using 2-(3-(3-ethyl-5,5-dimethyl-2-oxomorpholin-3-yl)propyl)isoindoline-1,3-dione (45.0 mg, 0.13 mmol) in acetic acid (1.0 mL). Compound purified by column chromatography (dichloromethane to 10% ethyl acetate in dichloromethane) to yield the title compound as a pale yellow oil (42.8 mg, 75%). **TLC** R*f* = 0.28 (10% ethyl acetate in dichloromethane); **IR** V_{max}/cm^{-1} (neat film) 2969, 1771, 1734, 1709, 1467, 1437, 1397, 1366, 1287, 1243, 1186, 1036; **^{1}H NMR** (400 MHz, Chloroform-*d*) δ: 7.84 (2H, dd, *J* = 5.5, 3.0 Hz,), 7.72 (2H, dd, *J* = 5.4, 3.0 Hz), 4.21 (2H, t, *J* = 6.5 Hz), 4.16–4.05 (2H, m), 3.70 (2H, t, *J* = 6.6 Hz), 2.15–2.05 (1H, m), 2.03 (3H, s), 1.91 (1H, dt, *J* = 14.5, 6.3 Hz, 1H), 1.81–1.75 (3H, m), 1.73–1.68 (1H, m), 1.17 (6H, s); **^{13}C NMR** (101 MHz, Chloroform-*d*) δ: 172.9, 170.8, 168.3, 134.0, 132.0, 123.3, 77.6, 60.6, 59.2, 48.5, 38.4, 38.3, 37.9, 26.6, 26.4, 23.2, 20.9; **HRMS**-NSI: $[C_{21}H_{26}N_2O_6+H]^+$ requires: 403.1864, found: 403.1855.

2-(3-((Benzyloxy)methyl)-5,5-dimethyl-2-oxomorpholin-3-yl)ethyl acetate 2i

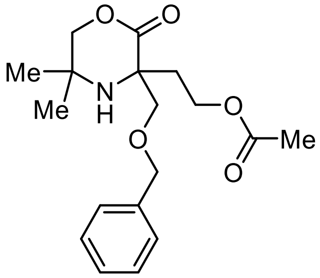

Prepared according to General Procedure **6** using 3-((benzyloxy)methyl)-3-ethyl-5,5-dimethylmorpholin-2-one (83 mg, 0.28 mmol). The reaction was stirred at 70 °C for 2.5 h. The crude material was purified by flash chromatography (silica gel, 11 cm, 2 cm Ø) eluting with 0-10% EtOAc/CH_2Cl_2 to give the title compound as a yellow oil (52 mg, 0.16 mmol, 56%). **TLC** R_f = 0.33 (20% EtOAc/CH_2Cl_2). **IR** V_{max}/cm^{-1} ($CHCl_3$) 2967, 2916, 1732, 1365, 1232, 1097, 1045, 731. **^{1}H NMR** (400 MHz, Chloroform-*d*) δ 7.42 – 7.26 (m, 5H), 4.56 (d, *J* = 12.0 Hz, 1H), 4.52 (d, *J* = 12.0 Hz, 1H), 4.26 (t, *J* = 6.3 Hz, 2H), 4.11 (d, *J* = 10.5 Hz, 1H), 4.06 (d, *J* = 10.5 Hz, 1H), 3.67 (d, *J* = 9.0 Hz, 1H), 3.49 (d, *J* = 9.0 Hz, 1H), 2.14 – 1.97 (m, 5H), 1.81 (br. s, 1H), 1.17 (s, 3H), 1.14 (s, 3H). **^{13}C NMR** (101 MHz, Chloroform-*d*) δ 172.5, 171.0, 137.6, 128.7, 128.1, 127.9, 77.8, 75.6, 73.6, 60.7, 60.0, 48.3, 37.5, 26.2, 26.1, 21.0. **HRMS**-NSI: $[C_{18}H_{25}NO_5+H]^+$ requires: 336.1805, found: 336.1809.

2-(4-(4-Cyanophenyl)-2-ethyl-6,6-dimethyl-3-oxopiperazin-2-yl)ethyl acetate 2j

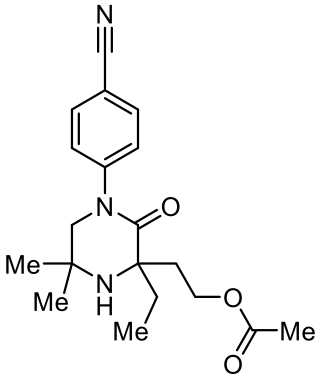

Prepared according to General Procedure **6** using **1j** (85.5 mg, 0.3 mmol), palladium(II) acetate (10.0 mg, 0.045 mmol) in dichloromethane/acetic anhydride (3 mL, 4:1 v/v). The crude product was purified by column chromatography (dichloromethane to 20% ethyl acetate in dichloromethane) to afford the title compound as a yellow oil (67 mg, 65%). **TLC** R_f = 0.26 (20% EtOAc/CH_2Cl_2). **IR** V_{max}/cm^{-1} ($CHCl_3$) 2970, 1733, 1656, 1600, 1505, 1313, 1235, 1177, 1033, 845, 731. **^{1}H NMR** (400 MHz, Chloroform-*d*) δ 7.70 – 7.65 (m, 2H), 7.48 – 7.41 (m, 2H), 4.29 (td, *J* = 6.8, 2.9 Hz, 2H), 3.57 (app. d, *J* = 1.4 Hz, 2H), 2.16 – 2.06 (m, 1H), 2.04 – 1.97 (m, 4H), 1.86 (dq, *J* = 14.5, 7.4 Hz, 1H), 1.72 (dq, *J* = 14.5, 7.4 Hz, 1H), 1.30 (s, 3H), 1.28 (s, 3H), 0.98 (t, *J* = 7.4 Hz, 3H). **^{13}C NMR** (101 MHz, Chloroform-*d*) δ 172.7, 171.1, 147.2, 133.0, 125.9, 118.6, 109.8, 61.5, 61.3, 61.1, 49.2, 38.4, 33.7, 28.3, 27.8, 21.2, 8.4. **HRMS**-NSI: $[C_{19}H_{25}N_3O_3+H]^+$ requires: 344.1969, found: 344.1970.

3-((1-hydroxy-2-methylpropan-2-yl)amino)-3-propyldihydrofuran-2(3H)-one

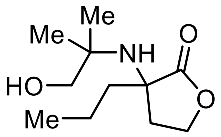

A 10 mL microwave vial equipped with stir bar was charged with SX (51.4 mg, 0.2 mmol). To this was added concentrated hydrochloric acid (1.0 mL, 12 M) and the vial was sealed. The reaction mixture was heated at 90 °C for 16 h before being allowed to cool to room temperature. Mixture was neutralized to pH 6 by addition of solid sodium carbonate, diluted with dichloromethane (5 mL) and extracted. Organic layer was dried and concentrated *in vacuo* . The crude material was purified by flash column chromatography (dichloromethane to 20% ethyl acetate in dichloromethane) to provide the title compound as a colourless oil (30.9 mg, 72% yield). **IR** V_{max}/cm^{-1} (neat film) 2967, 2936, 1730, 1464, 1379, 1285, 1220, 1147, 1060; **^{1}H NMR** (400 MHz, Chloroform-*d*) δ: 4.51 (1H, d, *J* = 11.5 Hz), 4.06 (1H, d, *J* = 11.5 Hz), 3.34 (1H, dt, *J* = 9.7, 8.3 Hz), 3.24 (1H, td, *J* = 8.1, 5.0 Hz), 2.37–2.24 (m, 2H), 1.87-1.78 (1H, m), 1.77 – 1.63 (2H, m), 1.53 (2H, m), 1.25 (1H, s, NH), 1.12 (3H, s), 1.01 (3H, s), 0.95 (3H, t, *J* = 7.3 Hz); **^{13}C NMR** (101 MHz, Chloroform-*d*) δ: 175.6, 73.3, 66.0, 50.0, 42.9, 42.3, 26.8, 25.3, 21.1, 17.2, 14.5; **HRMS**-NSI: $[C_{11}H_{21}NO_3+H]^+$ requires: 216.1594, found: 216.1593.

2-(4-(4-(aminomethyl)phenyl)-2-ethyl-6,6-dimethylpiperazin-2-yl)ethanol 3

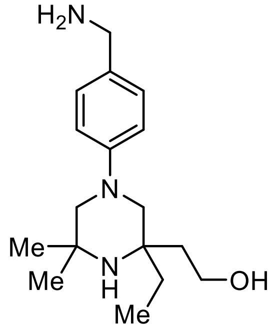

An oven dried 25 mL round bottom flask equipped with a condenser and stir bar was charged with **2j** (67.1 mg, 0.2 mmol) and anhydrous tetrahydrofuran (3 mL). To this was added lithium aluminium hydride (40 mg, 1 mmol) at room temperature portionwise. After addition complete, mixture was heated at 60 °C for 3 h then cooled to room temperature. The mixture was quenched with a saturated solution of Rochelle's salt (2 mL) and stirred for 16 h. Organic layer was extracted with dichloromethane (3 x 5 mL), dried (Na_2SO_4) and concentrated *in vaacuo* . The crude mixture was purified using SCX-2 column eluting with a solution of ammonia in methanol (3.5 M) to yield the title compound as a pale yellow oil (42 mg, 72% yield). **IR** V_{max}/cm^{-1} (neat film) 2960, 2916, 2865, 1613, 1515, 1457, 1383; **^{1}H NMR** (400 MHz, Chloroform-*d*) δ: 7.21 (2H, d, *J* = 8.4 Hz), 6.90–6.83 (2H, m), 3.95–3.80 (2H, m), 3.79 (2H, s), 3.18 (1H, d, *J* = 12.1 Hz), 2.96 (1H, t, *J* =10.8 Hz), 2.90–2.80 (2H, m), 1.97–1.84 (2H, m), 1.64-1.56 (2H, m), 1.50-1.38 (2H, m), 1.29 (3H, s), 1.24 (3H, s), 0.90 (3H, t, *J* = 7.5 Hz); **^{13}C NMR** (101 MHz, Chloroform-*d*) δ: 151.2, 134.6, 128.0, 116.8, 61.3, 59.8, 58.0, 56.5, 50.8, 46.0, 35.5, 32.2, 30.3, 30.0, 8.1; **HRMS**-NSI: $[C_{17}H_{29}N_3O+H]^+$ requires: 292.2383, found 292.2380.

Trinuclear five-membered ring palladacycle

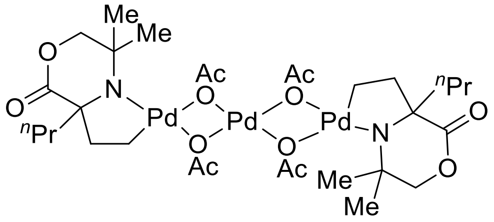

3-Ethyl-5,5-dimethyl-3-propylmorpholin-2-one (99.5 mg, 0.5 mmol) and palladium(II) acetate (168 mg, 0.75 mmol) were stirred in chloroform (4 mL) at 60 °C for 16 hours under air. The solution was cooled to room temperature and filtered through celite, eluting with chloroform. The solvent was then removed in vacuo and the residue was re-dissolved in a minimum amount of chloroform. This solution was added dropwise to 40-60 ºC petroleum ether (15 mL) and the resulting precipitate was filtered, washed with 40-60 ºC petroleum ether and dried in vacuo to afford the title palladacycle as a green solid (147 mg, 30% yield).

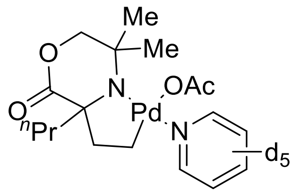

Mononuclear five-membered ring palladacycle-d_5-pyridine complex: Resolved NMR spectra were obtained by treating the trinuclear palladacycle int-V with pyridine-d_5 (2 drops added to 15 mg of palladacycle in $CDCl_3$) to generate the corresponding pyridine complex. **^{1}H NMR** (400 MHz, CDCl3) δ 4.07 (2H, q, *J* = 11.7 Hz), 2.74 (1H, td, *J* = 12.8, 12.3, 3.9 Hz), 2.43-2.35 (1H, m), 2.02-1.95 (1H, m), 1.85 (3H, s), 1.76 (4H,s), 1.68 (3H, s), 1.29 (3H, s), 0.97 (3H, t, *J* = 7.1 Hz), 0.75 (1H, m). **^{13}C NMR** (100 MHz, CDCl3) δ 179.2, 178.2, 170.7, 151.1 (t, *J* = 28.3 Hz), 149.3 (t, *J* = 27.1 Hz), 138.1 (t, *J* = 25.5 Hz), 135.4 (t, *J* = 24.7 Hz), 124. 3 (t, *J* = 25.8 Hz), 123.1 (t, *J* = 24.9 Hz), 76.4, 73.1, 53.9, 45.4, 40.6, 25.3, 25.2, 24.0, 23.1, 18.9, 17.9, 14.2.

Stoichiometric C–H acetoxylation

A 5 mL vial equipped with a magnetic stir bar was charged with palladacycle (0.02 mmol), $PhI(OAc)_2$ (38 mg, 0.12 mmol), and 1,2-dichloroethane (0.5 mL). Then the vial was sealed under air with a screw cap and Teflon septum, and placed in a pre-heated oil bath at 70 ºC stirred for 1 hours. The reaction mixture was cooled to room temperature and filtered through celite, eluting with ethyl acetate. Yields were determined by 1H NMR against 1,1,2,2-tetrachloroethylene as internal standard.

Kinetics data

Order in PIDA - Table S1

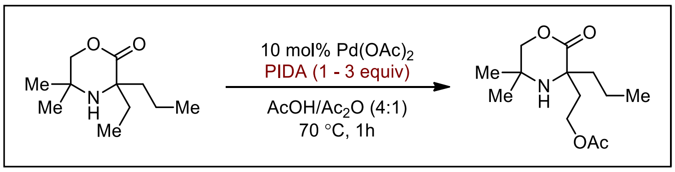

An oven dried 25 mL round bottom flask equipped with a stir bar was charged with **1b** (59 mg, 0.3 mmol, 1.0 equiv), palladium acetate (6.6 mg, 0.03 mmol), PhI(OAc)$_2$ (1.0–3.0 equiv), 1,1,2,2-tetrachloroethane (30.6 μL, internal standard) and AcOH/Ac$_2$O (4:1) (3.0 ml). The vial was sealed with a Teflon cap, placed in a pre-heated oil bath at 70 °C and aliquots were analysed by GC/FID over 1 hour.

Entry	Equivalents of PIDA	Rate (M s^{-1})
1	3.0	1.8×10^{-3}
2	2.0	2.0×10^{-3}
3	1.5	1.9×10^{-3}
4	1.0	1.7×10^{-3}

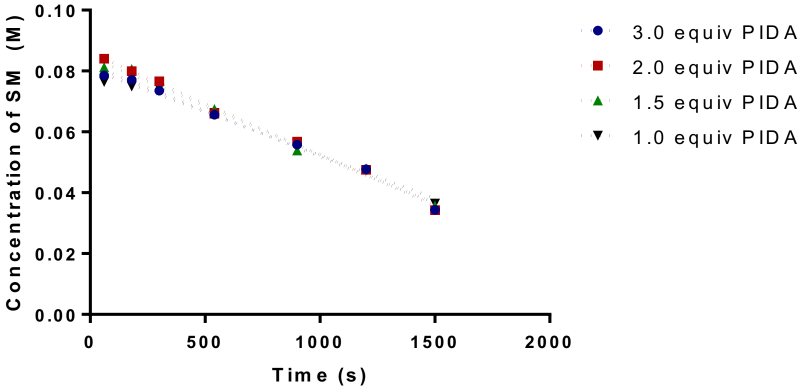

Table S1. Negligible change in rate is seen on alteration of the oxidant concentration. Conditions: substrate (0.10 M), Pd(OAc)$_2$ (0.01 M), oxidant, 3 mL AcOH/Ac$_2$O (4:1) , 70 °C

Order in Palladium acetate - Table S2

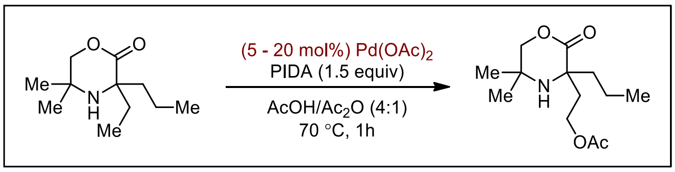

An oven dried 25 mL round bottom flask equipped with a stir bar was charged with **1b** (59 mg, 0.3 mmol, 1.0 equiv), palladium acetate (5–20 mol%), $PhI(OAc)_2$ (145 mg, 1.5 equiv), 1,1,2,2-tetrachloroethane (30.6 μL, internal standard) and $AcOH/Ac_2O$ (4:1) (3.0 ml). The vial was sealed with a Teflon cap, placed in a pre-heated oil bath at 70 °C and aliquots were analysed by GC/FID over 1 hour.

Variation of initial rates with concentration of catalyst. Conditions: substrate (0.10 M), catalyst, $PhI(OAc)_2$ (0.15 M), 3 ml $AcOH/Ac_2O$ (4:1), 70 °C

Concentration of $Pd(OAc)_2$ /M	Rate 1	Rate 2	Rate 3	Average rate	Standard deviation
0.0045	1.646	1.712	1.3787	1.579	0.176491
0.00907	2.1497	2.2835	1.834	2.089	0.230803
0.014	2.024	2.4932	2.4662	2.328	0.263445
0.018	2.5604	2.4311	2.2997	2.430	0.130351

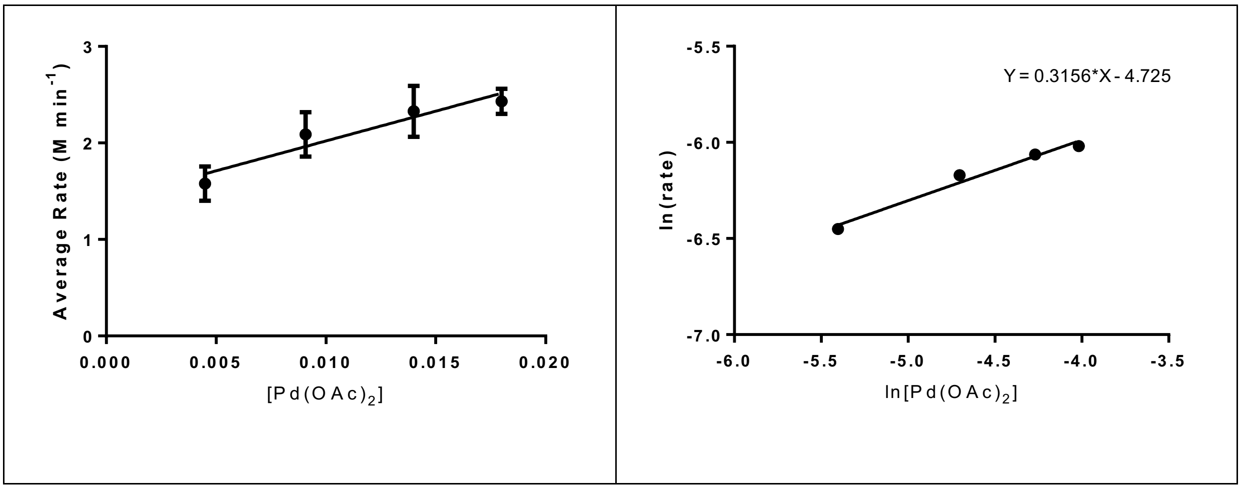

Table S2. The plot of $[Pd(OAc)_2]$ vs average rate indicates saturation type kinetics (left), with the plot of ln[Pd(OAc)2] vs ln(rate) indicating an order of 0.3 in palladium (right).

Order in Amine - Table S3

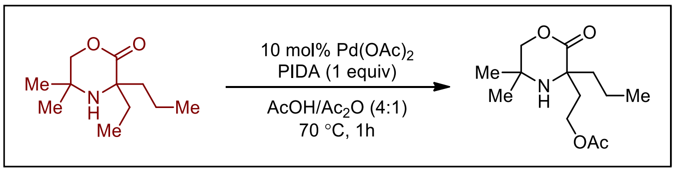

An oven dried microwave vial equipped with a stir bar was charged with $PhI(OAc)_2$ (145 mg, 0.45 mmol, 1.5 equiv), 1,1,2,2-tetrachloroethane (30.6 µL), stock A (x ml), stock B (1.5 ml) and $AcOH/Ac_2O$ (4:1) (y ml). The vial was sealed with a Teflon cap, placed in a pre-heated oil bath at 70 °C and aliquots were analysed by GC/FID for 1 hour.

Stock solutions made up in volumetric flasks:
Stock A: 0.30 M amine and 0.30 M 1,1,2,2-tetrachloroethane in $AcOH/Ac_2O$ (4:1)
Stock B: 0.01 M $Pd(OAc)_2$ in $AcOH/Ac_2O$ (4:1)
(0.075 M): x = 0.750; y = 0.750
(0.100 M): x = 1.000; y = 0.500
(0.125 M): x = 1.250; y = 0.250
(0.150 M): x = 1.500; y = 0.000

[1] / M	1/[1] M^{-1}	Rate 1	Rate 2	Rate 3	Average rate	Standard deviation
0.075	13.3	2.998	3.0366	2.9663	3.0003	0.035206
0.1	10	2.7185	2.6163	2.7511	2.6953	0.070331
0.125	8	2.4427	2.859	2.7267	2.676133	0.212707
0.15	6.7	2.5356	2.618	2.6392	2.5976	0.05473

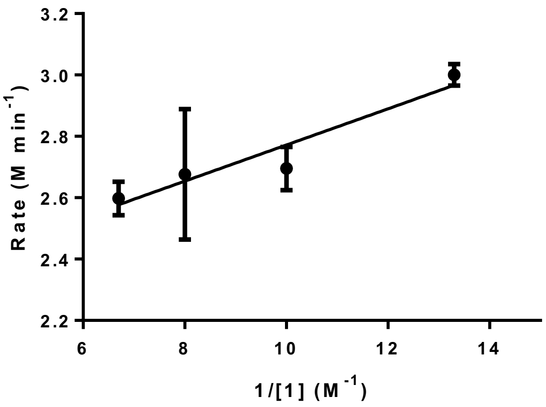

Table S3. The straight line of the graph also confirms the initial order of substrate to be -1st with respect to reaction.

Kinetic isotope effect study

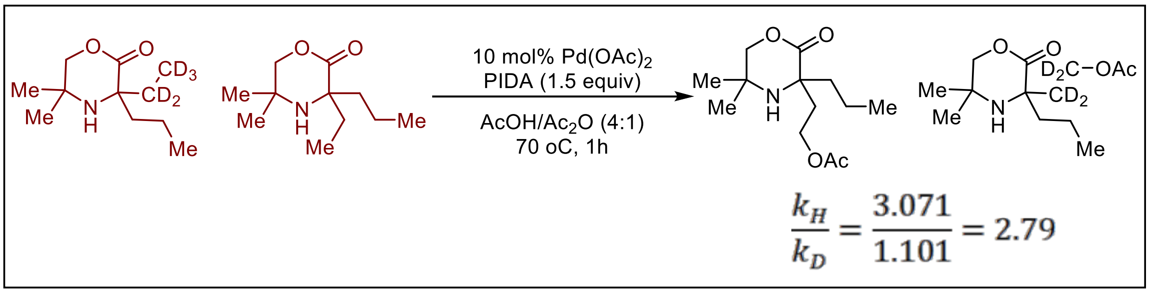

An oven dried 25 mL round bottom flask equipped with a stir bar was charged with **1b** (59 mg, 0.3 mmol, 1.0 equiv) OR **1b-d$_5$** (61.3 mg, 0.3 mmol). Palladium acetate (6.6 mg, 0.03 mmol, 10 mol%), $PhI(OAc)_2$ (145 mg, 1.5 equiv), 1,1,2,2-tetrachloroethane (30.6 μL, internal standard) and $AcOH/Ac_2O$ (4:1) (3.0 ml) were added to the flask and sealed Teflon cap. The vessel was placed in a pre-heated oil bath at 70 °C and aliquots were analysed by GC/FID over 1 hour.

Same excess experiments - Table S4

We ran the reaction at 20% completion. If we were to see either product inhibition or catalyst deactivation, then the reaction starting at 20% completion would be faster than that started at 0% completion. Time adjusting the 20% completion reaction and overlaying onto the 0% completion plot, such that the initial amine concentrations are equivalent, indicates there are negligible differences between the two plots. We also tested the effect of adding the product (0.2 equivalents of acetoxylated product) to the 20% completion reaction and again negligible differences were observed.

Time (min)	Starting material concentration (M)	
	Normal run	20% completion
3.0	0.106753	
6.0	0.101705	
9.0	0.098958	
12.0	0.088567	
15.0	0.081128	
18.0	0.075796	0.077382
21.0	0.069932	0.070547
24.0	0.064225	0.065332
27.0	0.059480	0.061176
30.0	0.053343	0.055854
33.0	0.049593	0.051381
36.0	0.045573	0.048000
39.0	0.042769	0.042888
42.0	0.038853	0.038736
45.0	0.036339	0.035608

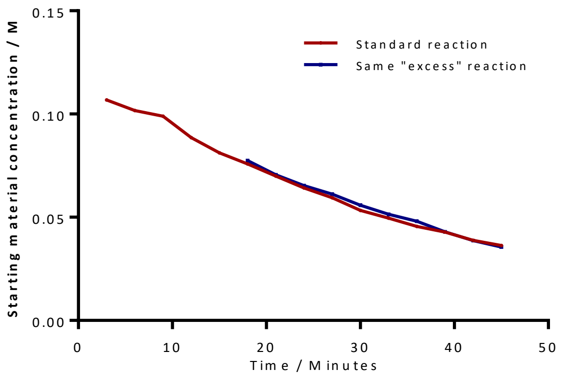

Standard reaction

1a + $PhI(OAc)_2$ —[$Pd(OAc)_2$ (0.1 equiv)]→ 2a + $PhI(OAc)_2$ + PhI

(1.0 equiv) (1.5 equiv) (1.0 equiv) (0.5 equiv) (1.0 equiv)

Same "excess" reaction

1a + $PhI(OAc)_2$ —[$Pd(OAc)_2$ (0.1 equiv)]→ 2a + $PhI(OAc)_2$ + PhI

(0.8 equiv) (1.3 equiv) (0.8 equiv) (0.5 equiv) (0.8 equiv)

Preliminary enantioselectivity study

Table 2 in manuscript

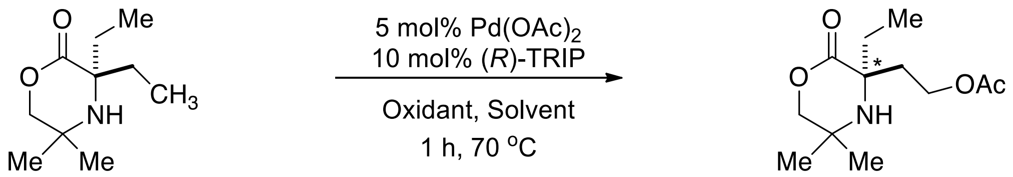

Entry	Oxidant	Solvent	Yield (%)	ee (%)
1	$PhI(OAc)_2$	$AcOH/Ac_2O$	70	-
2	$PhI(OAc)_2$	$MeNO_2$	42	-
3	$PhI(OAc)_2$	EtOAc	-	-
4	$PhI(OAc)_2$	1,2-DCE	-	-
5	$PhI(OAc)_2$	CH_2Cl_2	56	5
6	I_2/AgOAc	CH_2Cl_2	39	70
7	I_2/AgOAc	CH_2Cl_2	33	50

A microwave vial equipped with a stir bar was charged with (*R*)-TRIP (7.5 mg, 0.01 mmol, 0.1 equiv.) and AgOAc (50.1 mg, 0.300 mmol, 3.0 equiv). A solution of Pd(OAc)2 (1.1 mg, 0.005 mmol, 0.05 equiv.) in dichloromethane (1 mL) was added and the vial was sealed with a Teflon cap and placed in an oil bath pre-heated to 70 °C. After 1 minute, a solution of iodine (50.8 mg, 0.200 mmol, 2.0 equiv.) and substrate (0.100

mmol, 1.0 equiv.) in dichloromethane (2 mL) was added. The reaction was heated at this temperature for 1 hour. After the reaction was complete, the crude mixture was cooled to room temperature and filtered through a plug of silica (1.0 cm), eluting with dichloromethane (6 × 1 mL). The filtrate was concentrated in vacuo then the residue was purified by flash column chromatography to afford the acetoxylated product in 39% yield with 70%ee.

HPLC Analysis

Column: Chiralpak IC
Solvent: n-hexane/ iPrOH (85:15)
Flow rate: 1.0 mL min^{-1}
Temperature: 30 °C
λmax : 254 nm

Racemic:

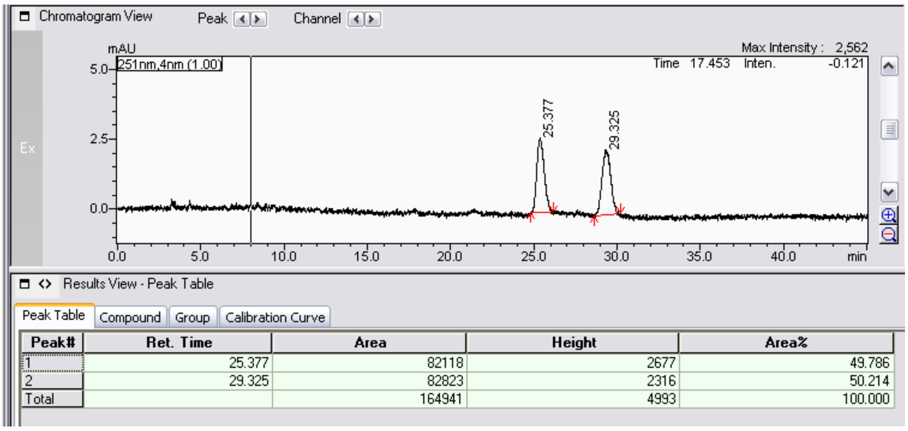

Peak#	Ret. Time	Area	Height	Area%
1	25.377	82118	2677	49.786
2	29.325	82823	2316	50.214
Total		164941	4993	100.000

Chiral:

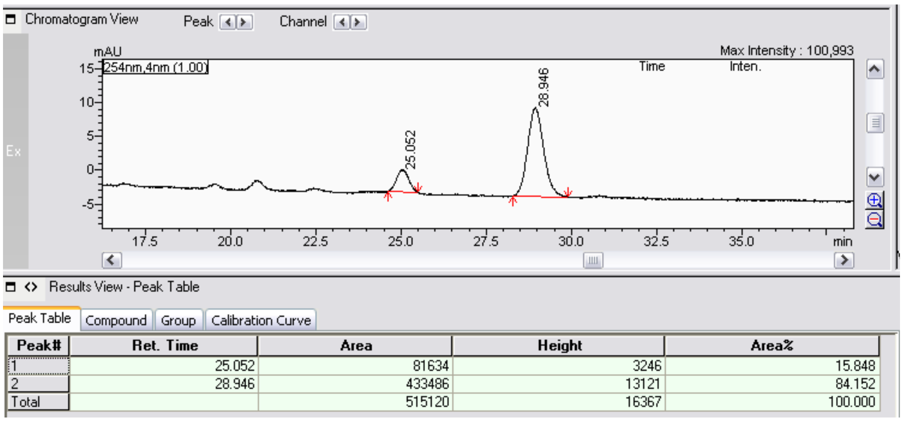

Peak#	Ret. Time	Area	Height	Area%
1	25.052	81634	3246	15.848
2	28.946	433486	13121	84.152
Total		515120	16367	100.000

References

1. *Chem. Sci.*, 2017,**8**, 3586-3592.
2. *J. Am. Chem. Soc.*, 2015, **137** (33), 10632–10641.

3-Ethyl-5,5-dimethyl-3-propylmorpholin-2-one 1a

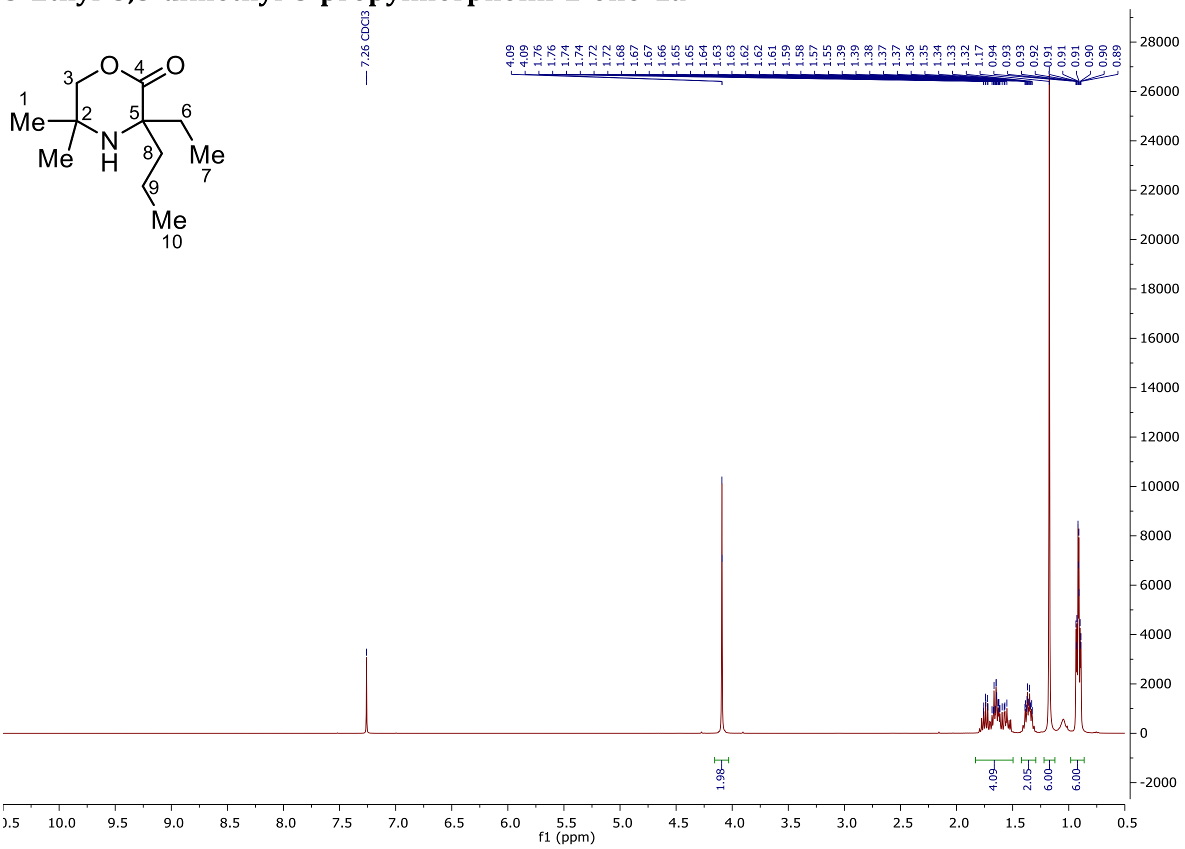

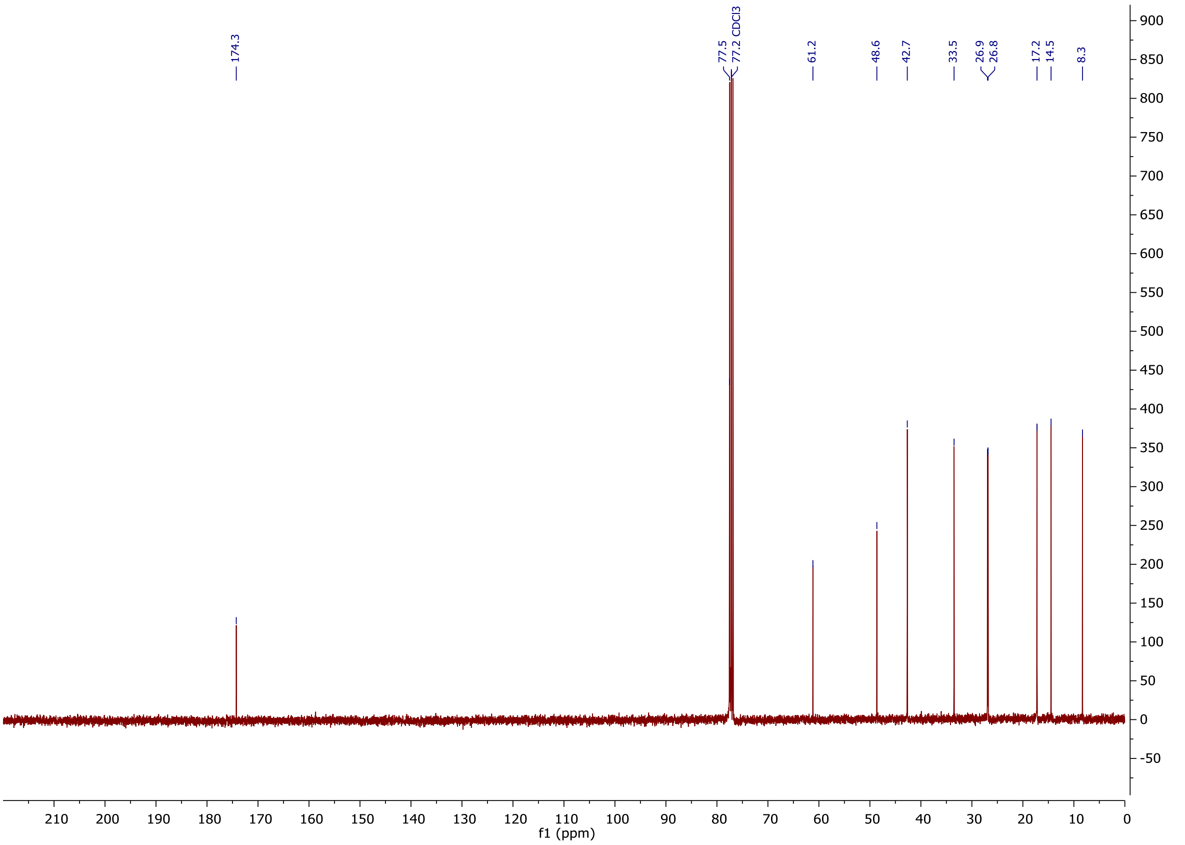

3,3-Diethyl-5,5-dimethylmorpholin-2-one 1b

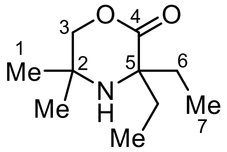

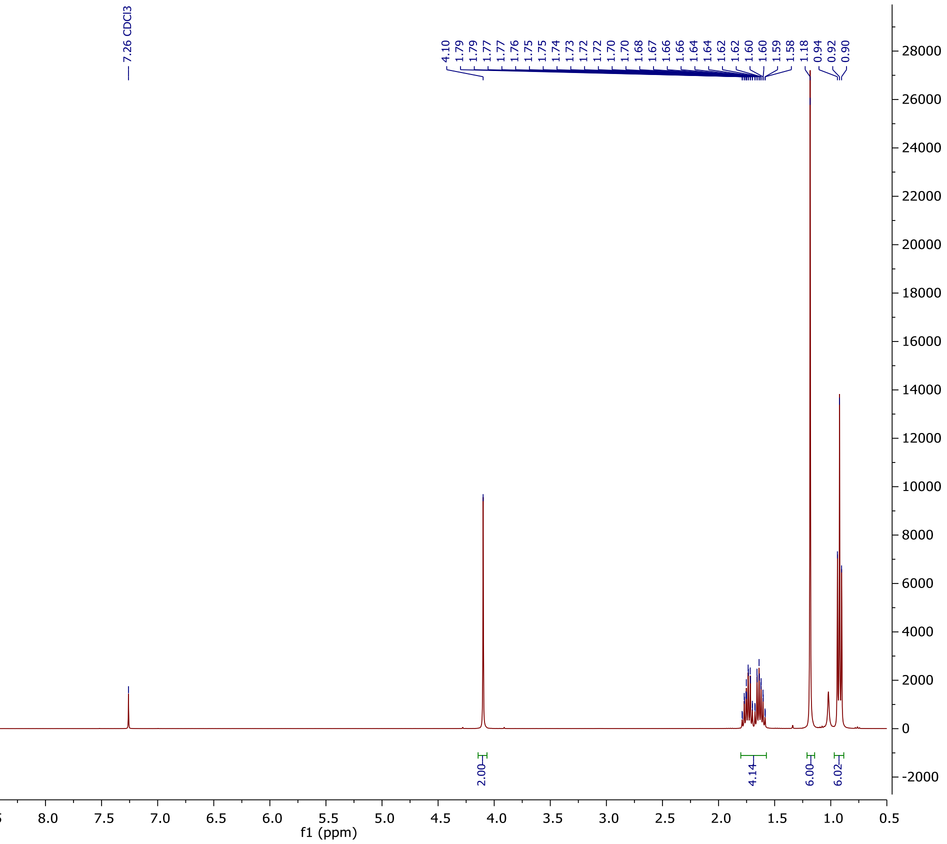

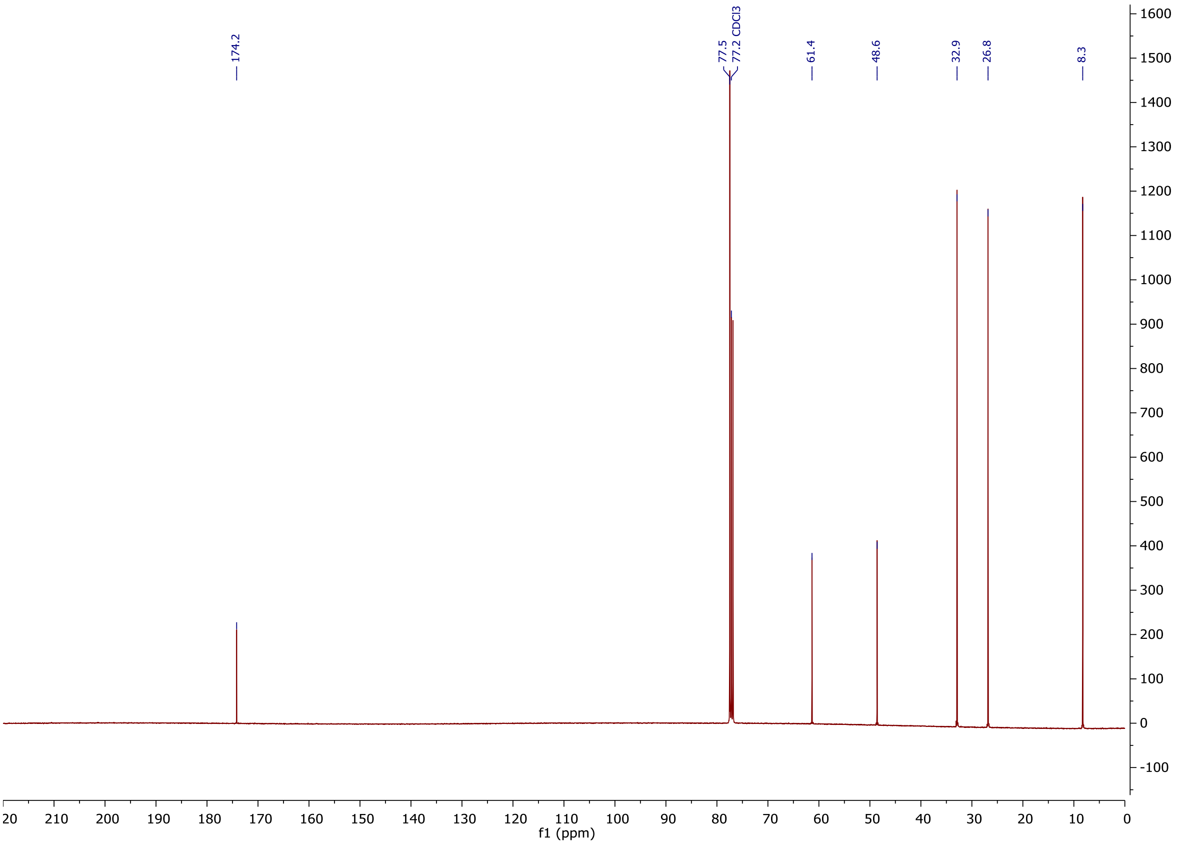

3-Ethyl-5,5-dimethyl-3-phenethylmorpholin-2-one 1c

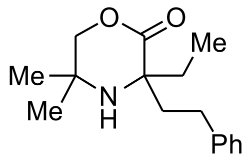

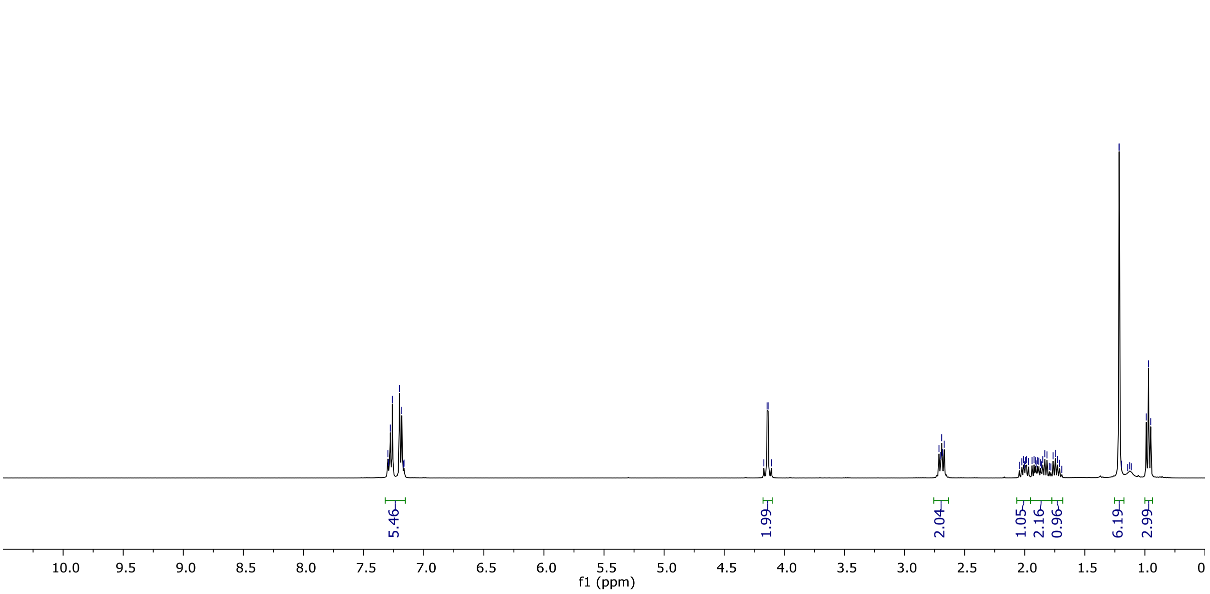

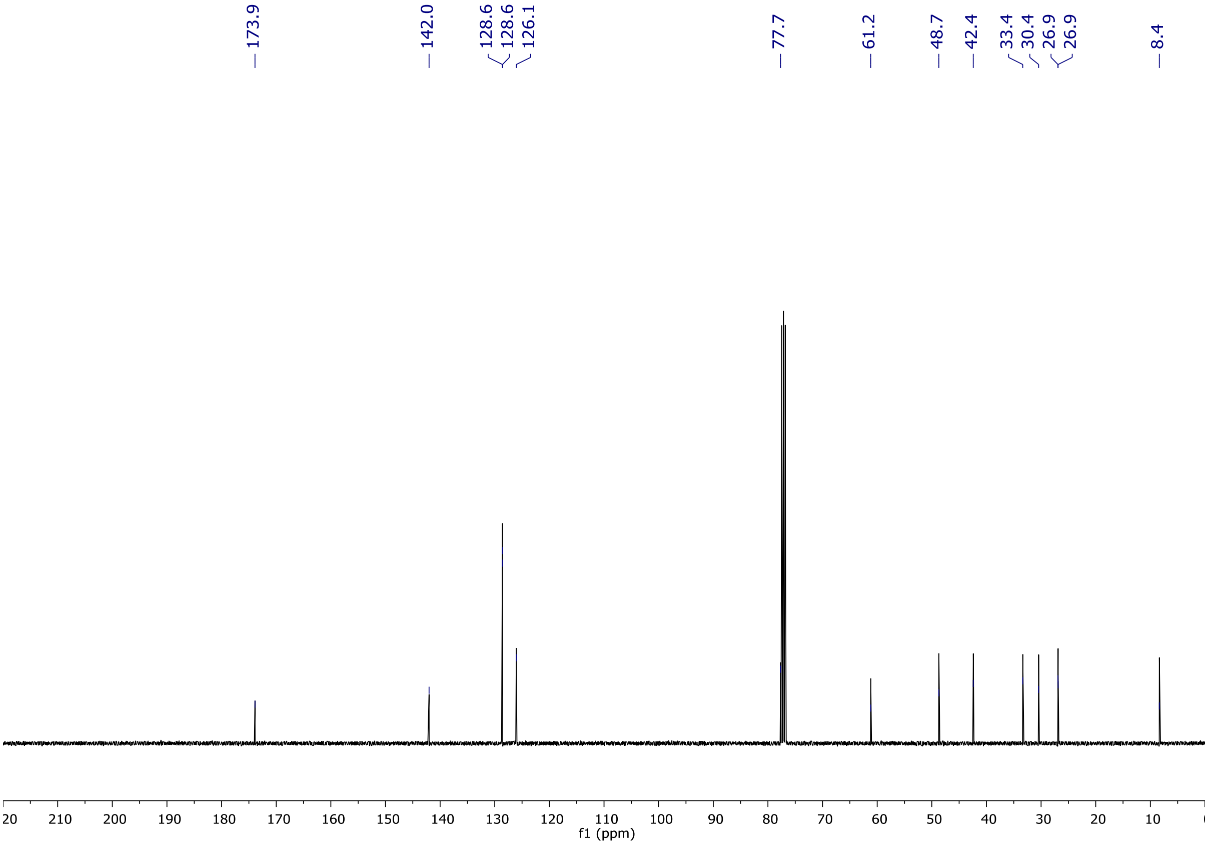

4-Benzyl-5,5-dimethylmorpholin-2-one

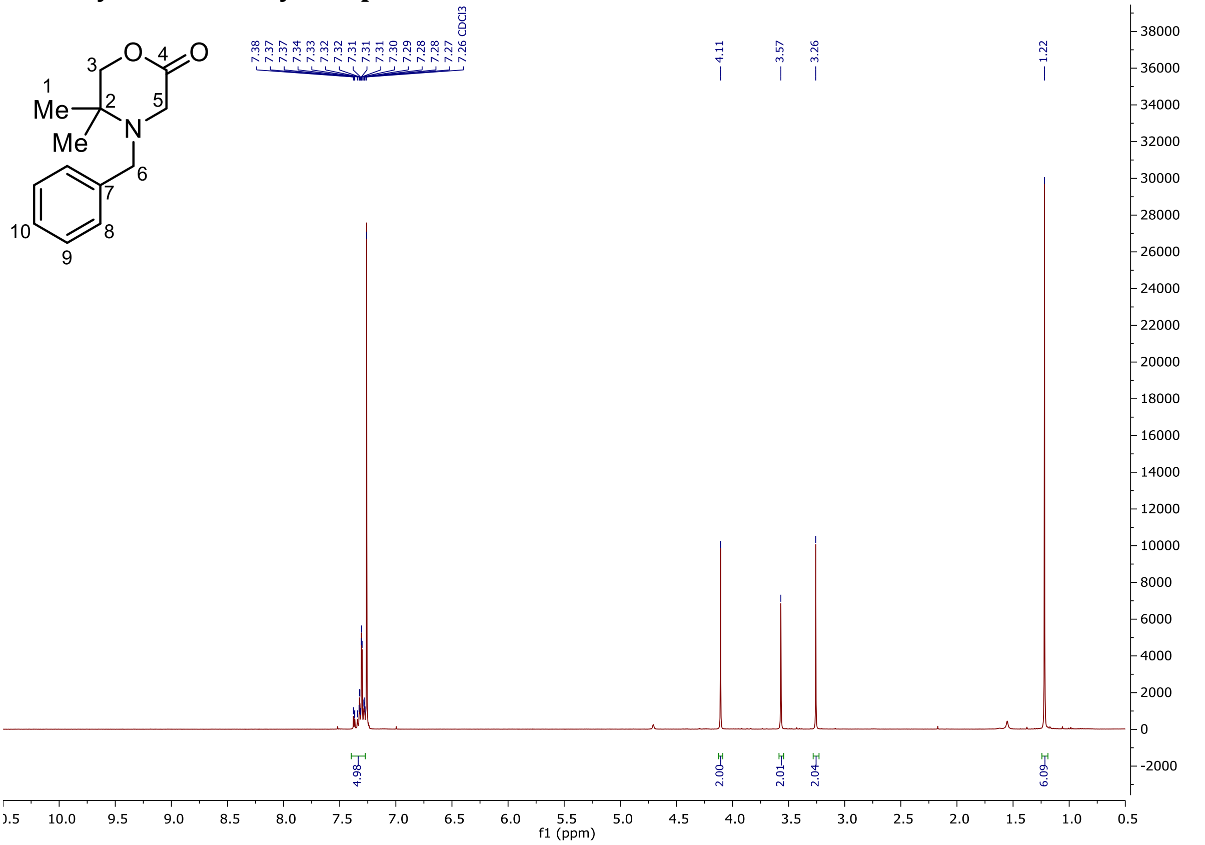

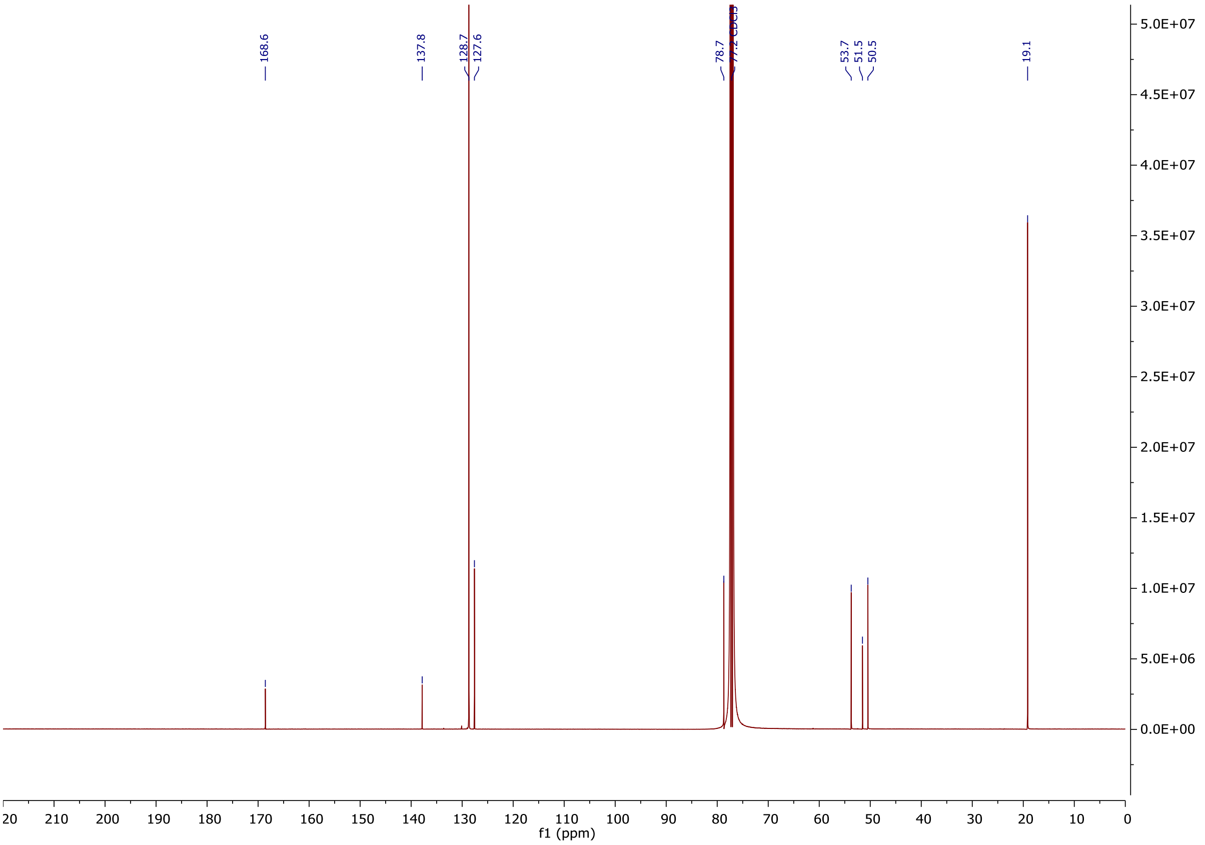

4-Benzyl-3-ethyl-5,5-dimethylmorpholin-2-one

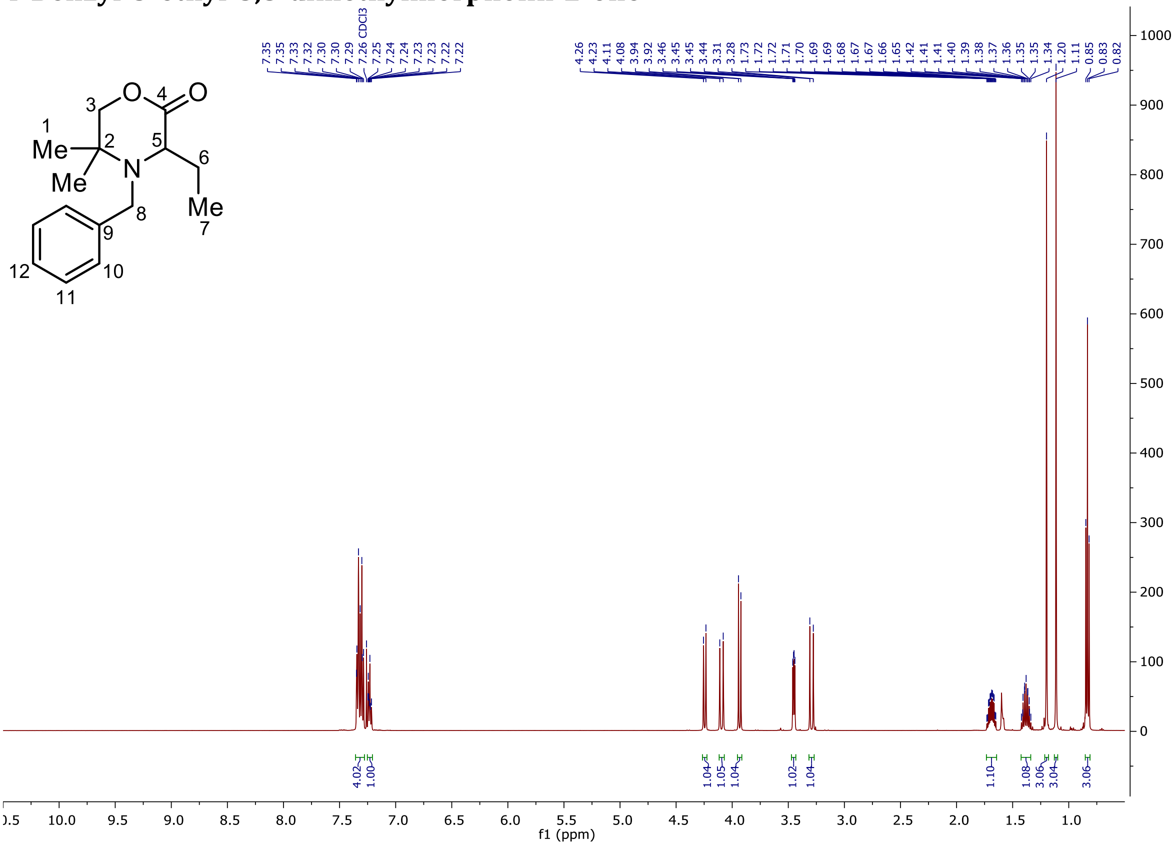

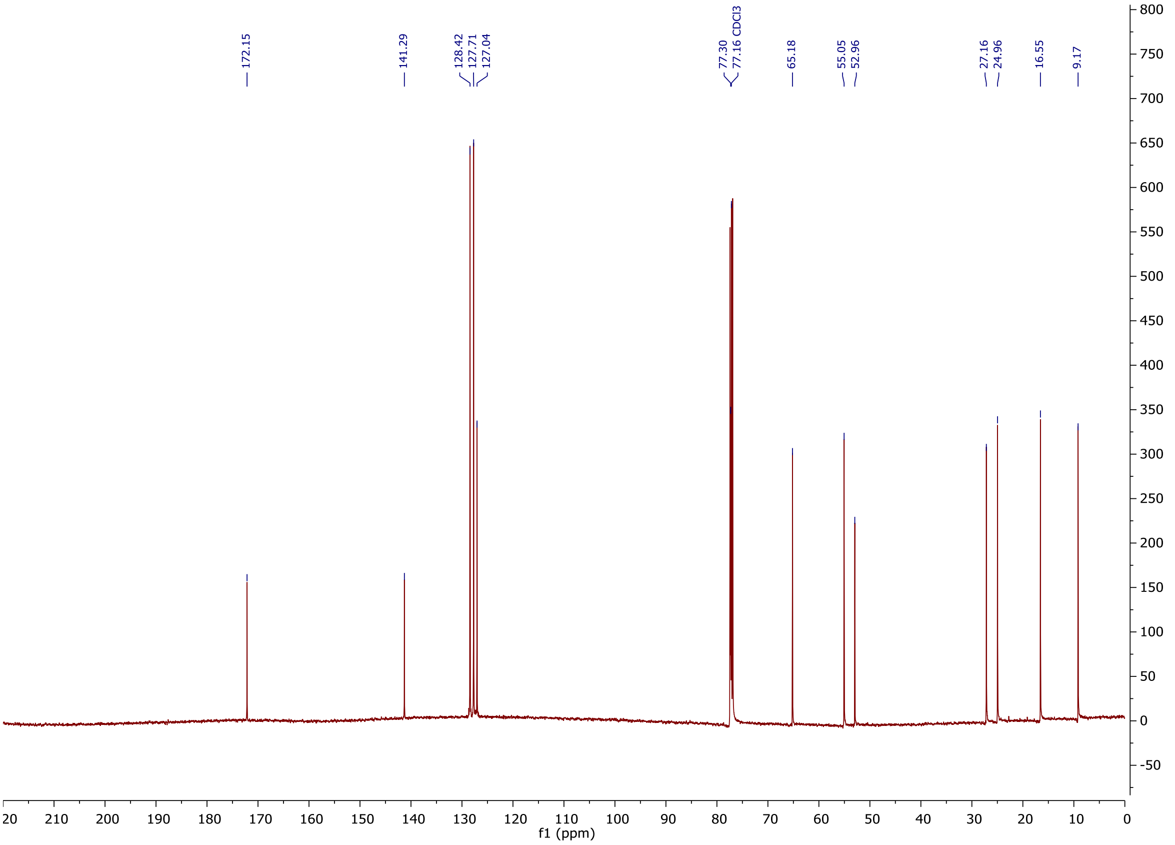

3-Allyl-4-benzyl-3-ethyl-5,5-dimethylmorpholin-2-one

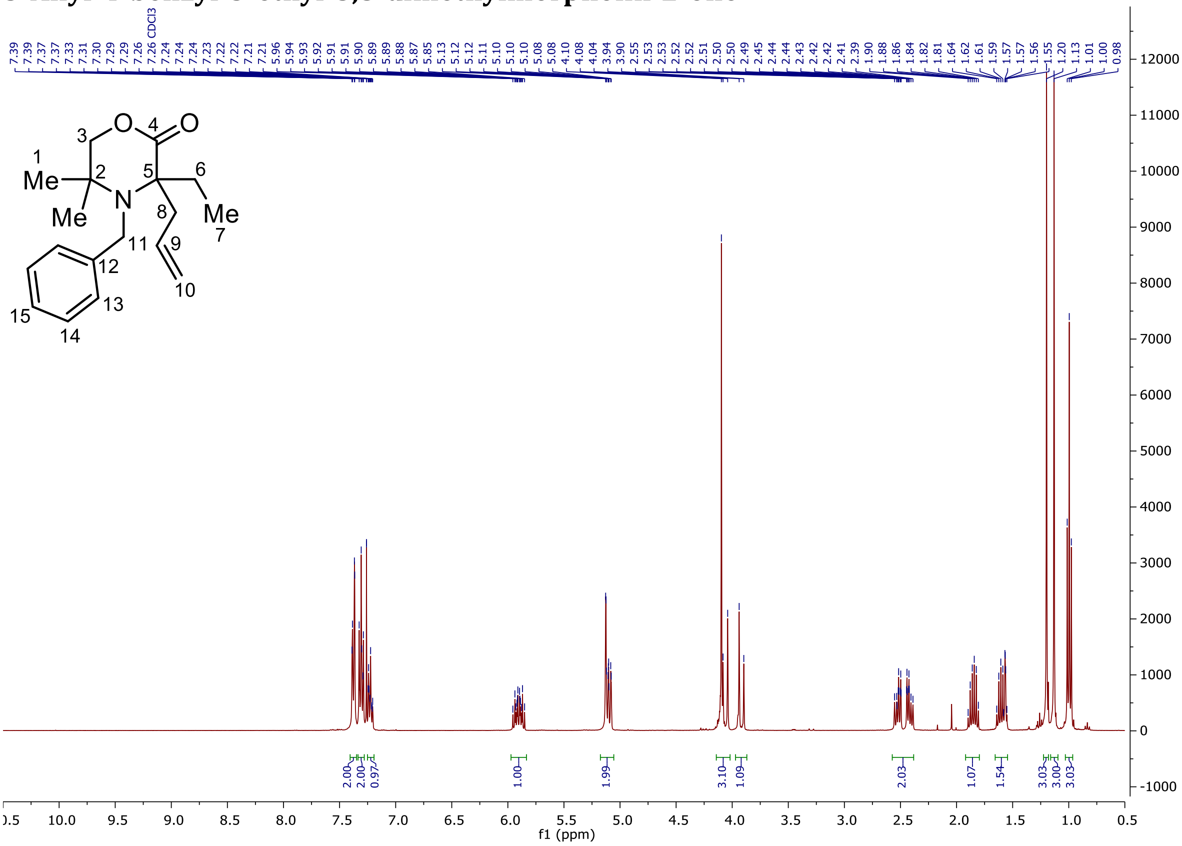

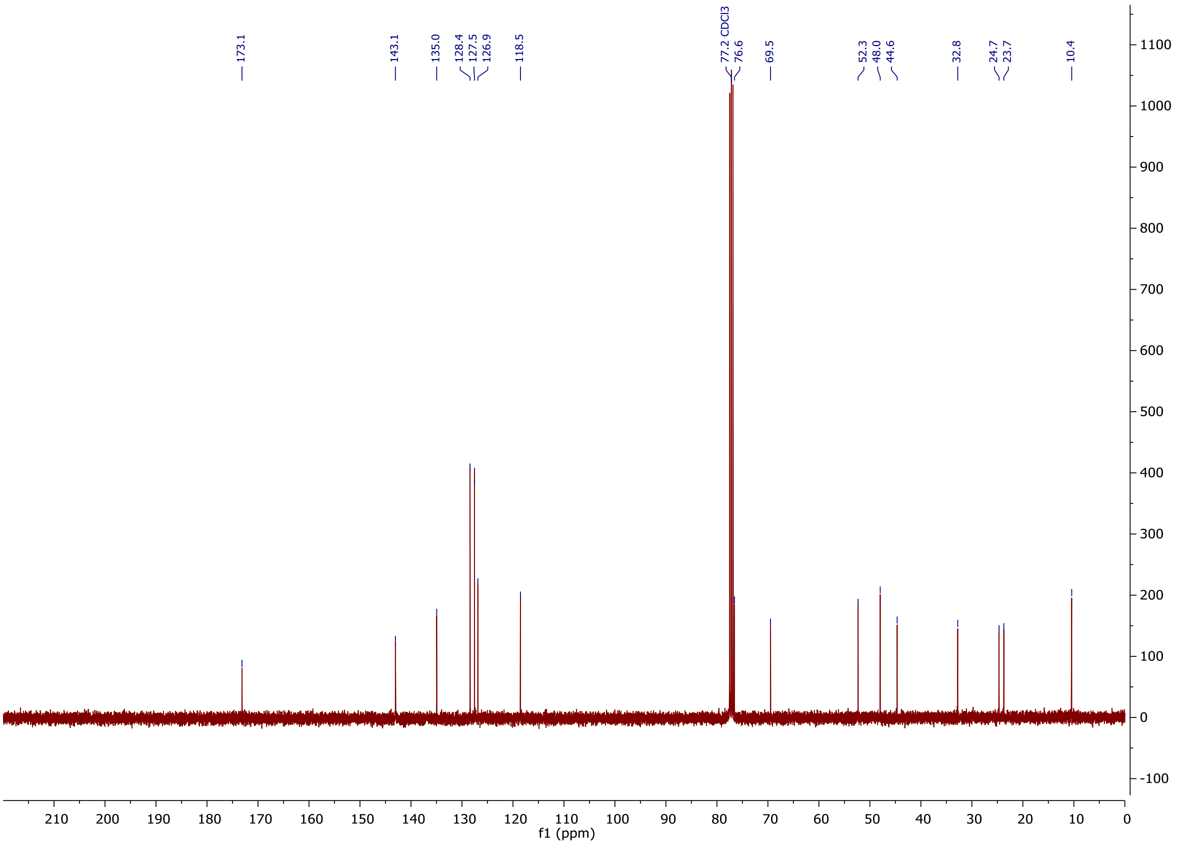

(*E*)-Ethyl 4-(4-benzyl-3-ethyl-5,5-dimethyl-2-oxomorpholin-3-yl)but-2-enoate

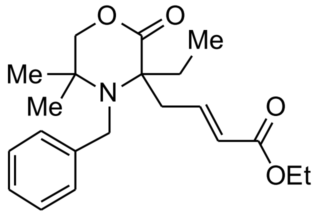

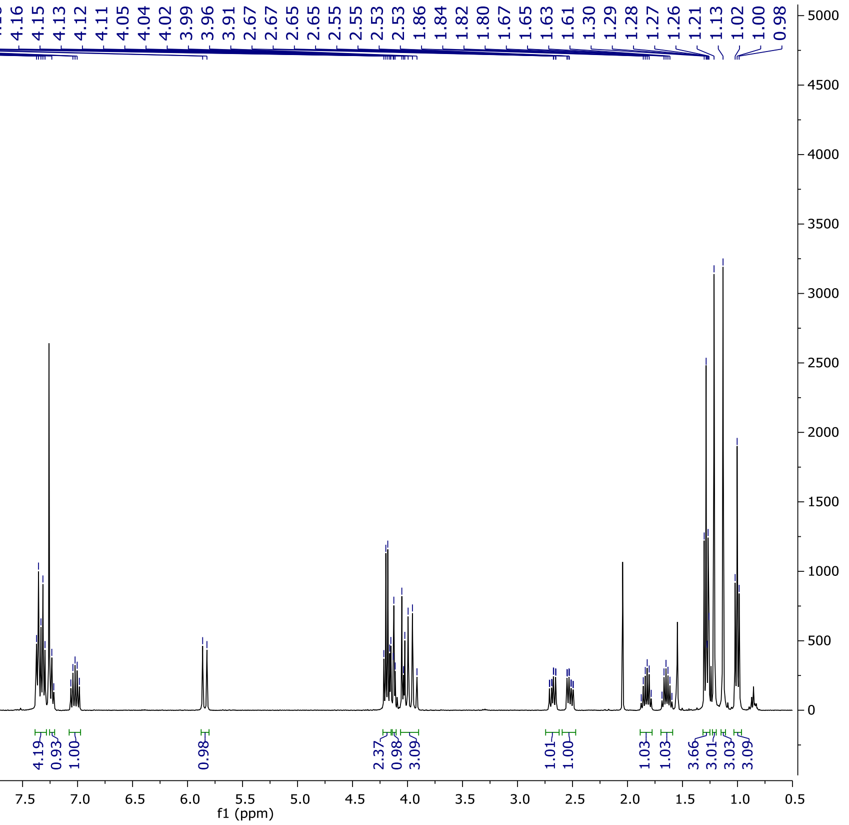

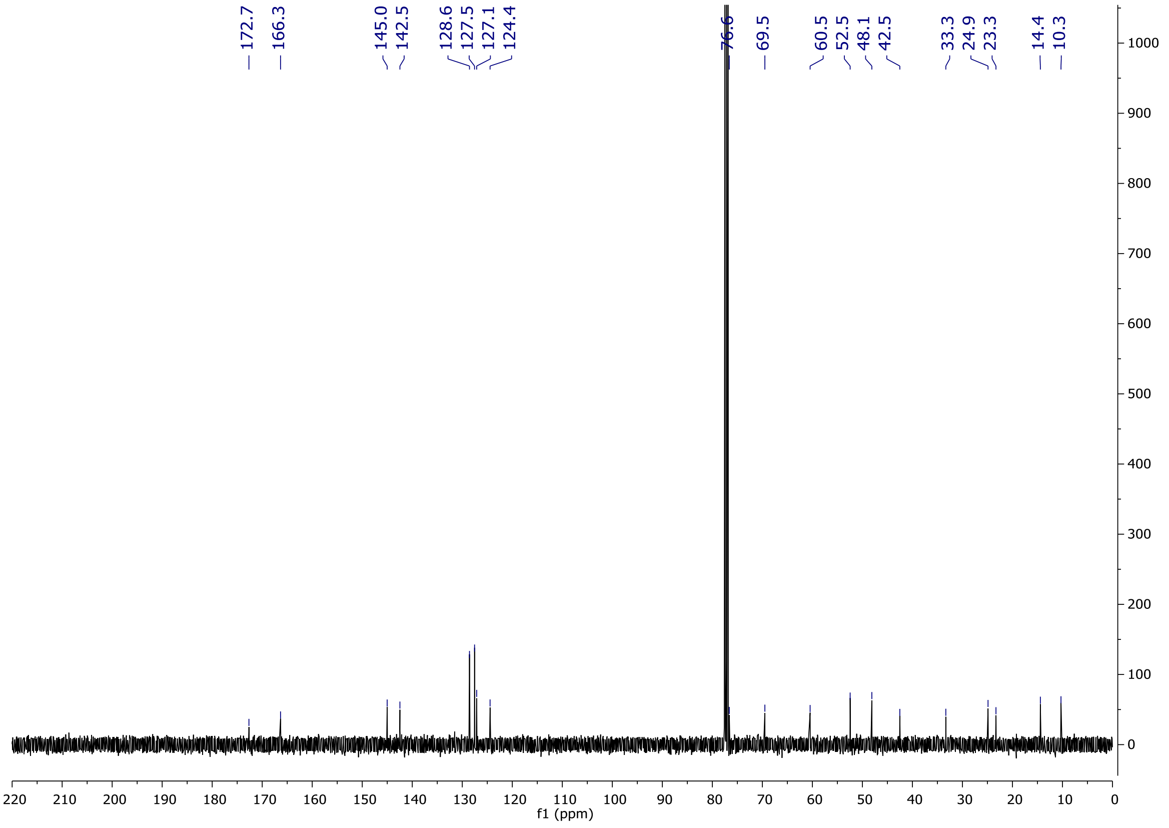

Ethyl 4-(3-ethyl-5,5-dimethyl-2-oxomorpholin-3-yl)butanoate 1d

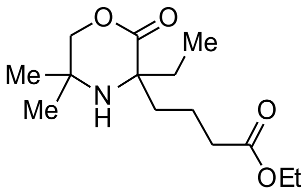

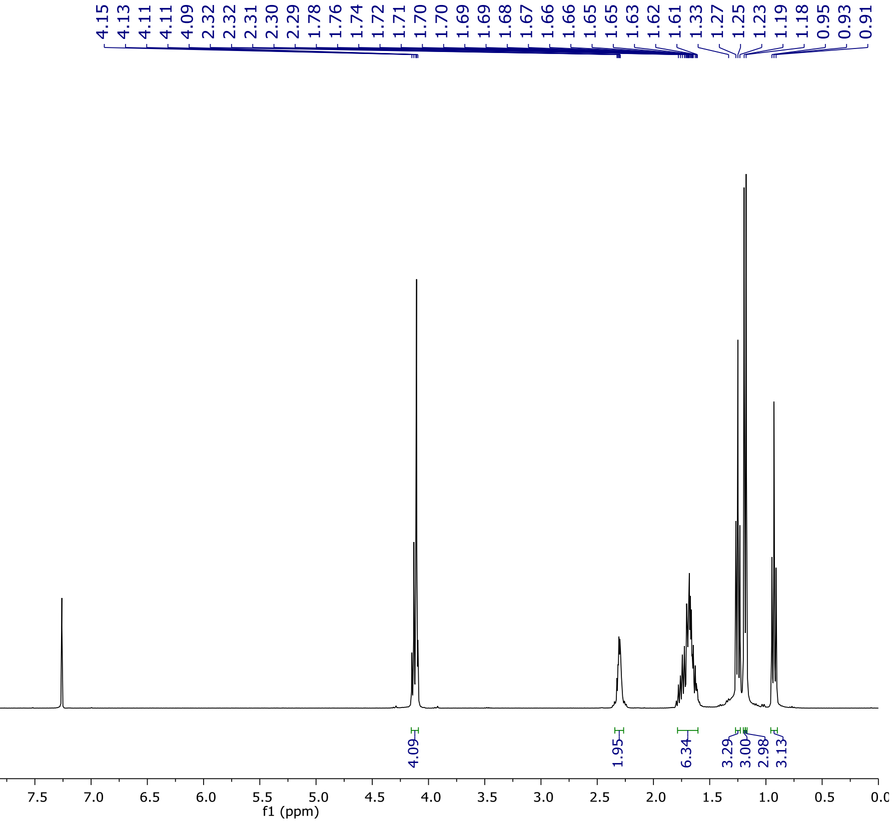

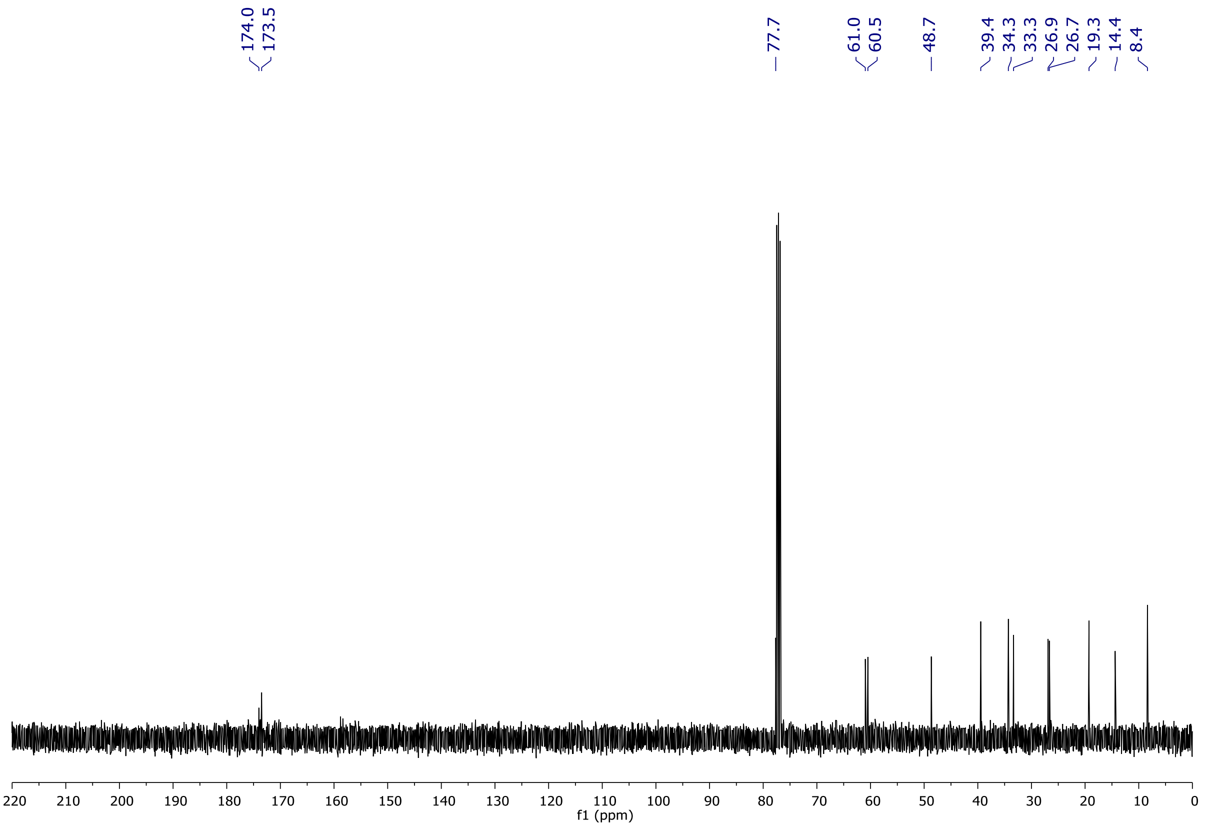

tert-Butyl 5,5-dimethyl-2-oxomorpholine-4-carboxylate

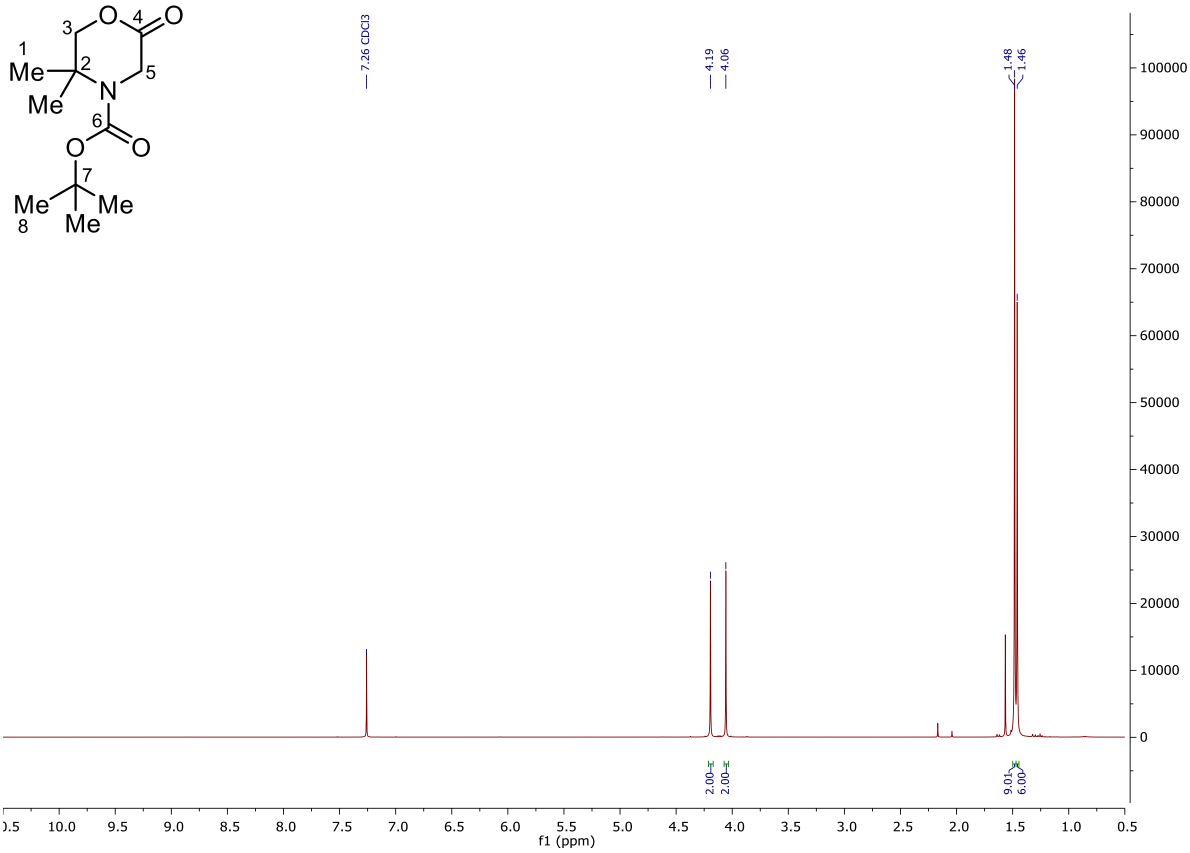

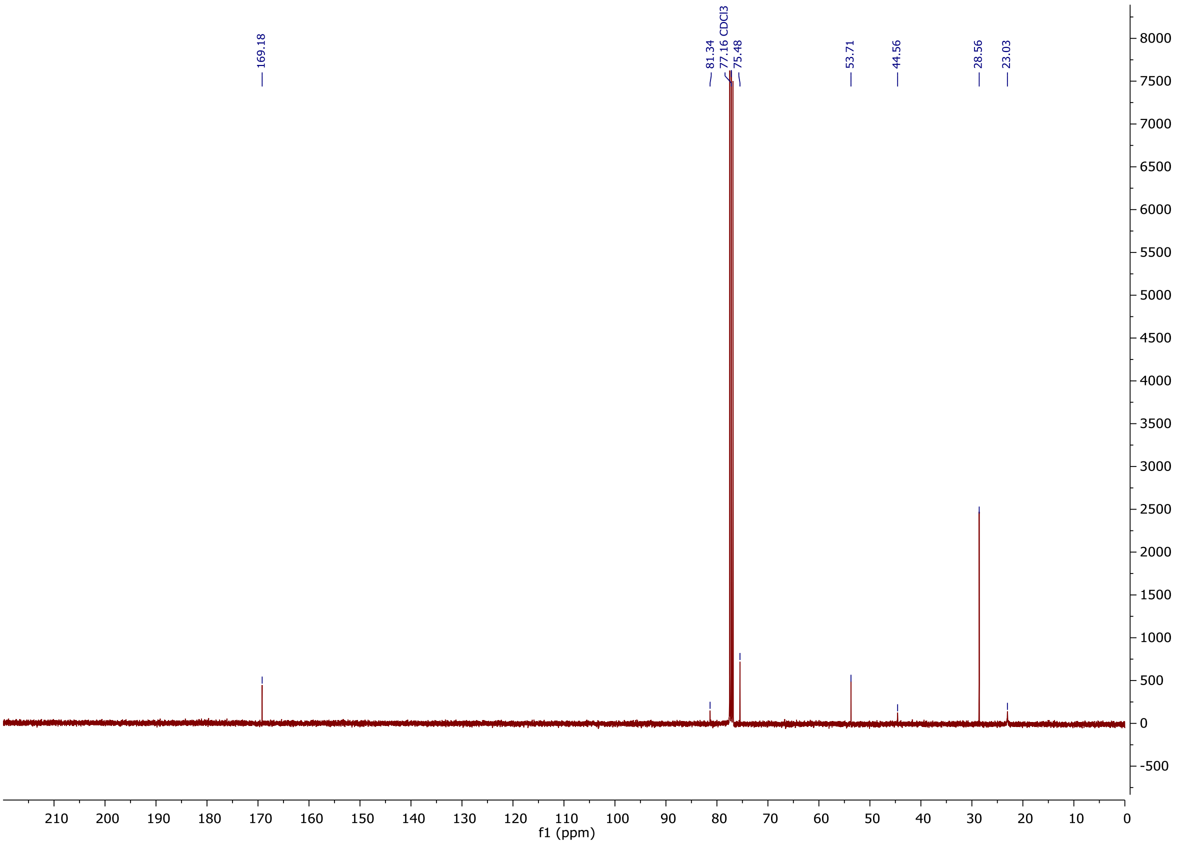

Some carbon signals have broadened due to the coupling to nitrogen giving intermediate relaxation .

tert-Butyl 3-ethyl-5,5-dimethyl-2-oxomorpholine-4-carboxylate

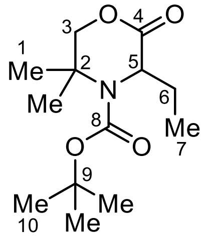

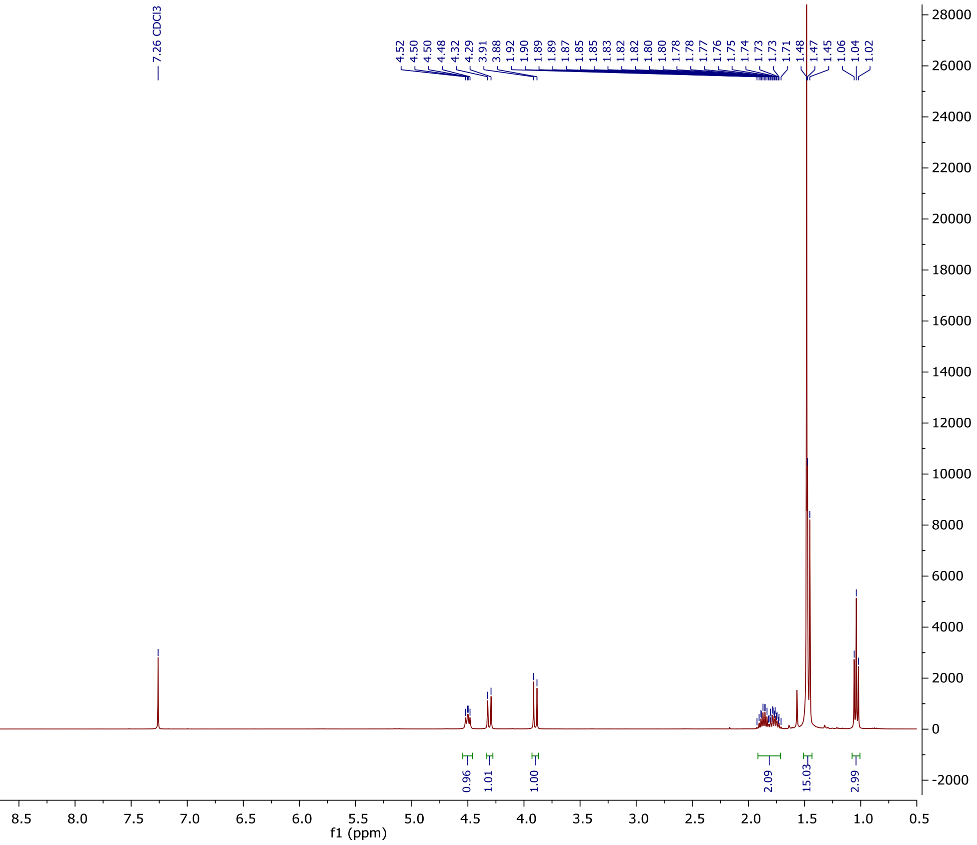

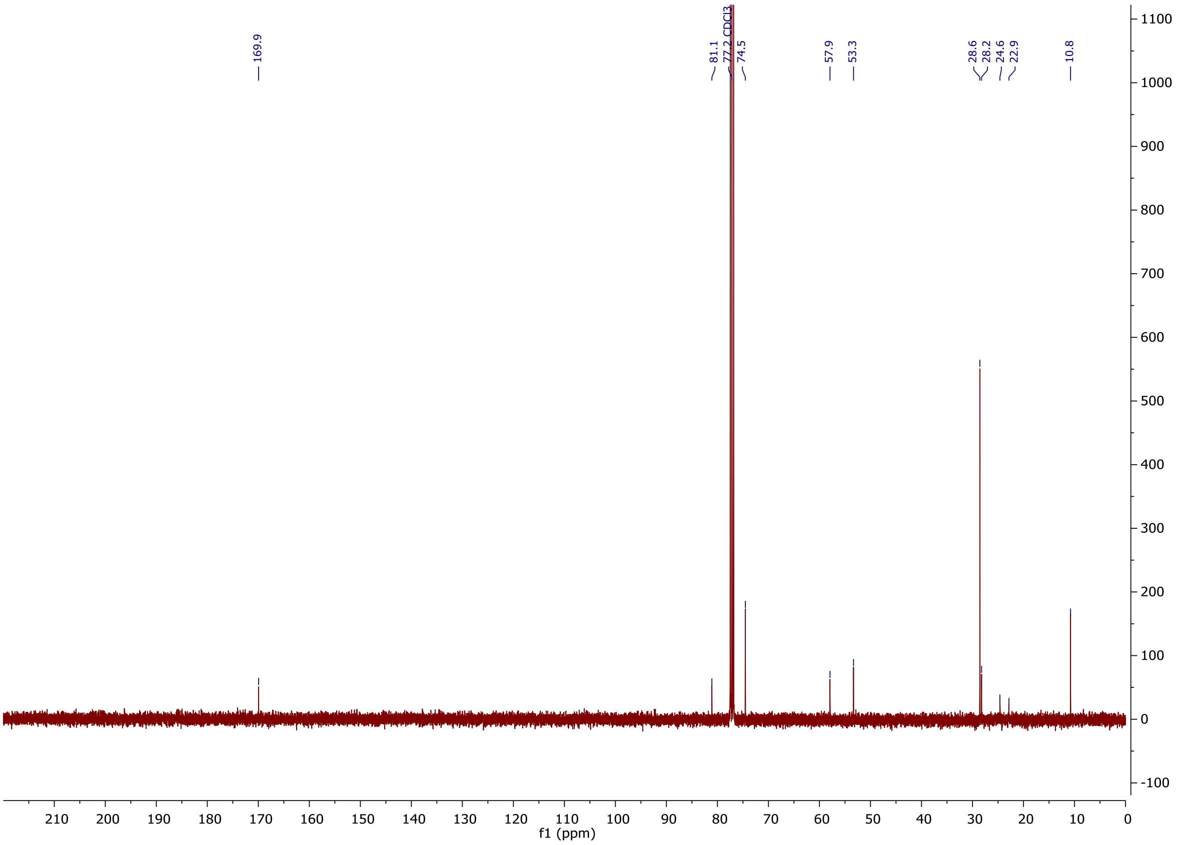

Some carbon signals have broadened due to the coupling to nitrogen giving intermediate relaxation .

***tert*-Butyl 3-allyl-3-ethyl-5,5-dimethyl-2-oxomorpholine-4-carboxylate**

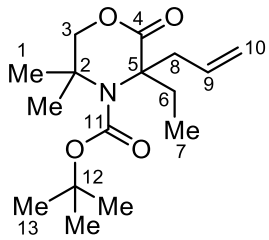

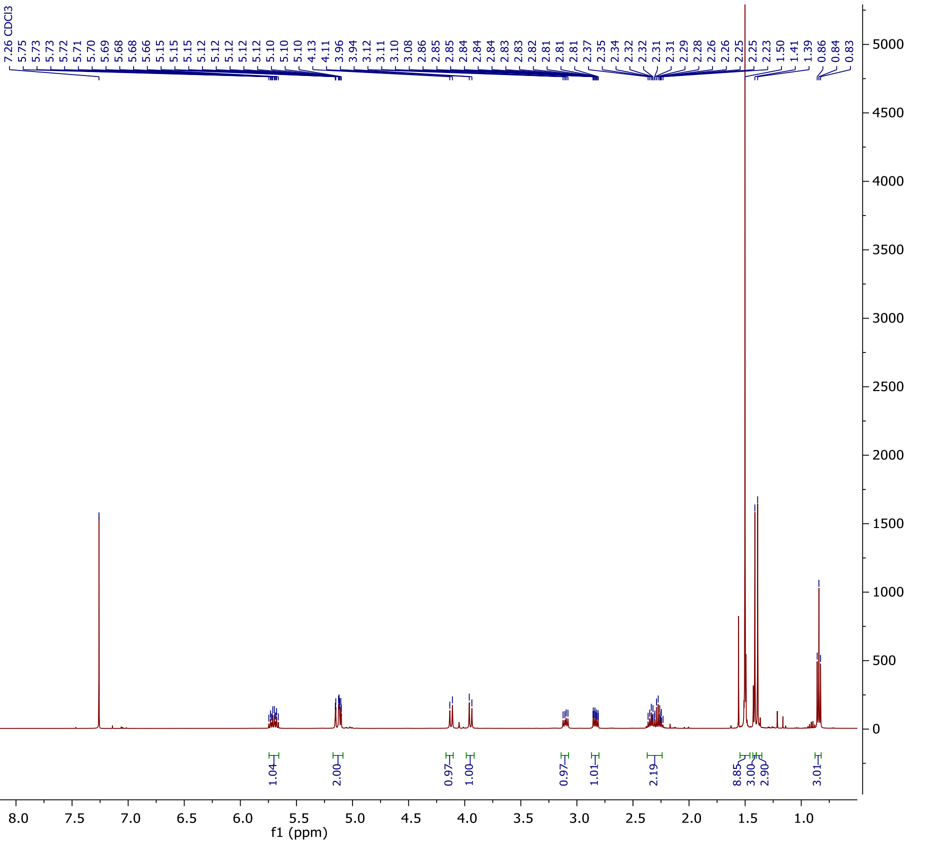

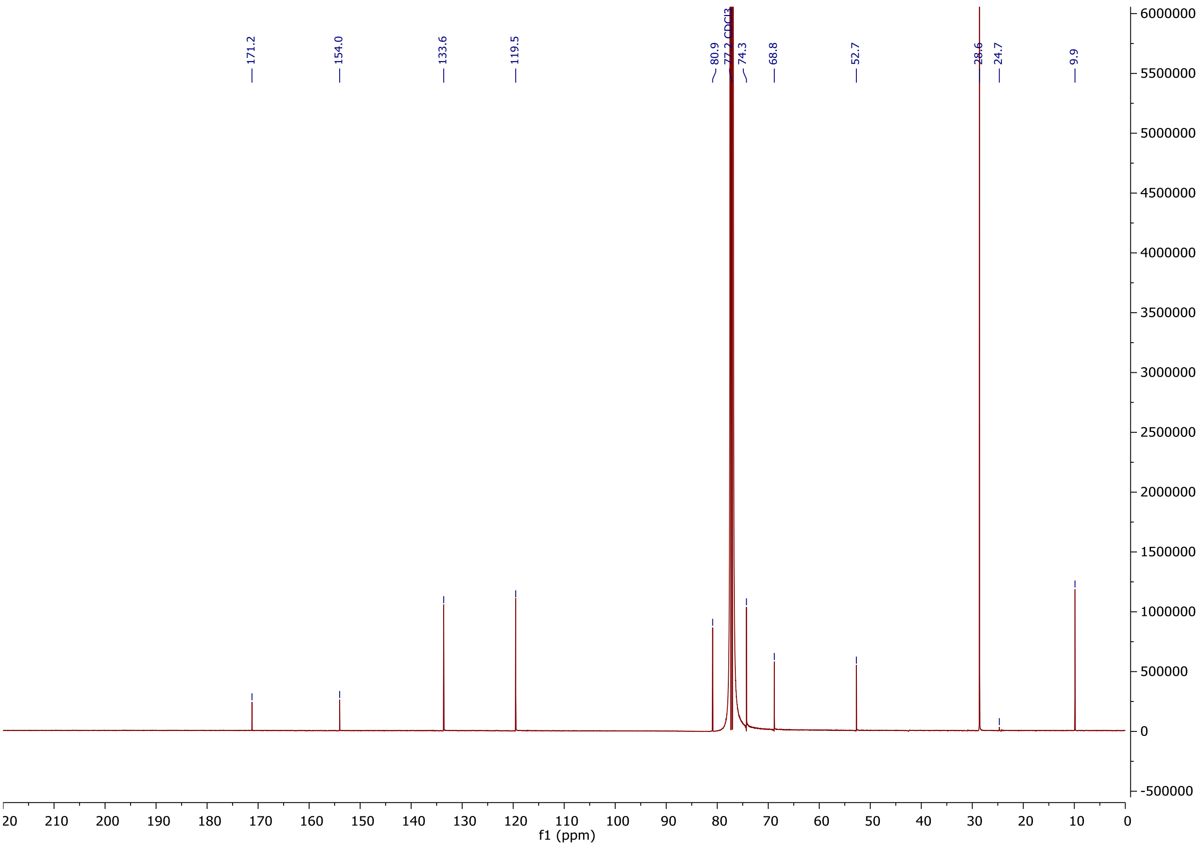

Some carbon signals have broadened due to the coupling to nitrogen giving intermediate relaxation .

***tert*-Butyl 3-(3-cyanoallyl)-3-ethyl-5,5-dimethyl-2-oxomorpholine-4-carboxylate**

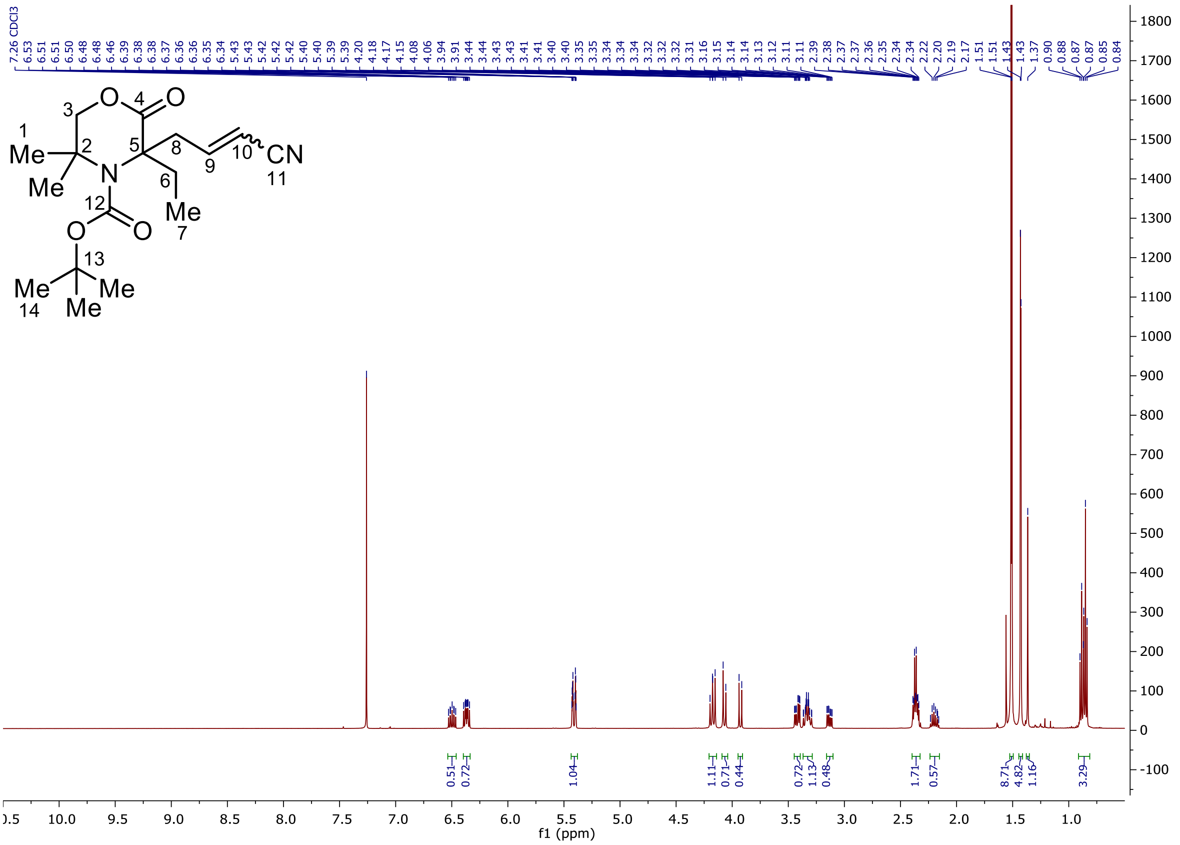

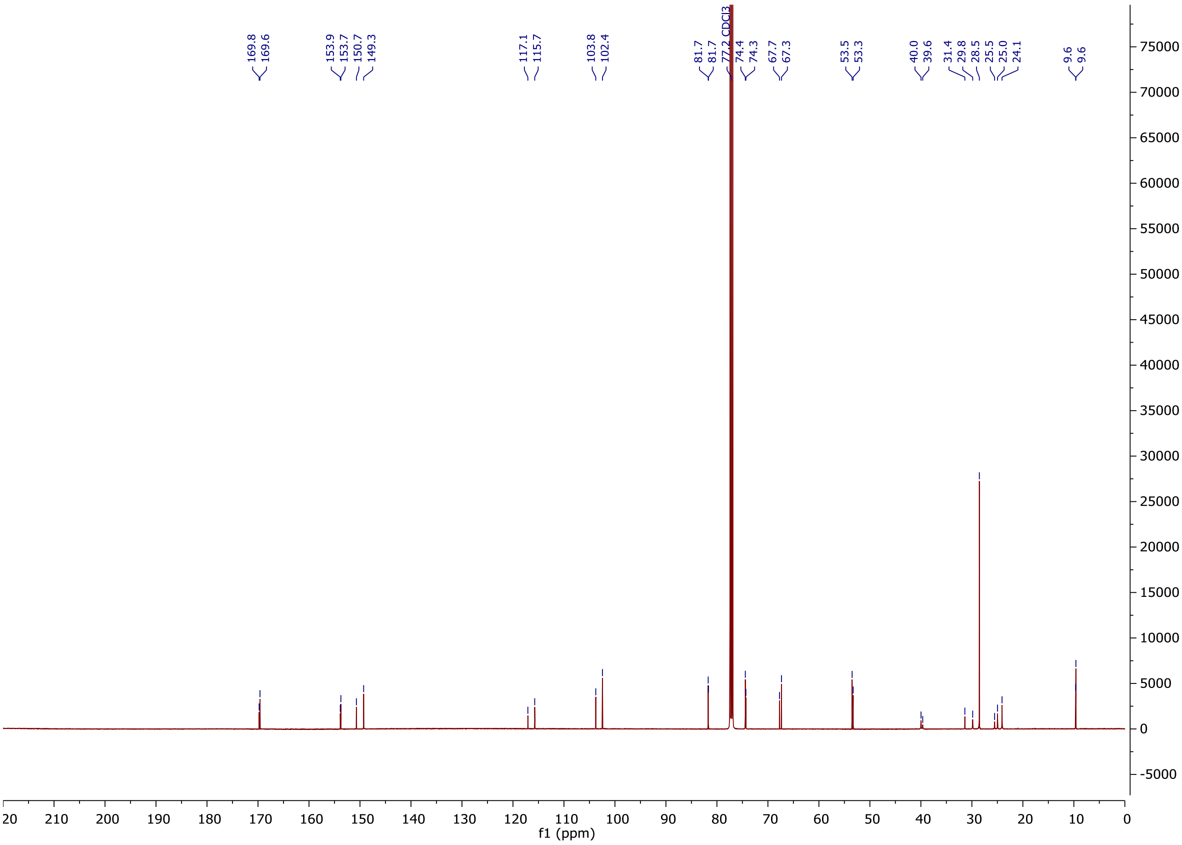

4-(3-Ethyl-5,5-dimethyl-2-oxomorpholin-3-yl)butanenitrile 1f

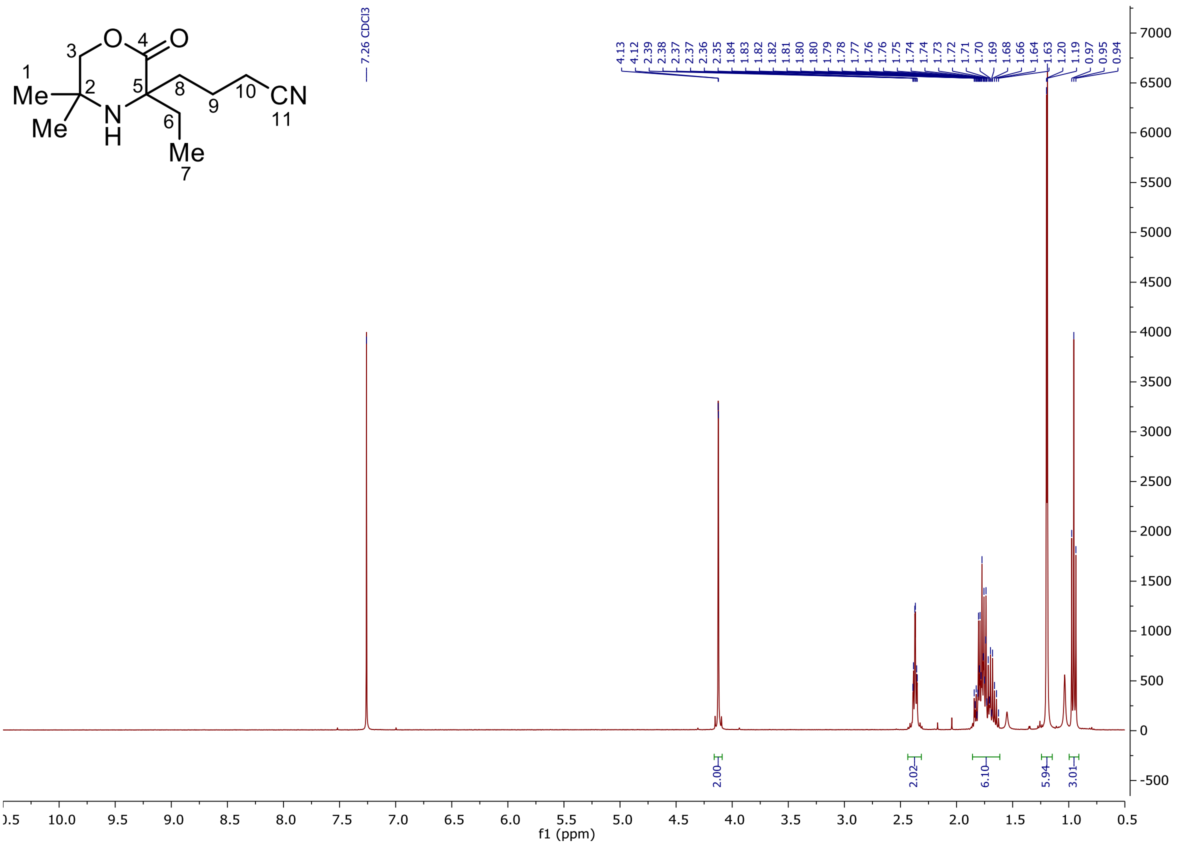

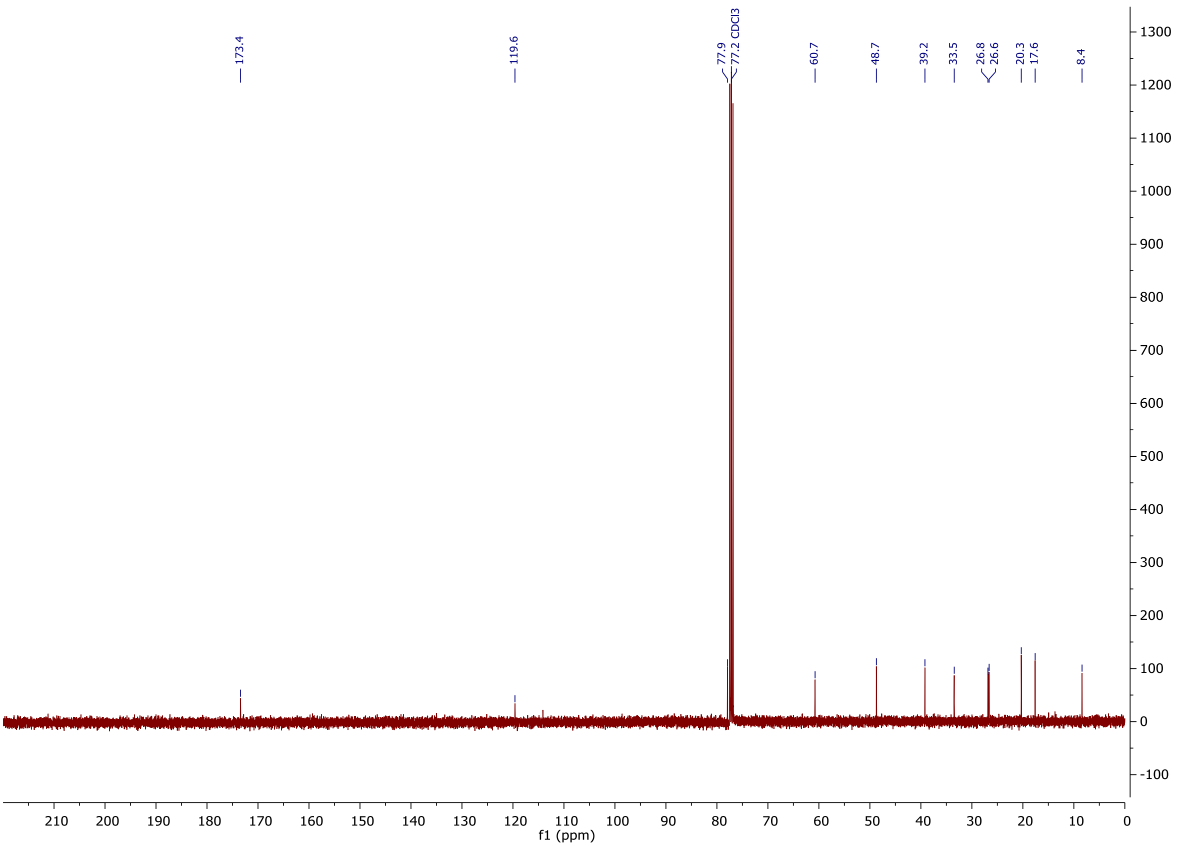

***tert*-Butyl 3-ethyl-5,5-dimethyl-2-oxo-3-(3-(phenylsulfonyl)propyl)morpholine-4-carboxylate**

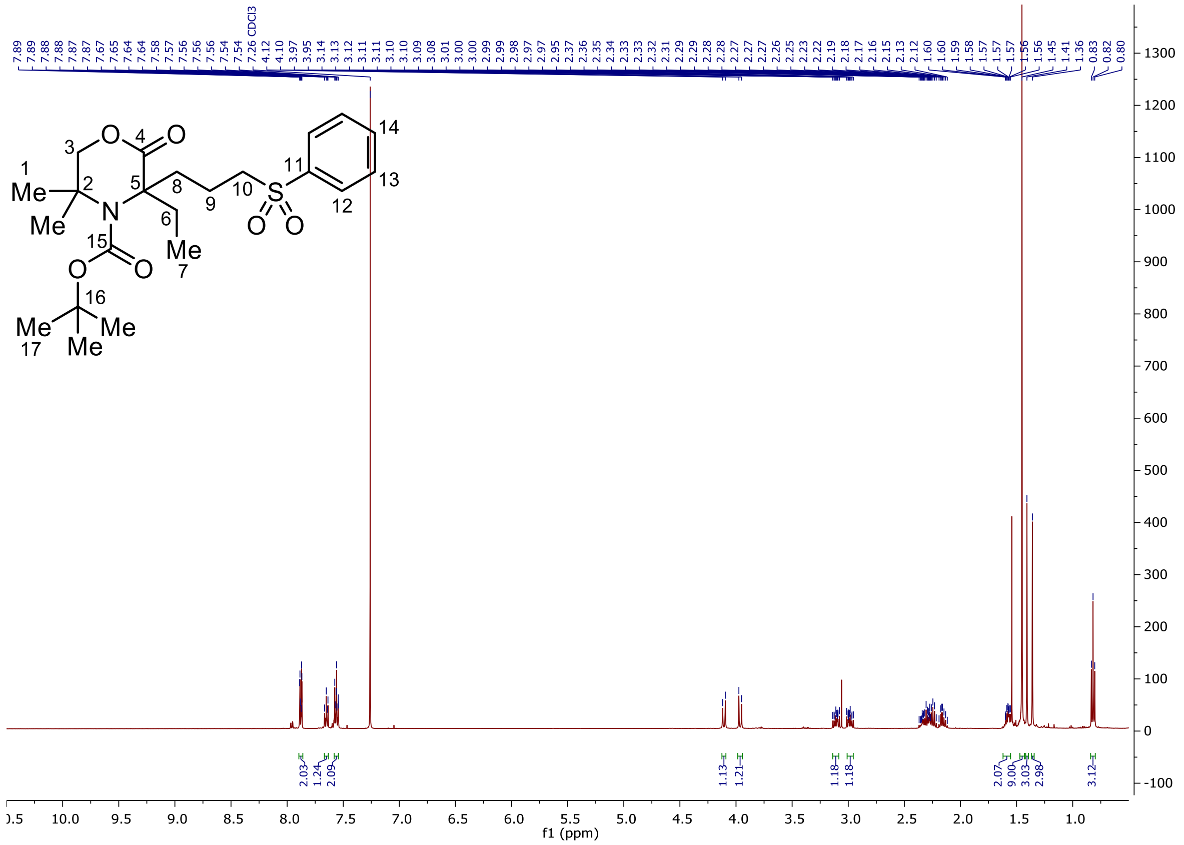

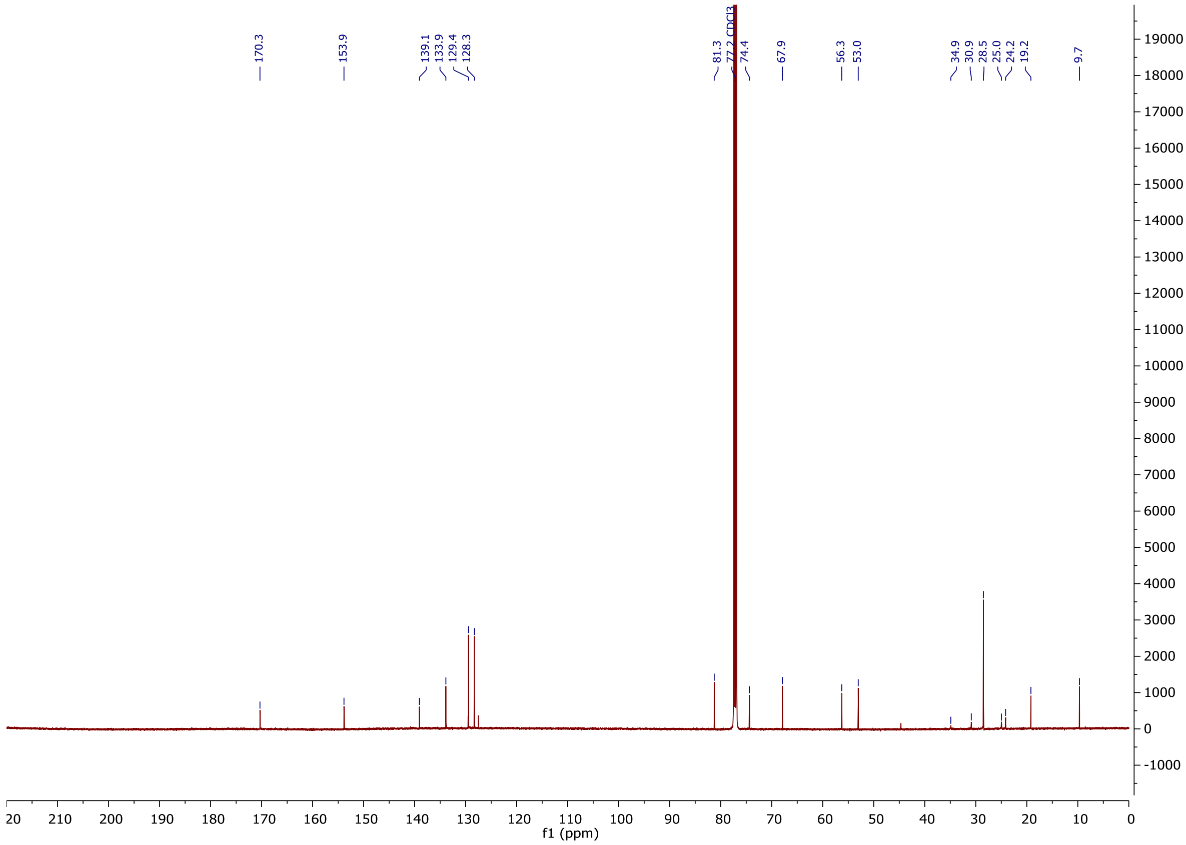

3-Ethyl-5,5-dimethyl-3-(3-(phenylsulfonyl)propyl)morpholin-2-one 1g

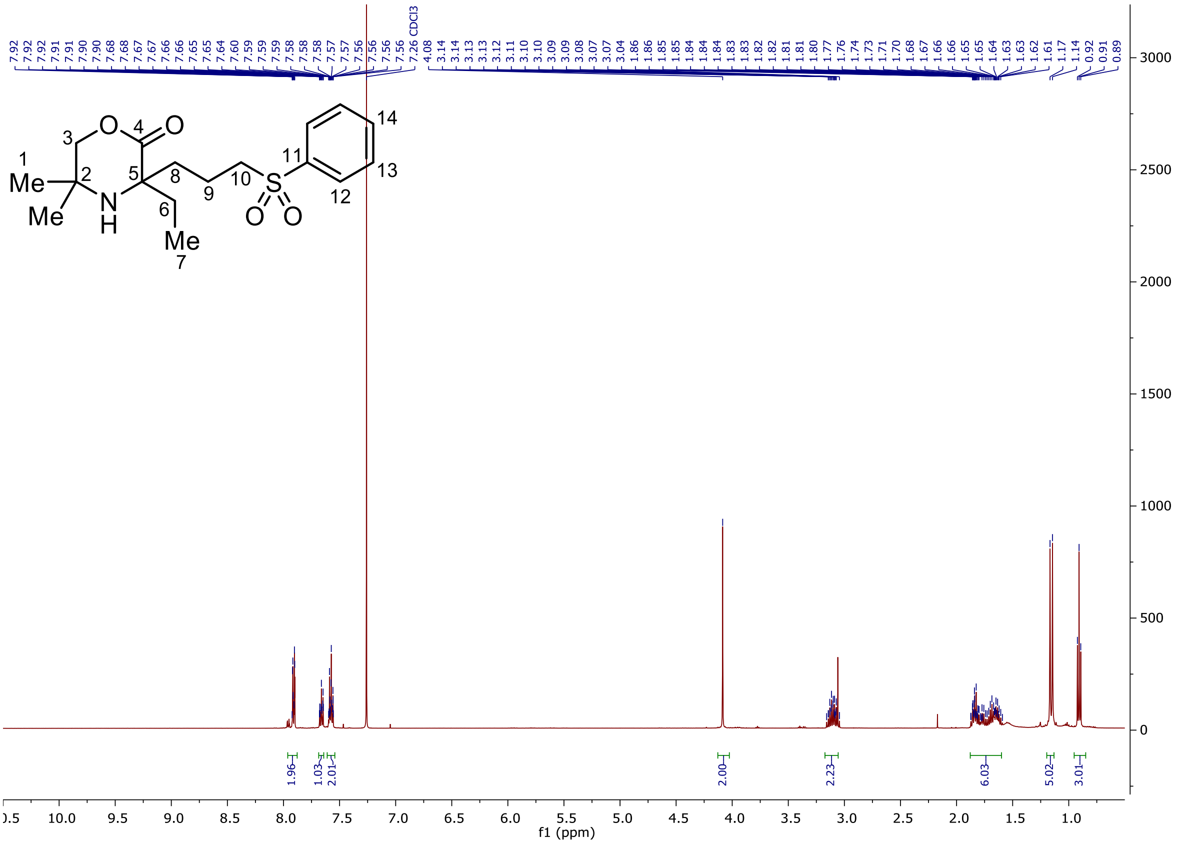

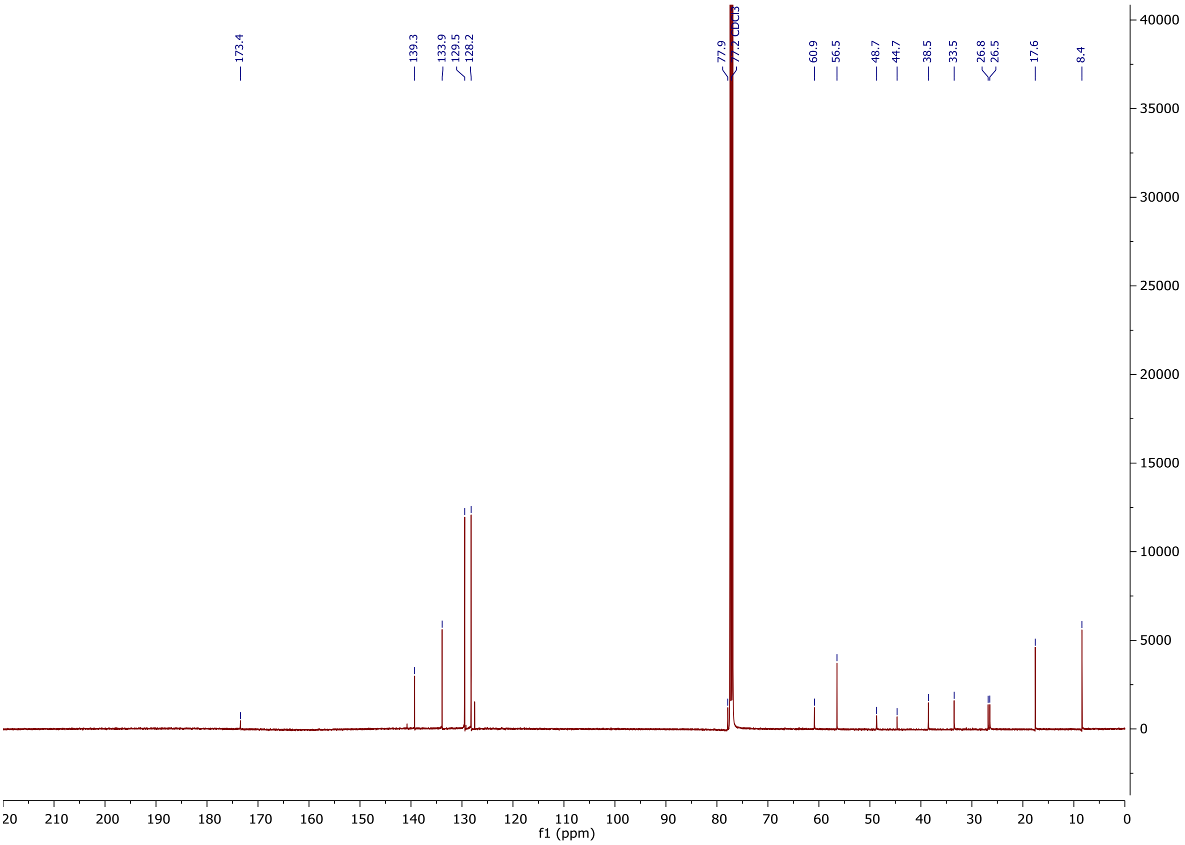

2-(3-(4-Benzyl-5,5-dimethyl-2-oxomorpholin-3-yl)propyl)isoindoline-1,3-dione

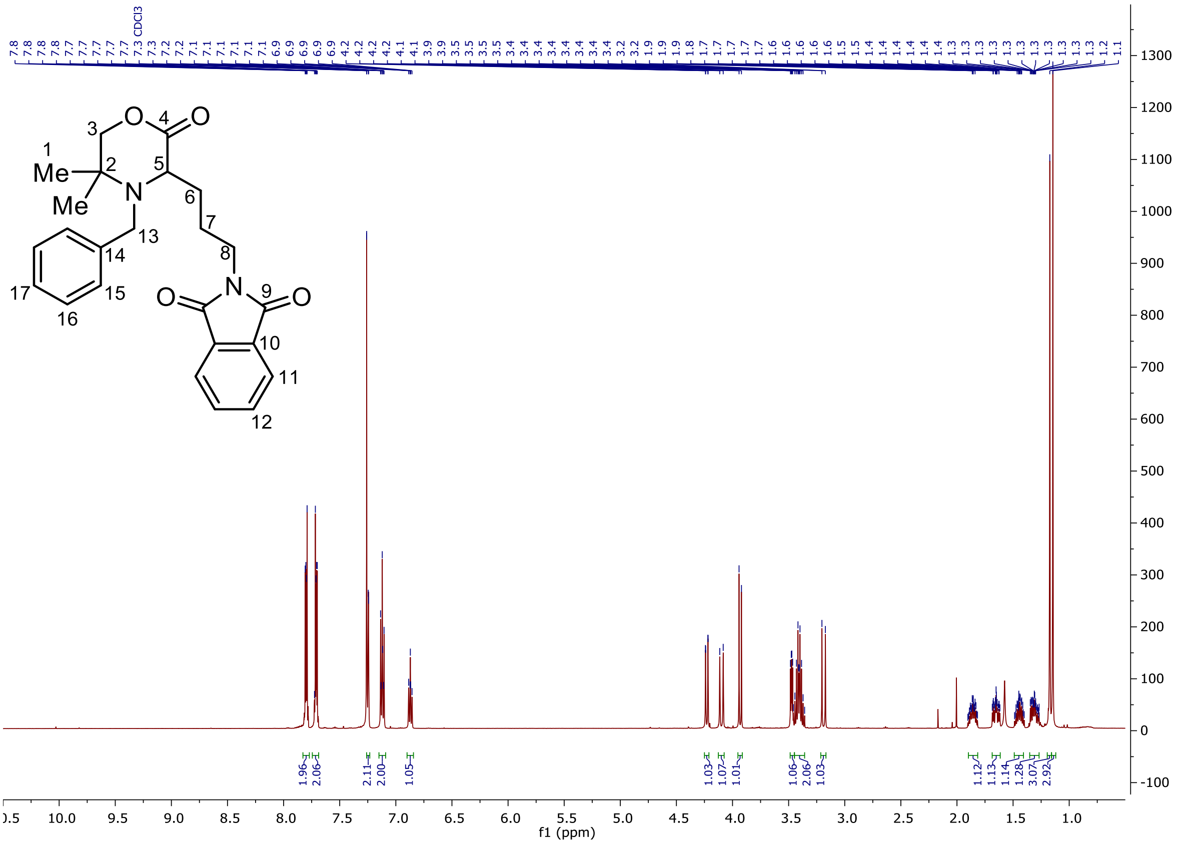

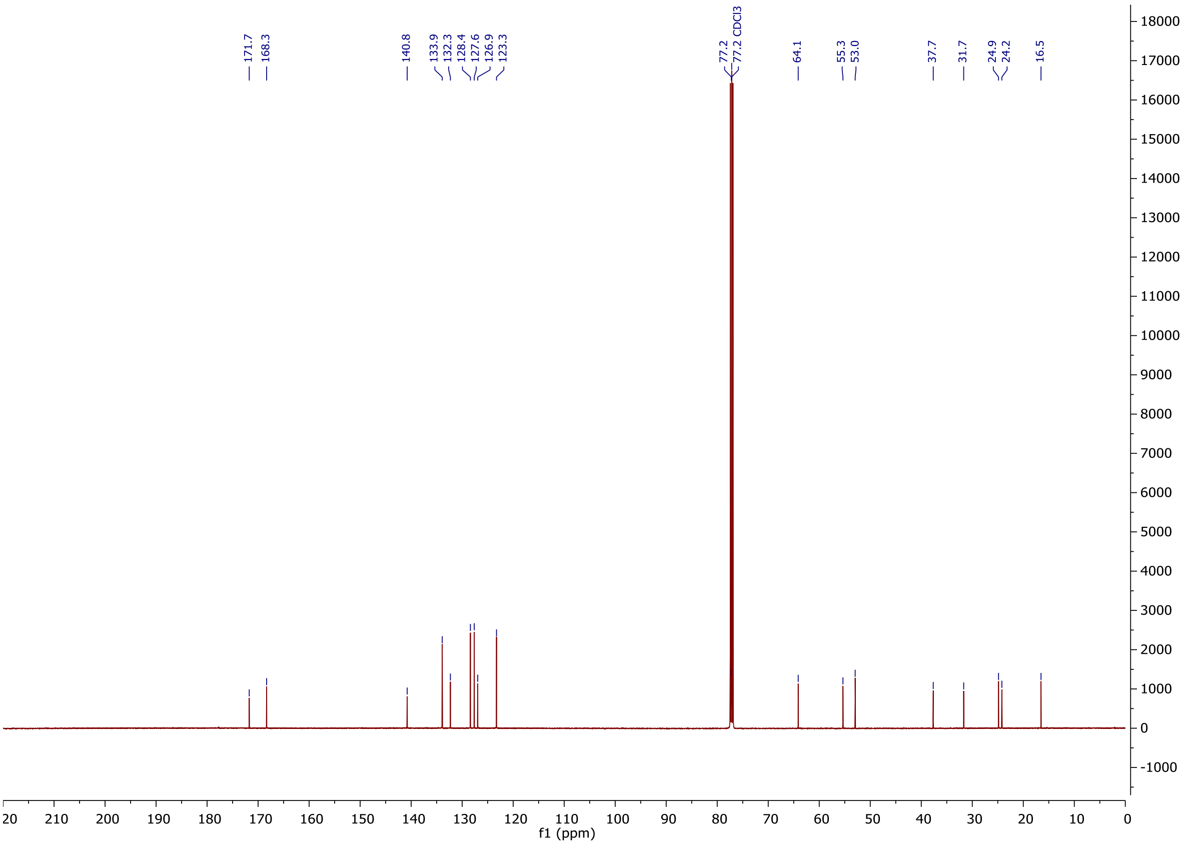

2-(3-(4-Benzyl-3-ethyl-5,5-dimethyl-2-oxomorpholin-3-yl)propyl)isoindoline-1,3-dione

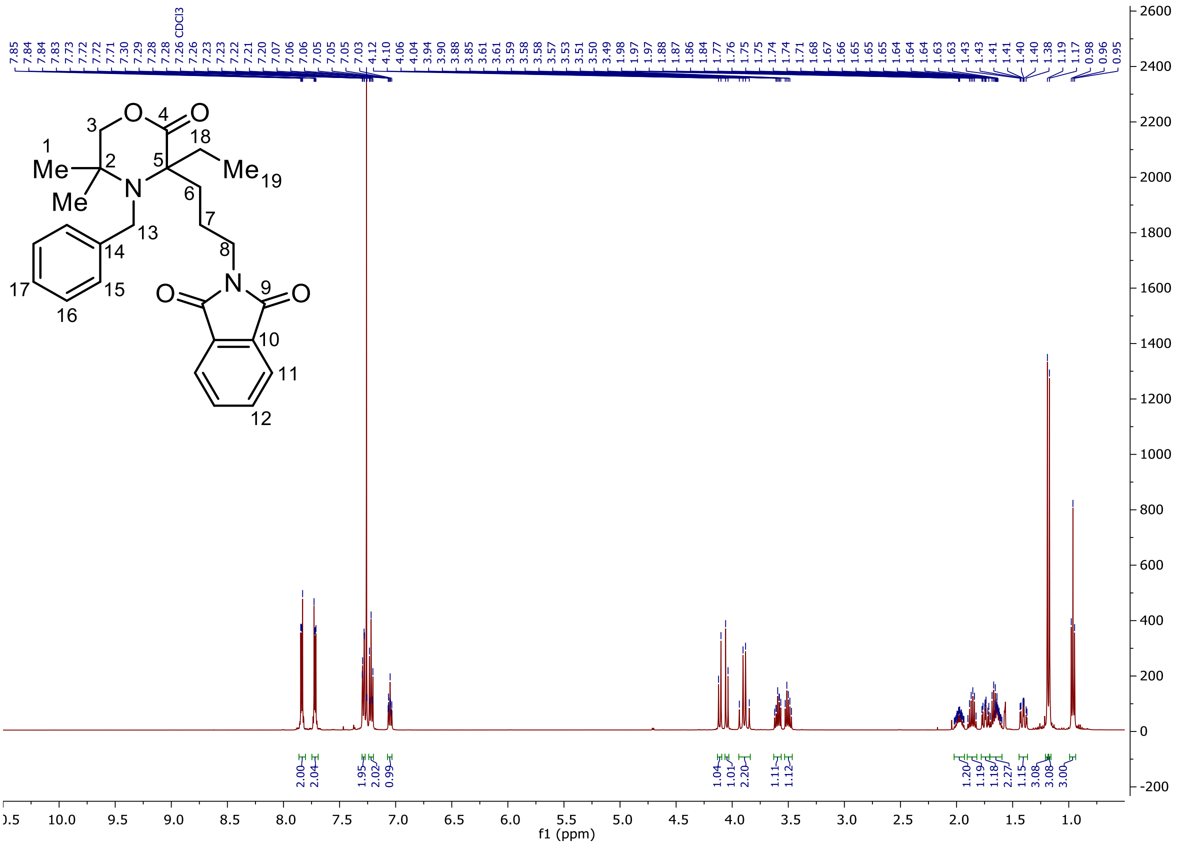

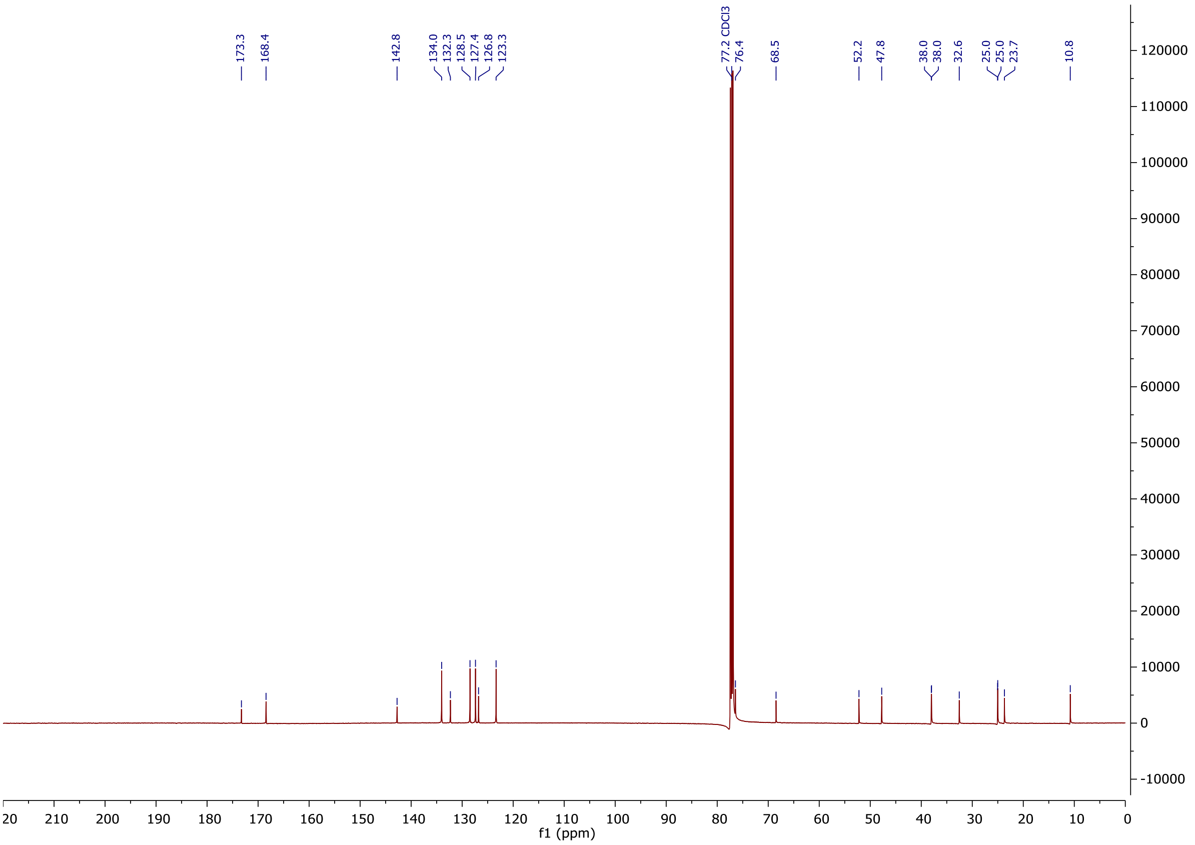

2-(3-(3-Ethyl-5,5-dimethyl-2-oxomorpholin-3-yl)propyl)isoindoline-1,3-dione 1h

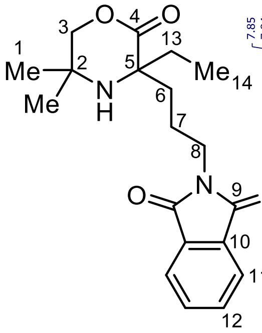

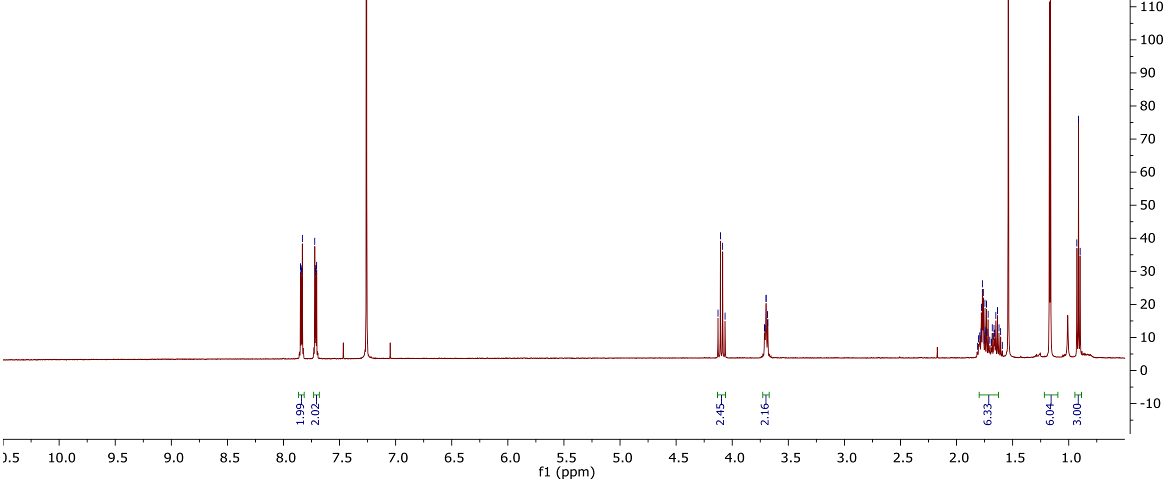

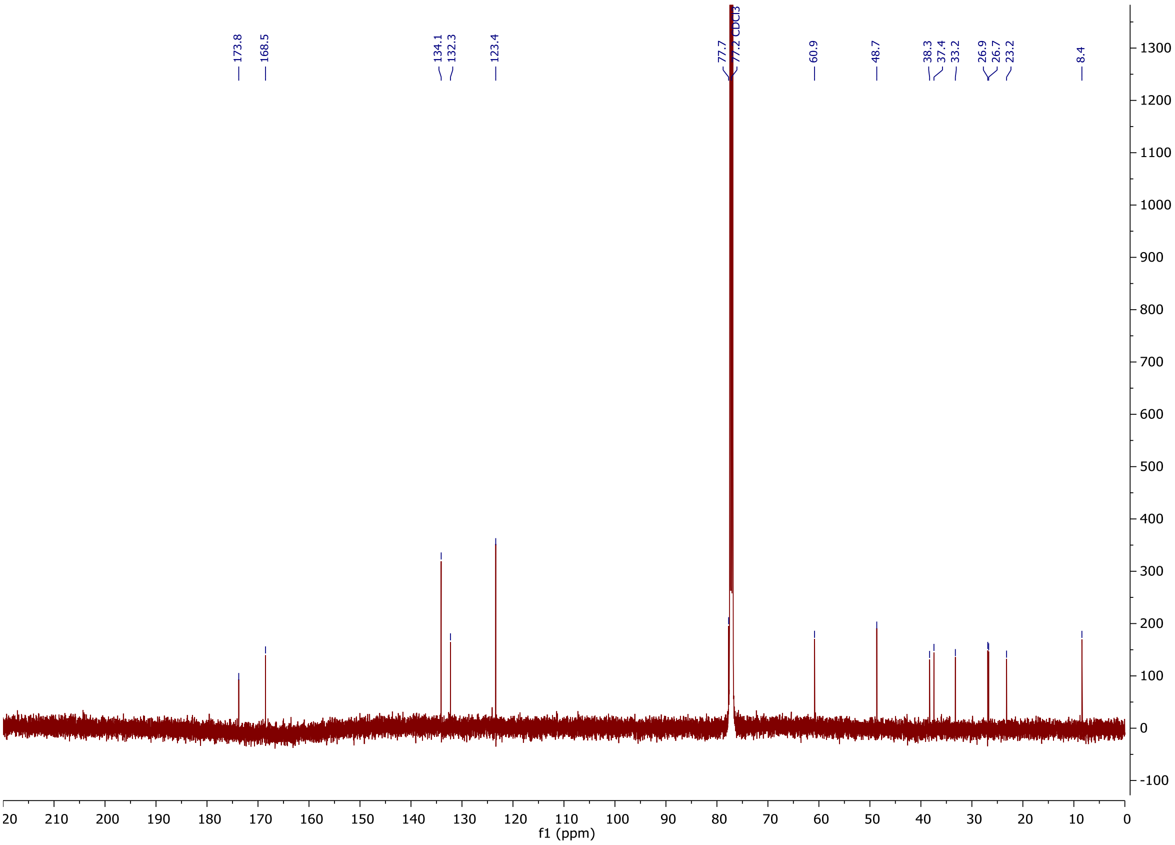

3-Ethyl-3,5,5-trimethylmorpholin-2-one

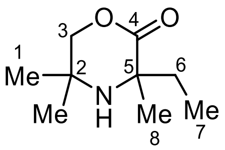

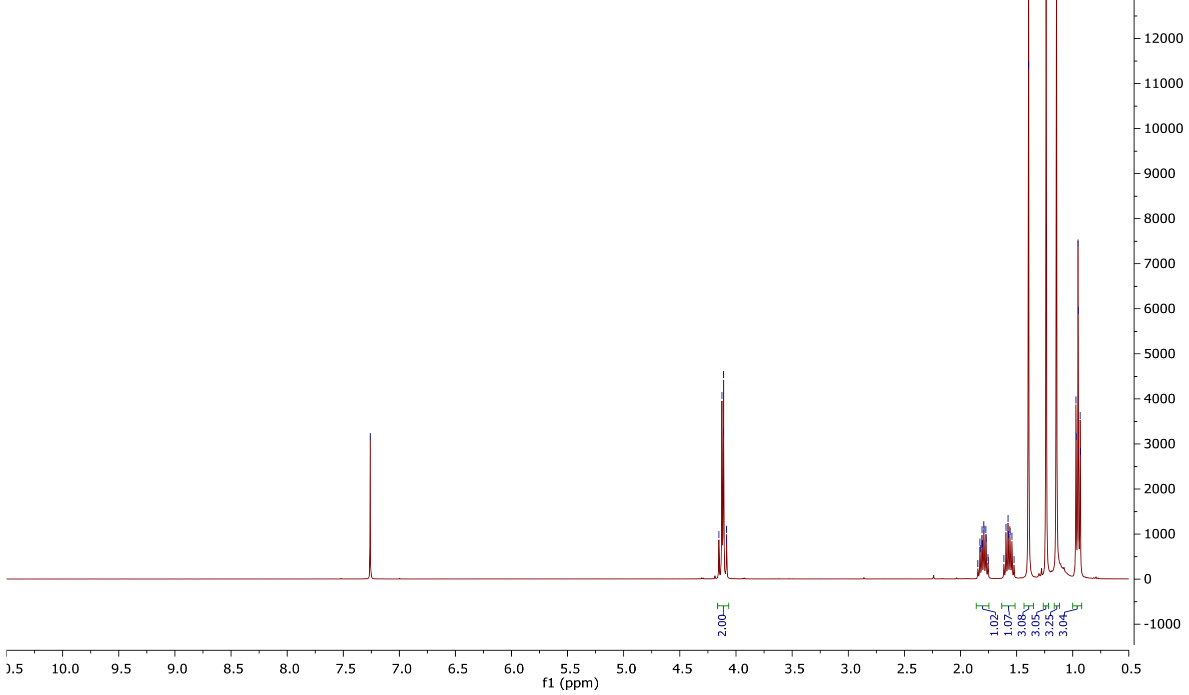

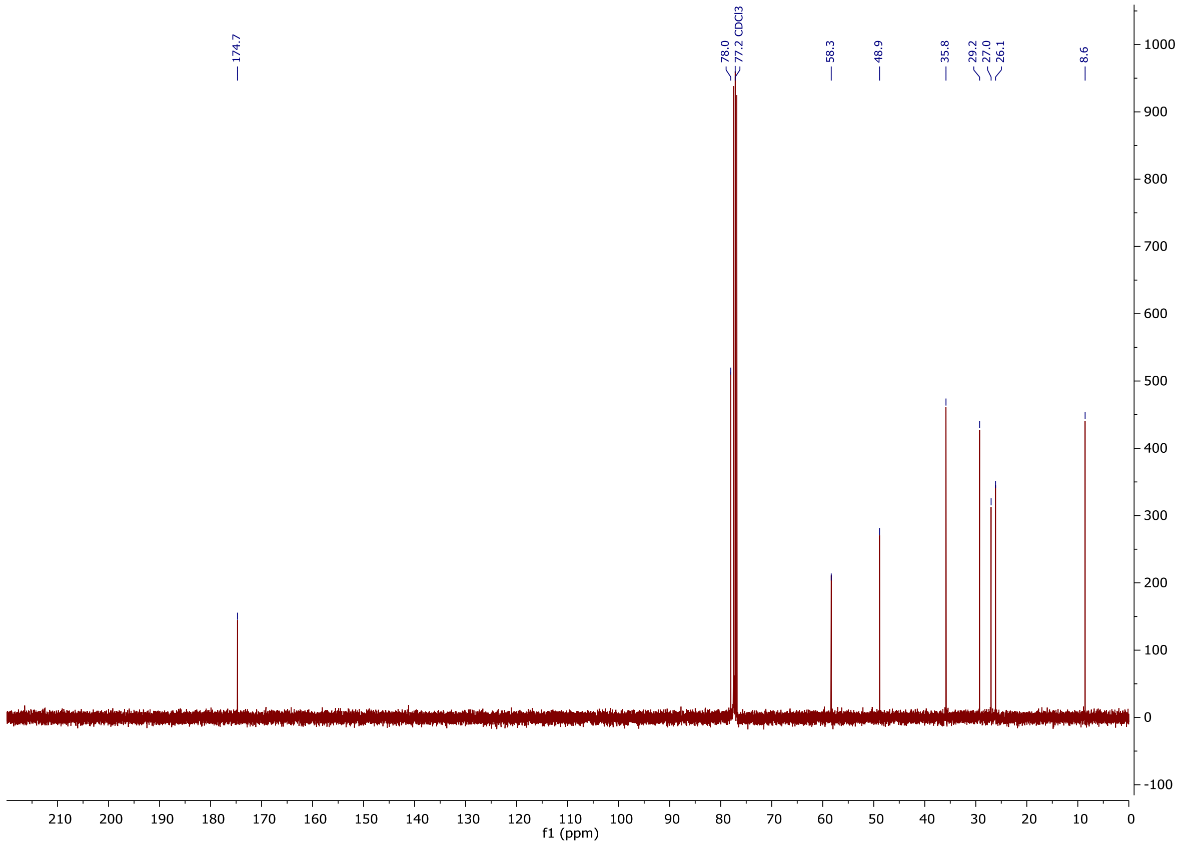

3-((Benzyloxy)methyl)-3-ethyl-5,5-dimethylmorpholin-2-one 1i

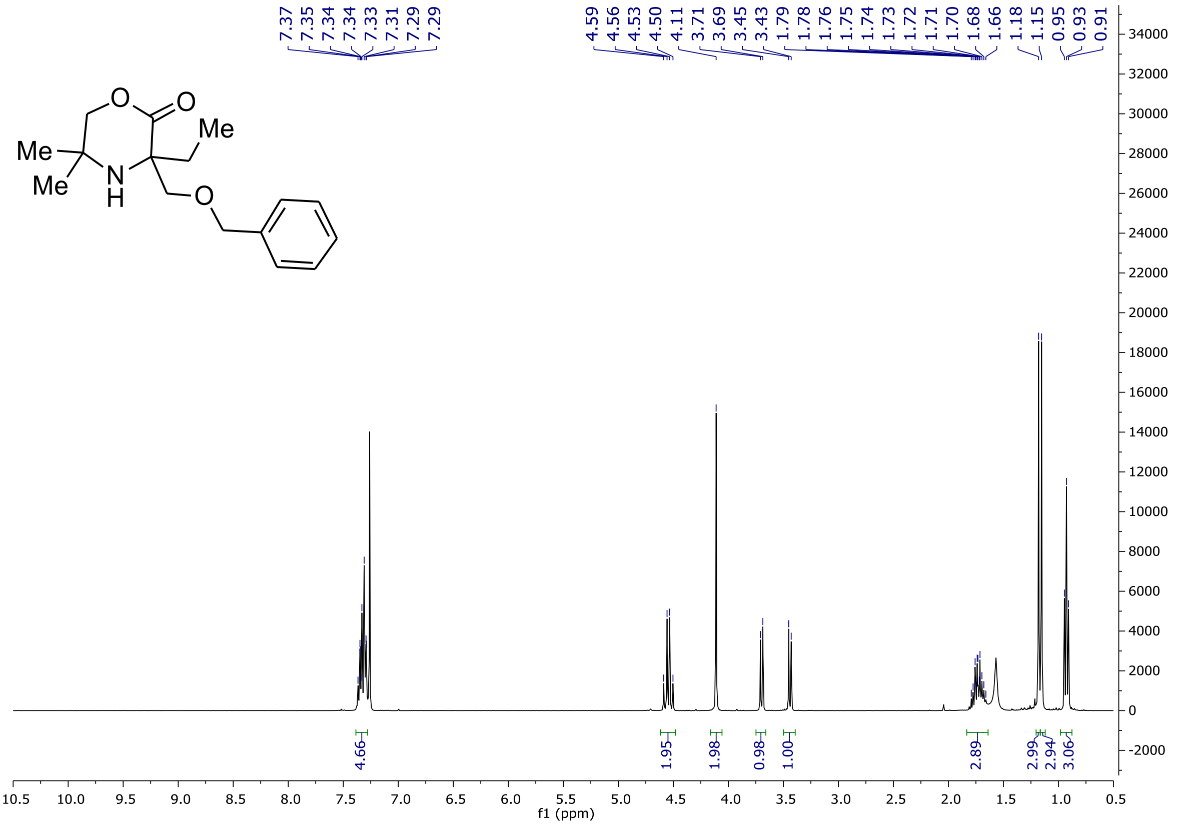

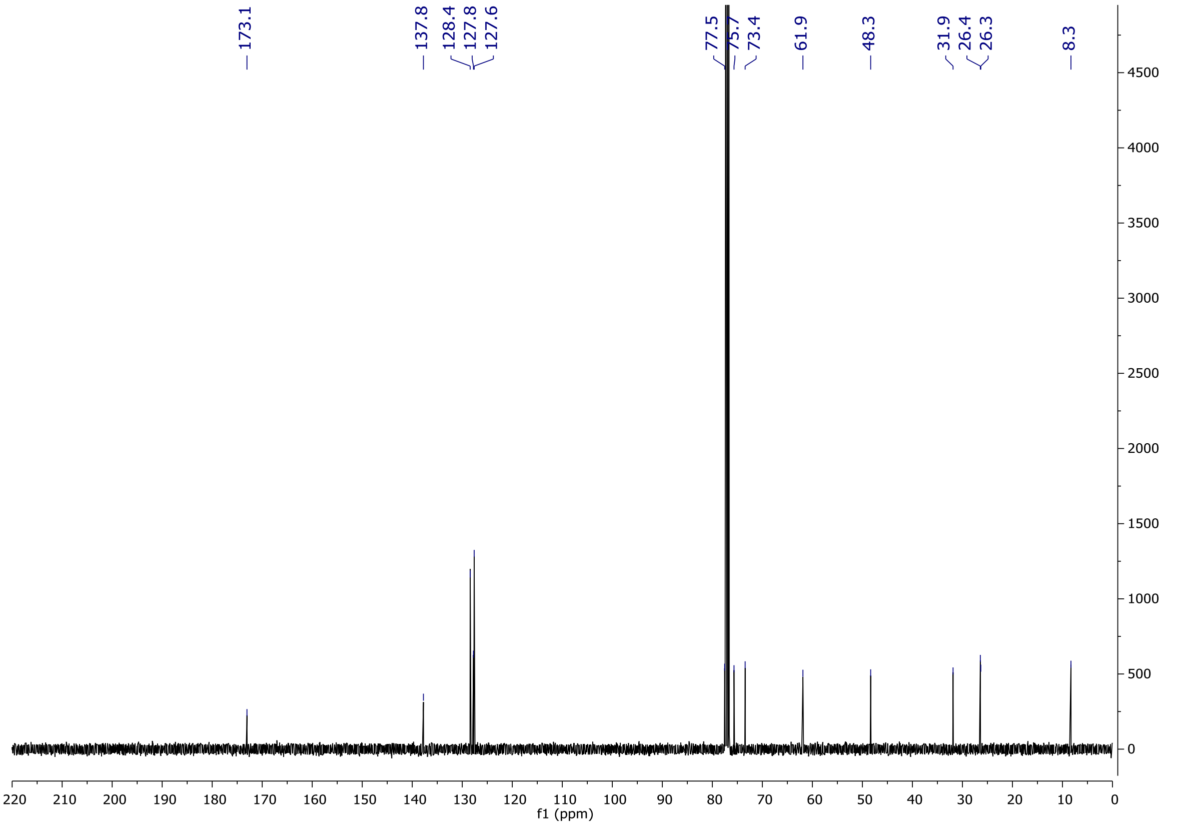

4-((2-Amino-2-methylpropyl)amino)benzonitrile

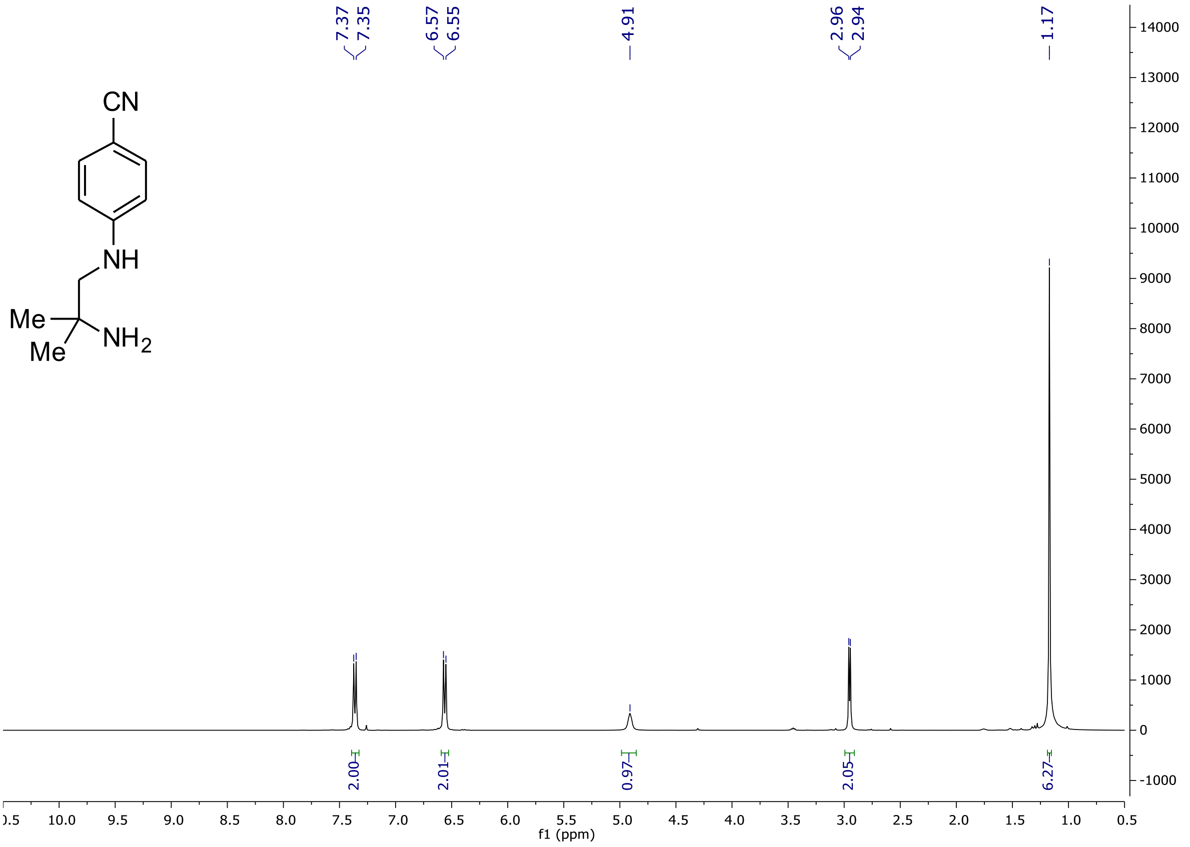

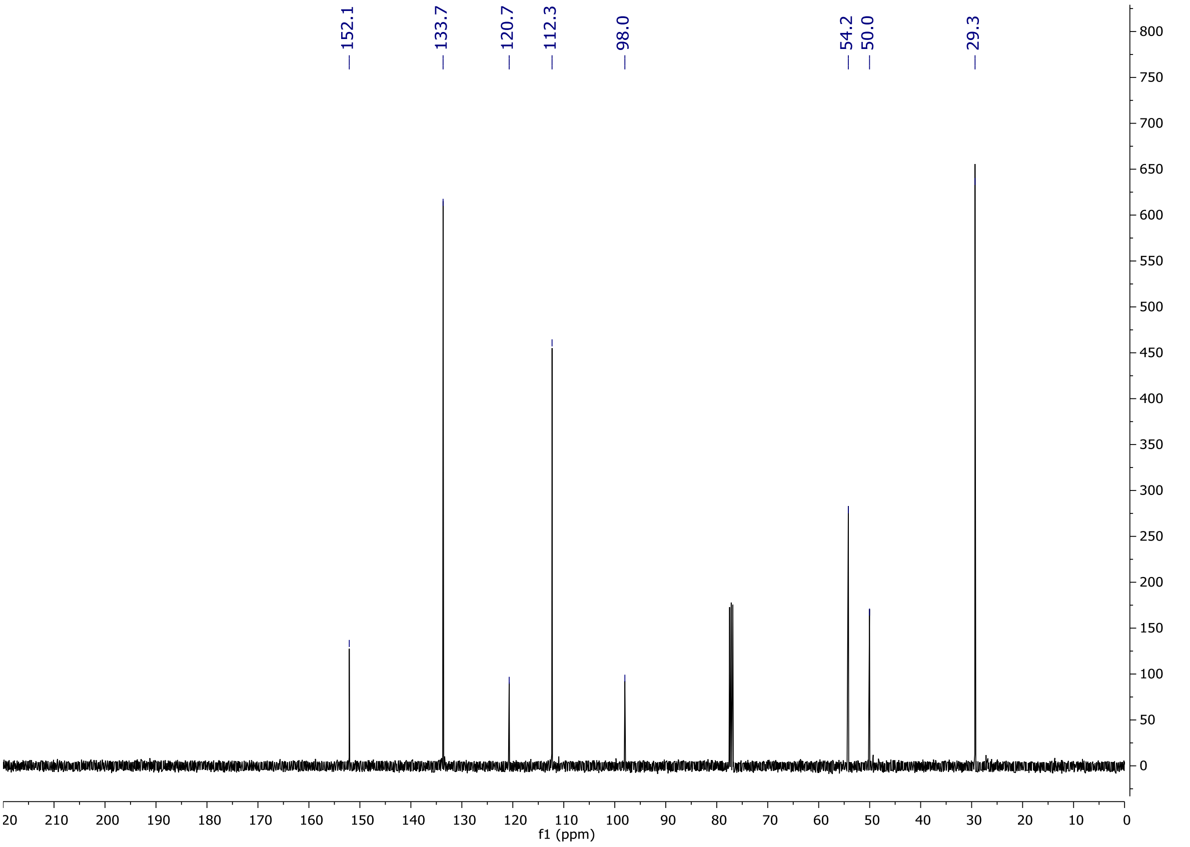

4-(3,3-diethyl-5,5-dimethyl-2-oxopiperazin-1-yl)benzonitrile 1j

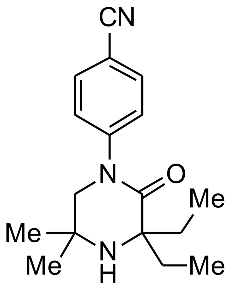

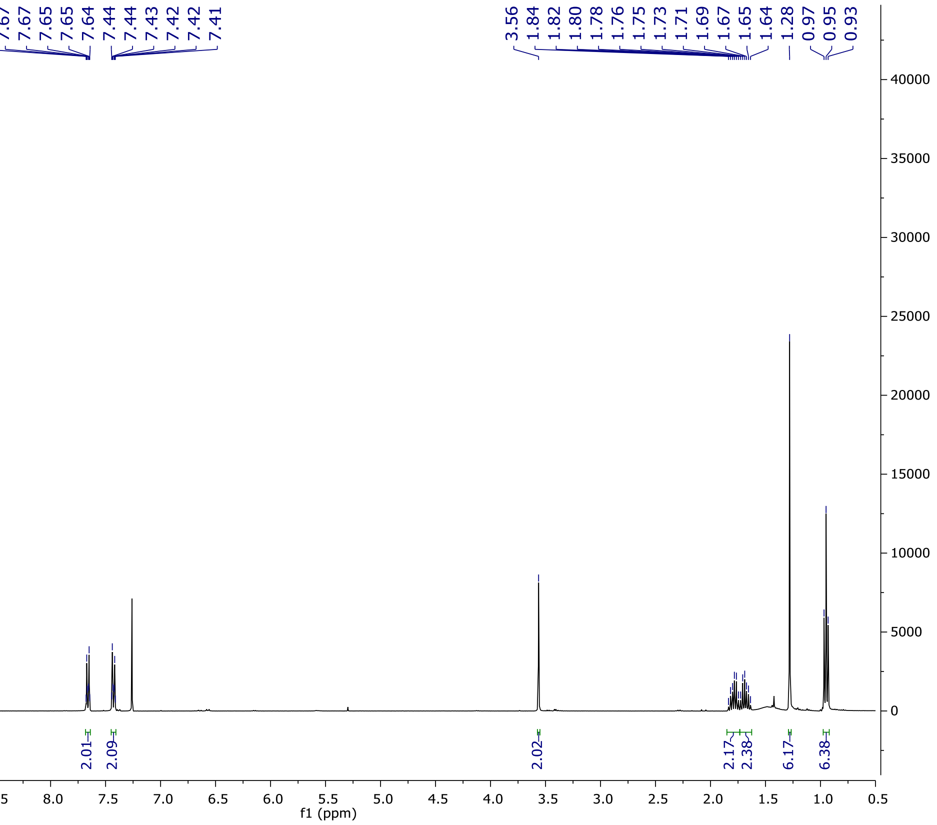

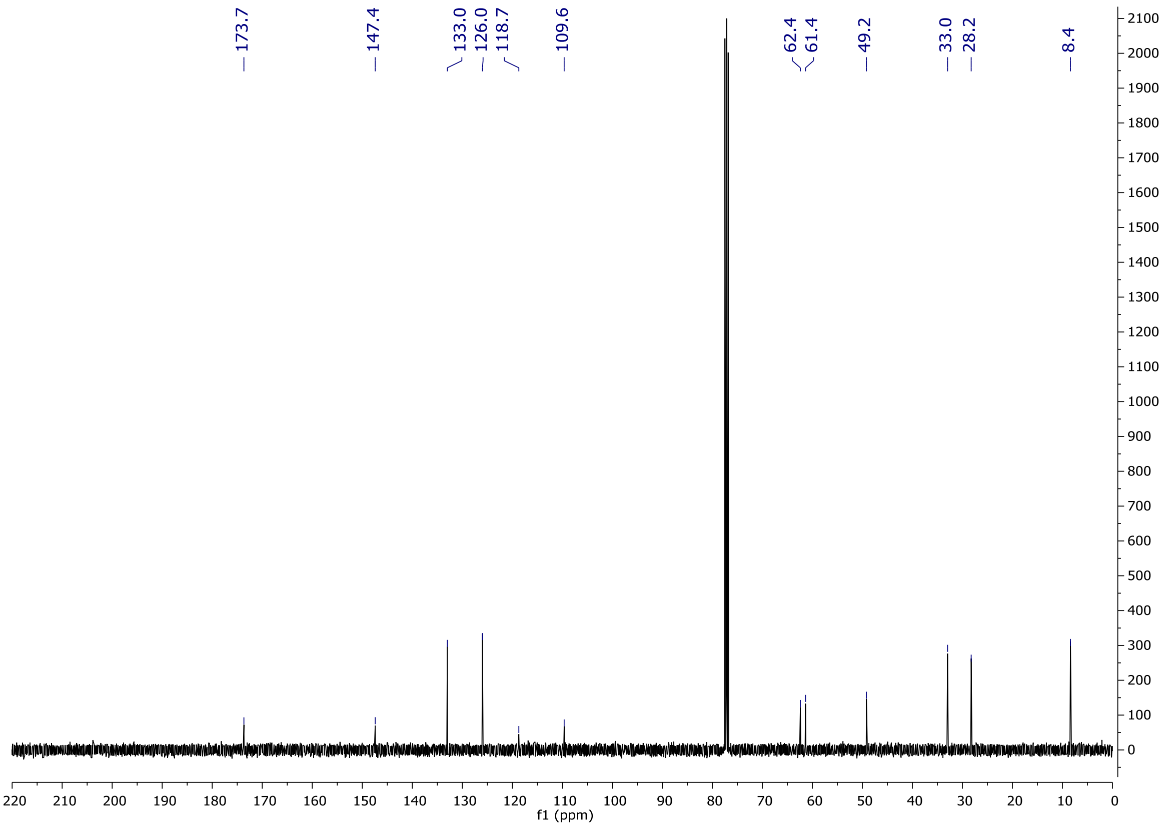

4-Benzyl-5,5-dimethyl-3-propylmorpholin-2-one

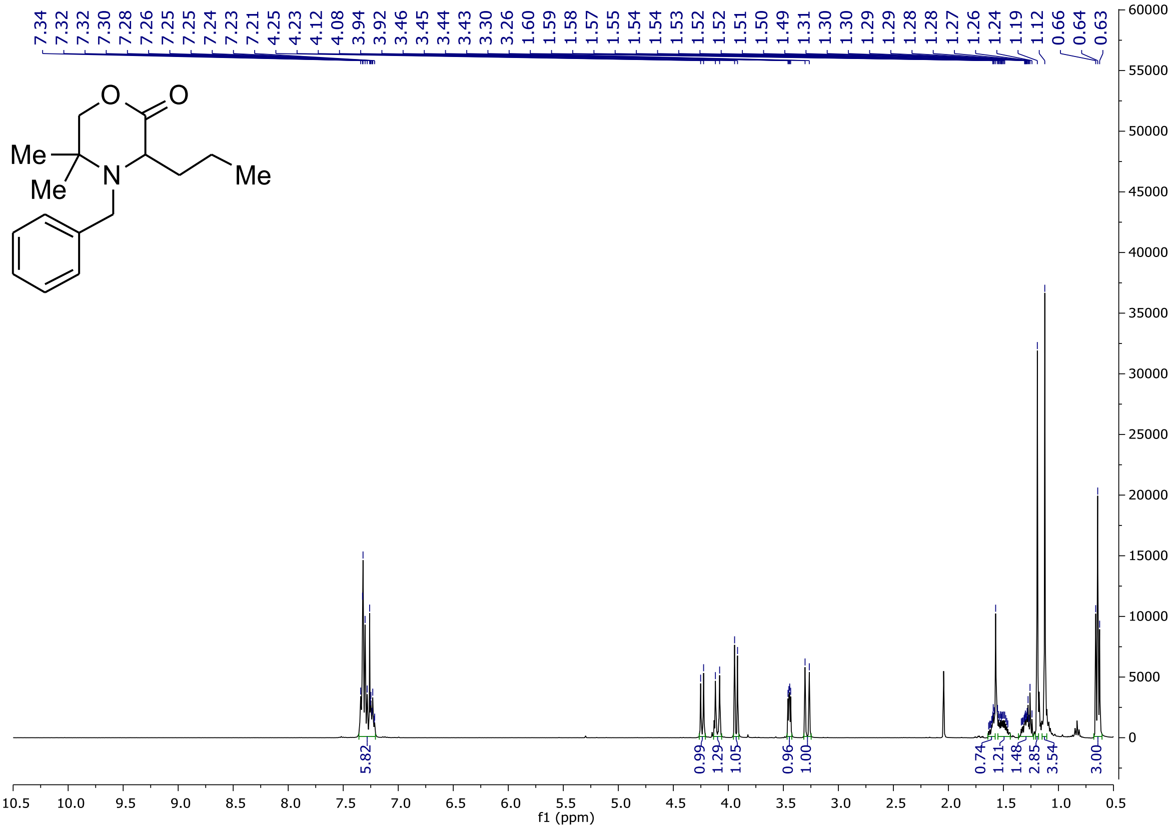

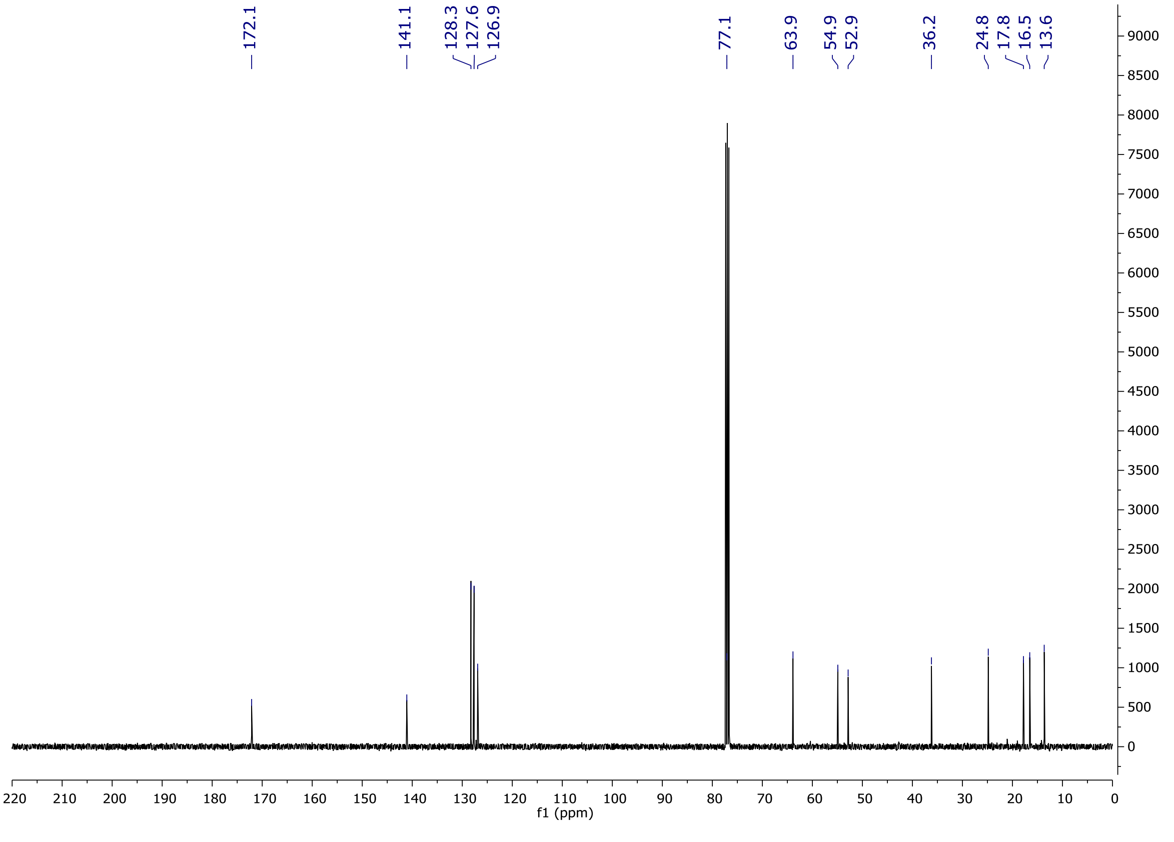

4-Benzyl-3-*d*[5]-ethyl-5,5-dimethyl-3-propylmorpholin-2-one

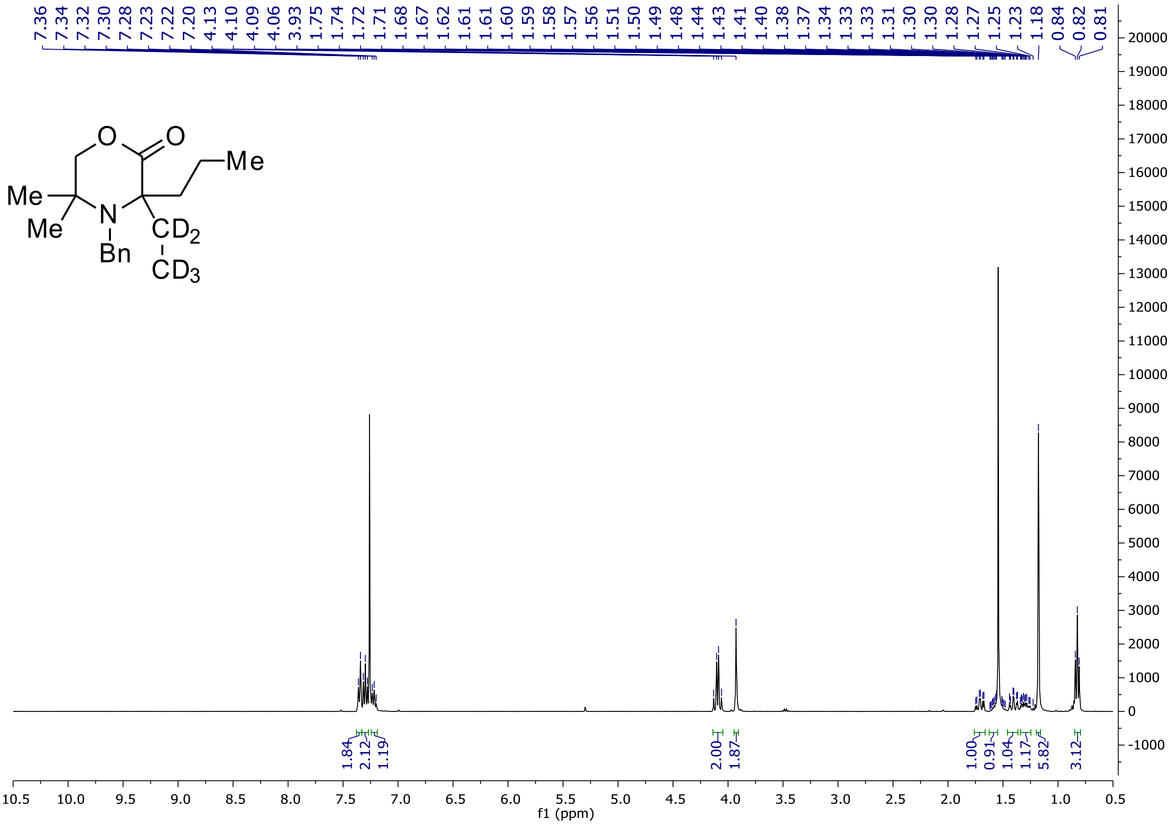

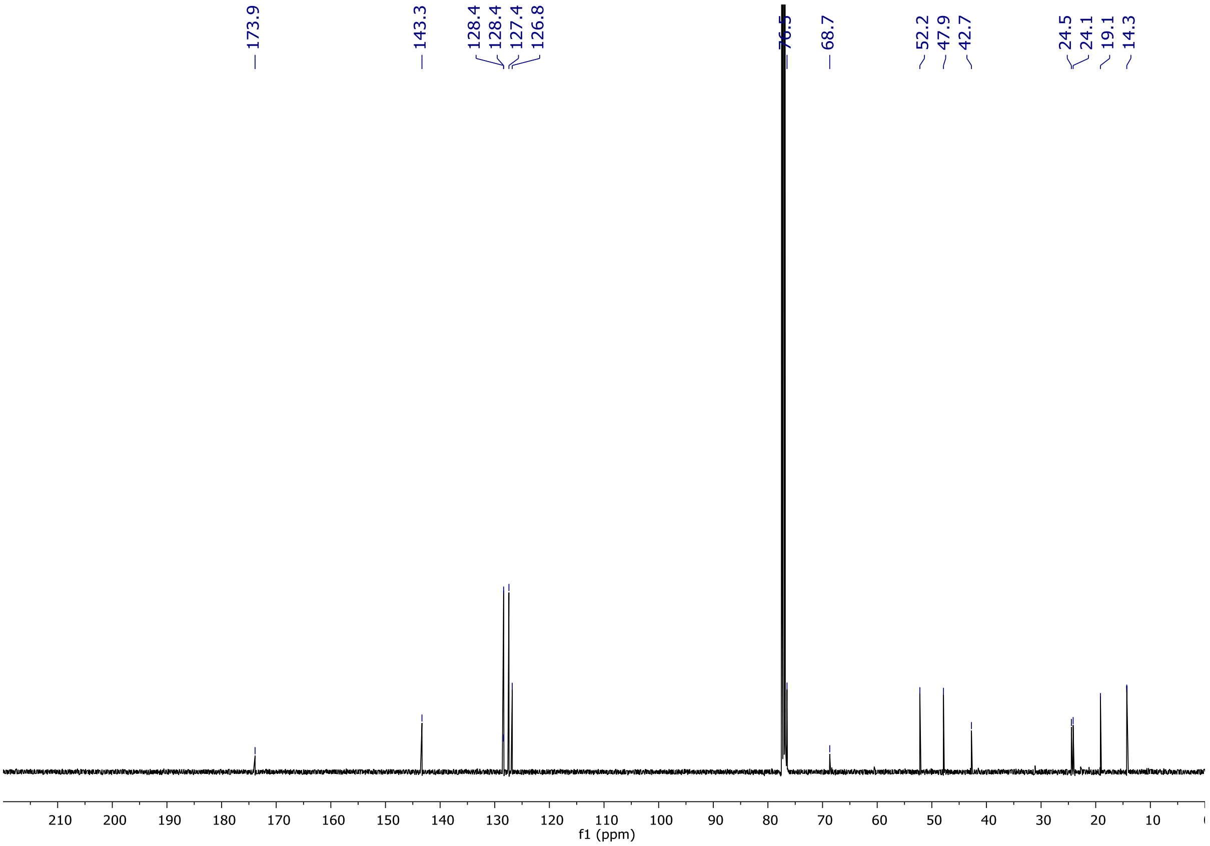

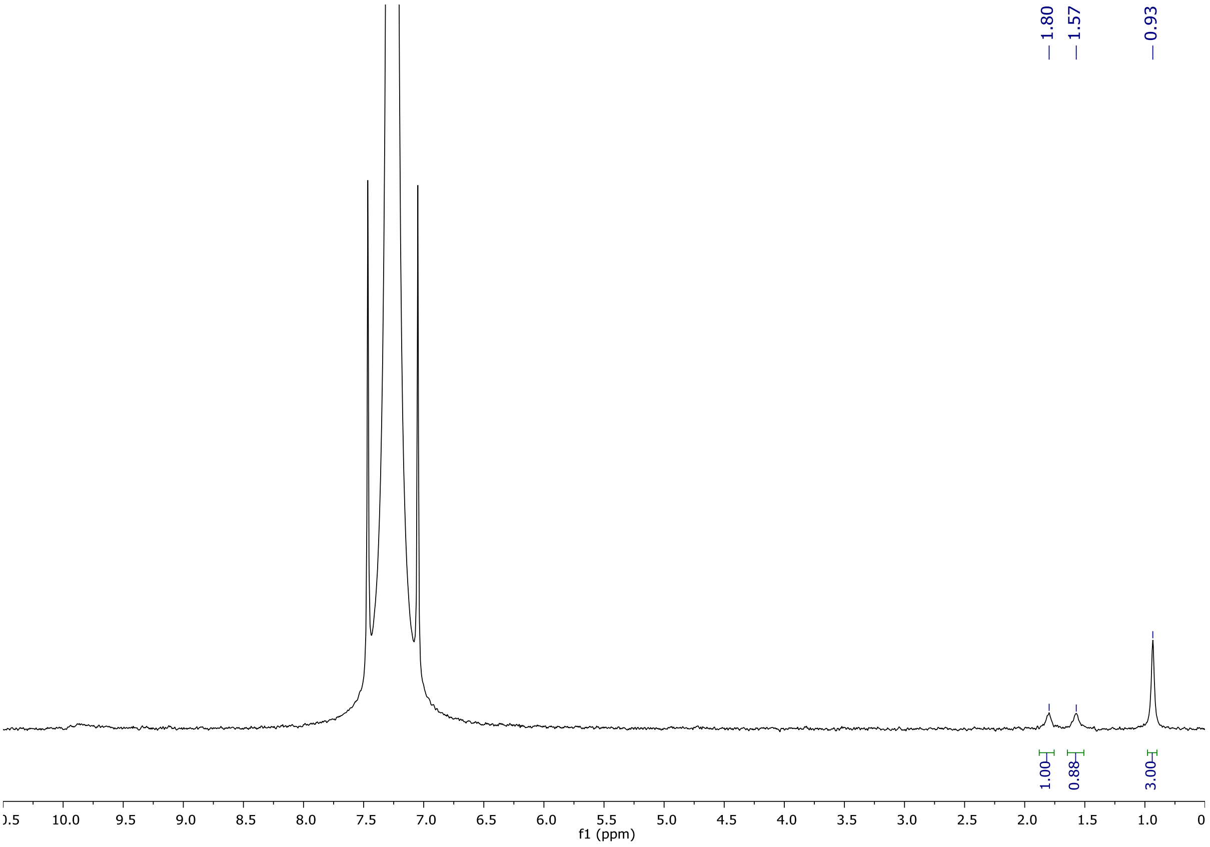

3-d^5-Ethyl-5,5-dimethyl-3-propylmorpholin-2-one d_5-1a

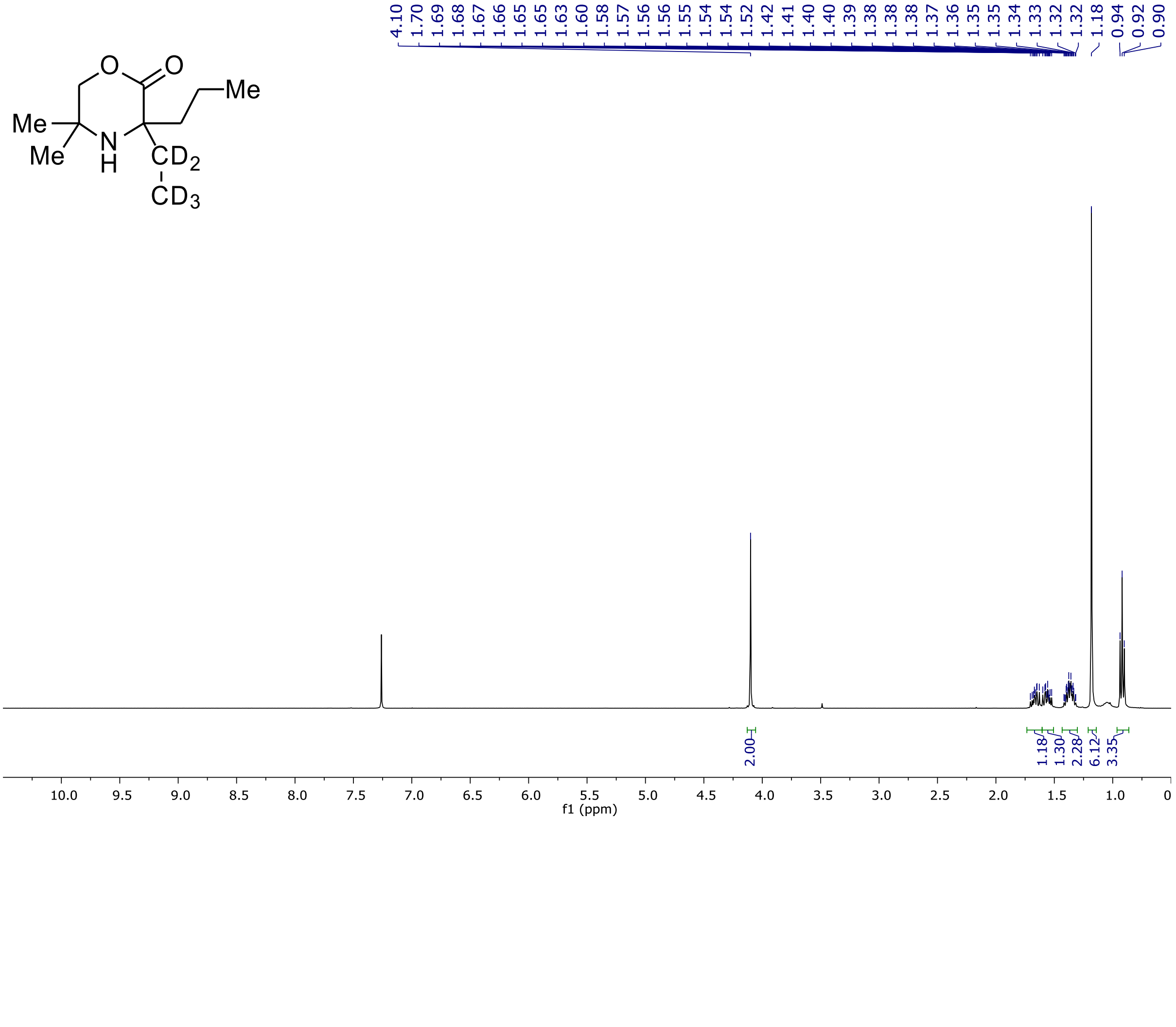

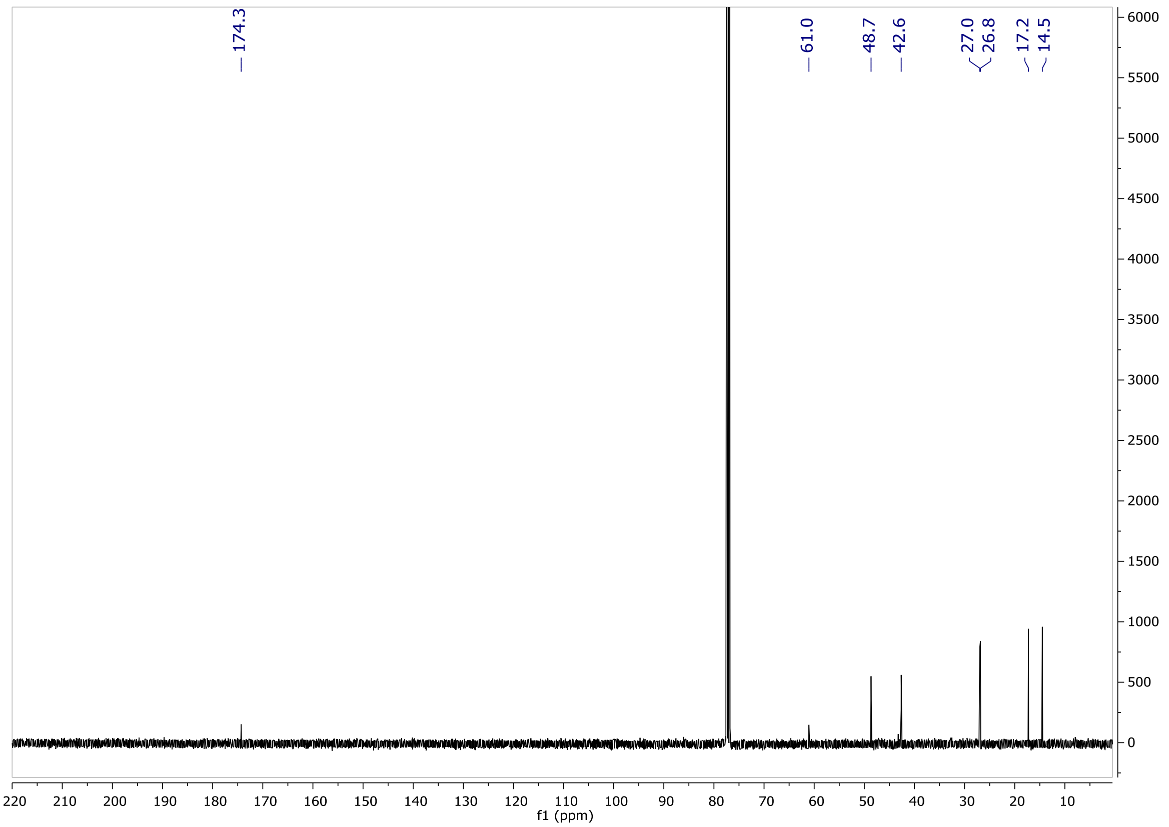
174.3
61.0
48.7
42.6
27.0
26.8
17.2
14.5
f1 (ppm)

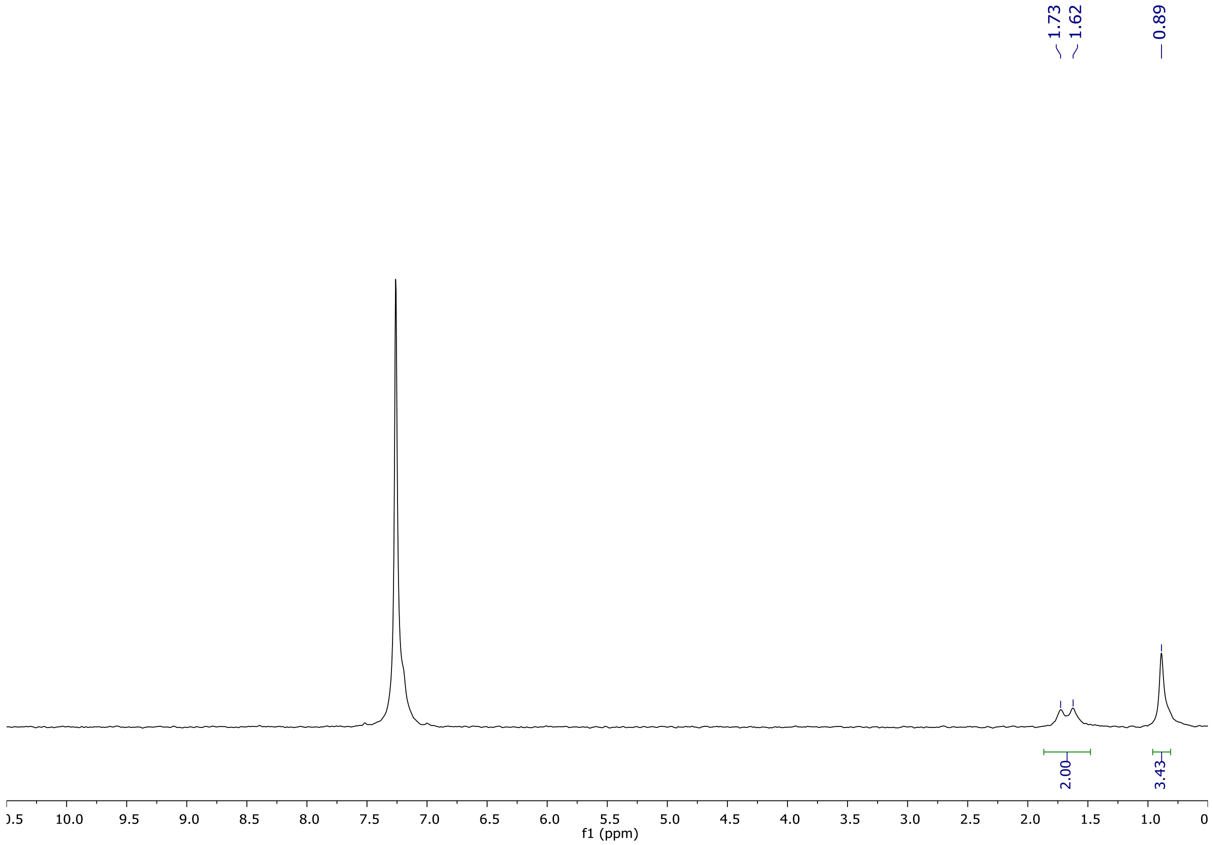
1.73
1.62
0.89
2.00
3.43
f1 (ppm)

2-(5,5-Dimethyl-2-oxo-3-propylmorpholin-3-yl)ethyl acetate, 2a

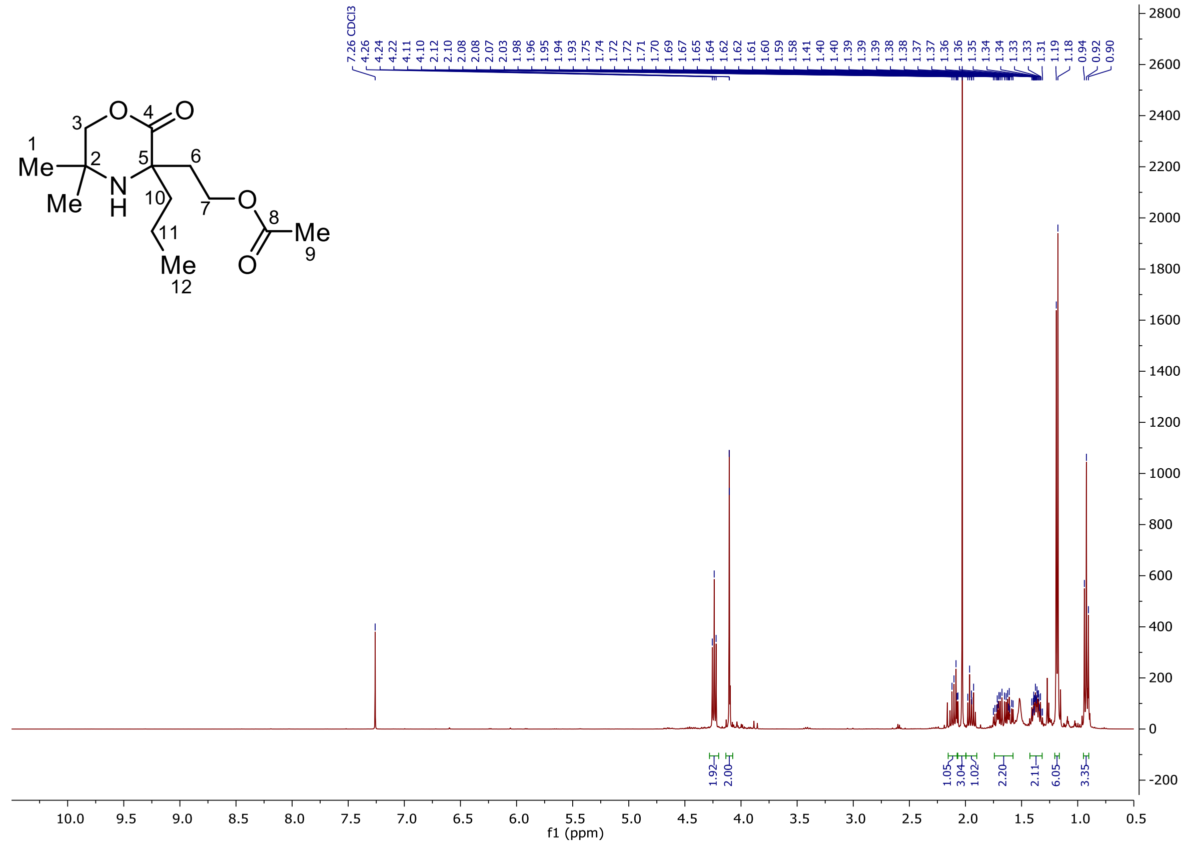

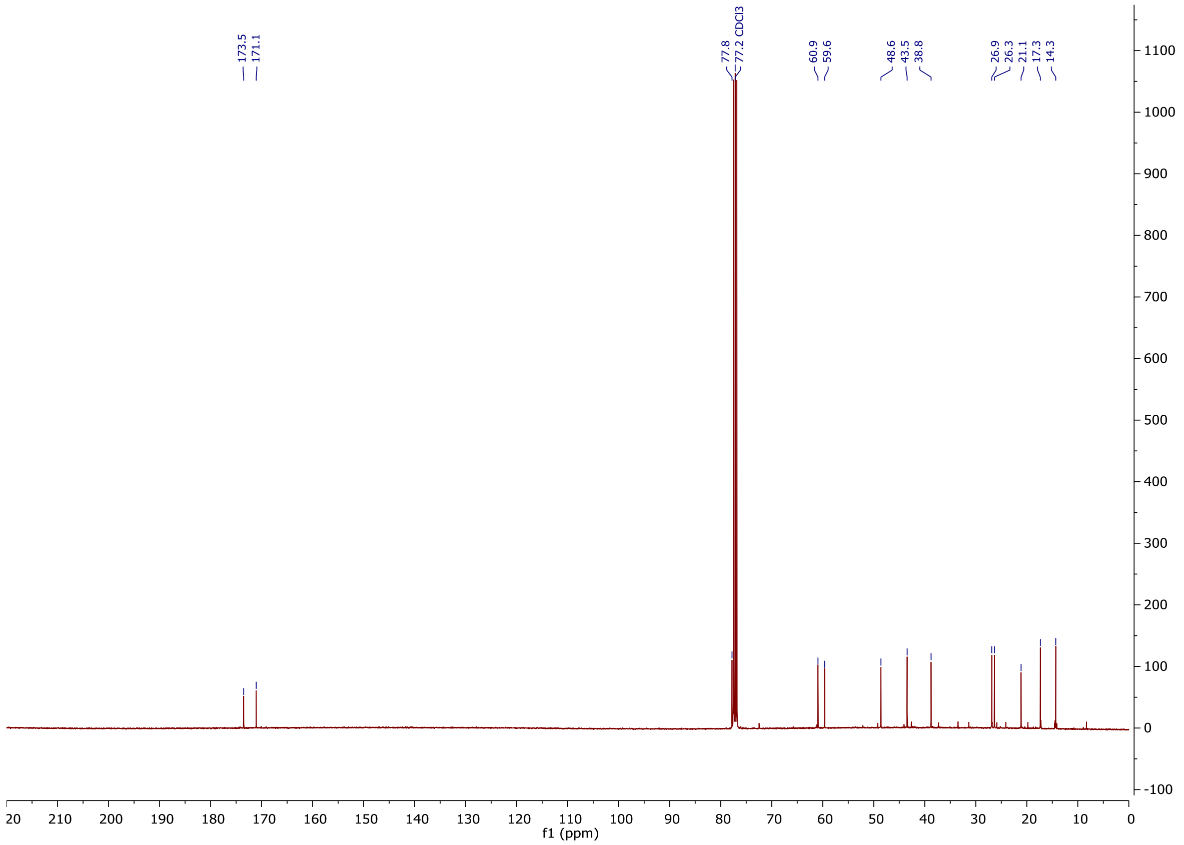

2-(3-Ethyl-5,5-dimethyl-2-oxomorpholin-3-yl)ethyl acetate 2b

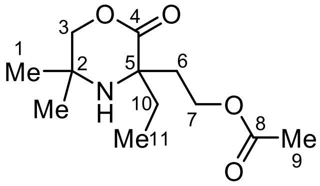

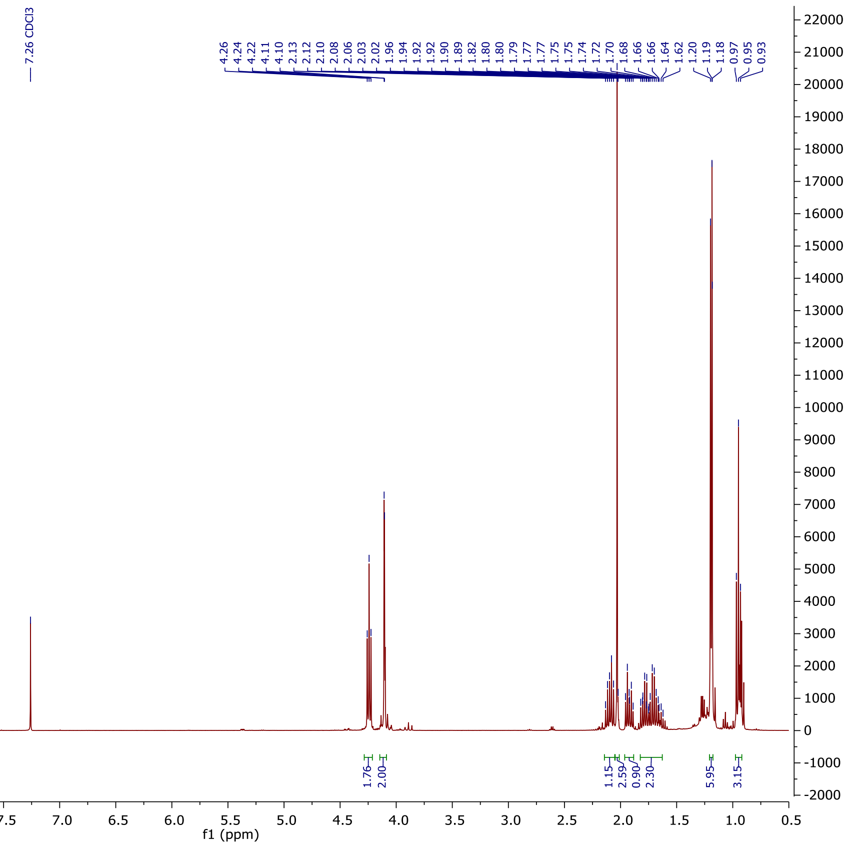

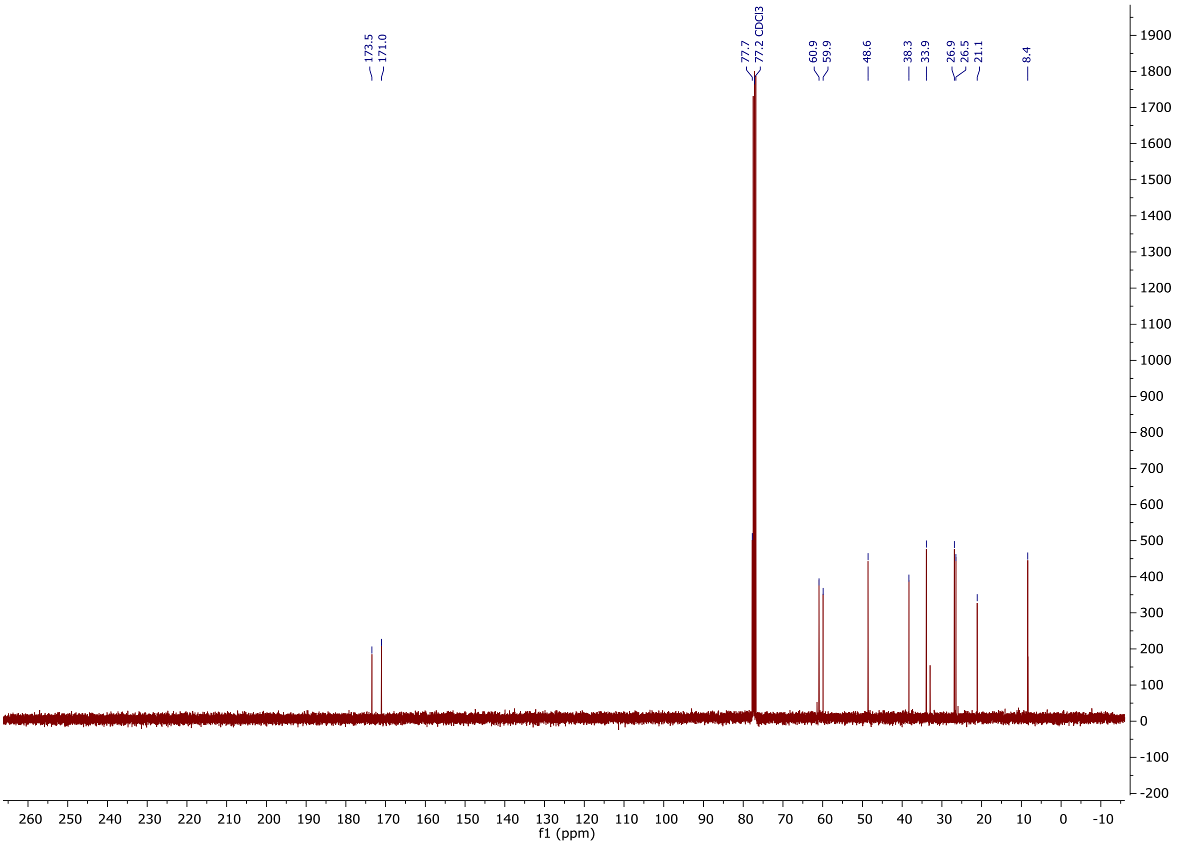

(5,5-Dimethyl-2-oxomorpholine-3,3-diyl)bis(ethane-2,1-diyl) diacetate

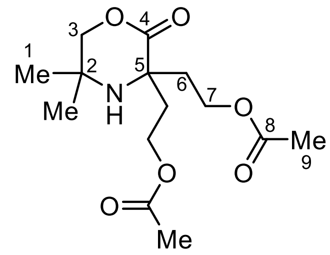

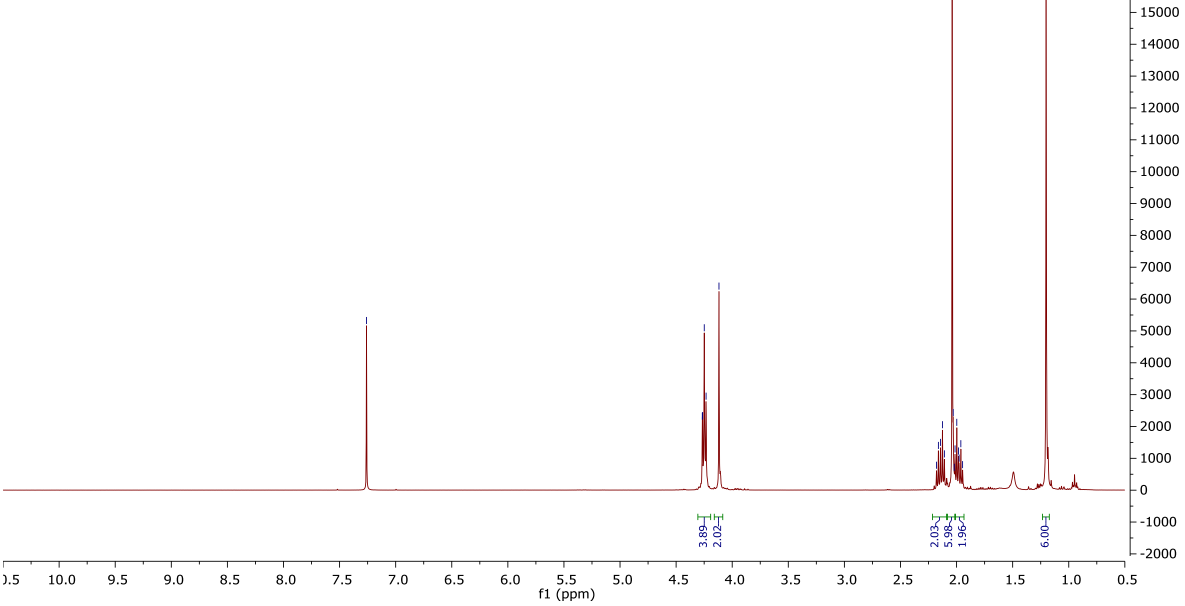

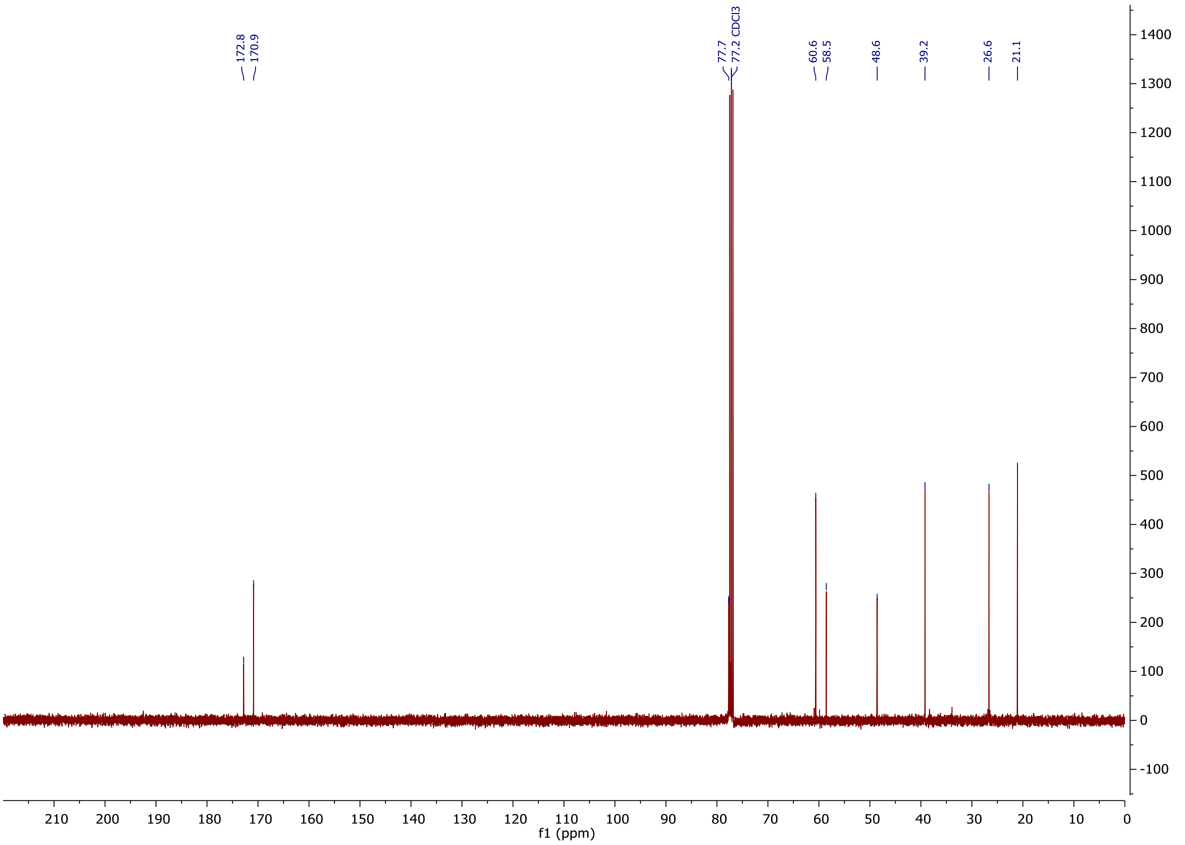

2-(5,5-Dimethyl-2-oxo-3-phenethylmorpholin-3-yl)ethyl acetate 2c

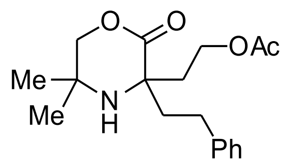

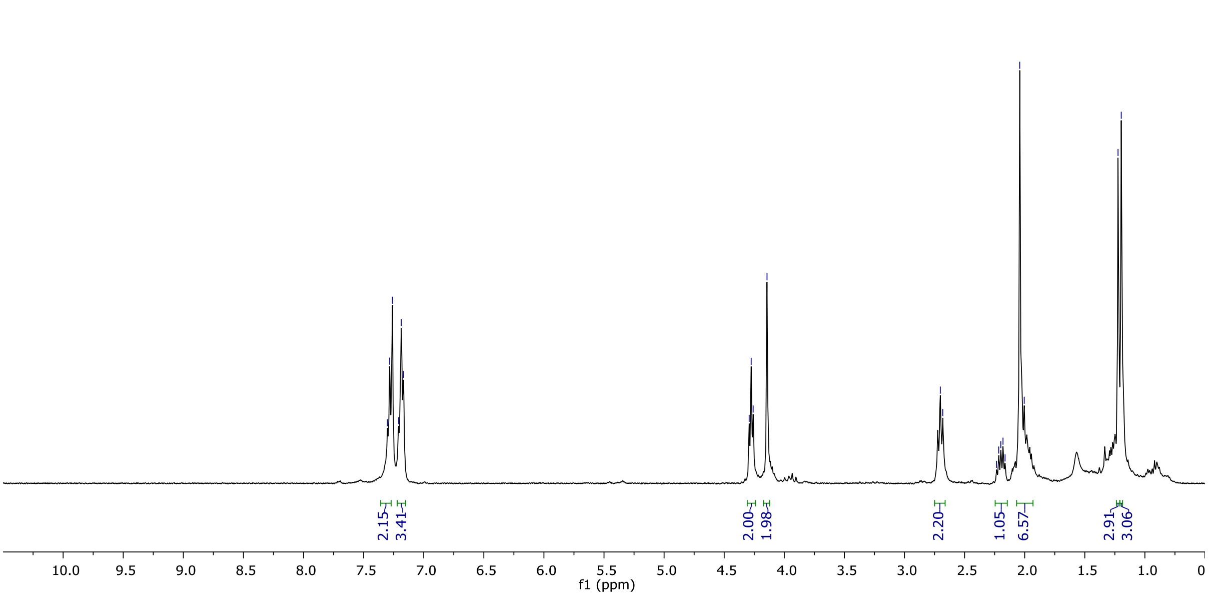

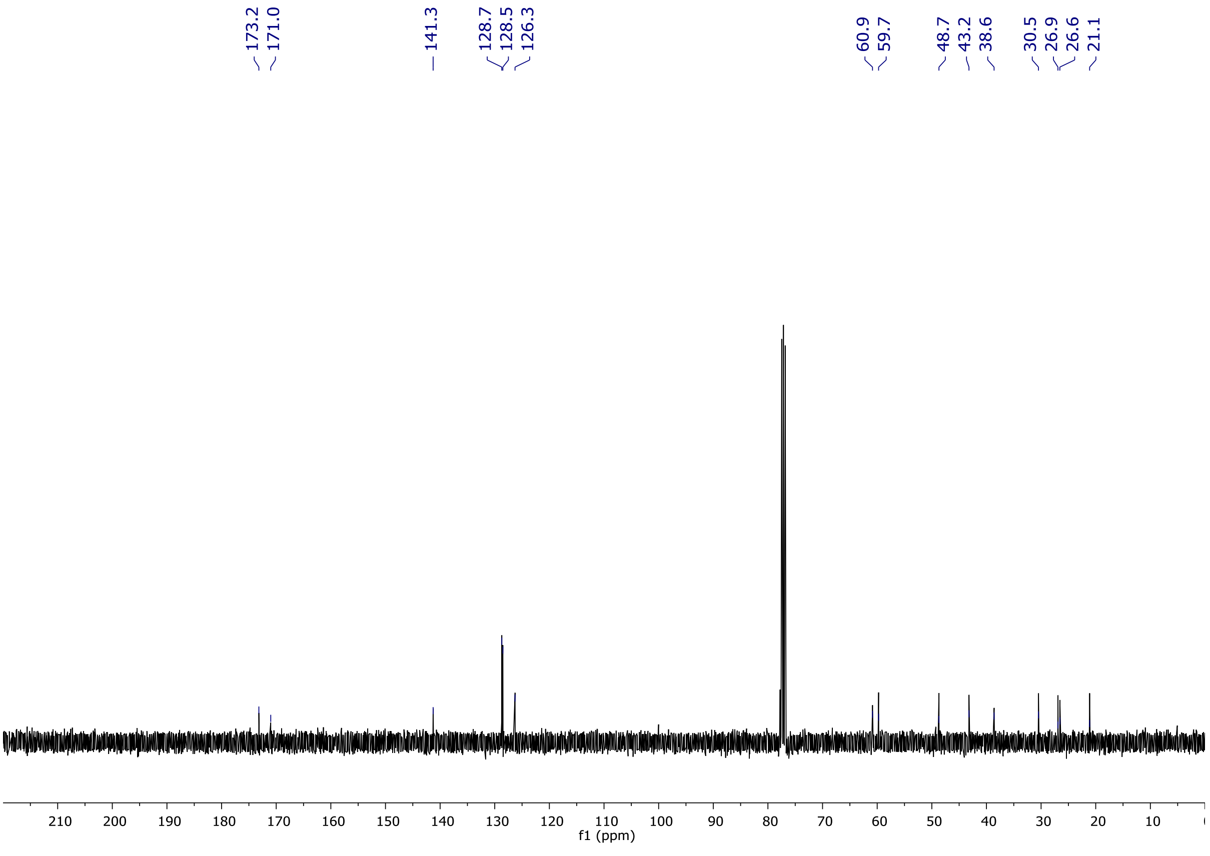

Ethyl 4-(3-(2-acetoxyethyl)-5,5-dimethyl-2-oxomorpholin-3-yl)butanoate 2d

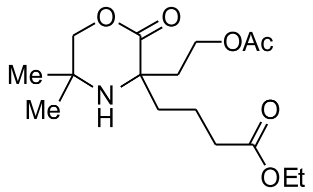

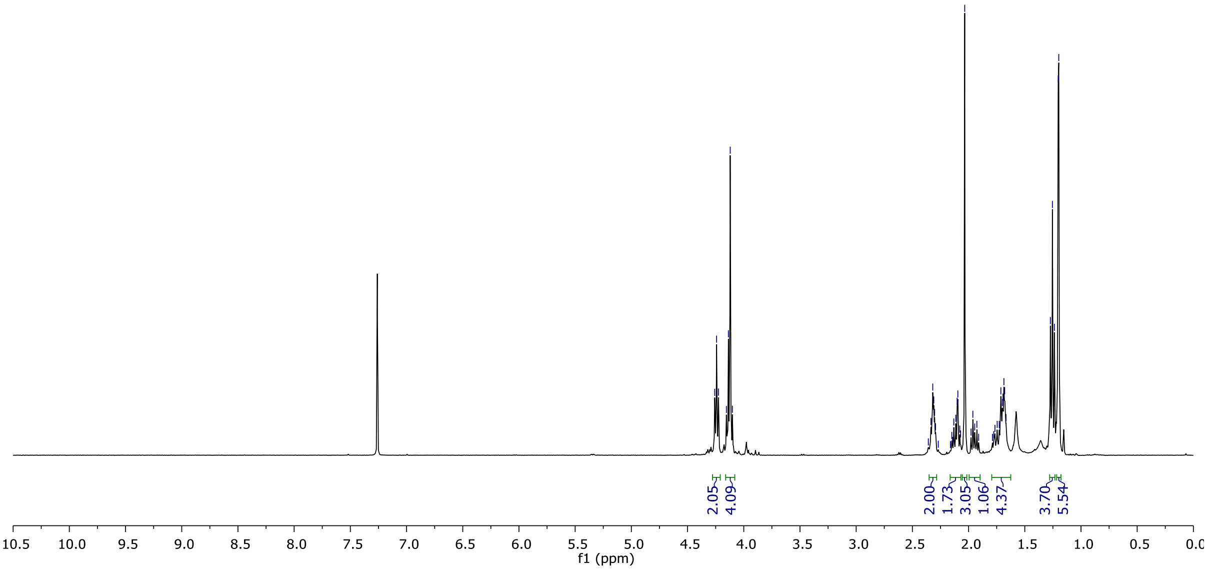

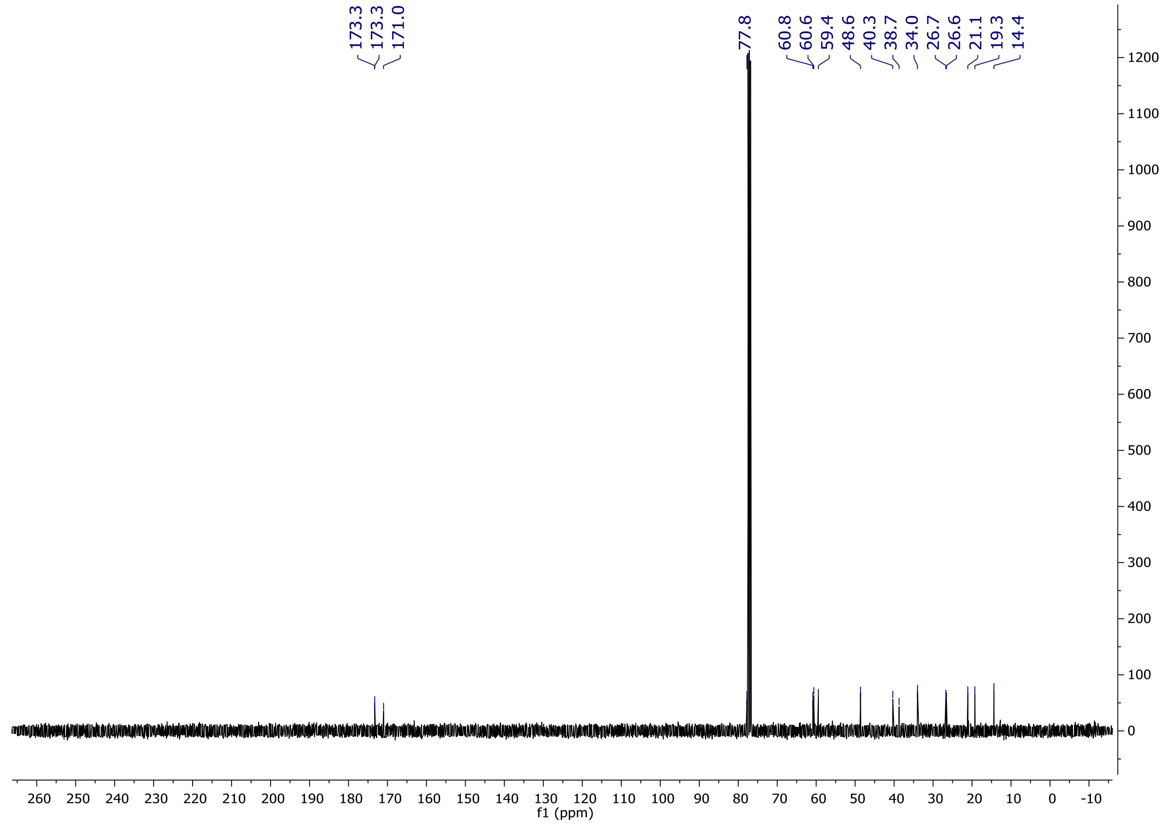

2-(2-Ethyl-3-oxo-4-oxa-1-azaspiro[5.5]undecan-2-yl)ethyl acetate 2e

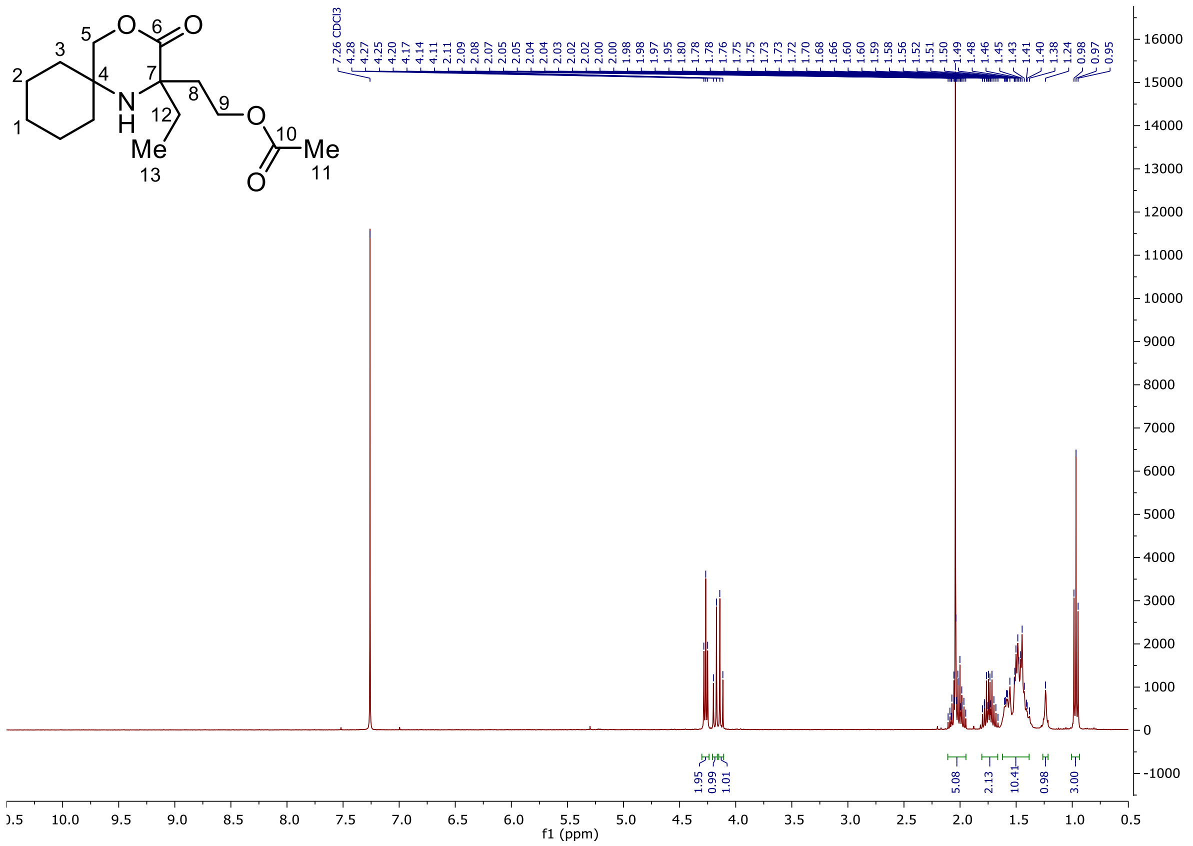

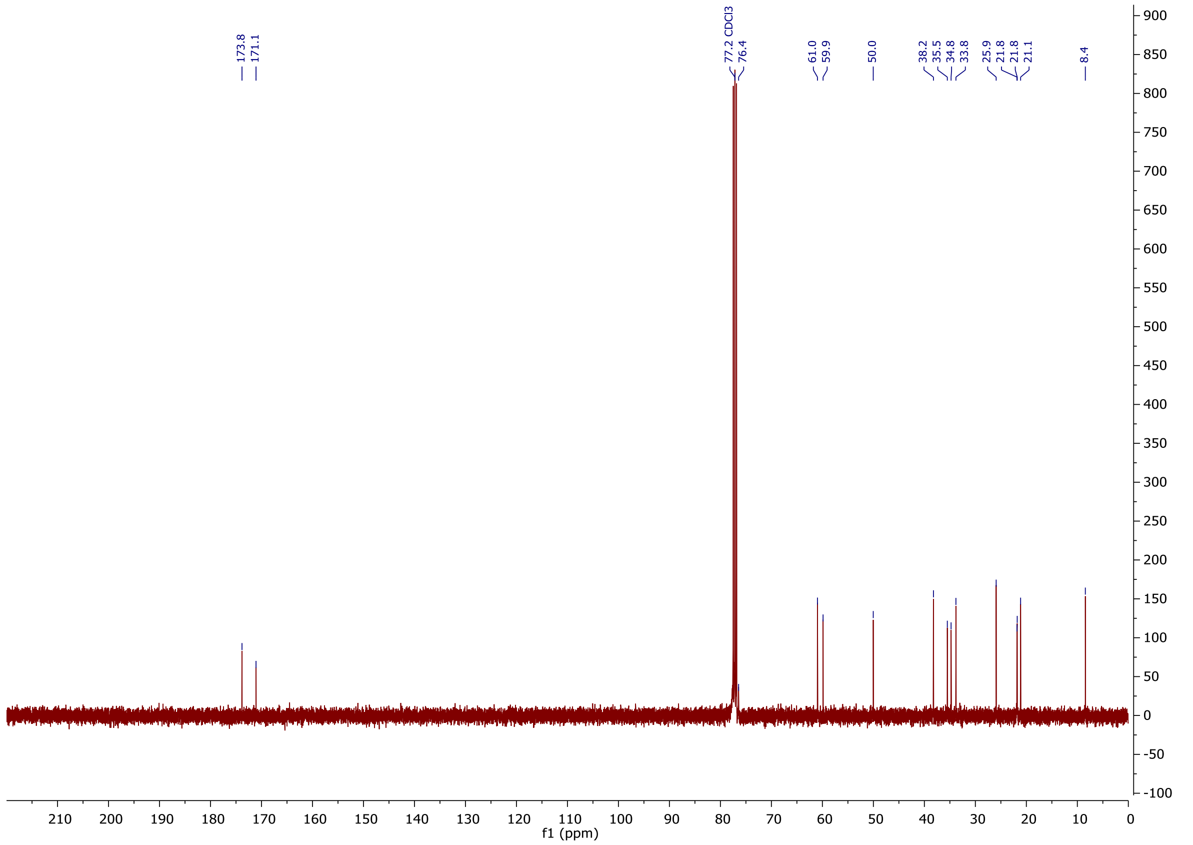

2-(3-(3-Cyanopropyl)-5,5-dimethyl-2-oxomorpholin-3-yl)ethyl acetate 2f

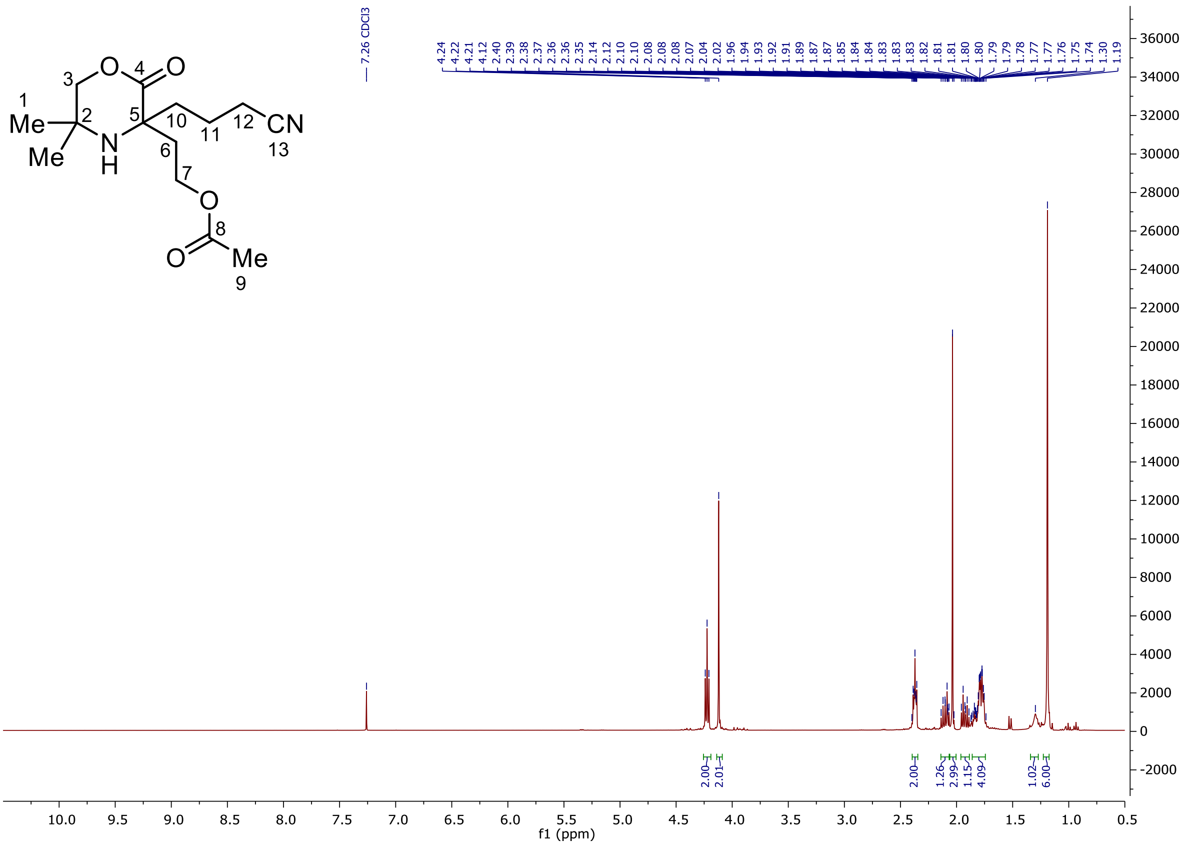

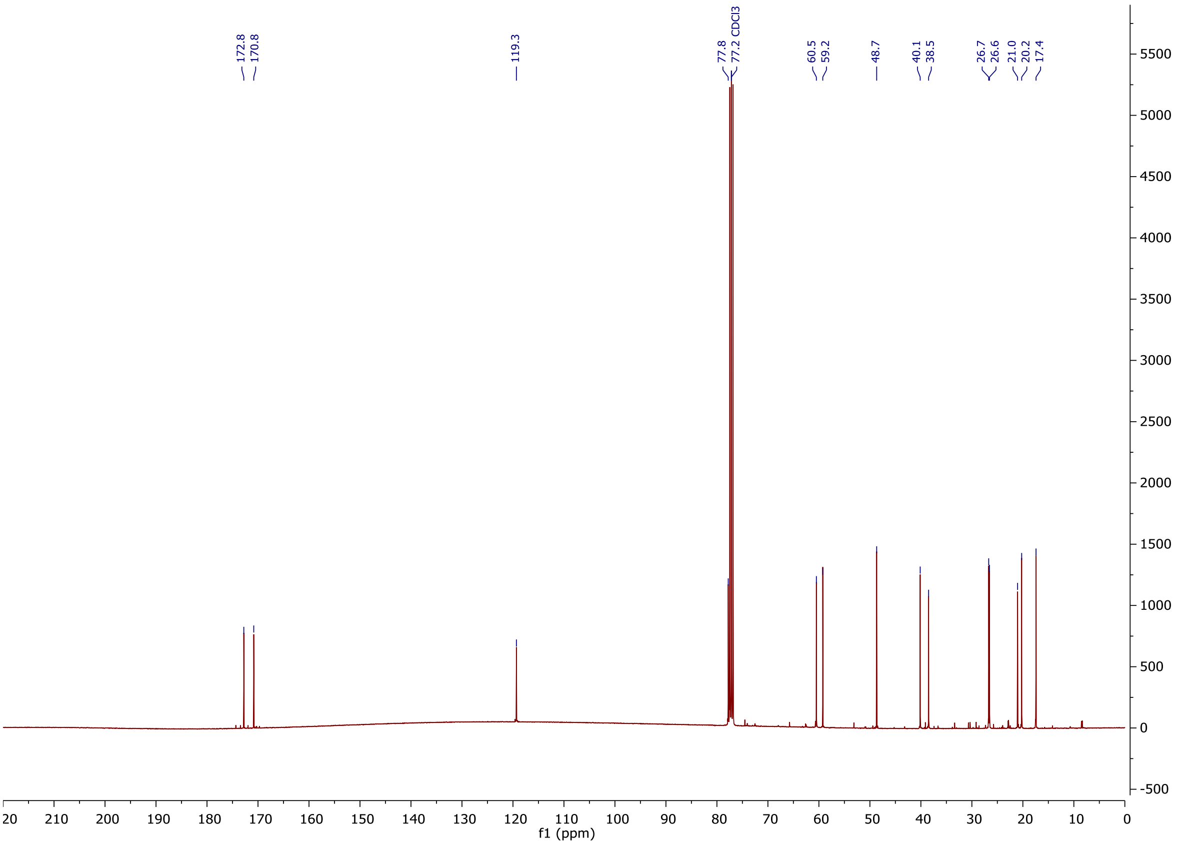

2-(5,5-Dimethyl-2-oxo-3-(3-(phenylsulfonyl)propyl)morpholin-3-yl)ethyl acetate 2g

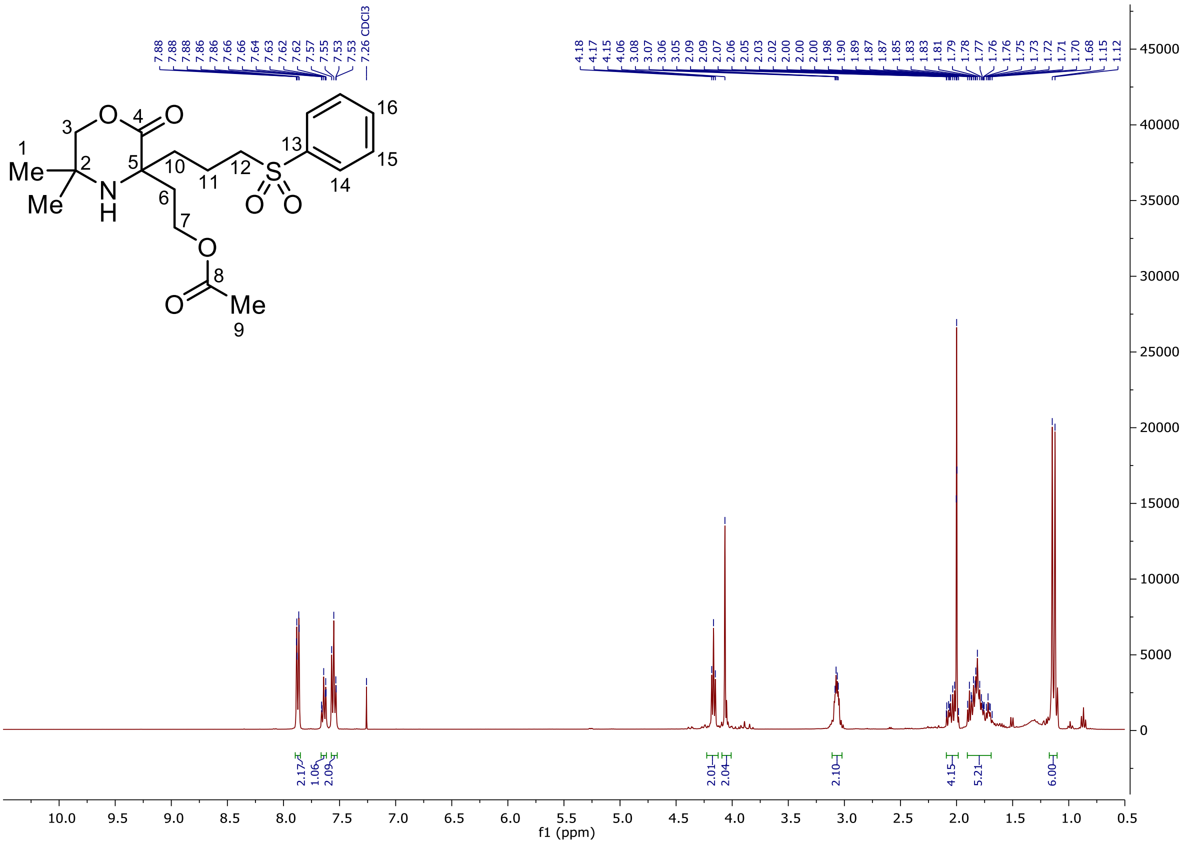

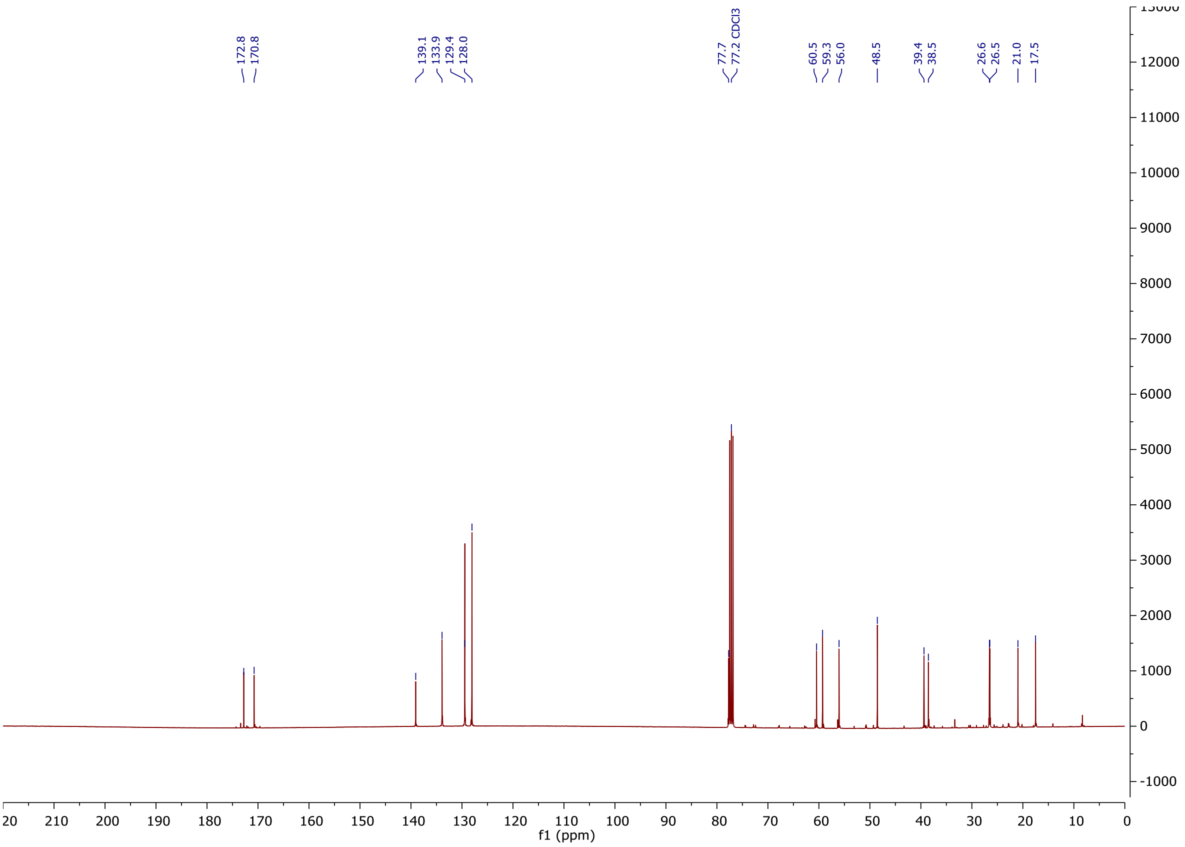

2-(3-(3-(1,3-dioxoisoindolin-2-yl)propyl)-5,5-dimethyl-2-oxomorpholin-3-yl)ethyl acetate 2h

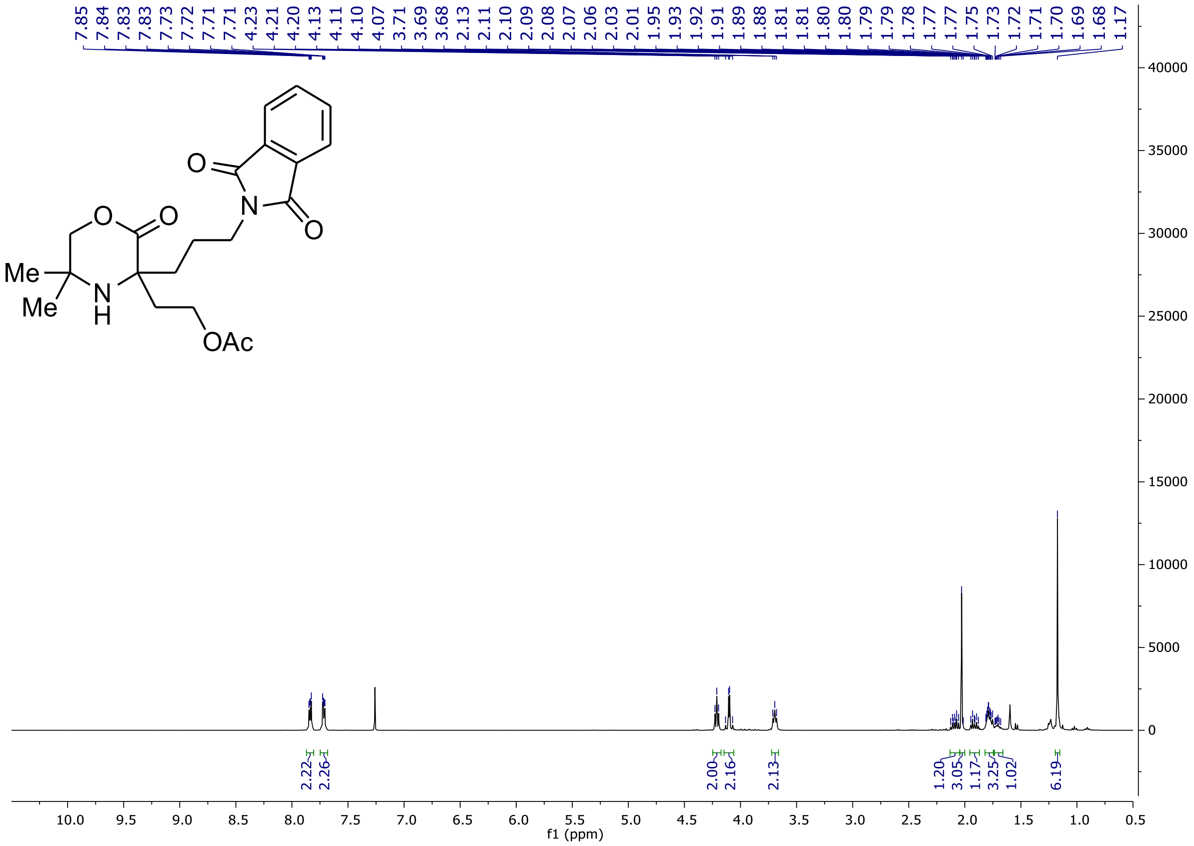

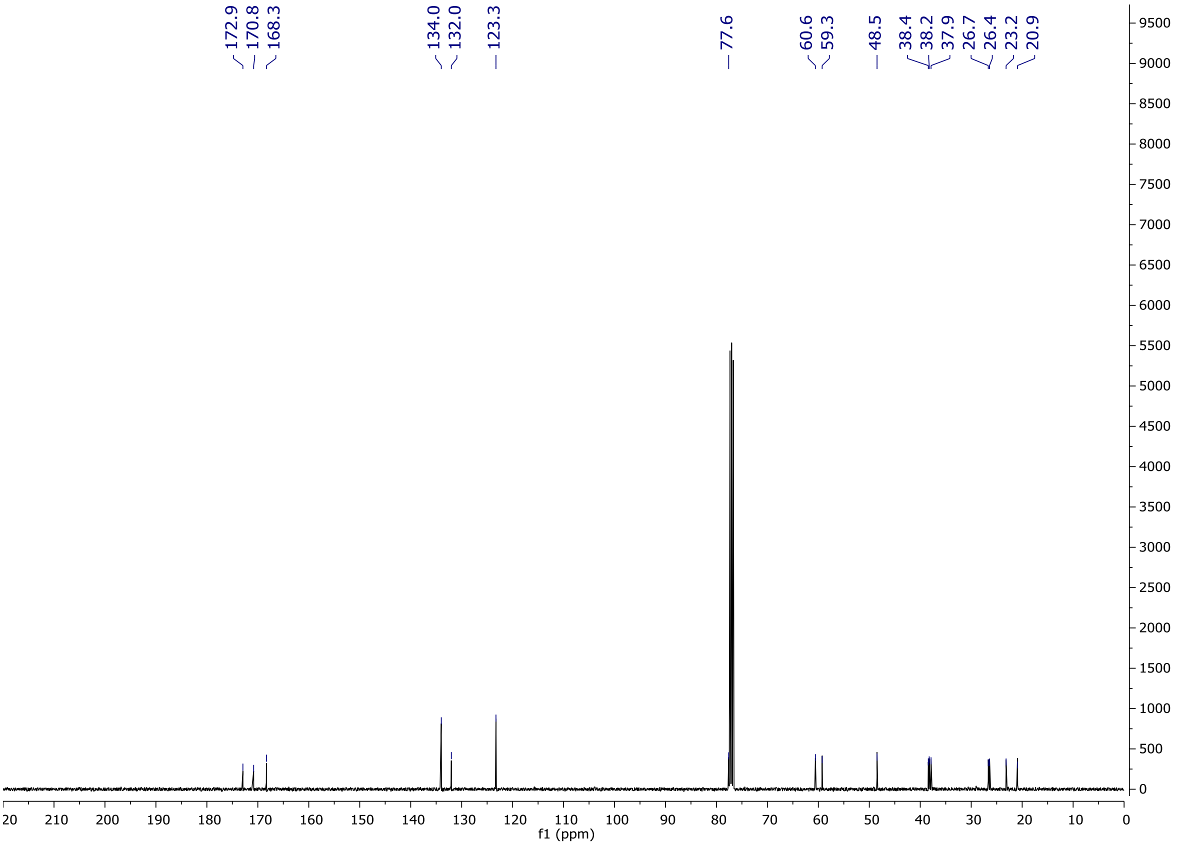

2-(3-((Benzyloxy)methyl)-5,5-dimethyl-2-oxomorpholin-3-yl)ethyl acetate 2i

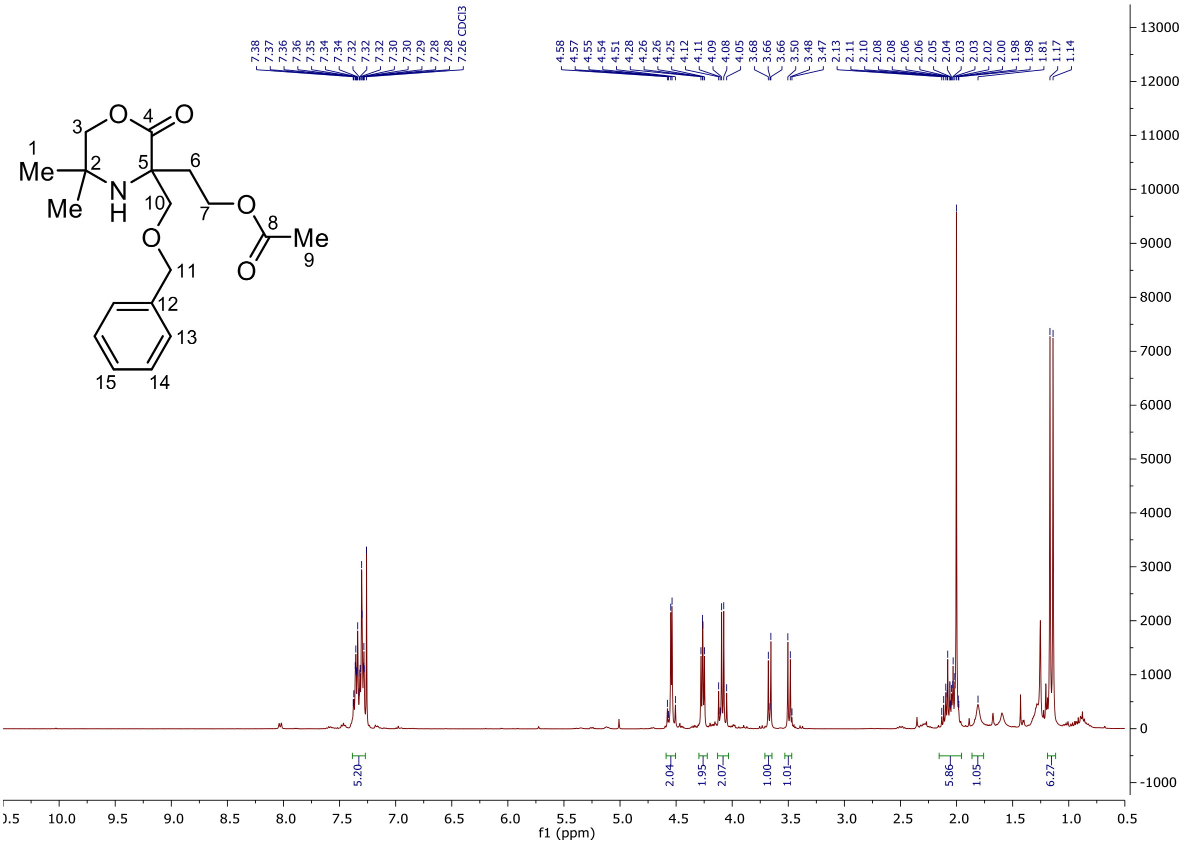

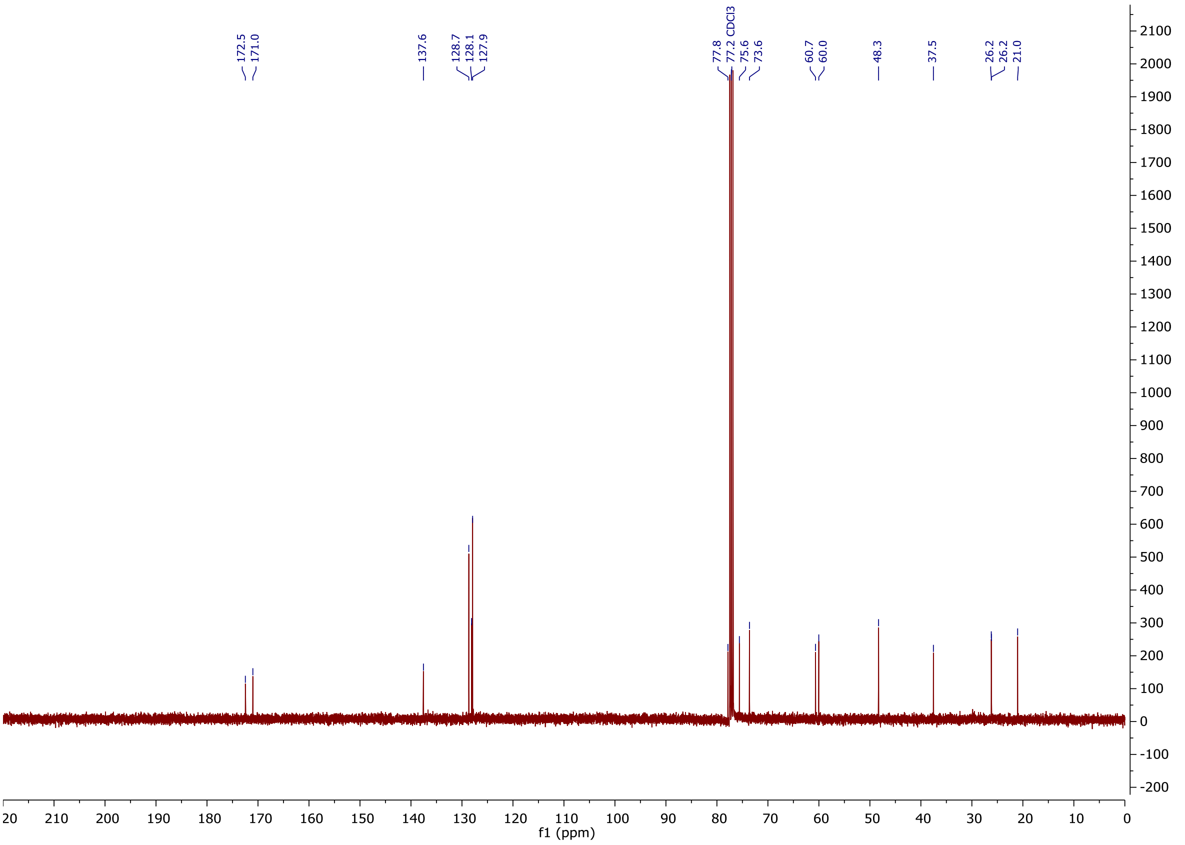

2-(4-(4-Cyanophenyl)-2-ethyl-6,6-dimethyl-3-oxopiperazin-2-yl)ethyl acetate 2j

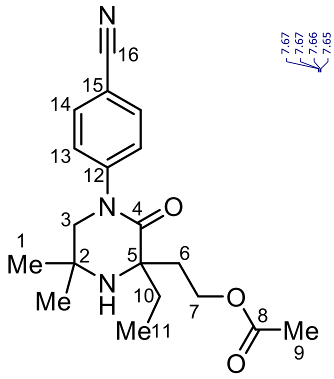

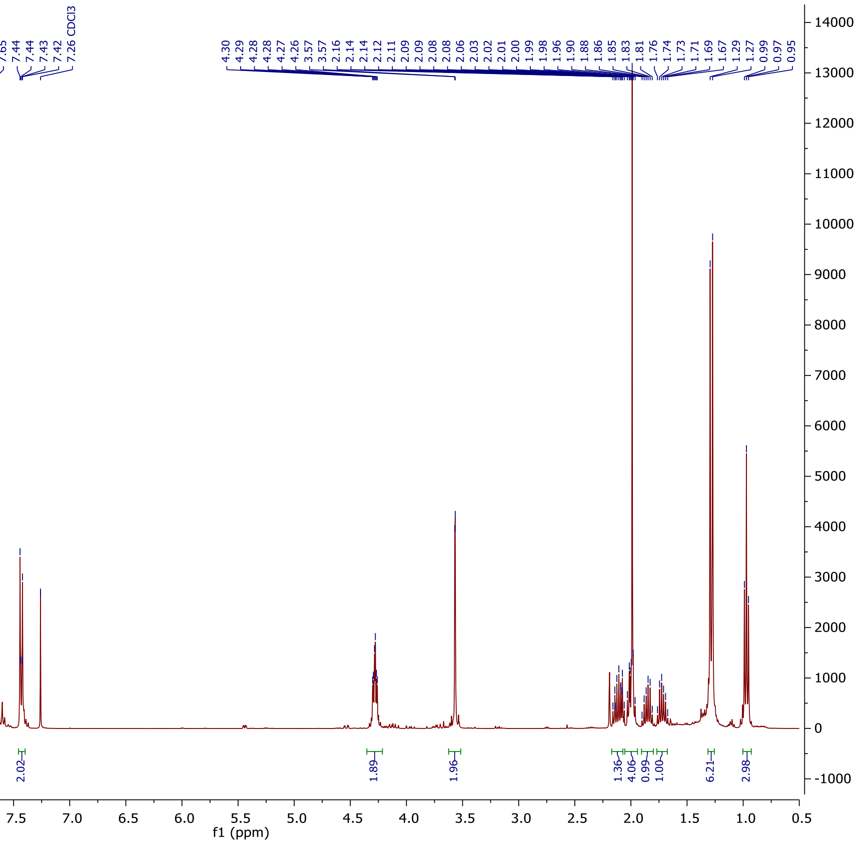

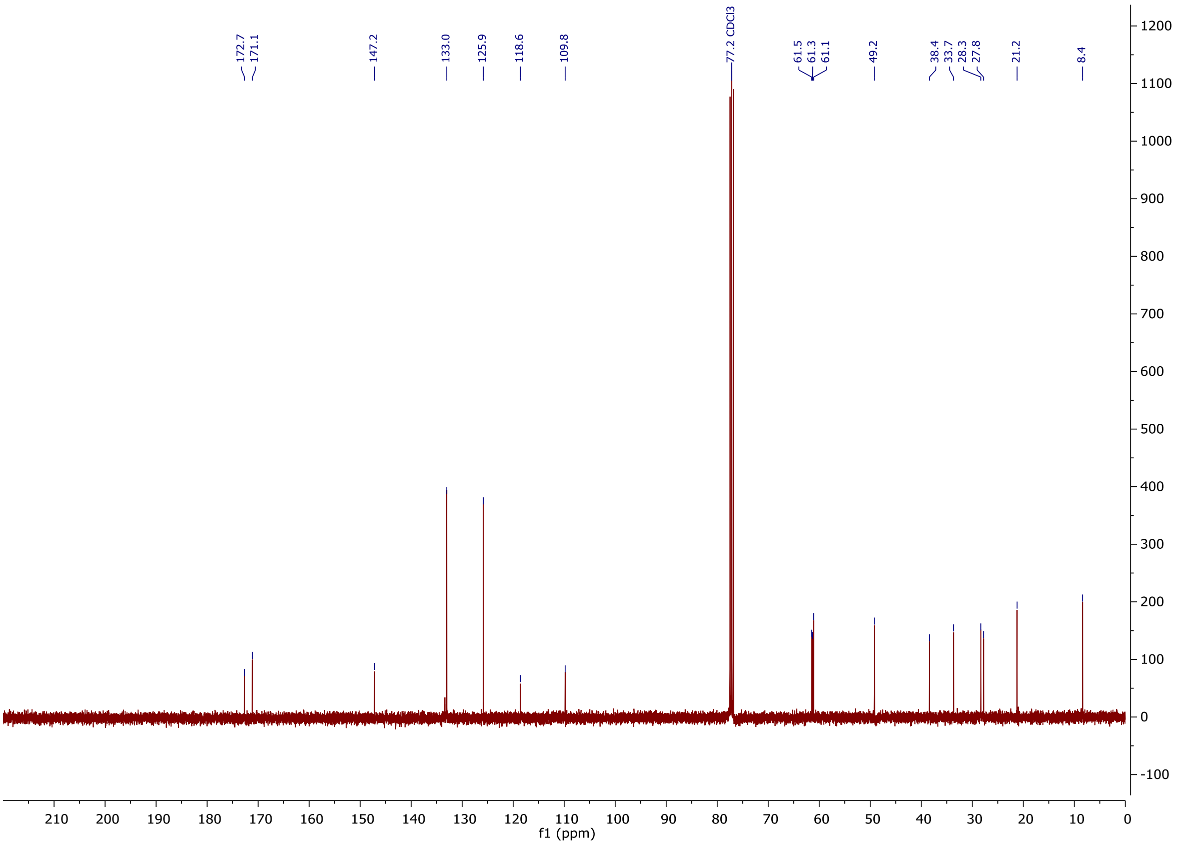

3-((1-hydroxy-2-methylpropan-2-yl)amino)-3-propyldihydrofuran-2(3H)-one

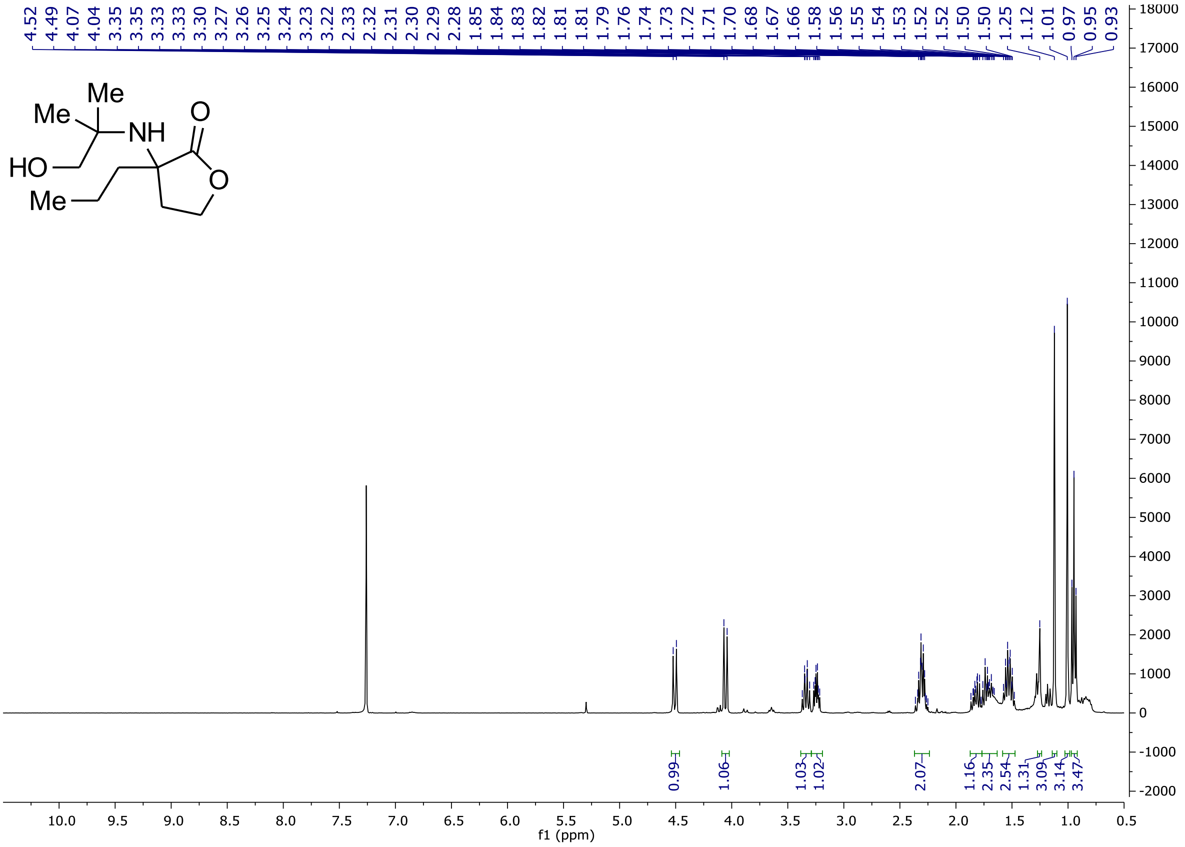

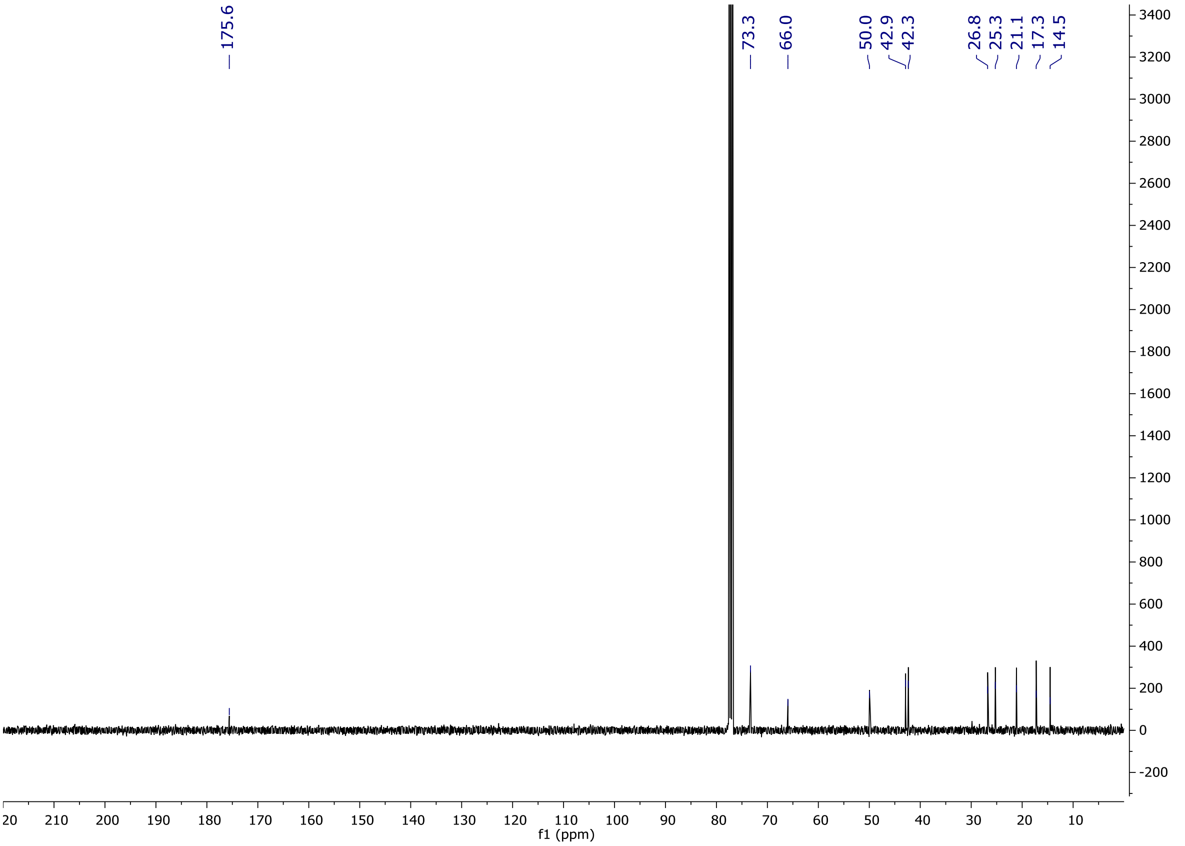

2-(4-(4-(aminomethyl)phenyl)-2-ethyl-6,6-dimethylpiperazin-2-yl)ethanol 3

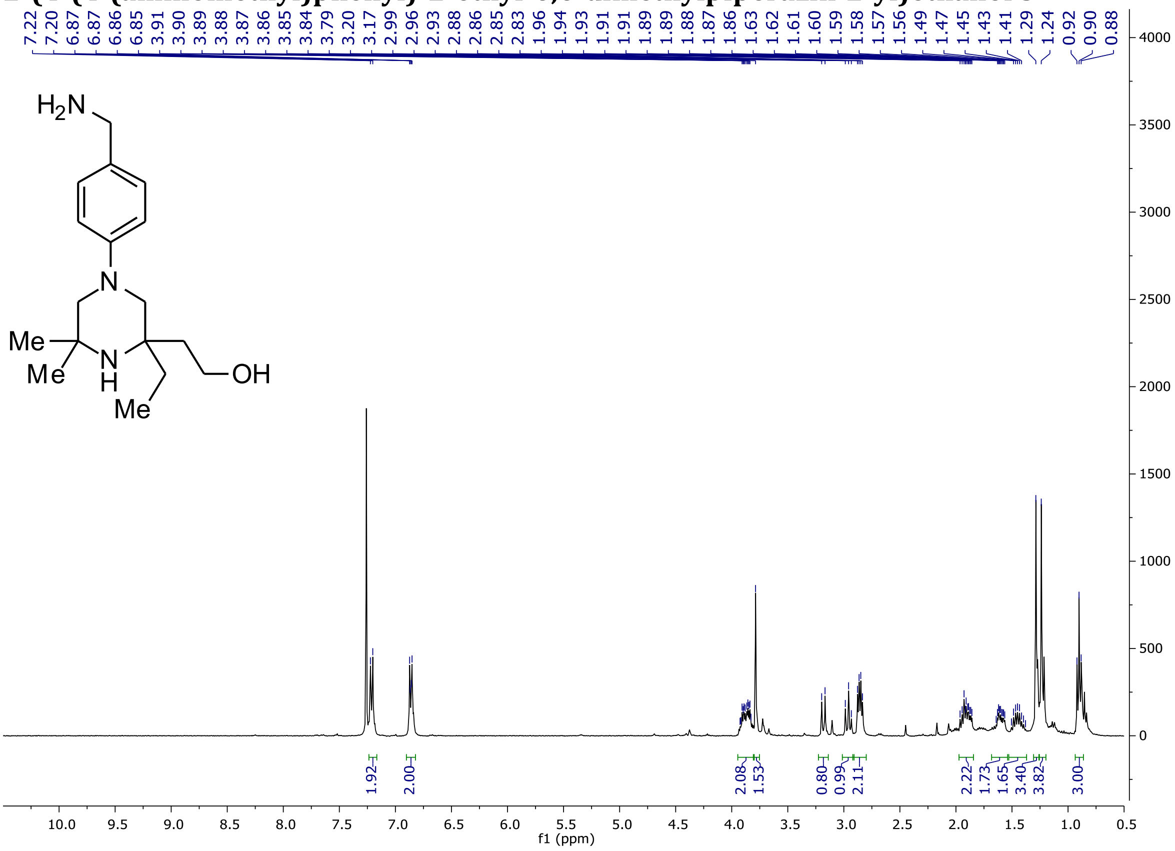

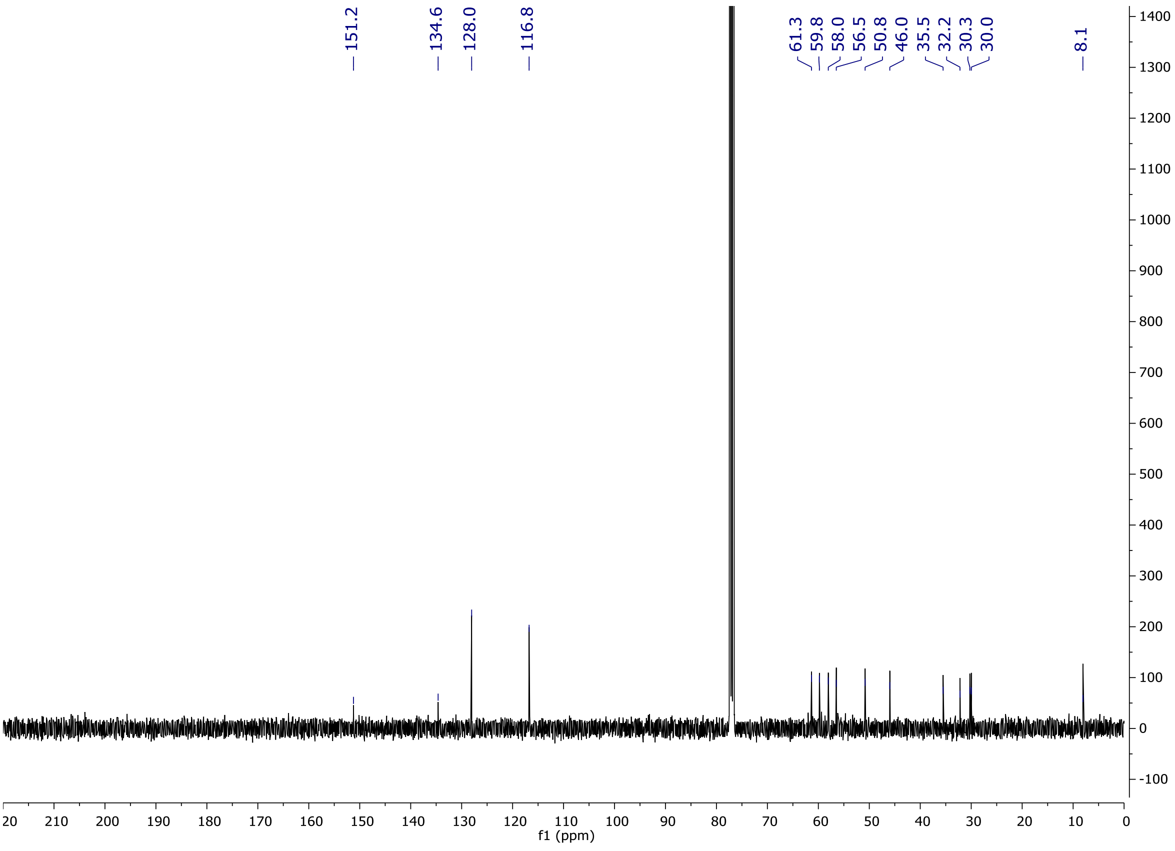

Palladacycle

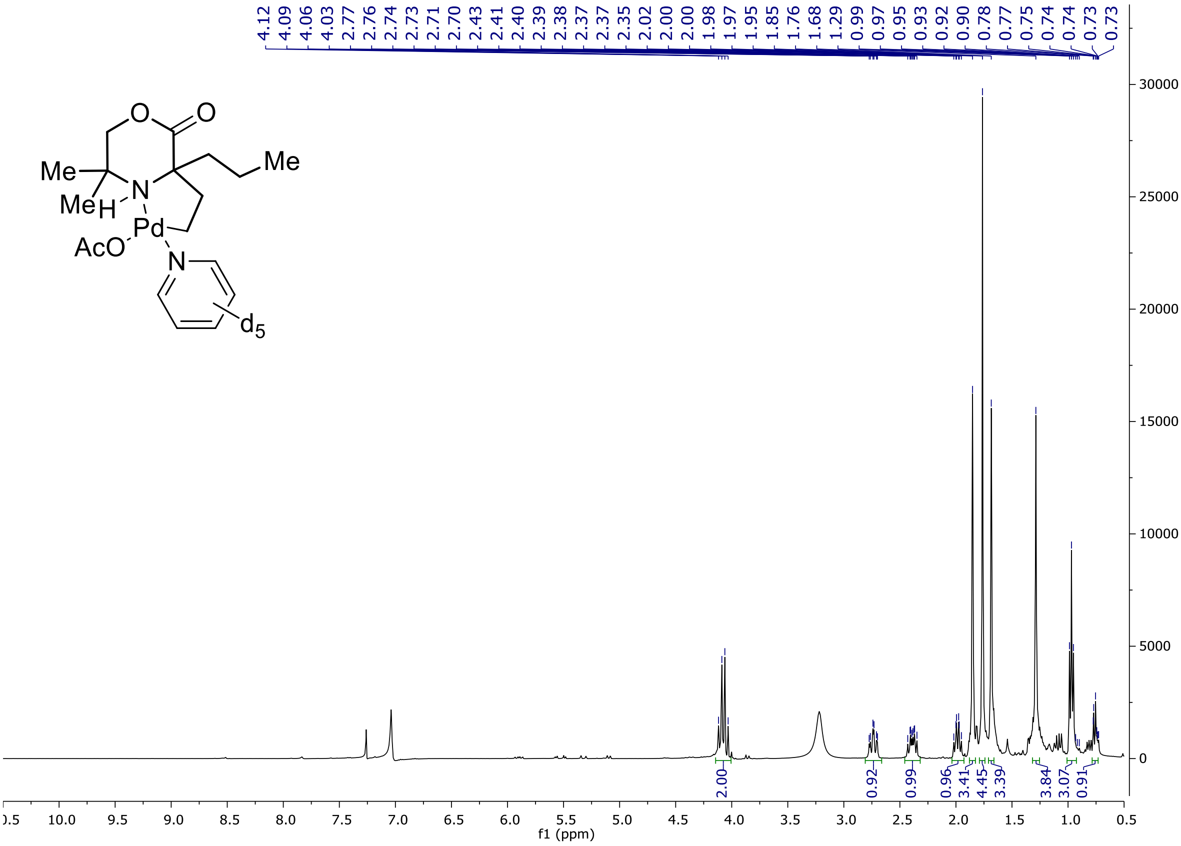

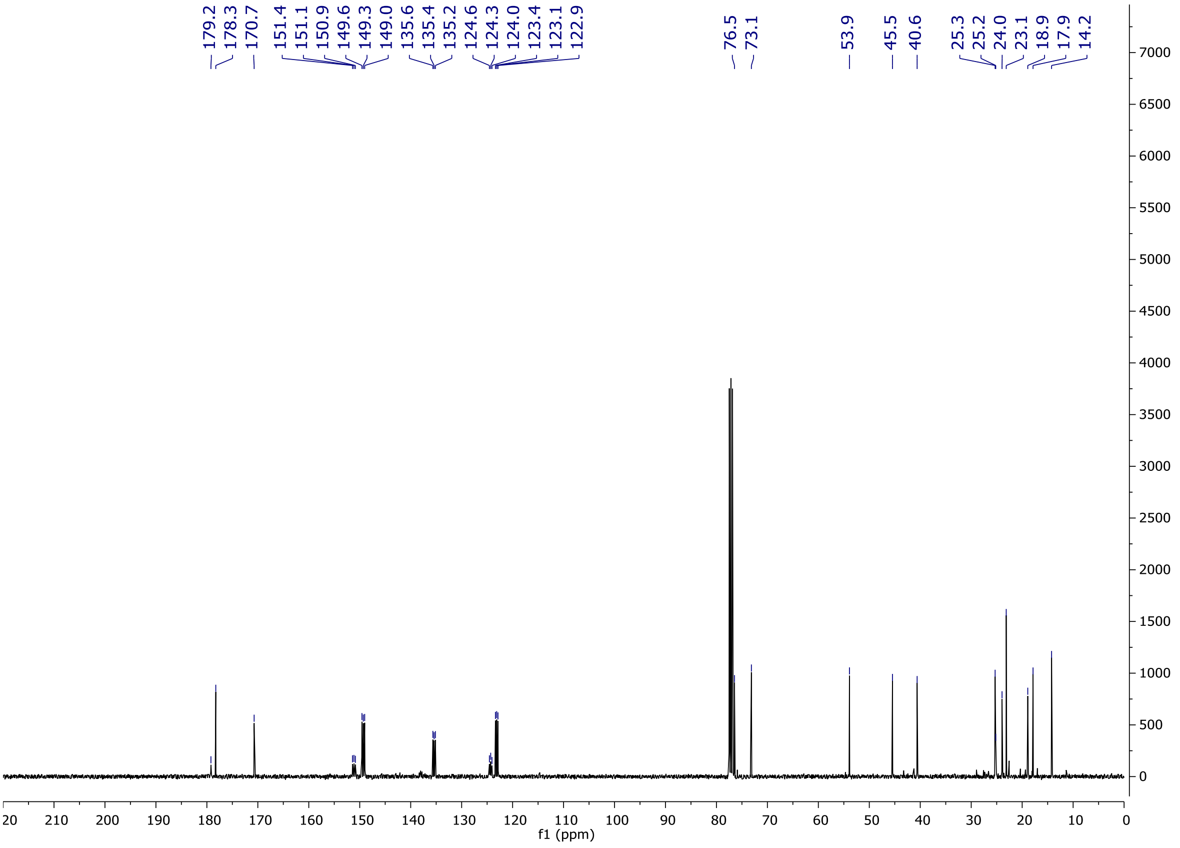

Computational experimental for the acetoxylation of 3,3-diethyl-5,5-dimethylmorpholin-2-one with PIDA

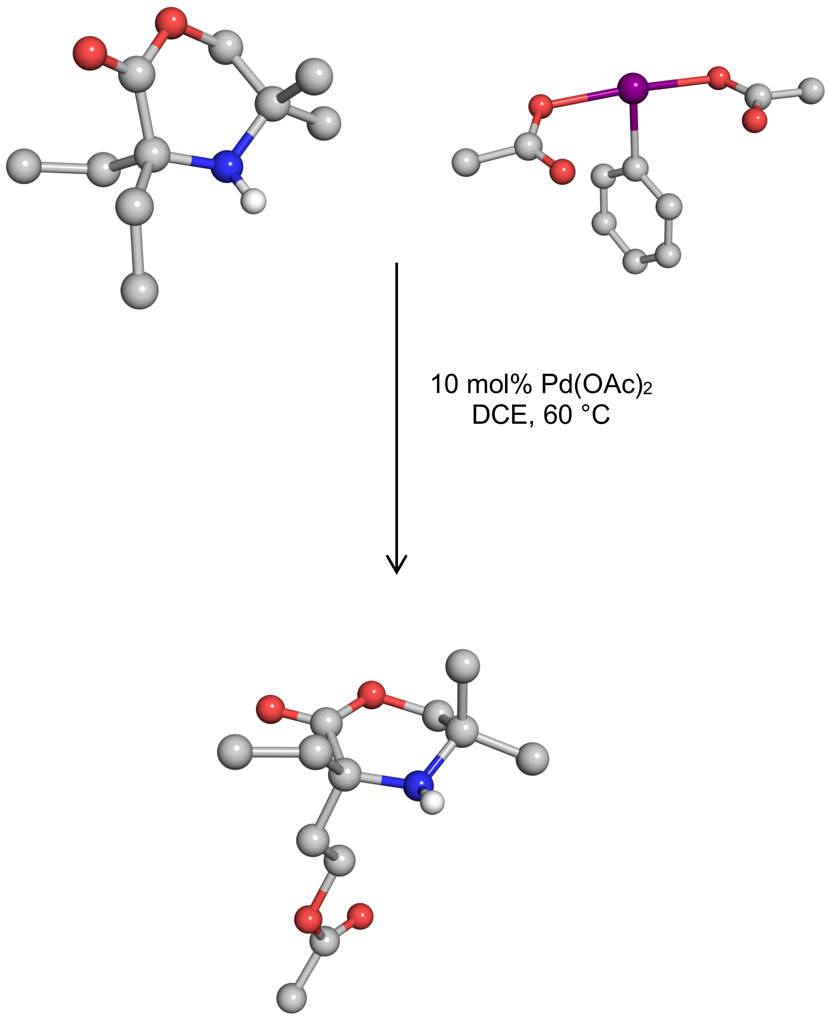

General considerations

Calculations were performed using the Amsterdam Density Functional (ADF) program[1-3] with Zero-Order Regular Approximation (ZORA) scalar relativistic BLYP-D3 exchange correlation (XC) potential, a small frozen core, ‘good’ numerical integration quality and solvent effects were considered using an implicit conductor like screening model (COSMO). This combination of exchange-correlation potential and basis set has been previously benchmarked for palladium catalysis by others[4,5] and also used previously within our group for studying C(sp^3)-H activation reactions of amines with palladium.[6,7]

Proposed intermediates were first optimised using the Universal Forcefield (UFF) molecular mechanics forcefield.[8] The resultant structures were then subjected to DFT geometry optimisation using a TZP basis set for palladium and iodine and a DZP basis set for all other atoms. Vibrational frequency analysis was then performed on structures using a TZ2P basis set for all atoms to confirm that the structures were minima. Transition states were found from DFT minimised intermediates using a series of linear transits employing a TZP basis set for palladium and a DZP basis set for all other atoms. Transition state structures were then confirmed by vibrational frequency analysis, with one negative frequency observed corresponding to the reaction co-ordinate.

Coordinates are listed in angstroms. All enthalpies and Gibbs free energies are given in kcal mol^{-1}, entropy is reported in kcal mol^{-1} K^{-1}.

All species were modelled at 333.15 K at 1 atm in dichloroethane.

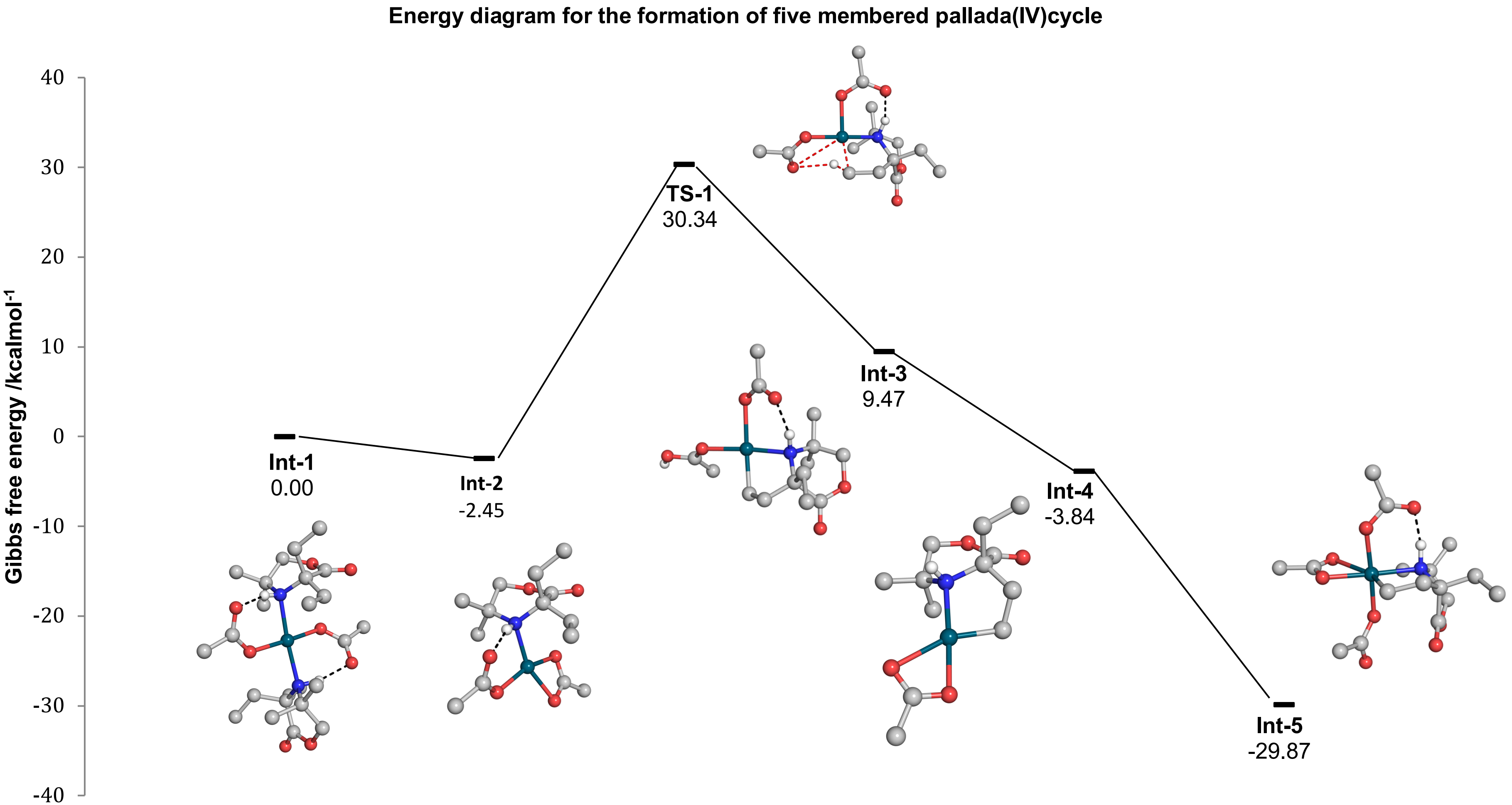

Figure S1: An energy level diagram to show the formation of the pallada(II)cycle. The Gibbs energy for Int-1 has been set to zero and all other energies are relative to Int-1.

Formation of pallada(IV)cycle

3,3-diethyl-5,5-dimethyl-morpholin-2-one

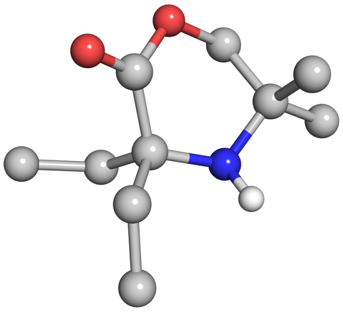

Bonding energy	Internal energy	Entropy	Gibbs energy
-4028.38	183.461	120.778	-3885.16

Atom	X	Y	Z
1 C	-1.613011	0.769633	-0.326136
2 C	-2.148519	-0.559157	0.203817
3 N	-0.970528	-1.360090	0.640226
4 C	0.273665	-1.288557	-0.200708
5 C	0.081506	-0.507013	-1.519559
6 O	-0.770795	0.558069	-1.520698
7 O	0.703387	-0.741168	-2.548948
8 C	1.352224	-0.492946	0.623818
9 C	2.751166	-0.392891	-0.004946
10 C	0.754743	-2.725661	-0.547528
11 C	1.138647	-3.590068	0.667841
12 C	-2.987423	-1.283880	-0.881446
13 C	-3.023217	-0.276671	1.444578
14 H	-1.016720	1.290225	0.433618
15 H	-2.420646	1.422228	-0.672160
16 H	-1.265335	-2.339552	0.692035
17 H	1.401921	-0.986244	1.604933
18 H	0.958455	0.516162	0.811485
19 H	3.409804	0.200978	0.645302
20 H	2.718794	0.092417	-0.990302
21 H	3.209907	-1.382650	-0.132831
22 H	1.610072	-2.653079	-1.230411
23 H	-0.053465	-3.214538	-1.115495
24 H	2.026832	-3.195402	1.179324
25 H	1.369676	-4.611956	0.335428
26 H	0.328192	-3.656500	1.407634
27 H	-2.384185	-1.520750	-1.767632
28 H	-3.840204	-0.667004	-1.200871
29 H	-3.377749	-2.226804	-0.471708
30 H	-2.447116	0.270766	2.202668
31 H	-3.358477	-1.226768	1.884184
32 H	-3.913546	0.308507	1.172997

Acetic acid

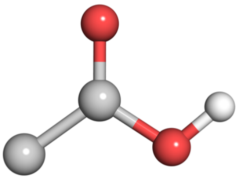

Bonding energy	Internal energy	Entropy	Gibbs energy
-1040.57	40.415	70.079	-1023.50

Atom	X	Y	Z
1 H	4.764653	-4.132255	-0.246616
2 O	3.954873	-3.694904	0.125029
3 C	1.719231	-4.113849	0.818787
4 C	3.000330	-4.669245	0.260994
5 O	3.202703	-5.837370	-0.046265
6 H	1.908379	-3.664055	1.802270
7 H	0.977658	-4.911726	0.909506
8 H	1.340579	-3.322095	0.159077

Palladium acetate

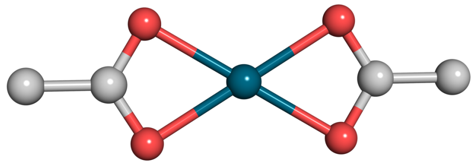

Bonding energy	Internal energy	Entropy	Gibbs energy
-1955.39	69.809	110.805	-1922.50

Atom	X	Y	Z
1 Pd	3.185298	-5.179270	0.194030
2 O	1.355311	-4.115352	0.332930
3 C	0.691206	-5.189826	0.063430
4 O	1.388051	-6.258166	-0.141418
5 O	4.983502	-4.100686	0.529257
6 C	5.679260	-5.170180	0.326051
7 O	5.014997	-6.244158	0.056275
8 C	7.167387	-5.156648	0.399100
9 C	-0.796679	-5.204901	-0.005527
10 H	7.558203	-4.418905	-0.314401
11 H	7.570705	-6.148475	0.170177
12 H	7.476785	-4.851309	1.407670
13 H	-1.199855	-4.209443	0.207368
14 H	-1.183846	-5.930311	0.722536
15 H	-1.107956	-5.529301	-1.007322

PIDA

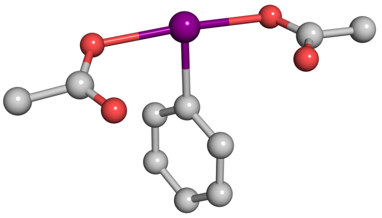

Bonding energy	Internal energy	Entropy	Gibbs energy
-3478.14	128.644	147.003	-3398.47

Atom	X	Y	Z
1 C	0.058859	-4.355179	2.535183

2 C	1.315069	-3.793502	2.801930
3 C	1.956382	-2.997166	1.843080
4 C	1.297872	-2.805843	0.634324
5 C	0.052312	-3.343883	0.325018
6 C	-0.570484	-4.130056	1.305294
7 I	2.245774	-1.507105	-0.884417
8 O	0.300049	-0.229164	-0.697929
9 C	0.162563	0.372282	0.481355
10 O	0.991851	0.372524	1.399019
11 O	3.885318	-3.165510	-1.108676
12 C	4.851144	-3.090576	-0.204604
13 O	4.937232	-2.249326	0.700923
14 C	-1.200169	1.045597	0.622260
15 H	-1.547911	-4.565558	1.095523
16 H	2.929324	-2.555720	2.038139
17 H	-0.431006	-4.969708	3.288759
18 H	1.805089	-3.963060	3.760800
19 C	5.869125	-4.218845	-0.360776
20 H	-0.431755	-3.158629	-0.630069
21 H	-1.627399	1.320153	-0.347748
22 H	-1.103963	1.928626	1.264665
23 H	-1.875065	0.329327	1.111781
24 H	6.840486	-3.886201	0.023436
25 H	5.958979	-4.548289	-1.402678
26 H	5.522845	-5.071099	0.243932

Phenyl iodide

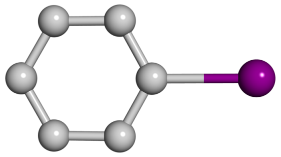

Bonding energy	Internal energy	Entropy	Gibbs energy
-1605.59	59.121	84.812	-1574.72

Atom	X	Y	Z
1 C	-0.299348	-4.967409	2.000547
2 C	1.025338	-5.224708	1.629325
3 C	1.799798	-4.228136	1.017846
4 C	1.221267	-2.976734	0.788262
5 C	-0.098713	-2.697530	1.150317
6 C	-0.857097	-3.706546	1.760497
7 I	2.415450	-1.394134	-0.170406
8 H	1.466290	-6.204394	1.813212
9 H	-0.537792	-1.719503	0.966351
10 H	-1.887641	-3.495952	2.045704
11 H	2.828729	-4.433358	0.731351
12 H	-0.894453	-5.746756	2.475081

Int-1: *Bisamine palladium diacetate*

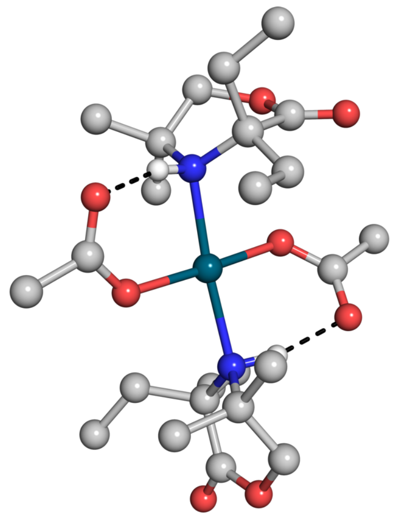

Bonding energy	Internal energy	Entropy	Gibbs energy
-10058.01	443.547	252.485	-9698.58

Atom	X	Y	Z
1 C	-1.398151	1.571691	-1.400865
2 C	-1.443880	1.181147	0.088476
3 N	-0.509402	-0.025102	0.350091
4 C	0.403963	-0.455101	-0.802557
5 C	-0.430129	-0.537935	-2.097628
6 O	-1.391632	0.407707	-2.294550
7 O	-0.254781	-1.376774	-2.967046
8 C	1.561365	0.594266	-0.973718
9 C	2.546401	0.281270	-2.113791
10 C	1.016625	-1.862336	-0.527304
11 C	2.030200	-1.948813	0.626358
12 C	-2.903335	0.834431	0.455234
13 C	-0.965785	2.377749	0.935615
14 H	-0.535757	2.197920	-1.647299
15 H	-2.305999	2.119664	-1.669577
16 H	0.115656	0.242447	1.145114
17 H	2.091979	0.639823	-0.012525
18 H	1.148916	1.592947	-1.135683
19 H	2.026890	0.188953	-3.077644
20 H	3.098713	-0.650238	-1.936928
21 H	3.277023	1.097734	-2.197238
22 H	1.493407	-2.188062	-1.459313
23 H	0.193469	-2.563700	-0.353738
24 H	1.630961	-1.544843	1.563046
25 H	2.961111	-1.412394	0.398427
26 H	2.288043	-3.003463	0.795500
27 H	-3.295544	0.048454	-0.199383
28 H	-3.521802	1.736293	0.339859
29 H	-2.974465	0.506342	1.499436
30 H	0.086088	2.618600	0.735732
31 H	-1.069630	2.151160	2.003679
32 H	-1.578309	3.260608	0.705079
33 Pd	-1.477170	-1.690227	1.371380
34 O	-2.249521	-4.400473	-0.390386
35 O	-2.277755	-2.127159	-0.521992

36 C	-2.447553	-3.325638	-1.007693
37 C	-2.968844	-3.321304	-2.436958
38 O	-0.799970	-1.120027	3.272089
39 C	0.101081	-0.229566	3.573946
40 C	0.243762	-0.016449	5.078019
41 O	0.794387	0.435048	2.766991
42 H	-4.040367	-3.074047	-2.416895
43 H	-2.840997	-4.311302	-2.888139
44 H	-2.453260	-2.557853	-3.029607
45 H	0.200375	-0.970598	5.616002
46 H	-0.596440	0.601836	5.424484
47 H	1.182946	0.501812	5.300405
48 N	-2.383982	-3.485197	2.270586
49 H	-2.450591	-3.951697	1.339751
50 C	-3.802933	-3.351184	2.818823
51 C	-4.283694	-4.655072	3.506643
52 O	-3.510509	-5.776990	3.518447
53 C	-2.149806	-5.785964	2.952093
54 C	-1.475194	-4.417124	3.065294
55 O	-5.390713	-4.736994	4.018898
56 C	-3.851901	-2.098883	3.766342
57 C	-4.845085	-2.078991	4.939542
58 C	-4.726480	-3.154748	1.561807
59 C	-6.187960	-2.757520	1.816099
60 C	-1.298486	-4.009165	4.540130
61 C	-0.103569	-4.488753	2.366196
62 H	-2.228313	-6.106165	1.904590
63 H	-1.617379	-6.547870	3.532028
64 H	-4.043897	-1.237352	3.109658
65 H	-2.846794	-1.938335	4.160734
66 H	-4.769910	-1.103512	5.442884
67 H	-4.610406	-2.857664	5.677181
68 H	-5.881349	-2.227288	4.619683
69 H	-4.249213	-2.369146	0.961766
70 H	-4.688888	-4.076559	0.961081
71 H	-6.260075	-1.789664	2.328754
72 H	-6.715985	-3.506796	2.415500
73 H	-6.702058	-2.660179	0.848814
74 H	-0.608690	-4.720432	5.014538
75 H	-2.237272	-4.035307	5.105257
76 H	-0.873314	-3.004460	4.601627
77 H	0.499362	-5.287077	2.820896
78 H	-0.211859	-4.694493	1.294062
79 H	0.428337	-3.540133	2.494723

Int-2: *Monoamine palladium diacetate*

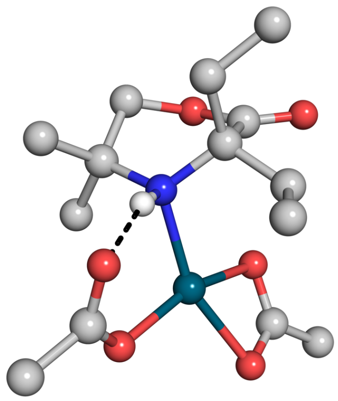

Bonding energy	Internal energy	Entropy	Gibbs energy
-6008.93	256.849	191.464	-5815.87

Atom	X	Y	Z
1 C	-2.221701	-0.089319	-1.403774
2 C	-2.162371	0.203198	0.117401
3 N	-0.927863	-0.495070	0.725333
4 C	0.185224	-0.945102	-0.232112
5 C	-0.501894	-1.822280	-1.303870
6 O	-1.764221	-1.448472	-1.701896
7 O	0.033363	-2.878699	-1.825318
8 C	0.898338	0.292315	-0.892903
9 C	2.051134	-0.080430	-1.844202
10 C	1.211665	-1.843234	0.524414
11 C	2.095925	-1.233477	1.625916
12 C	-3.485442	-0.339251	0.792799
13 C	-2.060758	1.735976	0.351004
14 H	-1.624664	0.624323	-1.990614
15 H	-3.249595	-0.045259	-1.778507
16 H	-0.498826	0.167013	1.408257
17 H	1.265792	0.930415	-0.074272
18 H	0.178748	0.901372	-1.446811
19 H	1.717066	-0.776093	-2.625392
20 H	2.901353	-0.538375	-1.320003
21 H	2.423288	0.829326	-2.341724
22 H	1.856887	-2.264765	-0.254227
23 H	0.650615	-2.699817	0.935875
24 H	1.505211	-0.776823	2.425029
25 H	2.783564	-0.476523	1.231933
26 H	2.710249	-2.030553	2.075269
27 H	-3.571504	-1.415560	0.607552
28 H	-4.353601	0.176595	0.357958
29 H	-3.492532	-0.151764	1.872378
30 H	-1.142040	2.160588	-0.055946
31 H	-2.088241	1.949219	1.428614
32 H	-2.912274	2.239365	-0.128990
33 Pd	-1.407832	-1.994188	2.128459
34 O	-1.535961	-4.000295	3.018848
35 O	-1.710088	-3.723662	0.862222
36 C	-1.710519	-4.513758	1.879285
37 C	-1.857787	-5.992654	1.729199
38 O	-1.549818	-0.746687	3.800745
39 C	-0.984685	0.429418	3.903438
40 C	-1.405616	1.219134	5.141414

41 O	-0.164080	0.928086	3.043677
42 H	-1.829039	-6.322332	0.683109
43 H	-2.736489	-6.328881	2.291706
44 H	-1.022410	-6.447757	2.297119
45 H	-0.976971	2.229495	5.110565
46 H	-1.059941	0.702206	6.047603
47 H	-2.501228	1.281252	5.199474

TS-1: *C-H activation of monoamine palladium diacetate*

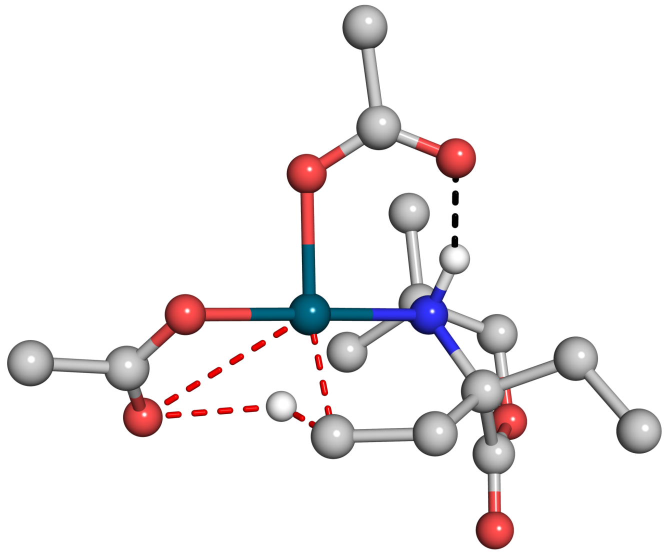

Imaginary frequency at -83 cm^{-1}

Bonding energy	Internal energy	Entropy	Gibbs energy
-5976.63	253.480	179.901	-5783.08

Atom	X	Y	Z
1 C	-1.849280	1.052207	-1.252642
2 C	-2.239224	0.177752	-0.054521
3 N	-0.946728	-0.380468	0.530146
4 C	0.200514	-0.649751	-0.452012
5 C	-0.296109	-0.700242	-1.908204
6 O	-1.199859	0.238860	-2.299356
7 O	0.149848	-1.476581	-2.738389
8 C	1.247147	0.511445	-0.350209
9 C	2.503939	0.309483	-1.212835
10 C	0.827835	-1.987023	-0.020386
11 C	-0.243502	-3.044063	0.387069
12 C	-3.180353	-0.956365	-0.511037
13 C	-2.921823	1.050922	1.012911
14 H	-1.179338	1.869852	-0.963510
15 H	-2.737129	1.467339	-1.739140
16 H	-0.589576	0.269927	1.270683
17 H	1.524258	0.587921	0.711846
18 H	0.767226	1.459605	-0.623655
19 H	2.249855	0.205416	-2.276160
20 H	3.066690	-0.580868	-0.905432
21 H	3.162328	1.181714	-1.103126
22 H	1.487730	-1.787342	0.834744
23 H	1.447244	-2.403351	-0.824425
24 H	-0.602865	-3.477466	-0.578645

25 H	-1.531989	-3.067976	0.558857
26 H	0.225792	-3.909849	0.874532
27 H	-2.687641	-1.657010	-1.196052
28 H	-4.041514	-0.522928	-1.037257
29 H	-3.550993	-1.516630	0.355242
30 H	-2.273240	1.878677	1.327606
31 H	-3.169415	0.444708	1.892803
32 H	-3.853892	1.464507	0.605909
33 Pd	-1.139116	-2.106850	1.798956
34 O	-1.345207	-3.804724	3.016600
35 O	-2.551166	-4.784345	1.346284
36 C	-2.058952	-4.775968	2.495736
37 C	-2.278635	-5.951617	3.442234
38 O	-1.541713	-0.935475	3.542352
39 C	-0.982870	0.227388	3.726397
40 C	-1.375675	0.924434	5.021689
41 O	-0.203685	0.804680	2.922486
42 H	-2.245521	-6.887424	2.873014
43 H	-3.281986	-5.851003	3.879407
44 H	-1.540713	-5.969988	4.251110
45 H	-2.124844	1.693109	4.781923
46 H	-0.501158	1.429635	5.449342
47 H	-1.806016	0.226999	5.748027

Int-3: *Five membered amino palladacycle with bound acetic acid*

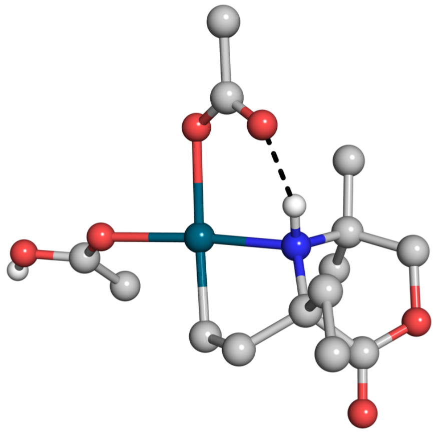

Bonding energy	Internal energy	Entropy	Gibbs energy
-5998.91	255.190	180.795	-5803.95

Atom	X	Y	Z
1 C	-0.608667	-0.374066	-2.843682
2 C	-1.224173	-1.351613	-1.836514
3 N	-0.969034	-0.774503	-0.450318
4 C	0.414670	-0.106951	-0.238933
5 C	1.369781	-0.326177	-1.429446
6 O	0.855225	-0.312947	-2.689633
7 O	2.583550	-0.412109	-1.303500
8 C	0.177179	1.437305	-0.131263
9 C	1.432752	2.264267	0.189272
10 C	1.021277	-0.674304	1.068986
11 C	0.608258	-2.131895	1.290450
12 C	-0.599524	-2.753839	-1.978863
13 C	-2.742133	-1.418162	-2.091863
14 O	-3.291513	0.570328	0.435984

15 Pd	-1.447577	-2.014995	1.203202
16 O	-1.776962	-3.265994	2.980478
17 O	-1.761803	-5.038421	4.285447
18 C	-1.485786	-4.463513	3.093929
19 C	-0.870357	-5.328139	2.034706
20 O	-3.691063	-1.563828	1.129979
21 C	-4.059616	-0.377792	0.784589
22 C	-5.571618	-0.137663	0.745446
23 H	-1.022174	0.635356	-2.728279
24 H	-0.762273	-0.713600	-3.872850
25 H	-0.577344	1.575445	0.658159
26 H	-0.271558	1.799640	-1.067357
27 H	1.871375	1.973471	1.152474
28 H	1.163478	3.327845	0.245557
29 H	2.201620	2.146326	-0.585506
30 H	0.653202	-0.059482	1.903218
31 H	2.116344	-0.562485	1.052250
32 H	0.918058	-2.511823	2.274904
33 H	0.971904	-2.805965	0.500587
34 H	0.474778	-2.760649	-1.761909
35 H	-1.092374	-3.448582	-1.288485
36 H	-0.739378	-3.113666	-3.007222
37 H	-3.223463	-2.061732	-1.347060
38 H	-2.922468	-1.837884	-3.090225
39 H	-3.200700	-0.422774	-2.034877
40 H	-1.500717	-5.991096	4.269546
41 H	0.045526	-5.796542	2.419753
42 H	-0.640875	-4.734451	1.147275
43 H	-1.577340	-6.127540	1.770552
44 H	-5.935973	-0.419355	-0.254573
45 H	-5.802104	0.922571	0.905063
46 H	-6.095504	-0.755931	1.484168
47 H	-1.712578	-0.061886	-0.259678

Int-4: *Five membered amino palladacycle with κ^2 acetate*

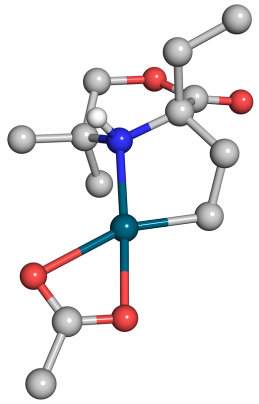

Bonding energy	Internal energy	Entropy	Gibbs energy
-4955.32	215.018	160.473	-4793.76

Atom	X	Y	Z
1 C	-0.346298	-0.240614	-2.911964
2 C	-1.152239	-1.121094	-1.952814
3 N	-0.914306	-0.600019	-0.535277
4 C	0.507874	-0.039433	-0.246152

5 C	1.511260	-0.426462	-1.349063
6 O	1.096849	-0.377022	-2.645537
7 O	2.685707	-0.676154	-1.124010
8 C	0.423953	1.522949	-0.232386
9 C	1.732456	2.230271	0.154673
10 C	0.945707	-0.577788	1.133214
11 C	0.451003	-2.001918	1.358067
12 C	-0.710621	-2.593290	-2.073728
13 C	-2.651883	-0.974788	-2.262032
14 O	-3.944146	-2.437384	1.238840
15 Pd	-1.570807	-1.950010	1.020103
16 H	-4.609273	-5.054986	2.387055
17 H	-5.552555	-3.635895	2.902945
18 H	-4.227558	-4.275874	3.939718
19 H	-1.547653	0.205279	-0.423077
20 O	-2.318147	-3.283738	2.524908
21 C	-3.568046	-3.203439	2.180644
22 C	-4.554655	-4.089119	2.910322
23 H	-0.627778	0.816393	-2.832521
24 H	-0.468056	-0.568378	-3.949001
25 H	-0.366828	1.781113	0.490518
26 H	0.091910	1.883391	-1.215511
27 H	2.052914	1.957988	1.168630
28 H	1.580783	3.317768	0.125794
29 H	2.544702	1.979029	-0.540524
30 H	0.527304	0.085511	1.903840
31 H	2.041759	-0.523760	1.222247
32 H	0.612182	-2.356216	2.385067
33 H	0.863539	-2.723667	0.635940
34 H	0.332577	-2.742112	-1.772165
35 H	-1.342216	-3.227942	-1.440105
36 H	-0.813233	-2.916815	-3.118589
37 H	-3.245401	-1.542156	-1.533142
38 H	-2.864546	-1.369850	-3.263800
39 H	-2.963008	0.079105	-2.228485

Int-5: *Five membered amino pallada(IV)cycle triacetate*

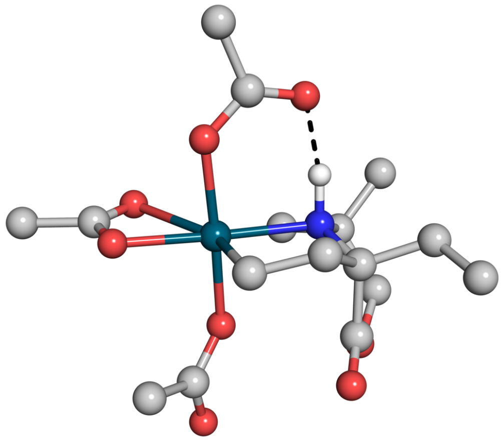

Bonding energy	Internal energy	Entropy	Gibbs energy
-6859.28	283.927	204.652	-6643.53

Atom	X	Y	Z

1 C	-0.438346	-1.102940	-2.713924
2 C	-1.485051	-0.595124	-1.686302
3 N	-0.870401	-0.627213	-0.287933
4 C	0.644885	-0.600940	-0.197032
5 C	1.171728	-1.796289	-1.009488
6 O	0.542241	-2.065919	-2.179796
7 O	2.104300	-2.495183	-0.648868
8 C	1.252129	0.734676	-0.742581
9 C	2.777996	0.841556	-0.572417
10 C	0.968915	-0.736871	1.306617
11 C	0.344149	-1.951572	1.932849
12 C	-2.751431	-1.472674	-1.731442
13 C	-1.881099	0.862049	-2.028504
14 O	-3.983986	-2.970540	1.080578
15 Pd	-1.600336	-2.114634	1.149946
16 H	-4.360745	-5.596569	2.302936
17 H	-5.512942	-4.310612	2.736349
18 H	-4.159723	-4.734307	3.847704
19 H	-1.262044	0.176410	0.276969
20 O	-2.368041	-3.497930	2.536471
21 C	-3.591038	-3.623656	2.078700
22 C	-4.464512	-4.622256	2.801420
23 H	0.126757	-0.275539	-3.156273
24 H	-0.936944	-1.651854	-3.517185
25 H	0.748566	1.556730	-0.211602
26 H	1.016473	0.845591	-1.803663
27 H	3.068095	0.930482	0.481902
28 H	3.137520	1.735898	-1.098572
29 H	3.288648	-0.032945	-0.997592
30 H	0.635888	0.180380	1.810911
31 H	2.057428	-0.823503	1.444502
32 H	0.156738	-1.889236	3.008234
33 H	0.786262	-2.896023	1.619394
34 H	-2.506149	-2.533361	-1.637251
35 H	-3.452230	-1.188451	-0.935928
36 H	-3.252559	-1.307948	-2.694885
37 H	-2.680259	1.199080	-1.356205
38 H	-2.259007	0.897467	-3.060273
39 H	-1.038834	1.555547	-1.943604
40 O	-2.249743	-0.672604	2.552600
41 C	-2.402927	0.609225	2.325831
42 O	-2.063650	1.230023	1.286302
43 C	-3.098375	1.299964	3.490015
44 O	-1.069797	-3.588939	-0.221409
45 C	-0.555791	-4.803571	-0.095103
46 C	-0.083272	-5.330891	1.243468
47 O	-0.451317	-5.488103	-1.127793
48 H	-2.677754	0.966252	4.445805
49 H	-4.161735	1.023303	3.478385
50 H	-3.005516	2.385896	3.388866
51 H	0.967941	-5.042842	1.385263
52 H	-0.125622	-6.426267	1.229039
53 H	-0.670794	-4.942262	2.078941

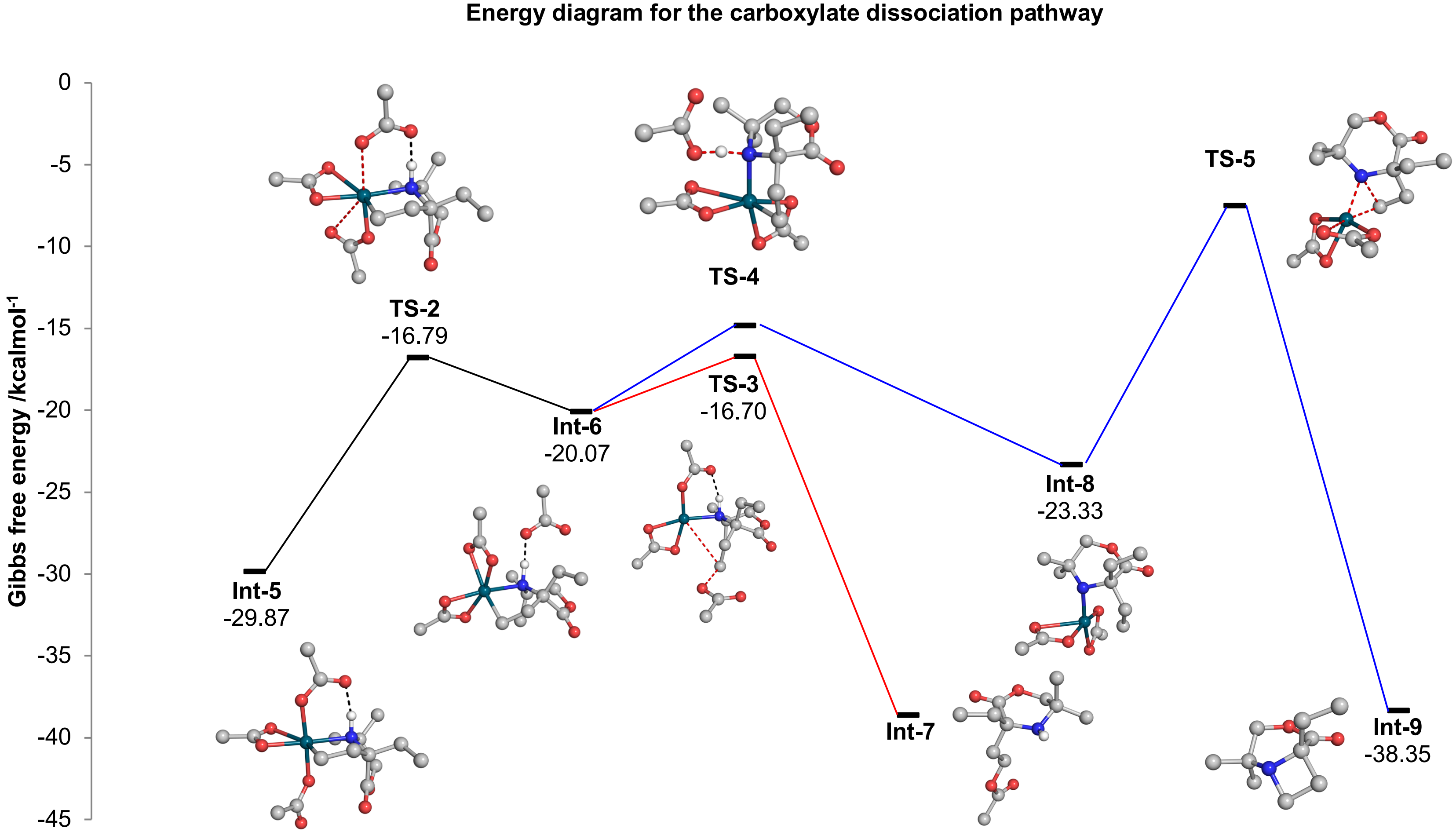

Figure S2: An energy level diagram to show the formation of the pallada(II)cycle. The Gibbs energy for Int-1 has been set to zero and all other energies are relative to Int-1. Red lines indicate Pathway 1A: dissociated carboxylate attack on the palladated carbon pathway. Blue lines indicate Pathway 1B: acetate N–H deprotonation of the amine.

Pathway 1: Carboxylate dissociation pathway

TS-2: *Acetate dissociation from five membered amino pallada(IV)cycle triacetate*

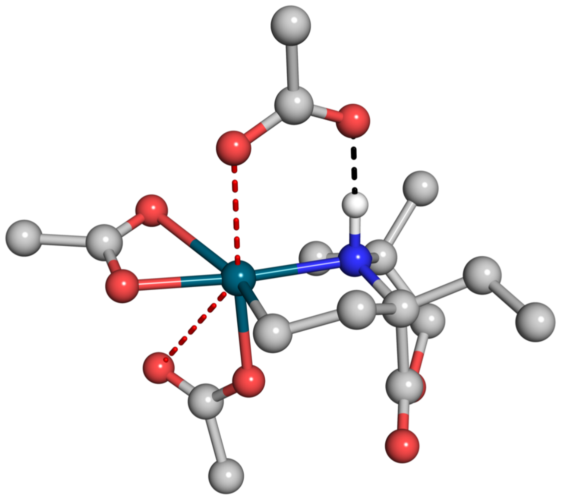

Imaginary frequency at -88 cm^{-1}

Bonding energy	Internal energy	Entropy	Gibbs energy
-6844.37	282.433	205.671	-6630.46

Atom	X	Y	Z
1 C	-0.214229	-1.279053	-2.620588
2 C	-1.404064	-0.978883	-1.677030
3 N	-0.883720	-0.810141	-0.246357
4 C	0.601880	-0.515824	-0.082693
5 C	1.388260	-1.612079	-0.821183
6 O	0.866666	-2.059832	-1.994221
7 O	2.429288	-2.088357	-0.401324
8 C	0.975864	0.903201	-0.629886
9 C	2.452411	1.285037	-0.423759
10 C	0.871186	-0.556918	1.441158
11 C	0.405846	-1.827706	2.095328
12 C	-2.435518	-2.123698	-1.742679
13 C	-2.090269	0.336815	-2.113579
14 O	-3.838994	-3.025605	1.342303
15 Pd	-1.460678	-2.276662	1.234372
16 H	-4.100274	-5.665627	2.645668
17 H	-5.319296	-4.404632	2.981886
18 H	-3.974073	-4.706905	4.140234
19 H	-1.409740	-0.004116	0.220151
20 O	-2.157635	-3.587865	2.707320
21 C	-3.409918	-3.690150	2.318770
22 C	-4.261263	-4.670038	3.083057
23 H	0.229211	-0.362640	-3.022514
24 H	-0.545505	-1.899019	-3.458205
25 H	0.316718	1.620173	-0.119086
26 H	0.747549	0.966353	-1.697215
27 H	2.705530	1.383953	0.639131
28 H	2.643058	2.252658	-0.907286
29 H	3.125481	0.541092	-0.870423
30 H	0.383135	0.315606	1.893875

31 H	1.954969	-0.477955	1.620665
32 H	0.133634	-1.745333	3.151598
33 H	0.980103	-2.724063	1.848591
34 H	-1.963959	-3.099440	-1.603684
35 H	-3.225053	-1.995991	-0.992158
36 H	-2.910027	-2.110337	-2.733367
37 H	-2.983897	0.513848	-1.501384
38 H	-2.398359	0.249793	-3.164457
39 H	-1.425706	1.201430	-2.015323
40 O	-2.335150	-0.212499	2.839553
41 C	-2.432358	0.937026	2.269956
42 O	-2.125551	1.208411	1.072569
43 C	-2.974887	2.063556	3.152245
44 O	-0.392029	-3.729673	0.211866
45 C	-0.924393	-4.908435	-0.087889
46 C	0.126906	-5.761689	-0.812438
47 O	-2.065168	-5.302046	0.143929
48 H	-3.128588	1.739689	4.186663
49 H	-3.930299	2.403825	2.730031
50 H	-2.273069	2.907280	3.123691
51 H	-0.388517	-6.397768	-1.540628
52 H	0.615998	-6.397579	-0.062513
53 H	0.884068	-5.143002	-1.303553

Int-6: *Five membered amino pallada(IV)cycle diacetate with dissociated acetate*

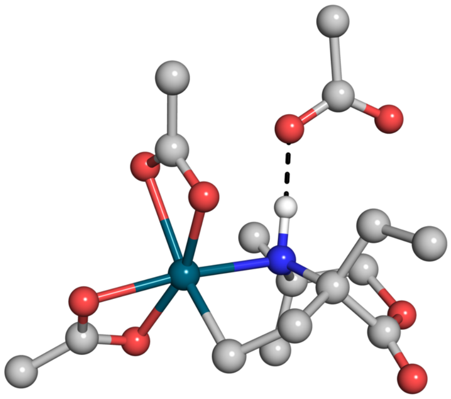

Low intensity, negative frequency at -78 cm^{-1} corresponding to acetate methyl rotation.

Bonding energy	Internal energy	Entropy	Gibbs energy
-6848.44	283.195	205.593	-6633.74

Atom	X	Y	Z
1 C	0.501155	0.153966	-2.271620
2 C	-0.298186	-1.144942	-2.064995
3 N	-0.619803	-1.153500	-0.568341
4 C	0.583967	-0.833614	0.368083
5 C	1.893816	-0.590343	-0.425346
6 O	1.815810	0.049674	-1.612762
7 O	2.992992	-0.847535	0.038519
8 C	0.278078	0.513318	1.113637
9 C	1.347736	0.944469	2.131284
10 C	0.742924	-1.989055	1.388828
11 C	0.280120	-3.313155	0.845460
12 C	0.528739	-2.361887	-2.531008

13 C	-1.621335	-1.057023	-2.848805
14 O	-2.473926	-4.740056	0.681378
15 Pd	-1.626478	-2.869385	0.143884
16 H	-4.182969	-0.559900	3.012120
17 H	-5.474937	-0.883642	1.802086
18 H	-4.309388	0.446545	1.543831
19 C	-2.479912	-6.637084	-0.848365
20 H	-3.425774	-6.595891	-1.403954
21 H	-2.621292	-7.260634	0.038550
22 H	-1.709694	-7.055633	-1.502052
23 H	-0.034814	1.029661	-1.888232
24 H	0.734669	0.288892	-3.329713
25 H	-0.684196	0.368237	1.614261
26 H	0.128714	1.303602	0.372435
27 H	1.500612	0.194013	2.914622
28 H	1.019556	1.870737	2.616303
29 H	2.314274	1.139653	1.655095
30 H	0.177927	-1.742213	2.292195
31 H	1.795515	-2.077011	1.691466
32 H	0.125743	-4.092535	1.593168
33 H	0.800183	-3.683831	-0.038012
34 H	1.417950	-2.541318	-1.921224
35 H	-0.071876	-3.269638	-2.538205
36 H	0.870122	-2.169921	-3.553192
37 H	-2.235703	-1.944285	-2.663471
38 H	-1.400340	-1.018890	-3.920141
39 H	-2.193796	-0.171253	-2.564051
40 O	-2.307240	-1.645745	1.654781
41 C	-3.515294	-1.448902	1.169890
42 O	-3.819060	-1.992940	0.075183
43 C	-4.439790	-0.563825	1.949550
44 O	-1.434721	-4.473221	-1.225814
45 C	-2.113433	-5.249723	-0.445683
46 H	-1.351003	-0.366426	-0.437665
47 O	-2.499456	0.763215	-0.297317
48 C	-2.382237	2.015080	-0.542933
49 C	-3.545832	2.902584	-0.067877
50 O	-1.388592	2.561904	-1.107184
51 H	-4.505195	2.395489	-0.216239
52 H	-3.545367	3.868051	-0.582508
53 H	-3.426333	3.082116	1.009261

Pathway 1A: Dissociated acetate attack on palladated carbon

TS-3: *Dissociated acetate attack on palladated carbon of five membered amino pallada(IV)cycle diacetate with dissociated acetate*

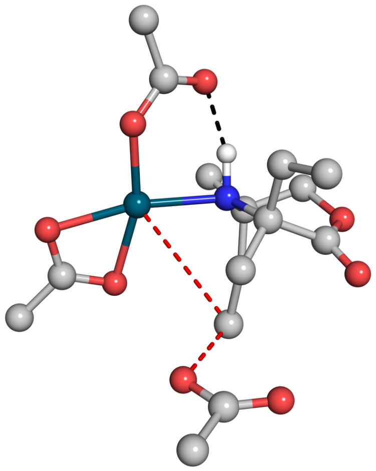

Imaginary frequency at -156 cm^{-1}

Bonding energy	Internal energy	Entropy	Gibbs energy
-6843.86	283.233	209.342	-6630.37

Atom	X	Y	Z
1 C	0.431296	0.545037	-2.282656
2 C	-0.204469	-0.834839	-2.061591
3 N	-0.261404	-0.993547	-0.545023
4 C	1.058426	-0.748129	0.194948
5 C	2.163665	-0.136182	-0.702170
6 O	1.822828	0.583639	-1.798141
7 O	3.341963	-0.232697	-0.398918
8 C	0.735835	0.304999	1.316172
9 C	1.892156	0.726840	2.233245
10 C	1.613570	-2.047382	0.878280
11 C	2.035586	-3.118547	-0.060127
12 C	0.602582	-1.949711	-2.745060
13 C	-1.645519	-0.800508	-2.606845
14 O	-2.519090	-4.460290	0.631040
15 Pd	-1.504444	-2.583798	0.206403
16 H	-3.639648	-0.036303	3.338602
17 H	-4.693151	0.505415	2.007181
18 H	-3.389723	1.574932	2.574022
19 C	-1.958548	-6.486810	-0.591289
20 H	-2.662007	-6.579175	-1.431139
21 H	-2.366330	-7.029053	0.269001
22 H	-0.997198	-6.915461	-0.897417
23 H	-0.135835	1.334310	-1.771864
24 H	0.508193	0.780051	-3.348955
25 H	-0.070453	-0.132584	1.921228
26 H	0.318585	1.192193	0.817154
27 H	2.253111	-0.107472	2.847383
28 H	1.530105	1.508258	2.915749
29 H	2.738426	1.132781	1.666239
30 H	0.825873	-2.416092	1.548325
31 H	2.485912	-1.752481	1.467433
32 H	1.287607	-3.730479	-0.553821
33 H	3.004863	-3.023599	-0.543725
34 H	1.650077	-1.979883	-2.422546
35 H	0.143331	-2.924922	-2.558468
36 H	0.606862	-1.768559	-3.828359

37 H	-2.122170	-1.780517	-2.477045
38 H	-1.620449	-0.572404	-3.680709
39 H	-2.251912	-0.044606	-2.094196
40 O	-2.436588	-1.326048	1.579505
41 C	-2.745433	-0.072367	1.351441
42 O	-2.347840	0.611082	0.380376
43 C	-3.666905	0.528407	2.401283
44 O	-0.929626	-4.314356	-0.896472
45 C	-1.796999	-5.038748	-0.263320
46 H	-0.927013	-0.243771	-0.238344
47 O	2.503232	-4.691636	1.252905
48 C	3.699646	-4.464154	1.762123
49 C	4.093085	-5.512640	2.805643
50 O	4.448860	-3.517248	1.482705
51 H	4.006698	-5.050410	3.798723
52 H	5.141170	-5.792132	2.646249
53 H	3.455688	-6.401902	2.769482

Int-7: *2-(3-ethyl-5,5-dimethyl-2-oxomorpholin-3-yl)ethyl acetate*

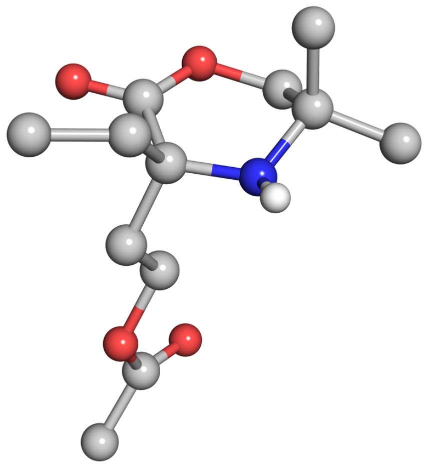

Bonding energy	Internal energy	Entropy	Gibbs energy
-4892.45	212.483	149.594	-4729.80

Atom	X	Y	Z
1 N	3.889856	-4.040944	-1.522358
2 C	2.690354	-3.235605	-1.852456
3 C	3.232857	-1.894905	-2.337369
4 O	4.085615	-2.054490	-3.536850
5 C	4.942247	-3.107618	-3.628191
6 C	4.917611	-4.246310	-2.583239
7 O	5.732171	-3.116657	-4.564223
8 C	6.315082	-4.251158	-1.889260
9 C	6.592076	-2.961231	-1.127314
10 O	7.917349	-3.122597	-0.496425
11 C	8.361398	-2.053848	0.223379
12 C	9.717677	-2.333796	0.821336
13 O	7.727593	-1.011575	0.350871
14 C	4.672866	-5.621377	-3.310568
15 C	5.770953	-6.174090	-4.234373
16 C	1.914784	-3.006734	-0.537631
17 C	1.748571	-3.861624	-2.915792

18 H	3.601846	-4.959982	-1.168829
19 H	3.844155	-1.413825	-1.563283
20 H	2.436438	-1.217028	-2.660286
21 H	7.098313	-4.400532	-2.641776
22 H	6.337445	-5.102189	-1.191232
23 H	5.844942	-2.783830	-0.347214
24 H	6.631553	-2.087436	-1.792475
25 H	10.046124	-1.472762	1.409586
26 H	10.440415	-2.535104	0.019867
27 H	9.668444	-3.226390	1.458189
28 H	3.729895	-5.545460	-3.867446
29 H	4.498646	-6.345316	-2.497855
30 H	5.989053	-5.481775	-5.055218
31 H	5.432654	-7.128698	-4.663287
32 H	6.703527	-6.367428	-3.687312
33 H	2.569627	-2.563190	0.224242
34 H	1.537765	-3.967189	-0.156509
35 H	1.053470	-2.345067	-0.703500
36 H	2.231311	-3.929949	-3.898357
37 H	0.839402	-3.252700	-3.029328
38 H	1.454622	-4.872945	-2.602205

Pathway 1B: N–H deprotonation of the amine by dissociated acetate

TS-4: *Deprotonation of five membered amino pallada(IV)cycle diacetate by dissociated acetate*

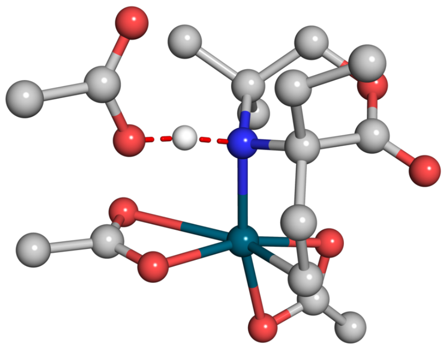

Imaginary frequency at -1226 cm^{-1}

Bonding energy	Internal energy	Entropy	Gibbs energy
-6840.63	280.242	204.445	-6628.50

Atom	X	Y	Z
1 C	0.453284	-0.294082	-2.364452
2 C	-0.971686	-0.599468	-1.868324
3 N	-0.970109	-0.736735	-0.332323
4 C	0.392691	-0.589822	0.334020
5 C	1.461413	-1.370189	-0.453757
6 O	1.431030	-1.247994	-1.807065
7 O	2.314465	-2.071473	0.067133
8 C	0.805172	0.928695	0.458078
9 C	2.322507	1.157084	0.541349
10 C	0.232254	-1.158282	1.763433
11 C	-0.177371	-2.603874	1.783300

12 C	-1.454402	-1.887676	-2.581390
13 C	-1.917675	0.557437	-2.251233
14 O	-2.059463	-4.814330	0.688427
15 Pd	-1.676922	-2.670554	0.310443
16 H	-5.349306	-1.072999	2.464028
17 H	-6.126528	-0.893743	0.849631
18 H	-4.916108	0.303114	1.425713
19 C	-0.849706	-6.538360	-0.522065
20 H	-1.472481	-6.806552	-1.387229
21 H	-1.102807	-7.203699	0.310799
22 H	0.203507	-6.651903	-0.801867
23 H	0.780568	0.721714	-2.127199
24 H	0.515921	-0.437362	-3.447182
25 H	0.312125	1.306944	1.362251
26 H	0.372714	1.508954	-0.359024
27 H	2.778287	0.570010	1.350285
28 H	2.520809	2.220066	0.735170
29 H	2.827178	0.886445	-0.397180
30 H	-0.513951	-0.542598	2.281825
31 H	1.187783	-1.068757	2.308393
32 H	-0.701820	-2.943381	2.681048
33 H	0.560328	-3.314833	1.403590
34 H	-0.735808	-2.706447	-2.486602
35 H	-2.437485	-2.200613	-2.210913
36 H	-1.566417	-1.655890	-3.649261
37 H	-2.935665	0.319279	-1.914779
38 H	-1.927789	0.670596	-3.344346
39 H	-1.609581	1.500414	-1.790066
40 O	-3.030028	-1.713859	1.554742
41 C	-4.071951	-1.514250	0.777632
42 O	-4.087856	-1.896406	-0.415132
43 C	-5.204480	-0.755968	1.424485
44 O	-0.470428	-4.155209	-0.700852
45 C	-1.136808	-5.119546	-0.153504
46 H	-1.726550	0.179420	0.237505
47 O	-2.440802	0.942759	0.889921
48 C	-2.415658	2.248376	0.743441
49 C	-3.481858	2.959179	1.574340
50 O	-1.645645	2.891953	0.004943
51 H	-3.594584	2.487115	2.558040
52 H	-4.446053	2.875985	1.051139
53 H	-3.236906	4.021215	1.686513

Int-8: *Five membered amido pallada(IV)cycle diacetate*

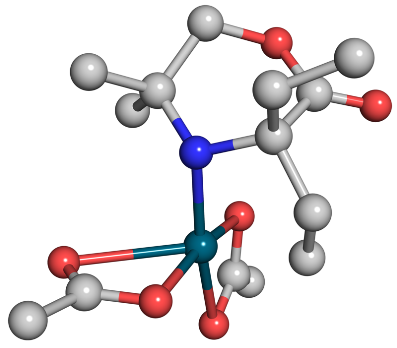

Bonding energy	Internal energy	Entropy	Gibbs energy
-5793.63	240.197	180.277	-5613.49

Atom	X	Y	Z
1 C	-0.007661	-0.871055	-2.952602
2 C	-1.235151	-0.925404	-2.014148
3 N	-0.862485	-0.678015	-0.575982
4 C	0.581716	-0.369380	-0.335056
5 C	1.513990	-1.307063	-1.128121
6 O	1.131994	-1.621752	-2.397514
7 O	2.531900	-1.814425	-0.676072
8 C	0.840562	1.141511	-0.706195
9 C	2.320743	1.557245	-0.662542
10 C	0.826928	-0.541679	1.183929
11 C	0.446620	-1.917896	1.660531
12 C	-1.933393	-2.293339	-2.234820
13 C	-2.228158	0.206206	-2.387888
14 O	-1.787414	-4.193961	1.922690
15 Pd	-1.418859	-2.214986	0.769293
16 H	-4.170624	1.417198	1.064538
17 H	-4.231487	0.833501	2.743337
18 H	-5.461351	0.282558	1.550331
19 C	-1.015919	-6.272771	0.920285
20 H	-1.694943	-6.667241	0.151284
21 H	-1.269192	-6.718325	1.887614
22 H	0.009528	-6.537727	0.633851
23 H	0.322313	0.151526	-3.158760
24 H	-0.221068	-1.362099	-3.906953
25 H	0.245244	1.726500	0.009444
26 H	0.437428	1.366846	-1.696459
27 H	2.761048	1.399499	0.330273
28 H	2.404191	2.625683	-0.905940
29 H	2.919021	0.996201	-1.393921
30 H	0.236144	0.220949	1.706845
31 H	1.890330	-0.379894	1.422099
32 H	0.237214	-2.012081	2.731081
33 H	1.051533	-2.738742	1.265371
34 H	-1.232490	-3.124490	-2.107805
35 H	-2.784757	-2.418623	-1.556138
36 H	-2.321113	-2.318281	-3.263268
37 H	-3.130718	0.102305	-1.771864
38 H	-2.506943	0.133505	-3.450114
39 H	-1.788481	1.193402	-2.201916
40 O	-2.329843	-0.623360	1.828445
41 C	-3.518027	-0.597418	1.281004
42 O	-3.880081	-1.449854	0.430892
43 C	-4.409796	0.551331	1.699087
44 O	-0.659213	-4.069001	0.013984
45 C	-1.164630	-4.780376	0.976905

TS-5: *C-N reductive elimination from five membered amido pallada(IV)cycle diacetate*

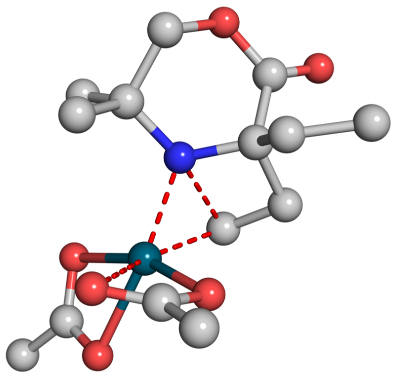

Imaginary frequency at -175 cm^{-1}

Bonding energy	Internal energy	Entropy	Gibbs energy
-5776.83	239.583	181.345	-5597.66

Atom	X	Y	Z
1 C	-0.045379	-0.582996	-3.023388
2 C	-0.828658	-1.577214	-2.133946
3 N	-0.280762	-1.509733	-0.743846
4 C	0.667657	-0.422555	-0.392505
5 C	1.793994	-0.466102	-1.465360
6 O	1.402506	-0.599920	-2.758263
7 O	2.982292	-0.376748	-1.203080
8 C	0.118912	1.036219	-0.277424
9 C	1.185314	2.047668	0.182304
10 C	1.178195	-0.983196	0.948021
11 C	0.701038	-2.410672	0.884139
12 C	-0.609662	-3.013646	-2.671324
13 C	-2.321619	-1.174547	-2.220095
14 O	-2.331107	-3.864141	2.062299
15 Pd	-1.446312	-2.335435	0.721122
16 H	-3.239296	1.872445	1.388678
17 H	-3.166595	1.225930	3.041654
18 H	-4.673620	1.059260	2.070728
19 C	-2.403463	-6.228569	1.526987
20 H	-3.396912	-6.417316	1.096265
21 H	-2.454957	-6.399443	2.608209
22 H	-1.687471	-6.915722	1.062563
23 H	-0.401466	0.445302	-2.911907
24 H	-0.133430	-0.876601	-4.073811
25 H	-0.700697	0.988128	0.447897
26 H	-0.310686	1.364815	-1.230436
27 H	1.615784	1.763774	1.152252
28 H	0.732181	3.042929	0.291070
29 H	2.007613	2.126025	-0.542378
30 H	0.722022	-0.451106	1.790350
31 H	2.270976	-0.937502	1.062877
32 H	0.628945	-2.918749	1.858557
33 H	1.173775	-3.057981	0.146737
34 H	0.463746	-3.247403	-2.687592
35 H	-1.116857	-3.746308	-2.037746
36 H	-1.006934	-3.095950	-3.692951
37 H	-2.953120	-1.922059	-1.726583

38 H	-2.628913	-1.105252	-3.274413
39 H	-2.490737	-0.207967	-1.730151
40 O	-1.831871	-0.536700	1.749211
41 C	-3.082983	-0.255960	1.459604
42 O	-3.790116	-0.984860	0.731863
43 C	-3.578872	1.054657	2.040747
44 O	-1.398592	-4.510047	0.153419
45 C	-2.021992	-4.806096	1.241214

Int-9: *Azetidine*

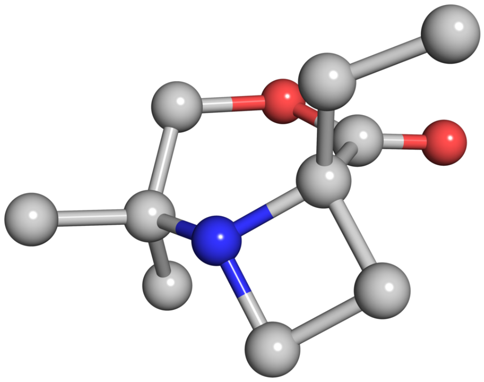

Bonding energy	Internal energy	Entropy	Gibbs energy
-3836.58	168.874	114.987	-3706.01

Atom	X	Y	Z
1 C	-1.566028	0.590426	-0.208308
2 C	-1.918137	-0.797288	0.348948
3 N	-0.609436	-1.485055	0.562675
4 C	0.571921	-1.070098	-0.284912
5 C	0.165733	-0.404067	-1.592960
6 O	-0.875332	0.479474	-1.515304
7 O	0.707622	-0.587958	-2.675863
8 C	1.593796	-0.173246	0.458832
9 C	2.933038	-0.023348	-0.283509
10 C	0.970587	-2.570165	-0.427249
11 C	-0.397619	-2.930187	0.215247
12 C	-2.871633	-1.521379	-0.633618
13 C	-2.611598	-0.622587	1.719343
14 H	-0.923224	1.146392	0.485223
15 H	-2.462961	1.179097	-0.424253
16 H	-3.570212	-0.092602	1.618576
17 H	1.743468	-0.642035	1.443778
18 H	1.154991	0.819010	0.640807
19 H	3.630458	0.584851	0.309333
20 H	2.797669	0.463534	-1.259143
21 H	3.399578	-1.003285	-0.458504
22 H	1.839273	-2.831154	0.188040
23 H	1.127178	-2.913814	-1.453990
24 H	-0.376463	-3.586396	1.096553
25 H	-1.107188	-3.338228	-0.517759
26 H	-2.808906	-1.612109	2.155221
27 H	-2.409187	-1.669898	-1.618313
28 H	-3.782386	-0.921340	-0.770842
29 H	-3.169267	-2.500187	-0.235911

30 H	-1.961064	-0.065794	2.407341

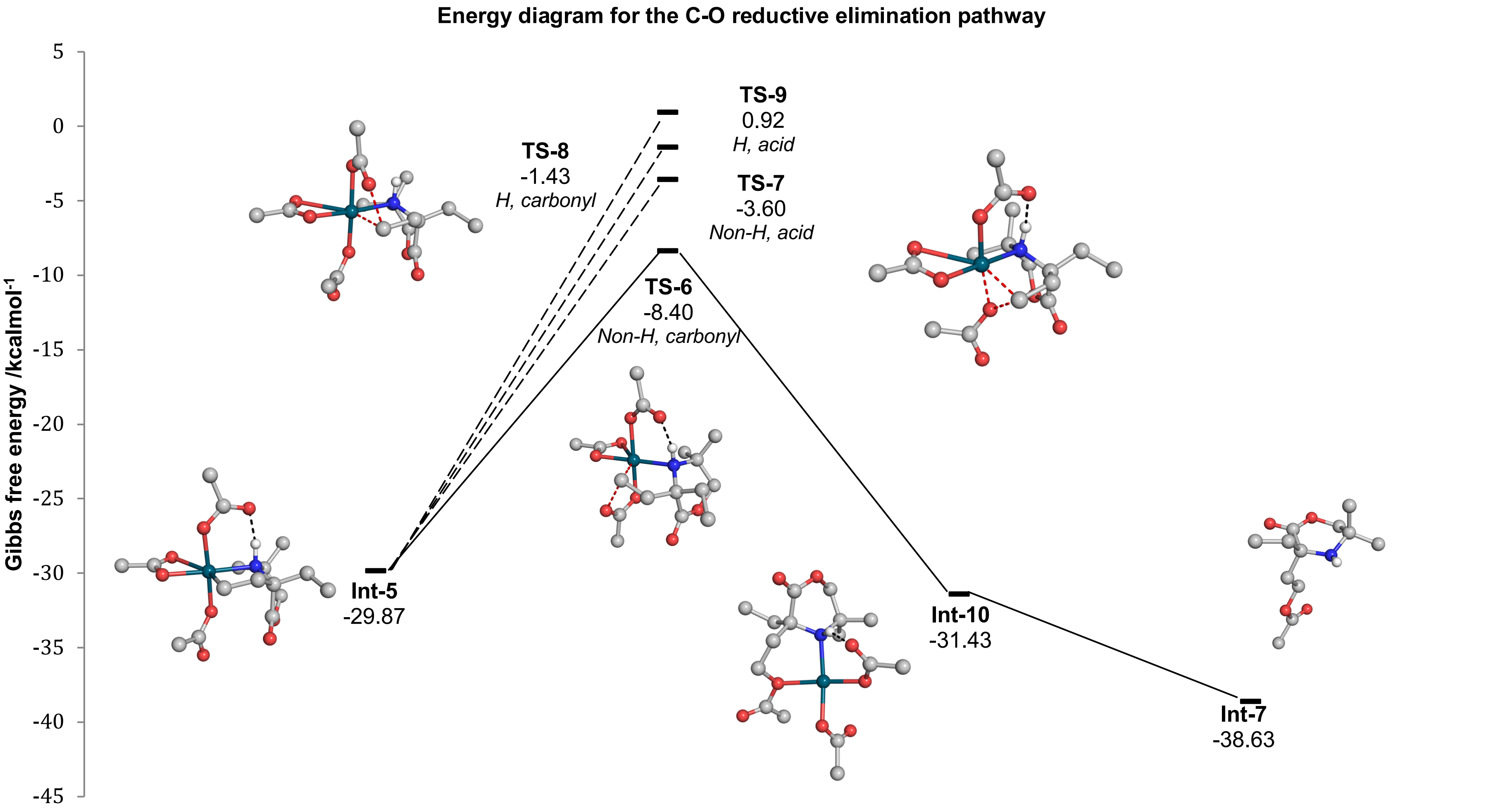

Figure S3: An energy level diagram to show the C–O reductive elimination pathways. The Gibbs energy for Int-1 has been set to zero and all other energies are relative to Int-1. Dotted lines indicate higher energy C–O reductive elimination pathways.

Pathway 2: C-O reductive elimination pathway

TS-6: *C–O reductive elimination of five membered amino pallada(IV)cycle triacetate; non-hydrogen bonded acetate, carbonyl oxygen*

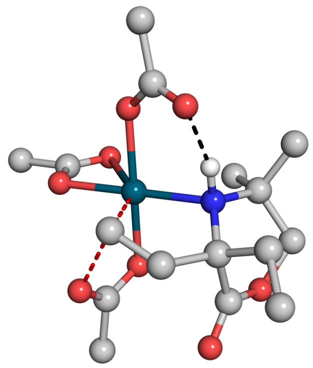

Imaginary frequency -276 cm^{-1}

Bonding energy	Internal energy	Entropy	Gibbs energy
-6837.13	283.159	204.397	-6622.07

Atom	X	Y	Z
1 C	-0.685043	-0.835679	-2.721556
2 C	-1.564896	-0.442939	-1.518095
3 N	-0.772065	-0.647278	-0.211809
4 C	0.731101	-0.835919	-0.332535
5 C	0.994528	-1.953826	-1.368113
6 O	0.170615	-2.000914	-2.448670
7 O	1.899130	-2.766174	-1.277546
8 C	1.426623	0.492241	-0.805655
9 C	2.944523	0.362625	-1.024483
10 C	1.345486	-1.229298	1.052431
11 C	0.421060	-1.645871	2.154762
12 C	-2.822230	-1.333674	-1.505443
13 C	-1.968170	1.041462	-1.628461
14 O	-4.133498	-3.436264	1.000473
15 Pd	-1.664881	-2.078289	1.152565
16 H	-4.302928	-5.834228	2.430257
17 H	-5.379156	-4.577329	3.074617
18 H	-3.847074	-4.969586	3.927117
19 H	-0.929608	0.188725	0.405520
20 O	-2.467551	-3.431052	2.530851
21 C	-3.630431	-3.834238	2.070772
22 C	-4.328061	-4.865581	2.948417
23 H	-0.042716	-0.018354	-3.061268
24 H	-1.313750	-1.149850	-3.559055
25 H	1.210743	1.244028	-0.031306
26 H	0.974168	0.856715	-1.730590
27 H	3.469909	0.054993	-0.111817
28 H	3.346989	1.335130	-1.337539
29 H	3.172446	-0.369983	-1.810247
30 H	1.846345	-0.338220	1.475970
31 H	2.120267	-1.980716	0.884033
32 H	-0.255307	-0.859942	2.503462
33 H	0.898040	-2.124225	3.003450
34 H	-2.555383	-2.397273	-1.520779

35 H	-3.449599	-1.131503	-0.628580
36 H	-3.420138	-1.114328	-2.401371
37 H	-2.655192	1.306383	-0.814510
38 H	-2.481423	1.213979	-2.584505
39 H	-1.095979	1.703827	-1.577149
40 O	-2.797463	-0.590592	2.159082
41 C	-2.462968	0.667293	2.241242
42 O	-1.449546	1.194125	1.714336
43 C	-3.428229	1.521265	3.050664
44 O	-0.879370	-3.694087	0.081353
45 C	0.084089	-4.244989	0.741387
46 C	0.652002	-5.517634	0.147195
47 O	0.582069	-3.755197	1.792819
48 H	-4.199330	0.917360	3.538465
49 H	-3.902153	2.246585	2.375475
50 H	-2.858928	2.083931	3.801550
51 H	1.183834	-6.092002	0.911767
52 H	1.359461	-5.220604	-0.639210
53 H	-0.142594	-6.118593	-0.307738

TS-7: *C-O reductive elimination of five membered amino pallada(IV)cycle triacetate; non-hydrogen bonded acetate, acid oxygen*

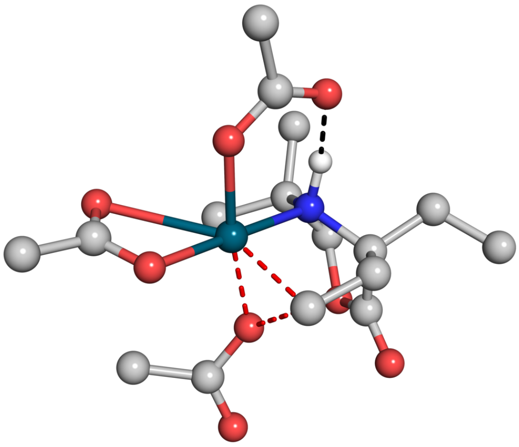

Imaginary frequency -252 cm^{-1}

Bonding energy	Internal energy	Entropy	Gibbs energy
-6832.73	283.39	203.885	-6617.26

Atom	X	Y	Z
1 C	-0.443892	-0.889336	-2.757229
2 C	-1.528199	-0.751941	-1.672151
3 N	-0.856438	-0.700307	-0.290178
4 C	0.670803	-0.696501	-0.243681
5 C	1.250688	-1.713363	-1.235883
6 O	0.583693	-1.884840	-2.411873
7 O	2.287001	-2.321219	-1.034706
8 C	1.198044	0.746086	-0.569116
9 C	2.729656	0.887618	-0.571835
10 C	1.067769	-1.055268	1.208130
11 C	0.468684	-2.335668	1.758264
12 C	-2.489199	-1.954258	-1.773191
13 C	-2.297567	0.568154	-1.874728

14 O	-4.136811	-3.110597	1.313679
15 Pd	-1.614471	-2.081003	1.192905
16 H	-4.356631	-5.579518	2.718590
17 H	-5.375057	-4.263144	3.343376
18 H	-3.850527	-4.708027	4.190698
19 H	-1.161723	0.171015	0.215055
20 O	-2.337567	-3.435757	2.632406
21 C	-3.597585	-3.631742	2.313760
22 C	-4.339238	-4.599012	3.216381
23 H	0.055762	0.059819	-2.973057
24 H	-0.889778	-1.263247	-3.683521
25 H	0.756227	1.414177	0.184470
26 H	0.808140	1.071319	-1.538576
27 H	3.158906	0.674116	0.415177
28 H	2.993926	1.919976	-0.839504
29 H	3.196897	0.214446	-1.302239
30 H	0.767218	-0.209003	1.839527
31 H	2.161617	-1.158841	1.255653
32 H	0.181682	-2.262439	2.815587
33 H	1.042443	-3.235422	1.578060
34 H	-1.933544	-2.896933	-1.760989
35 H	-3.223728	-1.957174	-0.957461
36 H	-3.041156	-1.885802	-2.720583
37 H	-3.097379	0.656611	-1.127399
38 H	-2.755817	0.583684	-2.872984
39 H	-1.633378	1.437096	-1.780218
40 O	-2.563090	-0.628974	2.381167
41 C	-2.407916	0.662578	2.259093
42 O	-1.696352	1.251909	1.409932
43 C	-3.218757	1.466874	3.266481
44 O	-0.384835	-3.367543	0.192327
45 C	-0.510092	-4.677287	-0.088721
46 C	-1.806737	-5.384853	0.216100
47 O	0.461613	-5.240876	-0.602944
48 H	-3.534759	0.854823	4.116836
49 H	-4.111238	1.854074	2.755011
50 H	-2.623992	2.321754	3.608718
51 H	-1.858074	-5.572217	1.296329
52 H	-1.834385	-6.340211	-0.317915
53 H	-2.671351	-4.770148	-0.055518

TS-8: *C-O reductive elimination of five membered amino pallada(IV)cycle triacetate; hydrogen bonded acetate, carbonyl oxygen*

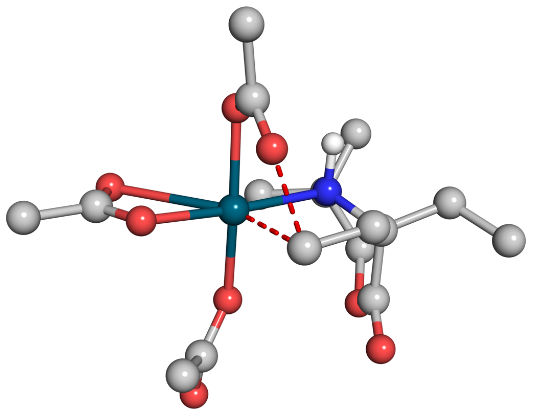

Imaginary frequency at -262 cm^{-1}, additional low intensity negative frequency at -38 cm^{-1} corresponding to molecular flexing

Bonding energy	Internal energy	Entropy	Gibbs energy
-6831.00	282.690	200.483	-6615.10

Atom	X	Y	Z
1 C	-0.348042	-1.182931	-2.867977
2 C	-1.481903	-0.675690	-1.943371
3 N	-0.938302	-0.554257	-0.501762
4 C	0.575291	-0.437308	-0.350002
5 C	1.176753	-1.682924	-1.029153
6 O	0.623838	-2.069437	-2.204616
7 O	2.107587	-2.317150	-0.560455
8 C	1.146107	0.871845	-0.995505
9 C	2.668765	1.042190	-0.846110
10 C	0.899062	-0.407412	1.162551
11 C	0.347362	-1.526828	1.982835
12 C	-2.665986	-1.661583	-1.967401
13 C	-1.964019	0.711790	-2.417303
14 O	-4.199435	-3.328722	0.999379
15 Pd	-1.724151	-1.982652	0.974409
16 H	-3.849975	-5.635242	2.851794
17 H	-5.368918	-4.698377	2.874762
18 H	-4.075676	-4.375920	4.084745
19 H	-1.349493	0.303010	-0.099477
20 O	-2.494876	-3.194348	2.479665
21 C	-3.663156	-3.650595	2.077967
22 C	-4.287856	-4.645306	3.043317
23 H	0.212517	-0.360793	-3.323217
24 H	-0.769210	-1.799071	-3.666785
25 H	0.621308	1.715197	-0.519916
26 H	0.901368	0.908625	-2.058903
27 H	2.966853	1.189446	0.199099
28 H	2.987140	1.925533	-1.415306
29 H	3.208992	0.170353	-1.239262
30 H	0.608523	0.568718	1.572346
31 H	1.993901	-0.480793	1.281671
32 H	0.646177	-1.555534	3.022730
33 H	0.477249	-2.508766	1.527847
34 H	-2.333698	-2.685932	-1.775356
35 H	-3.429537	-1.380817	-1.229341
36 H	-3.133358	-1.620702	-2.961259
37 H	-2.832184	1.027750	-1.821929
38 H	-2.282162	0.648487	-3.466935
39 H	-1.183024	1.475978	-2.340238
40 O	-2.706546	-0.306586	1.804464
41 C	-2.084006	0.080812	2.869122
42 O	-0.967168	-0.384561	3.231294
43 C	-2.774806	1.136897	3.709597
44 O	-1.012422	-3.609374	-0.153121
45 C	-0.556233	-4.799810	0.182373
46 C	-0.087671	-5.074130	1.600730
47 O	-0.468204	-5.677287	-0.699819
48 H	-3.461486	1.733876	3.100424
49 H	-2.032008	1.775019	4.198828
50 H	-3.354522	0.618963	4.486318
51 H	0.912063	-4.634741	1.733436

52 H	-0.005495	-6.155877	1.755724
53 H	-0.758093	-4.634028	2.344477

TS-9: *C-O reductive elimination of five membered amino pallada(IV)cycle triacetate; hydrogen bonded acetate, acid oxygen*

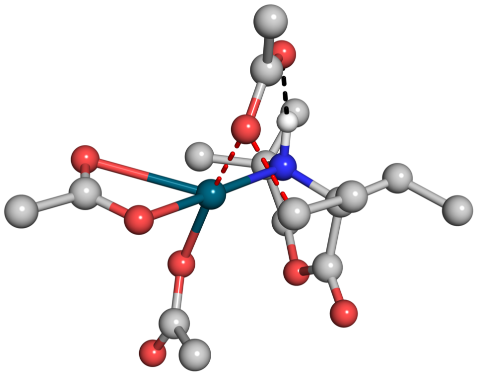

Imaginary frequency at -271cm^{-1}

Bonding energy	Internal energy	Entropy	Gibbs energy
-6828.20	283.365	203.846	-6612.75

Atom	X	Y	Z
1 C	-0.288075	-1.353375	-2.672255
2 C	-1.393285	-0.653278	-1.832172
3 N	-0.927804	-0.594396	-0.382846
4 C	0.569121	-0.479243	-0.140947
5 C	1.184192	-1.753016	-0.739413
6 O	0.655909	-2.195235	-1.905831
7 O	2.089782	-2.379594	-0.210443
8 C	1.231278	0.779176	-0.795954
9 C	2.724168	0.953061	-0.457738
10 C	0.762743	-0.402516	1.394439
11 C	0.071298	-1.482228	2.176880
12 C	-2.736216	-1.412153	-1.912361
13 C	-1.641831	0.782645	-2.362798
14 O	-4.222723	-3.148944	1.214793
15 Pd	-1.665521	-2.123450	1.031135
16 H	-4.111328	-5.781576	2.499499
17 H	-5.350847	-4.625172	3.050136
18 H	-3.832112	-4.851595	3.990741
19 H	-1.403390	0.213963	0.080899
20 O	-2.357997	-3.507531	2.437229
21 C	-3.619132	-3.735620	2.135405
22 C	-4.272542	-4.809967	2.987880
23 H	0.316565	-0.623152	-3.220527
24 H	-0.732780	-2.049934	-3.387674
25 H	0.661126	1.657937	-0.460628
26 H	1.134023	0.725126	-1.883620
27 H	2.881871	1.188635	0.601798
28 H	3.129347	1.783305	-1.051493
29 H	3.297962	0.048534	-0.701198
30 H	0.463766	0.598292	1.730587
31 H	1.833258	-0.522493	1.625073
32 H	0.057037	-1.356828	3.254639
33 H	0.464826	-2.478875	1.933798

34 H	-2.615037	-2.478323	-1.712600
35 H	-3.458886	-0.984769	-1.203229
36 H	-3.147951	-1.288472	-2.923193
37 H	-2.459736	1.249048	-1.796854
38 H	-1.948336	0.723032	-3.416199
39 H	-0.760899	1.425106	-2.301009
40 O	-1.730488	-0.723342	2.569002
41 C	-2.155529	0.532346	2.410950
42 O	-2.243303	1.140013	1.330931
43 C	-2.505366	1.169865	3.740266
44 O	-1.402254	-3.653800	-0.350441
45 C	-0.722203	-4.780962	-0.223480
46 C	0.128316	-5.023727	1.004721
47 O	-0.780827	-5.609042	-1.151793
48 H	-1.669882	1.048446	4.441535
49 H	-3.375738	0.655845	4.169468
50 H	-2.733066	2.230582	3.597877
51 H	0.993584	-4.348036	0.978086
52 H	0.492918	-6.057091	1.000739
53 H	-0.440657	-4.831550	1.921199

Int-10: *2-(3-ethyl-5,5-dimethyl-2-oxomorpholin-3-yl)ethyl acetate palladium diacetate (Int-G)*

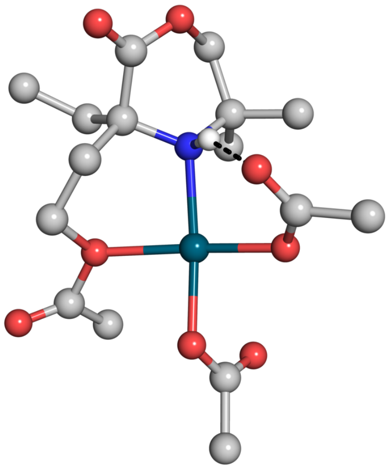

Bonding energy	Internal energy	Entropy	Gibbs energy
-6862.05	285.480	205.703	-6645.10

Atom	X	Y	Z
1 C	0.378057	-0.992650	-2.974920
2 C	-0.718612	-1.584799	-2.040871
3 N	-0.178832	-1.607531	-0.608372
4 C	0.895536	-0.561360	-0.279786
5 C	2.077872	-0.916911	-1.218761
6 O	1.749794	-1.253692	-2.499181
7 O	3.248152	-0.947327	-0.878120
8 C	0.473318	0.921006	-0.524753
9 C	1.615254	1.926989	-0.280595
10 C	1.324184	-0.793546	1.198783
11 C	0.512297	-0.062564	2.270311
12 C	-1.007497	-3.033546	-2.482263
13 C	-2.003477	-0.743257	-2.126951
14 O	-4.503164	-2.416225	1.625706
15 Pd	-1.509572	-2.068772	1.059382
16 H	-4.580961	-4.149441	3.992673

17 H	-5.571724	-2.668398	3.985609
18 H	-3.992035	-2.706609	4.847877
19 H	0.305597	-2.521955	-0.494774
20 O	-2.554274	-2.511239	2.797326
21 C	-3.861157	-2.623144	2.669787
22 C	-4.544507	-3.050762	3.966225
23 H	0.271955	0.085595	-3.120728
24 H	0.341126	-1.477686	-3.954042
25 H	-0.386726	1.143974	0.115572
26 H	0.125831	1.058796	-1.548920
27 H	2.006586	1.884658	0.742898
28 H	1.240771	2.943821	-0.456748
29 H	2.454641	1.751377	-0.966977
30 H	2.358032	-0.445572	1.320198
31 H	1.342742	-1.873739	1.397529
32 H	0.747631	1.003967	2.320909
33 H	0.672281	-0.514282	3.254344
34 H	-0.097439	-3.646553	-2.443229
35 H	-1.778626	-3.490239	-1.855554
36 H	-1.371651	-3.018938	-3.518068
37 H	-2.805218	-1.231768	-1.557802
38 H	-2.323132	-0.666660	-3.175645
39 H	-1.864718	0.268621	-1.733785
40 O	-0.956986	-0.138227	1.963060
41 C	-1.797970	0.564009	2.848351
42 O	-1.348596	1.084337	3.847824
43 C	-3.213320	0.645134	2.359026
44 O	-1.810511	-4.024031	0.469932
45 C	-0.733505	-4.738892	0.238678
46 C	-1.044864	-6.191041	-0.088583
47 O	0.441599	-4.309532	0.211365
48 H	-3.393090	1.686506	2.055758
49 H	-3.887268	0.414526	3.191778
50 H	-3.423959	-0.024471	1.522422
51 H	-0.258852	-6.833520	0.324920
52 H	-1.035402	-6.297118	-1.183193
53 H	-2.027111	-6.495730	0.286931

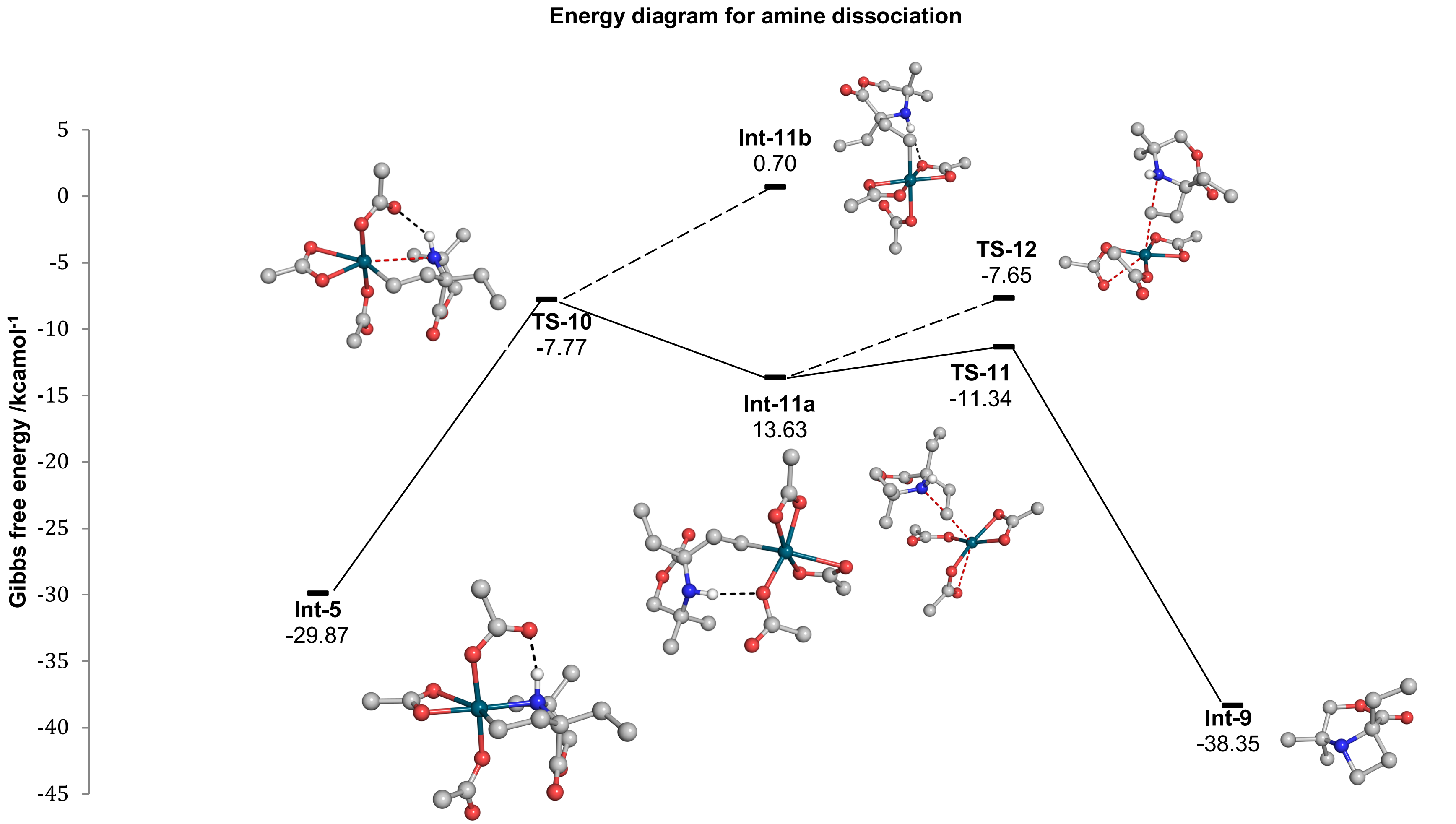

Figure S4: An energy level diagram to show the amine dissociation pathway. The Gibbs energy for Int-1 has been set to zero and all other energies are relative to Int-1.

Pathway 3: Amine dissociation pathway

TS-10: *Dissociation of nitrogen from five membered amino pallada(IV)cycle triacetate*

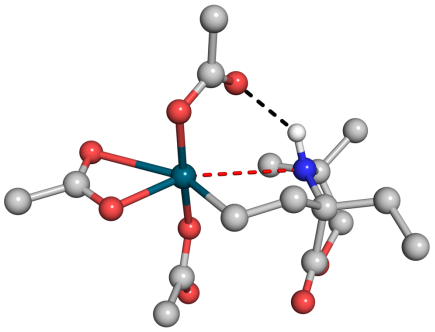

Imaginary frequency at -68 cm^{-1}

Bonding energy	Internal energy	Entropy	Gibbs energy
-6833.73	282.160	209.704	-6621.43

Atom	X	Y	Z
1 C	0.195120	-0.933185	-3.057306
2 C	-0.971351	-0.280062	-2.287885
3 N	-0.605136	-0.223599	-0.861504
4 C	0.764593	-0.432526	-0.387707
5 C	1.279058	-1.736692	-1.031870
6 O	0.853158	-2.013724	-2.292786
7 O	2.029566	-2.519915	-0.470767
8 C	1.731869	0.763380	-0.765229
9 C	3.229003	0.513462	-0.519089
10 C	0.711650	-0.542384	1.158217
11 C	0.159093	-1.790290	1.792351
12 C	-2.250862	-1.140250	-2.418135
13 C	-1.223889	1.139632	-2.855415
14 O	-4.092577	-3.406248	1.989053
15 Pd	-1.890369	-2.152029	1.428972
16 H	-3.749958	-6.074864	3.013906
17 H	-4.750602	-4.982145	4.004583
18 H	-3.044149	-5.318504	4.467915
19 H	-1.183059	0.396153	-0.287353
20 O	-2.032586	-3.702319	2.835381
21 C	-3.312865	-4.009909	2.755339
22 C	-3.736503	-5.167452	3.633248
23 H	0.969146	-0.207842	-3.331375
24 H	-0.165880	-1.422003	-3.966086
25 H	1.381282	1.628964	-0.181911
26 H	1.583195	1.019626	-1.818818
27 H	3.444138	0.303830	0.536851
28 H	3.799663	1.406553	-0.808570
29 H	3.598283	-0.332528	-1.114060
30 H	0.206510	0.356735	1.540694
31 H	1.742359	-0.492918	1.556809
32 H	0.133881	-1.735081	2.885158
33 H	0.587653	-2.721373	1.426303
34 H	-2.043388	-2.171378	-2.114935
35 H	-3.035772	-0.736274	-1.764097
36 H	-2.610548	-1.120388	-3.456868
37 H	-2.089972	1.589871	-2.350077

38 H	-1.446985	1.083538	-3.930571
39 H	-0.353104	1.790021	-2.706489
40 O	-2.293304	-0.750824	2.948727
41 C	-2.673366	0.339686	2.335292
42 O	-2.708847	0.404543	1.079673
43 C	-3.077529	1.496785	3.214446
44 O	-1.655763	-3.407281	-0.193657
45 C	-0.980626	-4.519918	-0.429083
46 C	-0.079053	-5.127498	0.621278
47 O	-1.096621	-5.030378	-1.558723
48 H	-2.698437	1.376722	4.234850
49 H	-4.175033	1.542546	3.245182
50 H	-2.710402	2.433444	2.777605
51 H	0.922368	-4.689522	0.505629
52 H	0.002940	-6.206114	0.442451
53 H	-0.435942	-4.941123	1.637773

TS-11a: *Dissociated amine five membered pallada(IV)cycle triacetate, syn geometry*

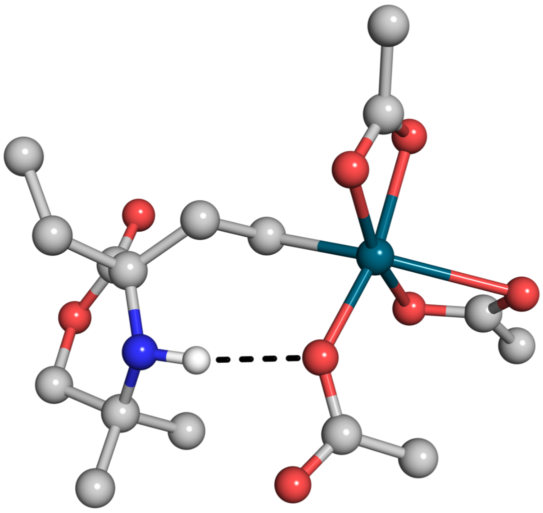

Bonding energy	Internal energy	Entropy	Gibbs energy
-6839.25	283.226	213.926	-6627.29

Atom	X	Y	Z
1 C	1.380094	-1.391898	-2.739669
2 C	-0.097588	-1.559981	-2.382219
3 N	-0.410235	-0.573735	-1.312342
4 C	0.655470	-0.247711	-0.328307
5 C	1.839728	-1.233475	-0.359688
6 O	2.244254	-1.691403	-1.571905
7 O	2.456155	-1.584077	0.640878
8 C	1.219143	1.185916	-0.656350
9 C	2.468855	1.634260	0.118306
10 C	0.039217	-0.220089	1.105052
11 C	-0.235746	-1.592256	1.658742
12 C	-0.384600	-3.021132	-1.946815
13 C	-0.949770	-1.206682	-3.621923
14 O	-3.903480	-3.586166	3.517170
15 Pd	-2.232705	-1.782548	2.436649
16 H	-3.780575	-6.165700	2.197530

17 H	-3.545623	-6.153931	3.963889
18 H	-2.128990	-6.278801	2.858111
19 H	-1.273633	-0.846488	-0.834467
20 O	-2.062641	-3.836294	2.247994
21 C	-3.058169	-4.322480	2.967496
22 C	-3.117242	-5.831358	3.008408
23 H	1.602234	-0.375015	-3.084178
24 H	1.695731	-2.112254	-3.500574
25 H	0.381524	1.879562	-0.484077
26 H	1.425481	1.214519	-1.734100
27 H	2.300147	1.656481	1.203222
28 H	2.751084	2.648851	-0.196625
29 H	3.324502	0.972070	-0.075053
30 H	-0.880774	0.375447	1.009567
31 H	0.689972	0.322724	1.798763
32 H	0.311400	-1.880830	2.558735
33 H	-0.328370	-2.402546	0.933421
34 H	0.220433	-3.314043	-1.079052
35 H	-1.445227	-3.122755	-1.686314
36 H	-0.162028	-3.723913	-2.763081
37 H	-2.012997	-1.268654	-3.354927
38 H	-0.753915	-1.907068	-4.446237
39 H	-0.731333	-0.183286	-3.955707
40 O	-1.545151	-1.383090	4.443389
41 C	-1.787163	-0.122841	4.289651
42 O	-2.244292	0.252464	3.141945
43 C	-1.520406	0.856113	5.380761
44 O	-2.829962	-1.476912	0.468067
45 C	-3.662989	-2.140723	-0.331695
46 C	-4.378084	-3.372042	0.156344
47 O	-3.789896	-1.703508	-1.486578
48 H	-0.438805	1.048438	5.421873
49 H	-1.828956	0.434192	6.344258
50 H	-2.046011	1.796800	5.185094
51 H	-5.161549	-3.645631	-0.558179
52 H	-4.809825	-3.211949	1.150032
53 H	-3.652708	-4.192737	0.230636

TS-11b: *Dissociated amine five membered pallada(IV)cycle triacetate, anti geometry*

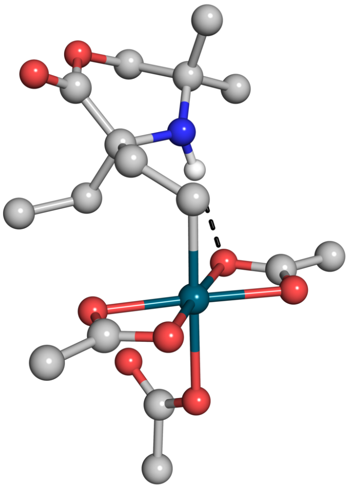

Bonding energy	Internal energy	Entropy	Gibbs energy

-6829.64	282.732	212.289	-6617.63

Atom	X	Y	Z
1 C	0.998582	-0.610660	-2.932676
2 C	0.803758	-2.030168	-2.378691
3 N	0.195468	-1.948113	-1.033299
4 C	0.580904	-0.826088	-0.143955
5 C	1.775839	-0.022862	-0.689927
6 O	1.869979	0.176778	-2.033558
7 O	2.601538	0.515466	0.036058
8 C	-0.601568	0.218572	-0.040950
9 C	-0.319597	1.513427	0.738150
10 C	0.987910	-1.407640	1.254467
11 C	0.223975	-2.612882	1.744777
12 C	2.159462	-2.771367	-2.276112
13 C	-0.158928	-2.804405	-3.297624
14 O	-2.333910	-4.195622	1.629897
15 Pd	-1.817932	-2.266735	2.337639
16 H	-3.233022	-5.685831	-0.256996
17 H	-2.475537	-4.614262	-1.483174
18 H	-4.135933	-4.292199	-0.943548
19 H	-0.823680	-2.017398	-1.074178
20 O	-2.447924	-2.505976	0.249454
21 C	-2.630494	-3.760477	0.448273
22 C	-3.151906	-4.655914	-0.619196
23 H	0.038436	-0.088328	-3.031390
24 H	1.523902	-0.602227	-3.894249
25 H	-1.458258	-0.291071	0.413867
26 H	-0.901159	0.471516	-1.069067
27 H	-0.008357	1.305933	1.768106
28 H	-1.243556	2.106266	0.784899
29 H	0.455705	2.123513	0.255976
30 H	1.029620	-0.613431	2.008020
31 H	2.027659	-1.776772	1.153907
32 H	0.621703	-3.008299	2.686197
33 H	0.095762	-3.386618	0.982207
34 H	2.846693	-2.267556	-1.584278
35 H	1.986038	-3.788645	-1.902702
36 H	2.646968	-2.825089	-3.259767
37 H	-0.353434	-3.799162	-2.873434
38 H	0.281288	-2.937555	-4.294644
39 H	-1.115811	-2.273073	-3.407034
40 O	-1.182899	-2.191783	4.386331
41 C	-1.069031	-0.906365	4.319191
42 O	-1.301641	-0.380717	3.166878
43 C	-0.700700	-0.082067	5.499439
44 O	-3.976219	-1.940904	3.005115
45 C	-4.507922	-0.916863	2.406018
46 C	-5.946277	-0.604175	2.840898
47 O	-3.943723	-0.203997	1.540549
48 H	0.196853	-0.499855	5.973030
49 H	-1.520877	-0.118222	6.229505
50 H	-0.523989	0.955337	5.195907
51 H	-6.343766	0.249889	2.281663
52 H	-5.964083	-0.379425	3.916311
53 H	-6.584059	-1.483279	2.675948

TS-11: *Nucleophilic attack of dissociated amine onto palladated carbon of dissociated amine five membered pallada(IV)cycle triacetate, syn geometry*

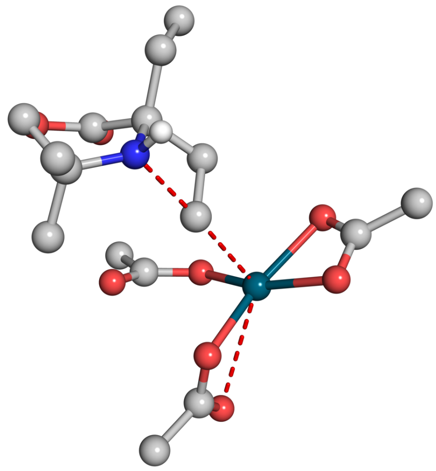

Imaginary frequency at -327cm^{-1}

Bonding energy	Internal energy	Entropy	Gibbs energy
-6836.94	282.929	213.116	-6625.01

Atom	X	Y	Z
1 C	2.736832	-2.126349	-0.446760
2 C	1.582861	-3.133465	-0.333997
3 N	0.313416	-2.400404	-0.690366
4 C	0.252436	-0.882215	-0.559133
5 C	1.301399	-0.365500	0.437182
6 O	2.521614	-0.965907	0.441982
7 O	1.100896	0.595325	1.159732
8 C	0.462095	-0.166676	-1.921034
9 C	0.239098	1.353664	-1.863849
10 C	-1.182830	-0.766285	0.005597
11 C	-1.384638	-2.193438	0.501902
12 C	1.479665	-3.685741	1.101253
13 C	1.790470	-4.272336	-1.347295
14 O	-4.242313	-0.424989	0.732852
15 Pd	-3.278447	-1.831236	2.084933
16 H	-5.859512	-0.101857	-1.220135
17 H	-6.827145	-1.505595	-0.659941
18 H	-5.515184	-1.758130	-1.826428
19 H	-0.015679	-2.669804	-1.626180
20 O	-4.653323	-2.594968	0.608179
21 C	-4.873800	-1.397739	0.172328
22 C	-5.826483	-1.164229	-0.957000
23 H	2.863035	-1.764029	-1.473447
24 H	3.676426	-2.566220	-0.099444
25 H	-0.250887	-0.623580	-2.626290
26 H	1.471171	-0.383232	-2.298982
27 H	-0.767848	1.595251	-1.497915
28 H	0.348708	1.778318	-2.870238
29 H	0.967238	1.840454	-1.202882
30 H	-1.899147	-0.519839	-0.786343
31 H	-1.267712	-0.016195	0.796971
32 H	-0.900719	-2.507993	1.420669

33 H	-2.023865	-2.896053	-0.026275
34 H	1.326759	-2.887787	1.836005
35 H	0.637982	-4.386771	1.170613
36 H	2.404794	-4.221319	1.353548
37 H	0.935246	-4.961256	-1.314326
38 H	2.695458	-4.839224	-1.093774
39 H	1.896297	-3.882989	-2.369565
40 O	-0.230658	-1.764518	3.352648
41 C	-0.907147	-0.758184	3.602633
42 O	-2.164612	-0.543472	3.252818
43 C	-0.302466	0.436840	4.337878
44 O	-2.622867	-3.572345	2.964766
45 C	-2.856564	-3.696692	4.254541
46 C	-2.187878	-4.938342	4.837961
47 O	-3.511268	-2.905394	4.953391
48 H	0.303273	0.070903	5.175725
49 H	-1.061294	1.139841	4.697790
50 H	0.361412	0.949447	3.628985
51 H	-2.707586	-5.244761	5.752129
52 H	-1.149387	-4.675605	5.085423
53 H	-2.167966	-5.762792	4.115673

TS-12: *Higher energy nucleophilic attack of dissociated amine onto palladated carbon of dissociated amine five membered pallada(IV)cycle triacetate, syn geometry*

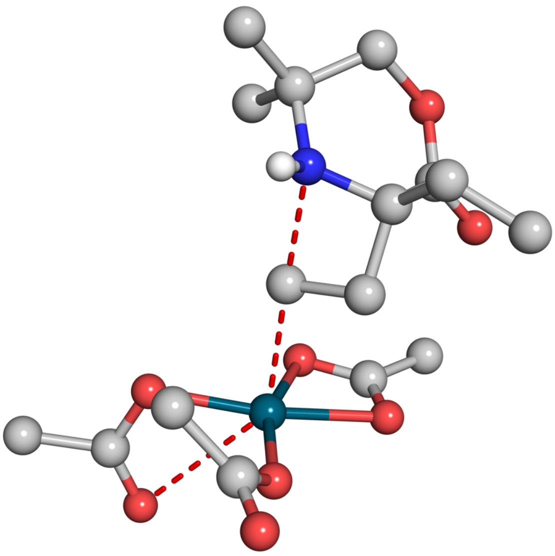

Imaginary frequency at -209cm^{-1}

Bonding energy	Internal energy	Entropy	Gibbs energy
-6833.45	282.955	212.577	-6621.32

Atom	X	Y	Z
1 C	2.683161	-1.747302	-1.618911
2 C	1.467821	-2.685264	-1.603894
3 N	0.247151	-1.860727	-1.286547
4 C	0.390674	-0.433514	-0.795468
5 C	1.660605	-0.321680	0.070476
6 O	2.773951	-0.967022	-0.366063

7 O	1.707563	0.333421	1.098428
8 C	0.414122	0.633985	-1.918465
9 C	0.332900	2.075591	-1.386162
10 C	-0.871036	-0.396567	0.090879
11 C	-0.967660	-1.866276	0.467675
12 C	1.627674	-3.767244	-0.516012
13 C	1.296458	-3.322010	-2.994597
14 O	-4.281181	-3.527469	3.780016
15 Pd	-2.365794	-1.784072	2.416948
16 H	-5.382377	-5.307205	1.708223
17 H	-4.815018	-6.019586	3.241745
18 H	-3.773643	-6.063771	1.774500
19 H	-0.422507	-1.880235	-2.066051
20 O	-3.075237	-3.645872	1.859607
21 C	-3.935514	-4.120586	2.743206
22 C	-4.501567	-5.475473	2.344351
23 H	2.653220	-1.050472	-2.463559
24 H	3.616001	-2.316922	-1.653090
25 H	-0.448976	0.424811	-2.570757
26 H	1.316476	0.514292	-2.533050
27 H	-0.589907	2.237907	-0.812673
28 H	0.338473	2.780291	-2.227944
29 H	1.185208	2.309525	-0.734564
30 H	-1.743513	-0.088608	-0.498156
31 H	-0.769050	0.272624	0.948471
32 H	-0.180842	-2.309819	1.070426
33 H	-1.703460	-2.541707	0.048130
34 H	1.831324	-3.318527	0.464295
35 H	0.711581	-4.367147	-0.448045
36 H	2.466066	-4.430158	-0.767871
37 H	0.420703	-3.985961	-2.998187
38 H	2.179614	-3.923092	-3.247573
39 H	1.162425	-2.552171	-3.767048
40 O	-0.591430	-2.237804	3.561565
41 C	-0.367036	-0.976485	3.743925
42 O	-1.216577	-0.143042	3.250142
43 C	0.862400	-0.500310	4.442052
44 O	-3.911299	-0.710018	1.546581
45 C	-4.588012	-0.824376	0.414273
46 C	-4.330536	-1.978268	-0.533106
47 O	-5.419297	0.061500	0.137769
48 H	1.585238	-0.190546	3.673078
49 H	1.298514	-1.300217	5.049607
50 H	0.627035	0.372163	5.062992
51 H	-5.209310	-2.122399	-1.172232
52 H	-4.087555	-2.902990	-0.003245
53 H	-3.486637	-1.715847	-1.187491

References and notes

1) G. te Velde, F.M. Bickelhaupt, E.J. Baerends, C.Fonseca Guerra, S.J.A. van Gisbergen, J.G. Snijders, T. Ziegler, Chemistry with ADF, *J. Comput. Chem.* **22**, 931-967 (2001)

2) G. Fonseca Guerra, J.G. Snijders, G. te Velde, E.J. Baerends, Towards an order-N DFT method, *Theor. Chem. Acc.* **99**, 391-403 (1998)

3) ADF2016, SCM, Theoretical Chemistry, Vrije Universiteit, Amsterdam, The Netherlands, http://www.scm.com

4) J. Wassenaar, E. Jansen, W.-J. van Zeist, F.M. Bickelhaupt, M.A. Siegler, A.L. Spek, J.N.H. Reek, Catalyst selection based on intermediate stability measured by mass spectroscopy, *Nat. Chem* **2**, 417-421 (2010)

5) G.T. de Jong, F.M. Bickelhaupt, Transition-State Energy and Position along the Reaction Coordinate in an Extended Activation Strain Model, *ChemPhysChem* **8**, 1170-1181 (2007)

6) A.P. Smalley, M.J. Gaunt, Mechanistic Insights into the Palladium-Catalyzed Aziridination of Aliphatic Amines by C-H Activation, *J. Am. Chem. Soc.* **137**, 10632-10641 (2015)

7) D. Willcox, B.G.N. Chappell, K.F. Hogg, J. Calleja, A.P. Smalley, M.J. Gaunt, A general catalytic β-C–H carbonylation of aliphatic amines to β-lactams, *Science* **354**, 851-857 (2016)

8) A.K. Rappe, C.J. Casewit, K.S. Colwell, W.A. GoddardIII, W.M. Skiff, UFF, a full periodic table force field for molecular mechanics and molecular dynamics simulations, *J. Am. Chem. Soc.* **114**, 10024-10035 (1992)
